# The Complex Reactivity of [(salen)Fe]_2_(μ-O) with HBpin and Its Implications in Catalysis

**DOI:** 10.1021/acscatal.3c02898

**Published:** 2023-08-23

**Authors:** Thomas M. Hood, Samantha Lau, Martin Diefenbach, Leah Firmstone, Mary Mahon, Vera Krewald, Ruth L. Webster

**Affiliations:** † Department of Chemistry, University of Bath, Claverton Down, Bath, United Kingdom BA2 7AY; ‡ Department of Chemistry, TU Darmstadt, Peter-Grünberg-Str. 4, 64287 Darmstadt, Germany

**Keywords:** iron, cyclotrimerization, mechanism, salen, ligand reduction

## Abstract

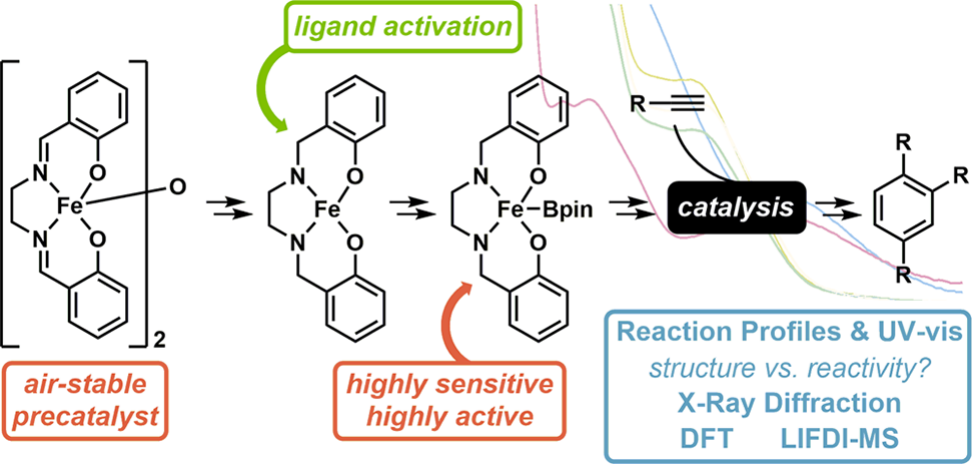

We report a detailed study into the method of precatalyst activation during alkyne cyclotrimerization. During these studies we have prepared a homologous series of Fe(III)-μ-oxo(salen) complexes and use a range of techniques including UV–vis, reaction monitoring studies, single crystal X-ray diffraction, NMR spectroscopy, and LIFDI mass spectrometry to provide experimental evidence for the nature of the on-cycle iron catalyst. These data infer the likelihood of ligand reduction, generating an iron(salan)-boryl complex as a key on-cycle intermediate. We use DFT studies to interrogate spin states, connecting this to experimentally identified diamagnetic and paramagnetic species. The extreme conformational flexibility of the salan system appears connected to challenges associated with crystallization of likely on-cycle species.

## 1 Introduction

Our precious-metal supply is rapidly diminishing, and abundant alternatives are needed to replace them, particularly in catalysis. For many researchers across the world, iron is an attractive target to this end, owing to its low toxicity and natural abundance.^1−5^ Catalysis mediated by the platinum-group metals is well-understood, and the corresponding mechanisms are often well-elucidated. The same cannot be said for iron.^6−9^ If iron is to compete with and ultimately replace the precious metals in catalysis, a deeper mechanistic understanding is essential. However, the mechanisms underpinning iron-mediated catalysis can be more complex and thus more challenging to elucidate than for late 4d and 5d metals. This is not least due to the large number of accessible oxidation states and spin states: multistate reactivity is common,^10^ and paramagnetism can be problematic when using standard characterization techniques such as NMR spectroscopy. This report is an accurate reflection of how challenging mechanistic elucidation of an iron-catalyzed process can be.

**Scheme 1:**
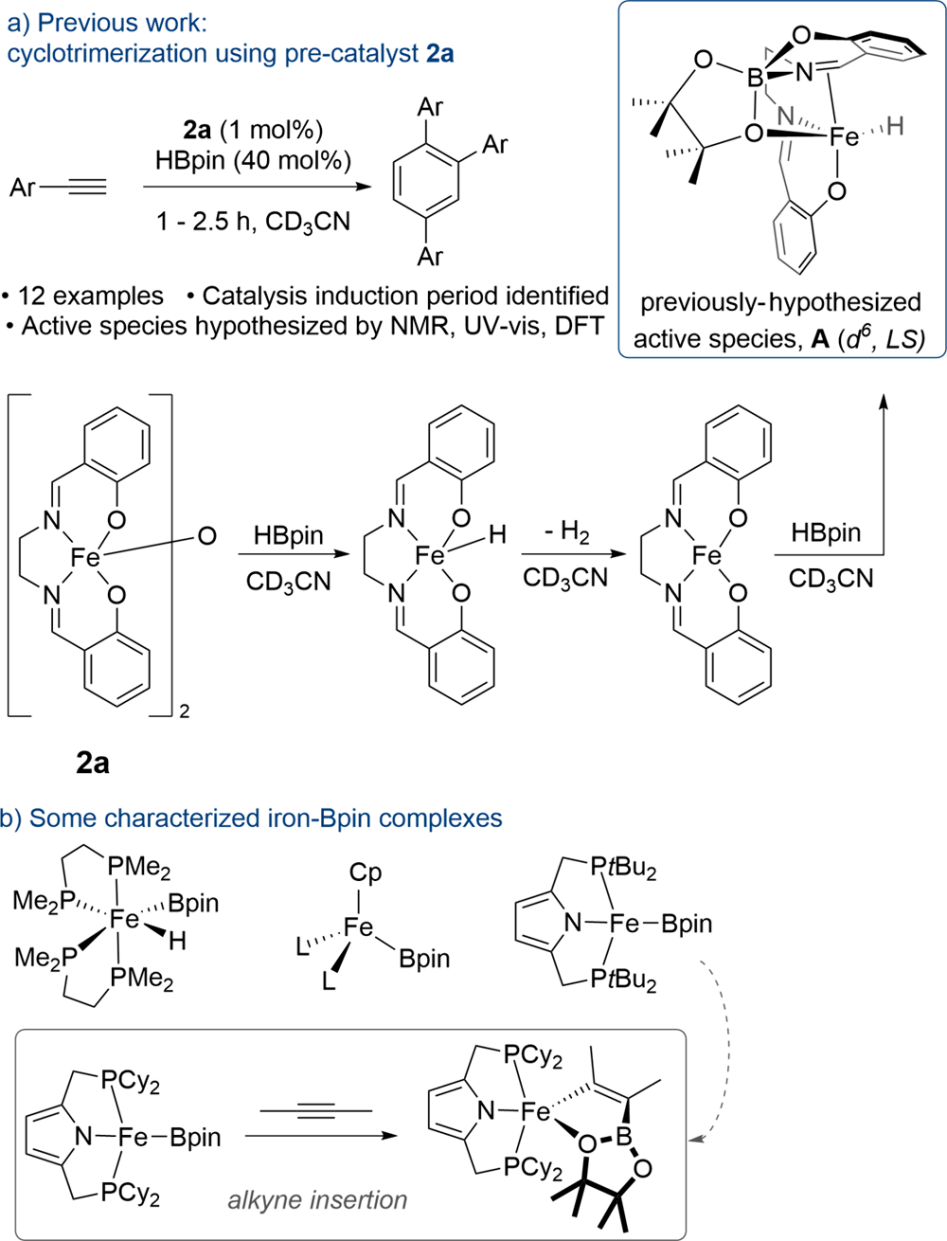
(a) Previous Work and (b) Literature Examples of Fe-Boryl Species

In recent years, the scientific community has produced some remarkable progress within iron-mediated catalysis and the associated mechanistic pathways.^11−17^ Inspired by this, and building on our recent report on an iron-mediated regioselective alkyne trimerization,^18^ we set out to elucidate the pathway of this transformation in more detail. Our original work in this area was postulated to proceed via a precatalyst activation that involves (i) μ-oxo reduction to form Fe(III)-hydride, (ii) Fe(III)-hydride dimerization leading to facile release of hydrogen gas, and (iii) Fe(II)-salen complex formation and reaction with pinacolborane (HBpin) to form an on-cycle diamagnetic species, **A** (Scheme 1a).^18^

Not only have catalytic reactions involving HBpin been expanded upon beyond alkene hydroboration in the past two years (e.g., reductions,^19−22^ direct borylations,^23,24^ and chain-walking transformations^25^ to name but a few) but also metal boryl species have become increasingly prevalent in the literature, particularly as intermediates in metal-mediated hydroboration reactions.^26−28^ Notably, coordinatively unsaturated Fe-boryl complexes have frequently been implemented as potential intermediates in catalytic processes. More recently such species have been isolated and characterized and their insertion into alkynes unequivocally demonstrated (Scheme 1b).^29−38^ Despite this, the unambiguous assignment of Fe-boryl moieties in catalysis remains a challenging endeavor. Herein we present strong evidence for the implication of a transient Fe-Bpin species in the trimerization of terminal alkynes and propose the use of liquid injection field desorption ionization mass spectrometry (LIFDI-MS) as a powerful tool to characterize such open-shell intermediates.^39−41^

## 2 Results and Discussion

### 2.1 Ligand Design

**Scheme 2:**
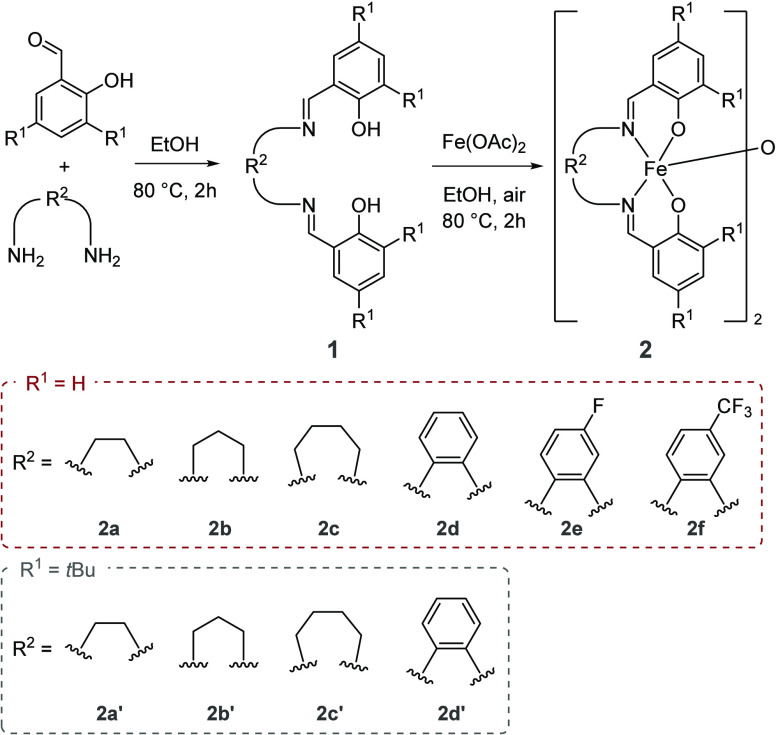
Precatalysts Synthesized in This Work

Motivated by a desire to further understand the mechanism by which **2a** catalyzes the cyclotrimerization of terminal alkynes and gain insight into the proposed active species **A** (Scheme 1a), we sought to (i) reinvestigate the nature of the diamagnetic species computationally assigned as **A** (Scheme 1a) and (ii) expand our library of Fe-salen precursors to give deeper mechanistic understanding (Scheme 2).

A diamagnetic species, assigned as **A** in previous work,^18^ is observed during catalysis. Through extended reaction monitoring studies it was shown to persist after the standard reaction time. We initiated our study into this species by reacting complex **2a** with HBpin (4 equiv) for 3 days. This generates the reassigned diamagnetic compound (herein referred to as **3a**) cleanly. Isolation and implementation in catalysis show that this species is in fact not catalytically active; this is unsurprising given its persistence in catalysis. Although the exact structure of **3a** has not been unequivocally assigned, it is clear the imine moieties in the salen framework are reduced over the course of the stoichiometric reaction to form a salan species (see section 7.5 in the Supporting Information for detailed studies into speciation using a range of boranes across the homologous series **2**, along with likely structure of **3a**).

This in turn prompted us to reevaluate our original DFT-based hypothesis. Species **A** was further inspected computationally. A lower energy isomer of **A** with a shorter Fe–O bond of 2.1 Å vs 2.5 Å and an altered η^2^-interaction between the salen C–N double bond with iron in the original structure was found (see section 12.3 in the Supporting Information for an overlay of the structures). The Gibbs energy stabilization of **A-1**(42) over **A** as a diamagnetic species is predicted to be 2.9 kcal/mol using PBE0-D3/def2-TZVP and 8.6 kcal/mol using a wave function method, namely DLPNO-CCSD(T)/CBS(T,Q).

In the original identification of **A**, we had evaluated different hybrid density functionals in single-point calculations to confirm that it is a low-spin species. We now wanted to reference against a higher level of theory, DLPNO–CCSD(T) (see Computational Methods in the Supporting Information). This showed that **A** is in fact a high-spin species, with the singlet ^**1**^**A-1** and triplet ^**3**^**A-1** configurations destabilized by Δ*G* = 21.2 and 20.9 kcal/mol, respectively, using DLPNO-CCSD(T)/CBS(T,Q). A more detailed comparison is provided in section 12.3 in the Supporting Information.

**Scheme 3:**
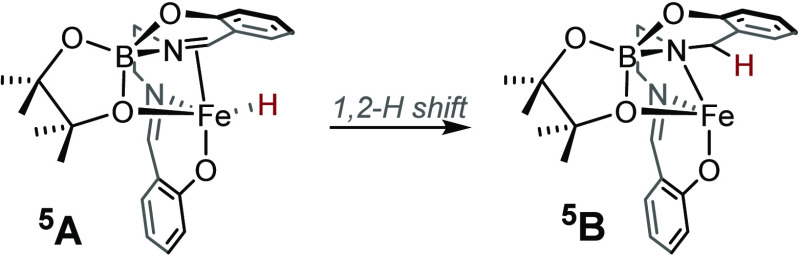
Transformation of Species ^**5**^**A-1** into ^**5**^**B** via a 1,2-Hydrogen Shift Reaction

Upon re-evaluating the geometry of ^**5**^**A-1** as a high-spin species, we found that it can transform via a 1,2-hydrogen shift to form ^**5**^**B** (Scheme 3). The reaction is exergonic with a Gibbs energy of −33.1 kcal/mol using DLPNO-CCSD(T)/CBS(T,Q). This computational observation made us question whether salen ligand reduction is taking place in the presence of HBpin, which might imply that **A** and **A-1** may be high-spin species formed transiently during the induction period. Whether the catalytically competent species has a partial or fully reduced salen backbone needed to be evaluated experimentally.

We hypothesized that through ligand modification (varying steric, flexibility, and electronic properties) we might gain further insight into the active species and mechanism of cyclotrimerization. We synthesized a range of modified salen ligands (see Scheme 2). The corresponding iron complexes (**2a**–**2d′**) were obtained by reaction of the proligand with Fe(OAc)_2_ in a 1:1 mixture in ethanol. Complexes **2a**–**2f** were characterized by ^1^H NMR, LIFDI-MS, and IR spectroscopy (see the Supporting Information for full details).^43^

Computational studies on the monometallic Fe^II^ salen congeners of **2a**–**2d** and **2a′**–**2d**′ allow us to quantify the structural flexibility of the iron coordination sphere. For **2a-mono**, **2a′-mono**, **2d-mono**, and **2d′-mono**, a distorted-square-planar iron environment is found (τ_4_′ = 0.24 (0.23) for **2a-mono** (**2a′-mono**) and τ_4_′ = 0.18 (0.18) for **2d-mono** (**2d′-mono**), where τ_4_′ = 0 for square-planar and τ_4_′ = 1 for tetrahedral geometries).^44^ In contrast, with the propyl and butyl backbones the coordination spheres are closer to a tetrahedral environment (τ_4_′ = 0.69 (0.67) for **2b-mono** (**2b′-mono**) and τ_4_′ = 0.65 (0.64) for **2c-mono** (**2c′-mono**)). In all cases, the iron ion is found in its high-spin state, with the intermediate- and low-spin states less stabilized by at least 7 and 41 kcal/mol using PBE0-D3/def2-TZVP single-point calculations (see section 12.4 in the Supporting Information). That this level of theory is appropriate was confirmed with DLPNO-CCSD(T)/CBS(T,Q) calculations (see section 12.2 in the Supporting Information).

In the case of **2b** and **2b′** only a minor quantity of crystalline material suitable for single-crystal X-ray diffraction analysis could be obtained, and this indicated the formation of analogous Fe(III) acetate complexes (Fe(**1b**)·OAc and Fe(**1b′**)·OAc) rather than the expected μ-oxo Fe(III) dimer.^45^ We cannot unambiguously speak to the bulk purity of **2b** and **2b′**: Fe(**1b**)·OAc and **2b** are indistinguishable by elemental analysis, while ESI-MS invariably corresponds to the [Fe(salen)]^+^ molecular ion in both cases. However, only the higher-order complex [Fe_2_(**1b**)_3_] is identified in the LIFDI-MS and by IR spectroscopy, indicating that Fe(**1b**)·OAc is likely to be a minor species. In contrast, for the bulkier ligand **1b′**, complex **2b′** was not detected by LIFDI-MS; rather, Fe(**1b′**)·OAc is detected as the major species (*vide infra*). Complex Fe(**1b′**)·OAc has been included in further studies because we envisage the mode of action to be analogous to that of other iron(III)-μ-oxo complexes in the series (see the Supporting Information for the anticipated method of catalyst activation). Despite some previously conflicting reports, the μ-oxo Fe(III) dimer was readily identified as the major species by LIFDI-MS for all other complexes **2a**–**2d′**, and no other Fe(III)·OAc species could be identified.^46,47^ These data highlight a key advantage of LIFDI-MS over ESI-MS, which has been noted previously.^48^

**Figure 1:**
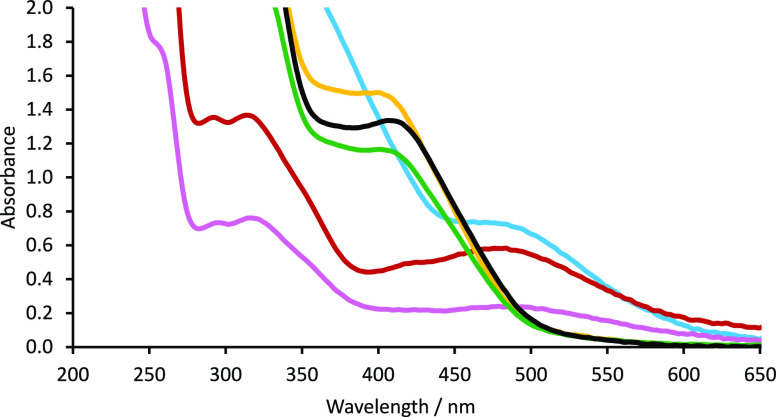
UV–vis spectra of selected precatalysts of the form **2**: (blue line) **2a**; (red line) **2b**; (pink line) **2c**; (yellow line) **2d**; (green line) **2e**; (black line) **2f**.

We initially measured the UV–vis spectrum of precatalysts **2** in order to compare any significant differences in Lewis acidity about the Fe(III) center (Figure 1). In particular, the LMCT band (∼400–500 nm) from the in-plane p_π_ orbital of the phenolate ring to the half-filled d_π*_ orbital of Fe(III) has been used to give an indication of Lewis acidity at the iron center.^49−51^ In our case, we observed only small changes in Lewis acidity upon incorporation of an additional methylene unit into the backbone (compare. **2a** → **2c** and **2a′** → **2c′**). As expected, furnishing the ligand with electron-donating *tert*-butyl substituents greatly increases the Lewis acidity at Fe(III) and substituting the ethylene backbone for a phenyl moiety results in a significantly less Lewis acidic metal center (see section 4 in the Supporting Information for full details). Surprisingly, functionalization of the phenyl backbone (i.e., **2e** and **2f**) results in very subtle electronic changes at the Fe(III) center.

In addition to expanding our library of Fe precursors we also extended our catalyst activator to include catecholborane (HBcat) and 9-borabicyclo[3.3.1]nonane (9-BBN) as well as HBpin to further understand the role of the coreductant.

### 2.2 Reaction Profile Studies

Pleasingly, early benchmarking studies showed that all our Fe precursors **2a**–**2d′** are catalytically competent in the cyclotrimerization of phenylacetylene. As we previously reported, the catalysis is associated with an induction period (20–120 min) in which the active catalyst is generated (*vide infra*). Notably, replacing HBpin with HBcat or 9-BBN completely shuts down catalytic activity and we only see inactive species of the form **3**. This is in line with our previous study and ultimately leads us to the same inference—HBpin is intimately involved in generating the active catalyst.

**Figure 2:**
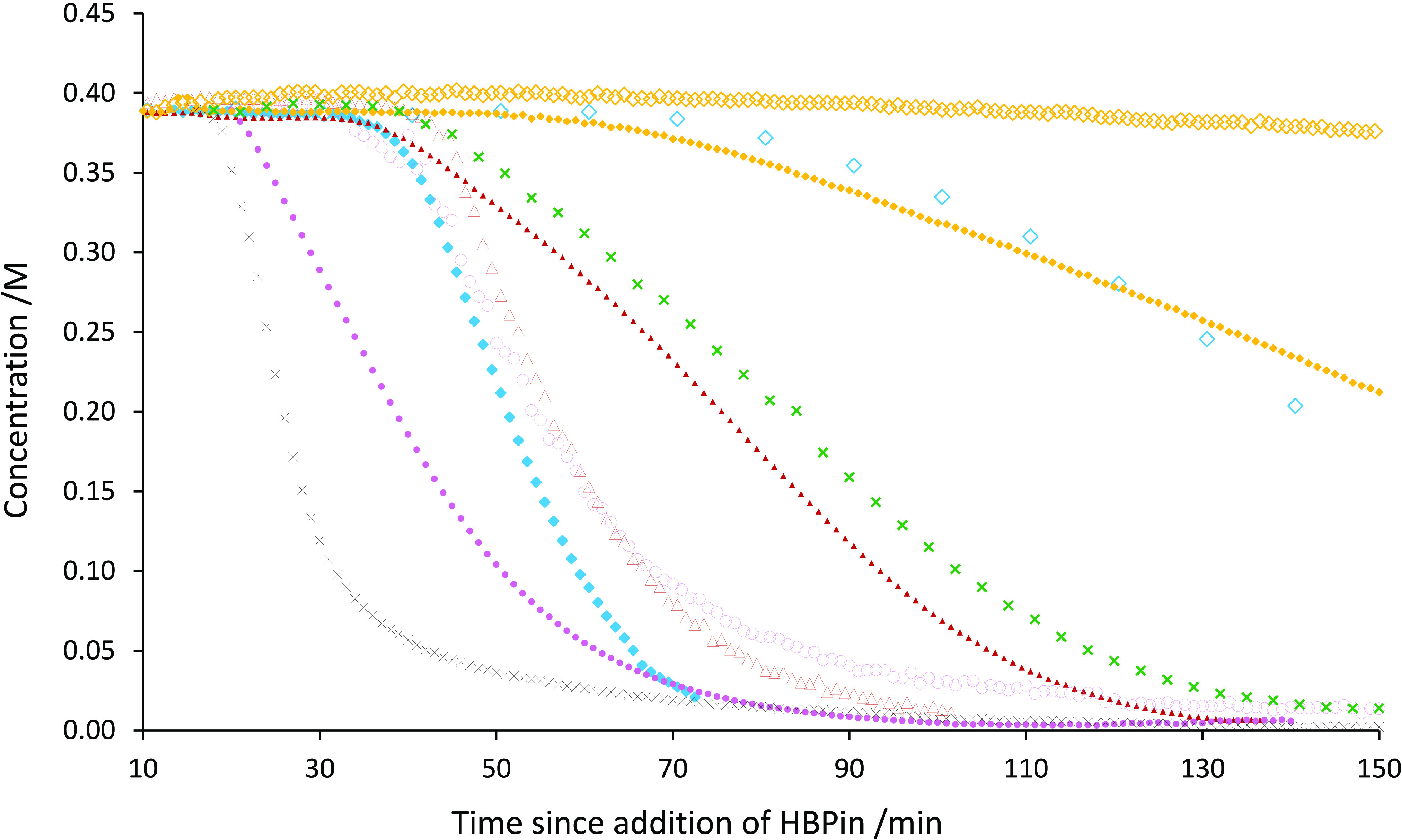
Reaction profiles showing uptake of phenylacetylene using complexes **2a** (blue ◆), **2b** (red ▲), **2c** (pink ●), **2d** (yellow ◆), **2a′** (blue ◇), **2b′** (red △), **2c′** (pink ○), **2d′** (yellow ◇), **2e** (green ×), and **2f** (black ×).

MeCN is the most effective cyclotrimerization solvent, although THF gives a comparable catalytic turnover. The kinetic profile of phenylacetylene cyclotrimerization by each precatalyst in CD_3_CN is summarized in Figure 2 and clearly indicates a significant change in both the induction period and the rate of catalysis.

Generally, the length of the induction period follows a rational trend based on the steric profile of the ligands. In all cases, **2** proceeds with a shorter induction period when compared to its *tert*-butyl-functionalized analogue **2′** (Table 1). This is readily rationalized, as the activation of the precatalysts (and approach of HBpin) is perturbed by the additional *t*Bu appendages. Expanding the ligand backbone to add “structural flexibility” (in the order **2c′** > **2b′** > **2a′** > **2d′**) generally results in a reduction in induction period (in the order **2c′** > **2b′** > **2a′** > **2d′**).

**Table 1:** Summary of Rate Data for the Range of Precatalysts^a^

complex	induction time (min)	rate (10^–2^ mol L^–1^ s^–1^)	time to completion (min)^b^
**2a**	35	1.11	91
**2b**	38	0.53	129
**2c**	22	0.96	88
**2d**	60	0.22	264
**2e**	22	0.50	147
**2f**	40	1.70	97
**2a′**	70	0.30	241
**2b′**	46	1.07	68
**2c′**	35	1.39	108
**2d′**	100–120	0.03	≫300^c^

a Conditions: precatalysts **2** (1 mol %), phenylacetylene (0.25 mmol), HBpin (0.10 mmol), RT, CD_3_CN (600 μL). Spectroscopic consumption of phenylacetylene measured by ^1^H NMR spectroscopy against 1,3,5-trimethoxybenzene.

b Time point where [SM] < 2.5%.

c [SM] plateau.

Based on our initial hypothesis we expected to see a clear trend in induction period (or even catalytic competency) reflected in the flexibility of the ligand backbone. However, the rates of reaction following catalytic induction show no obvious trends. In the case of the *tert*-butylated series (**2**′) the rate increases in the order **2c′** > **2b′** > **2a′** > **2d′**, which corresponds well with the addition of flexibility in the ligand backbone. However, this trend does not hold up in the absence of *tert*-butyl groups, where we see a rate of the order **2a** > **2c** > **2b** > **2d**. Clearly the data cannot be explained simply through ligand backbone flexibility.

It is worth noting that the final ratio of 1,2,4-triphenylbenzene to 1,3,5-triphenylbenzene products, tested across this diverse range of precatalysts, is almost identical (98% 1,2,4- to 2% 1,3,5-product). This indicates that ligand modification has little effect on the regioselectivity (*vide infra*).

Precatalysts **2e** and **2f** have been included for completeness, but **2e** suffered from very poor solubility in the reaction solvent. As such the induction period and rate of reaction of **2e** cannot be accurately compared to the rest of the series. The rapid rate demonstrated by **2f** is noteworthy and counterintuitive, especially in comparison with **2d** and **2e**. We have undertaken homogeneity tests^52^ using both PMe_3_ and Hg with **2a** and **2f**, and the results of these support a homogeneous reaction (see section 5.3 in the Supporting Information).

### 2.3 Stoichiometric Studies

In order to revisit the mechanism of alkyne cyclotrimerization and gain further insight into catalyst activation, an extensive range of stoichiometric reactions and LIFDI mass spectrometry studies were undertaken. The necessity for coordinating solvents in catalysis was elucidated by reacting complexes **2a**–**2d′** with 10 equiv of HBpin in both CD_3_CN and C_6_D_6_. These reactions were monitored periodically by ^1^H, ^11^B, and, where appropriate, ^19^F NMR spectroscopy over 48 h (see the Supporting Information). In all cases the spectra were associated with the rapid generation of O(Bpin)_2_ (BOB, ^11^B NMR = 21 ppm), presumably forming [(Fe^III^salen)H] as per our originally hypothesized catalyst activation process (*vide supra*). As reported previously, the reaction of precatalyst **2a** with HBpin generates a diamagnetic complex as the major species in CD_3_CN (**3a**) after 1 h at RT, i.e. after the induction period for **2a**. However, we can now confirm that in most cases the reaction of precatalysts **2** and HBpin also result in broad paramagnetic signals in the ^1^H NMR spectra that elude meaningful characterization (see section 7 in the Supporting Information). This likely indicates that on-cycle catalysis is dominated by paramagnetic intermediates (in keeping with our DFT studies). The only other exceptions are those of **2e** and **2f**; upon treatment with HBpin in C_6_D_6_ well-defined diamagnetic species (**3e** and **3f**) form with BOB as the only isolable byproduct (see section 7 in the Supporting Information).

**Figure 3:**
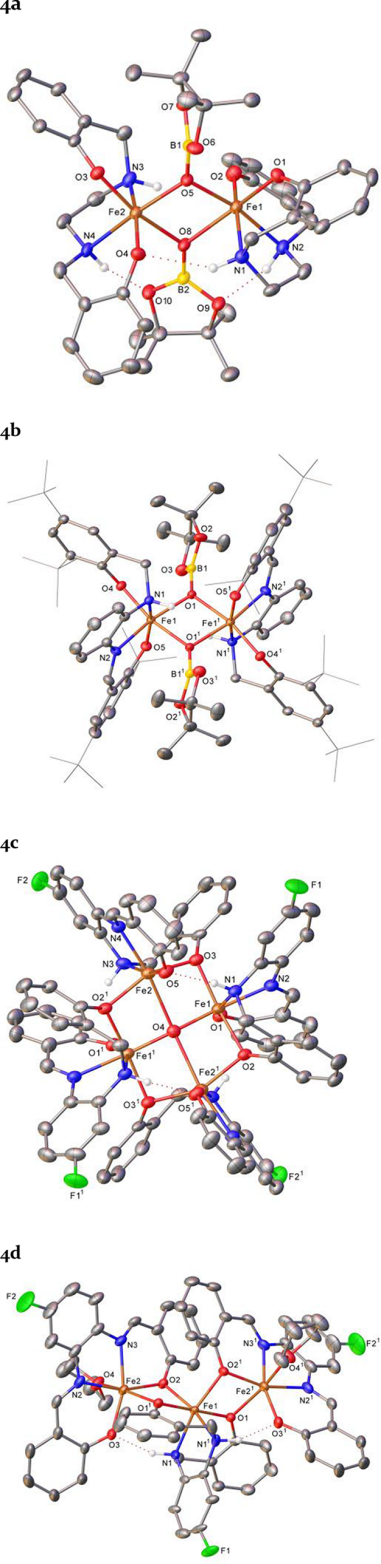
Molecular structure representation (30% ellipsoids) of compounds **4a**–**4d**. **4a** (CCDC 2260432): hydrogen atoms, except for those which are nitrogen-bound, have been omitted for clarity. **4b** (CCDC 2260433): hydrogen atoms, except for those which are nitrogen-bound, have been omitted for clarity. The minor disordered component has also been omitted and *tert*-butyl groups are represented in wireframe mode, also for visual ease. Symmetry operations: (1) 1 – *x*, 1 – *y*, 1 – *z*. **4c** (CCDC 2260599): solvent, hydrogen atoms (except for those which are nitrogen-bound), and the minor disordered components have been omitted for clarity. Symmetry operations: (1) 1 – *x*, *y*, 1/2 – *z*. **4d** (CCDC 2260434): the minor disordered components and hydrogen atoms (except for H1) have been omitted for clarity. Symmetry operations: (1) 1 – *x*, *y*, 1/2 – *z*.

The onward catalyst activation (via a hypothesized backbone reduction) was confirmed by the formation and crystallographic characterization of complex **4a** by the intentional reaction of **3a** with water under inert conditions. The ligand reduction is stepwise, as evidenced by the serendipitous crystallizations of various intermediates, across a range of pro-ligands, along this pathway (**4a**–**d**, Figure 3). We believe the oxygen in complexes **4a**–**c** are the result of crystallographic trapping of intermediates (at various stages of reduction).^53^

Complex **4a**, which contains ligand **1a**, shows complete reduction of the iminyl moieties of the salen ligand to yield a dimeric iron species, in which each of the Fe(III) centers bears a salan ligand and the two iron centers are bridged by OBpin units. Complex **4b** shows a similar structure in which two Fe(III) centers are bridged by two OBpin units but the parent ligand (**1d**) has only undergone partial reduction. Thus, a salalen ligand system is present whereby the proligand contains one imine (at N2/N2^1^) and one amine fragment (at N1/N1^1^). **4c** is a mixed Fe(II)/Fe(III) complex consisting of four iron centers ligated by salalen ligands (from **1e**) with a central oxo ion. Finally, **4d** is a mixed salen-salan-salen trimeric species derived from proligand **1e**.

We emphasize our observation that ligated salen can be reduced *in situ* by a simple reductant like HBpin has important implications for the catalysis presented here and beyond because (i) formation of an active Fe-salan complex could indicate that catalyst activation and active cyclotrimerization catalysis involves some element of ligand reduction and (ii) salen ligands are commonly regarded as being synthetically robust and somewhat inert; our study herein indicates otherwise.^54−57^

We hypothesized that this *in situ* ligand reduction may be responsible for the observed induction period associated with our cyclotrimerization catalysis. However, **3a** is an end point of catalysis and cannot mediate cyclotrimerization. Therefore, we targeted an appropriate [Fe^III^(salan)] precursor, [Fe(^H^salan)(OH)]_2_ (**5**, Table 2), which has previously been reported.^58^

**Table 2:**
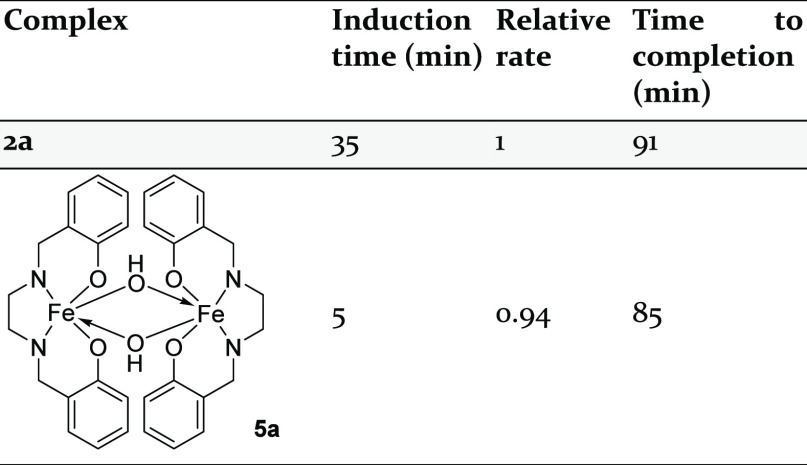
Comparison of Induction Period and Rate of Reaction (**2a** versus **5a**)^a^

a Conditions: complex **2a** or **5a** (1 mol %), *tert*-butylphenylacetylene (0.25 mmol), HBpin (0.10 mmol), RT, CD_3_CN (500 μL). Spectroscopic consumption of *tert*-butylphenylacetylene measured by ^1^H NMR spectroscopy against 1,3,5-trimethoxybenzene.

**Figure 4:**
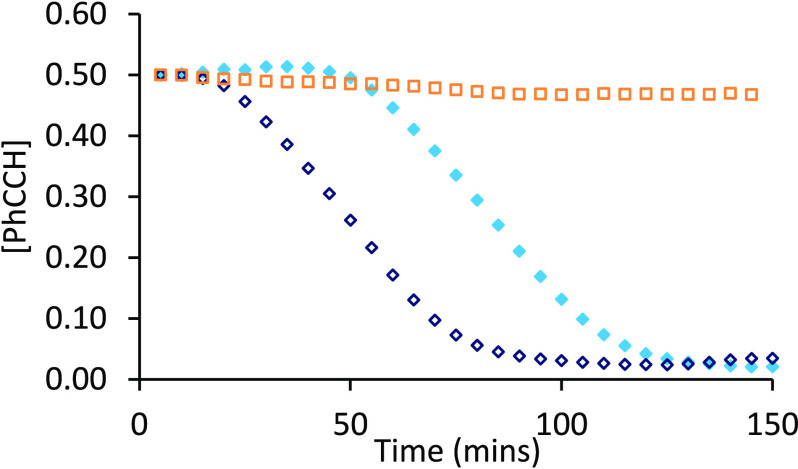
Reaction profile of cyclotrimerization of *tert*-butylphenylacetylene mediated by complexes **2a** (blue ◆), **5** (black ◇), and **3a** (yellow □).

Complex **5** shows catalytic competence in the cyclotrimerization of 4-*tert*-butylphenylacetylene. Catalysis with **5** proceeds with a significantly reduced induction period compared to **2a** (5 min with **5** compared to 35 min with **2a**, Figure 4), indicating that reduction of salen to salan is partially responsible for the induction periods observed in cyclotrimerization with precatalysts **2**. The rate of catalysis with **5a** is almost identical to that using **2a**, adding more weight to the argument that a salan-type ligand is key during active catalysis. Mirroring precatalysts **2**, the combination of **5** and HBpin is vitally important in mediating cyclotrimerization; complex **5** is not catalytically competent alone (nor is catalysis mediated by an *in situ* generated [Fe^II^(salan)] species; see the Supporting Information).

### 2.4 LIFDI-MS Studies

Identification of the exact nature of the active species still proved elusive and any signals detected by NMR spectroscopy were very broad, spanning tens of ppm range, possibly indicating a high-spin Fe(III) system, reinforcing our DFT calculations. ESI-MS is not helpful and only shows the [Fe(salen)]^+^ or [Fe(salan)]^+^ molecular ion. We therefore turned our attention to mass spectrometry to identify catalyst speciation and possible deactivation pathways.^59−63^ For this purpose we employed LIFDI-MS, which is the softest ionization for mass spectrometry and has been used to detect highly sensitive and otherwise elusive organometallic species.^64,65^

**Figure 5:**
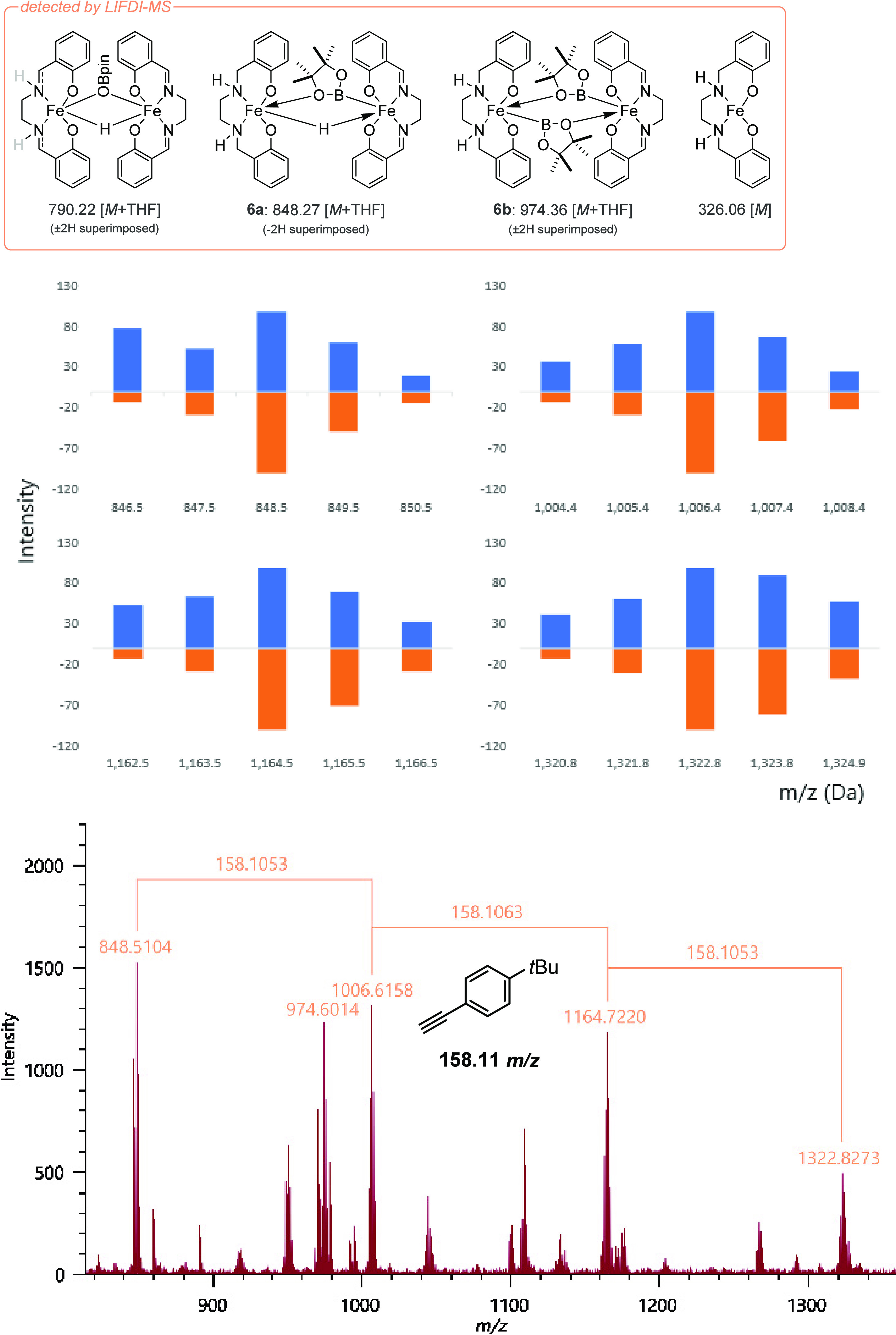
Selection of key species identified by LIFDI-MS and *in situ* LIFDI-MS of a catalytic run (top) along with predicted (blue) versus experimental (orange) isotope splitting patterns for species observed with *m*/*z* 848, 1006, 1164, 1322 Da (middle) and a full spectrum indicating trimerization observed *in situ* (bottom).

Our LIFDI-MS analysis targeted the detection of the intermediates associated with the reaction of **2a** with HBpin. The reaction was initially followed stoichiometrically with periodic LIFDI-MS measurements, facilitating detection of **6a** as well as the partial reduction of the salen backbone. After 45 min a signal at 848 *m*/*z* was observed which corresponds to [Fe(salen)H][Fe(salan)Bpin]·THF (**6a**·THF)^66^ and is congruent with the timeframe of catalyst induction (Figure 5). We assign this as the mixed salen/salan species rather than a [Fe(salalen)H][Fe(salalen)Bpin]·THF species (i) because of the superimposing rate data obtained using **5a** and **2a** and (ii) the fact that the Fe-hydride fragment can easily re-enter the catalyst activation process, eventually forming Fe(II)salen (see the Supporting Information), which can be further reduced to access the catalytic cycle. As expected, based on lack of cyclotrimerization catalysis, no analogous LIFDI-MS complexes are observed for the reaction of **2a** with HBcat or 9-BBN.

Following this, we studied the catalytic cyclotrimerization of 4-*tert*-butylphenylacetylene with a 5 mol % catalyst loading of **2a** using periodic LIFDI-MS measurements. 4-*tert*-Butylphenylacetylene undergoes cyclotrimerization in 20 h, compared to only 1 h with phenylacetylene, providing a more protracted time frame for reaction monitoring.

After 1 h the signal at *m*/*z* 848 was identified alongside signals corresponding to the insertion of 4-*tert*-butylphenylacetylene (*m*/*z* +152) (see Scheme 3). Thus we can infer that an iron(III) boryl is at least one of the active species in catalysis; the insertion of terminal alkynes into Fe-boryl species has recently been established.^34,37,38^ Furthermore, the peak at *m*/*z* 974 can be satisfactorily modeled as [Fe(salen)Bpin][Fe(salan)Bpin]·THF (**6b**·THF). We postulate that dimeric species (**6a**,**b**) are observed during catalysis due to the high concentrations required when undertaking LIFDI-MS studies; due to the relatively poor sensitivity of LIFDI-MS both the catalytic and stoichiometric reactions were undertaken at more than 4 times the concentration than in the NMR spectroscopy and kinetic experiments (*vide supra*). This is likely the cause of the discrepancy between the measured order in precatalyst (1/2) when compared to the observation of dimeric species detected by LIFDI-MS. A more detailed summary of iron speciation identified by LIFDI-MS is provided in section 8 in the Supporting Information.

## 3 Summary and Catalytic Cycle

In this comprehensive synthetic and computational study, we can summarize the findings as follows: (i) paramagnetism dominates catalysis and an iron(III) species is likely responsible for on-cycle catalysis; (ii) DFT studies show that even within our range of salen complexes there is a huge range of conformational flexibility based on the iminyl linker, where the geometry around the iron ranges from distorted square planar to tetrahedral; (iii) reduction of salen to salan further increases the number of accessible conformers, impeding crystallization of likely intermediates or catalyst resting states; (iv) crystallization of catalyst decomposition products invariably shows partial or complete reduction of the ligand from salen to salalen or salan; (v) LIFDI-MS studies give strong evidence, during catalysis, for an iron-boryl species that also contains a salan ligand system; (vi) LIFDI-MS studies show sequential insertion of *tert*-butylphenylacetylene into a mixed iron(III) dimer that likely contains both salen and salan ligands with iron-hydride and iron-boryl units.

We have attempted a huge range of reactions in our attempts to isolate an iron-boryl complex. That we cannot isolate such a species from stoichiometric reactions from salen, salalen, or salan complexes, even at low temperature, is not surprising given that much of the catalysis, irrespective of ligand design (**2a**–**f**, **2a′**–**d′**), is complete within 2–3 h at room temperature. The ease of catalytic cyclotrimerization is further indicated by the fact that we do not see alkene or alkenyl-borane side products being formed.

**Scheme 4:**
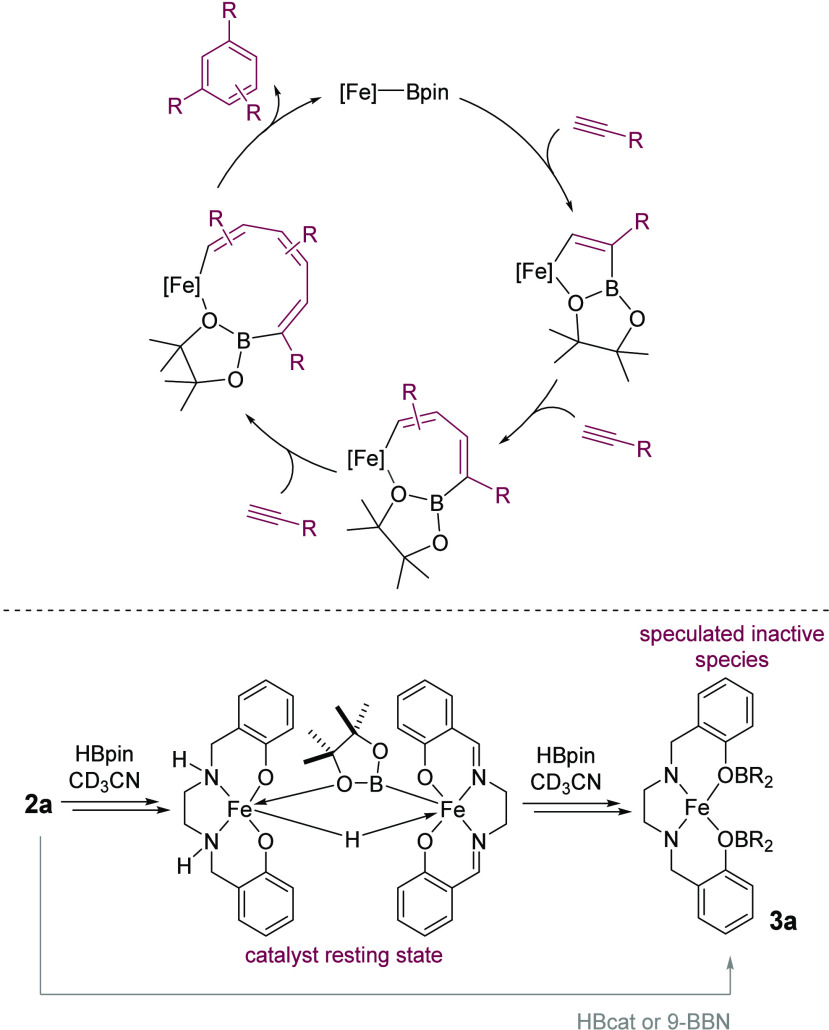
Proposed Catalytic Cycle and Overview of the Reactivity of Precatalyst **2a**

We therefore postulate a catalytic cycle (Scheme 4) that involves formation of an on-cycle Fe^III^(salan)Bpin species, insertion of alkyne into the Fe–B bond, which is likely stabilized by the presence of the pinacol oxygen, followed by subsequent insertion of alkyne before cyclization and elimination of the aromatic product. We link the ease of the catalysis to the conformational flexibility of the ligand system and the Bpin ligand: catalysis is not observed with 9-BBN, likely due to the lack of stabilizing oxygen atoms, while the rigidity of HBcat precludes stabilizing coordination. Thus 9-BBN and HBcat lead to the formation of the catalytically inactive species **3**, which we tentatively structurally assign as that depicted in Scheme 4.

## 4 Conclusion

We have prepared a homologous series of [(salen)Fe]_2_(μ-O) precursors in which the salen ligand has been modified. The catalytic cyclotrimerization of terminal alkynes mediated by this library of complexes prompted us to reinvestigate the associated mechanistic pathway with additional scrutiny. We have demonstrated that the activation of such precatalysts with HBpin is more complicated than one might expect. Reduction of the iminyl backbone of the assumed-unreactive salen ligand under these mild reductive conditions is of particular note.

Furthermore, we have used LIFDI-MS and interrogated decomposition pathways using NMR spectroscopy and X-ray crystallography to provide strong evidence for a transient iron(III)-boryl active species. Complete untangling of the complex kinetic data presented herein is beyond the scope of this study. We link the lack of crystallographic evidence to the extreme number of conformers available to such a flexible salan system, coupled with the facile nature of the cyclotrimerization catalysis. The extreme flexibility across the series of salan ligands tested may also result in a flexible reaction pocket that is unable to force reactivity away from the formation of the more energetically favored 1,2,4-triphenylbenzene product.

It is clear from this study that the role of the ligand and its potential reactivity, or activation, in order to effect catalysis is an important consideration, the implications of which may be important in the wider field of homogeneous catalysis.

## Supporting Information Available

Analytical data and NMR spectra for all products and all data relevant to the computational studies reported here, including methodology, benchmarking studies, energetics, and coordinates of characterized stationary points (PDF)
Coordinates of all the species discussed in this publication (CIF)

## Supplementary Material

cs3c02898_si_001.pdf

cs3c02898_si_002.cif
