# Supplementary material for: The Complex Reactivity of [(salen)Fe]2(μ-O) with HBpin and Its Implications in Catalysis
Source: ACS Catal. 2023 Aug 23;13(17):11841–50. doi: 10.1021/acscatal.3c02898 (PMC10476159; doi:10.1021/acscatal.3c02898)
Supplement: Supplementary file 1 — cs3c02898_si_001.pdf [file cs3c02898_si_001.pdf]

Electronic Supporting Information:

The complex reactivity of [(salen)Fe]$_2$(μ-O) with HBpin and its implications in catalysis

Thomas M. Hood,[†a] Samantha Lau,[†a] Martin Diefenbach, [†b] Leah Firmstone,[a] Mary Mahon,*[a]

Vera Krewald *[b] and Ruth Webster*[a]

[a]Department of Chemistry, University of Bath, Claverton Down, Bath, United Kingdom, BA2 7AY

[b]Department of Chemistry, TU Darmstadt, Peter-Grünberg-Str. 4, 64287 Darmstadt, Germany

* m.f.mahon@bath.ac.uk
* krewald@chemie.tu-darmstadt.de
* r.l.webster@bath.ac.uk

Table of Contents

1.	General Considerations	S3
2.	Synthesis of Ligands	S4
2.1.	General Procedure	S4
3.	Synthesis of precatalysts	S7
3.1.	General Procedure	S7
4.	UV-vis spectra	S16
4.1.	R^1 = H	S16
4.2.	R^1 = tBu	S16
4.3.	Comparison of all ethyl backbone [Fe] complexes	S17
5.	Kinetic experiments	S17
5.1.	General procedure for the trimerization of phenylacetylene	S17
5.2.	Trimerization of 4-tert-butylphenylacetylene	S23
5.3.	Catalysts inhibition experiments	S22
6.	Trimerization with **2a'**	S23
7.	Stoichiometric reactions	S23
7.1.	General procedure for the treatment of **2** with HBpin	S23
7.2.	Varying borane stoichiometry	S31
7.3.	Alternative boranes	S31
7.4.	Reaction with DBpin	S41
7.5.	Identification of **3**	S41
8.	Detection and characterisation of catalytically relevant species	S43
8.1.	Stoichiometric LIFDI-MS reaction monitoring	S43
8.2.	Catalytic monitoring with LIFDI-MS	S49
9.	Attempts to generate **3a** and [Fe(salan)Bpin] (**8**)	S53
9.1.	Attempt to generate **3**	S53
9.2.	Attempt to generate **3** or **8** from Fe(II)	S53
9.3.	Attempts to generate **8** from Fe(III)	S53
10.	Potential modes of activation of Precatalyst	S56
11.	Single Crystal X-ray Diffraction Analysis	S57
12.	Quantum-Chemical Studies	S59
12.1.	Computational Methods	S59
12.2.	Spin-State Energetics: Fe^{II}(salen)	S60
12.3.	Isomers of Species **A**	S62
12.4.	Coordination Sphere **2a**–**2d'**	S65
12.5.	Regioselectivity for 1,2,4- and 1,3,5-triphenylbenzene	S67
13.	References	S70
A.	Appendix: Cartesian coordinates of optimized geometries (Å)	S72

1. General Considerations

Ligand and precatalyst synthesis were performed in air. All other manipulations were carried out using standard Schlenk-line and glovebox techniques under an inert atmosphere of argon (Ar). An MBraun MB200B glovebox was employed operating at <0.1 ppm O_2 and <0.1 ppm H_2O. Acetonitrile was dried over two batches of 3 Å molecular sieves, sparged with Ar and stored under an inert atmosphere. Benzene, toluene, and tetrahydrofuran (THF) were distilled from Na/benzophenone and stored over activated 3 Å molecular sieves. d_3-Acetonitirile was dried over two batches of 3 Å molecular sieves, sparged with Ar and stored under an inert atmosphere. d_6-Benzene was degassed and dried over sodium and freeze-pump-thaw degassed. Glassware was dried for 12 hours at 120°C prior to use. NMR spectra were obtained on Bruker or Agilent 400 or 500 MHz instruments at 298 K, all peaks are referenced against residual solvent peak or internal standard peak where stated with values quoted in ppm and coupling constants in Hz. Data were processed in MestReNova. Crystal structures were obtained from either a Rigaku Oxford Diffraction Xcalibur (MoKα (λ = 0.71073)) or Supernova (CuKα (λ = 1.54184)) diffractometer. Melting point analyses were conducted on a Stuart SMP10 melting point apparatus. Infrared spectra were recorded at ambient temperature on a Perkin Elmer Spectrum 100 FT-IR spectrometer using a diamond ATR unit. UV-Vis spectra were recorded on a Mettler Toledo UV5 spectrometer. LIFDI mass spectrometry were carried out using a Waters GCT Premier MS Agilent 7890A GC performed at the University of York by Mr. Karl Heaton.

Unless otherwise stated, all chemicals were purchased from commercial sources and used as supplied. Pinacolborane, phenylacetylene and 4-*tert*-butylphenylacetylene were purchased from commercial sources and distilled and analysed before use. *N*,*N*'-bis(2-hydroxybenzyl)ethylenediamine (^{H}salan),[1] 3,5-di-*tert*-butyl-2-hydroxybenzaldehyde,[2] $Fe(HMDS)_2$,[3] benzyl potassium (KBn),[4] $O(Bpin)_2$[5] and [Mg(^{Dipp}Nacnac)(Bpin)(DMAP)][6] were prepared according to a literature procedures.

2. Synthesis of Ligands

2.1. General Procedure

A solution of aldehyde in ethanol was added to a solution of diamine in ethanol in air. The reaction was stirred at reflux for 2 hours and then allowed to cool to room temperature. The suspension was further cooled to -30 °C and filtered, the remaining yellow solid was washed with cold ethanol (-30 °C, 3 x ~ 10 – 20 mL) and dried under vacuum.

2.1.1. **Preparation of 1a**

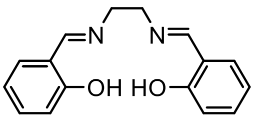

Following the general procedure and using salicylaldehyde (2.2 mL, 20.6 mmol) and ethylenediamine (0.34 mL, 5.09 mmol) in EtOH (40 mL). Yield: 1.34 g (98%). **^{1}H NMR** ($CDCl_3$, 500 MHz): δ 13.19 (br s, 2H OH), 8.36 (s, 2H, N=CH), 7.31-7.27 (m, 2H, Ar), 7.24-7.22 (m, 2H, Ar), 6.95-6.93 (m, 2H, Ar), 6.87-6.84 (m, 2H, Ar), 3.95 (s, 4H, CH_2). **FT-IR** (cm^{-1}): 2902.27 (C-H aromatic), 1609.99 (C=N), 1281.44 (C-O), 740.14 (=CH). **ESI-MS** (positive ion, 4 kV): 269.1285, [*M+H*] (calc. 269.1290) *m/z*. **M.p.** 125 – 127 °C. Data are consistent with the literature.[7]

2.1.2. **Preparation of 1b**

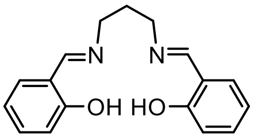

Following the general procedure and using salicylaldehyde (1.8 mL, 16.9 mmol) and 1,3-diaminopropane (0.45 mL, 5.34 mmol) in EtOH (40 mL). Yield: 918 mg (63%). **^{1}H NMR** (400 MHz; $CDCl_3$): *δ* 13.36 (br s, 2H, OH), 8.31 (s, 2H, N=CH), 7.24 (td, 2H, $^{3}J_{HH}$ = 7.8, $^{1}J_{HH}$ = 1.8, Ar), 7.18 (dd, 2H, $^{3}J_{HH}$ = 8.2, $^{4}J_{HH}$ = 1.7, Ar), 6.90 (dd, 2H, $^{3}J_{HH}$ = 8.3, $^{4}J_{HH}$ = 1.1, Ar), 6.81 (td, 2H, $^{3}J_{HH}$ = 7.5, $^{4}J_{HH}$ = 1.1, Ar), 3.65 (td, 4H, $^{3}J_{HH}$ = 6.7, $^{4}J_{HH}$ = 1.2, N-CH_2), 2.06 (p, 2H, $^{3}J_{HH}$ = 6.7, CH_2). **FT-IR** (cm^{-1}): 3052.63 (C-H aromatic), 2996.37 (C-H aromatic), 2947.30 (C-H aromatic), 2896.68 (C-H aromatic), 2870.60 (C-H aromatic), 1609.29 (C=N), 1276.38 (C-O), 748.94 (=CH), 662.33. **M.p.** 53 °C. Data are consistent with the literature.[8, 9]

2.1.3. **Preparation of 1c**

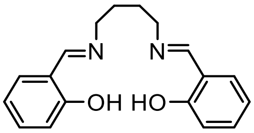

Following the general procedure and using salicylaldehyde (427 µL, 4.09 mmol) and 1,4-diaminobutane (117 µL, 1.17 mmol) in EtOH (10 mL). Yield: 210 mg (61%). **^{1}H NMR** (400 MHz, $CDCl_3$): *δ* 13.47 (br s, 2H, OH), 8.35 (s, 2H, N=CH), 7.34 – 7.28 (m, 4H, Ar), 6.96 (d, $^{3}J_{HH}$ = 7.3, 4H, Ar), 6.89 – 6.83 (m, 4H, Ar), 3.66 (s, 4H, NCH_2), 1.82 (s, 4H, CH_2). **FT-IR** (cm^{-1}): 3051 (O-H), 2947 (C-H), 2864 (C-H), 1676 (C=N), 1495 (C=C), 1379 (C-N), 1283 (C-O). **ESI-MS** (positive ion, 4 kV): 297.1597, $[M+H]^+$ (calc. 297.1603) *m/z*. **M.p.** 89 – 92 °C. Data are consistent with the literature.[9]

2.1.4. **Preparation of 1d**

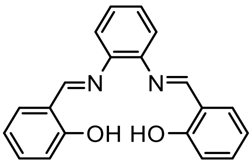

Following the general procedure and using salicylaldehyde (2.4 mL, 22.5 mmol) and *o*-phenylendiamine (1.08 g, 10.0 mmol) in EtOH (45 mL). Yield: 2.52 mg (80%). **^{1}H NMR** ($CDCl_3$, 500 MHz): δ 13.01 (br s, 2H, OH), 8.65 (s, 2H, N=CH), 7.41-7.35 (m, 6H, Ar), 7.27-7.25 (m, 4H, Ar), 7.07 (d, $^{3}J_{HH}$ = 8.24, 2H, Ar), 6.94 (td, $^{3}J_{HH}$ = 7.49, $^{4}J_{HH}$ = 1.11, 2H, Ar). Data are consistent with the literature.[10]

2.1.5. **Preparation of 1e**

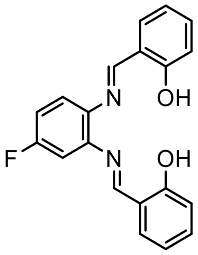

Following the general procedure and using salicylaldehyde (1.70 mL, 16.3 mmol) and 4-fluoro-1,2-phenylenediamine (1.00 g, 7.93 mmol) in EtOH (75 mL). Yield: 1.32 g (49%). **^{1}H NMR** ($CDCl_3$, 400 MHz): δ 12.81 (s, 1H, OH), 12.66 (s, 1H, OH), 8.51 (s, 2H, N=CH), 7.32 – 7.23 (m, 4H, Ar), 7.12 (dd, $^{3}J_{HH}$ = 8.8, $^{3}J_{FH}$ = 5.5, 1H, Ar), 6.98 – 6.92 (m, 3H, Ar), 6.88 (dd, $^{3}J_{HH}$ = 9.2, $^{4}J_{HH}$ = 2.7, 1H, Ar), 6.86 – 6.80 (m, 2H, Ar). **^{19}F NMR** ($CDCl_3$, 376 MHz): δ -113.9 (s). Data are consistent with the literature.[11]

2.1.6. **Preparation of 1f**

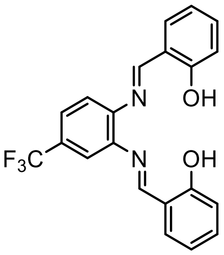

Following the general procedure and using salicylaldehyde (1.20 mL, 11.5 mmol) and 4-(trifluoromethyl)-1,2-phenylenediamine (1.00 g, 5.68 mmol) in EtOH (75 mL). Yield: 1.46 g (67%). **^{1}H NMR** ($CDCl_3$, 400 MHz): δ 12.58 (s, 1H, OH), 12.56 (s, 1H, OH), 8.57 (s, 1H, N=CH), 8.54 (s, 1H, N=CH), 7.50 (dd, $^{3}J_{HH}$ = 8.1, $^{2}J_{HH}$ = 1.4, 1H), 7.38 (d, $^{4}J_{HH}$ = 1.9, 1H), 7.31 (tdd, $^{3}J_{HH}$ = 6.9, 3.9, 1.7, 4H), 7.21 (d, $^{3}J_{HH}$ = 8.2, 1H), 6.95 (dd, $^{3}J_{HH}$ = 8.2, $^{4}J_{HH}$ = 3.4, 2H), 6.85 (t, $^{3}J_{HH}$ = 7.5, 2H). **^{19}F NMR** ($CDCl_3$, 376 MHz): δ -62.19 (s).

$^{13}C\{^1H\}$ **NMR** ($CDCl_3$, 101 MHz) δ 165.4 (d, J = 13, N=CH), 165.1 (d, J = 13, N=CH), 161.6 (d, J = 5, C-O), 145.7 (Ar), 143.0 (Ar), 134.4 – 134.0 (m, Ar), 133.1 – 132.5 (m, Ar), 129.7 (d, $^1J_{FC}$ = 33, CF_3), 124.6 – 124.4 (m, Ar), 120.7 – 120.4 (m, Ar), 119.4 (Ar), 119.9 (Ar) 118.0 – 117.6 (m, Ar), 117.3 – 116.9 (m, Ar).

2.1.7. **Preparation of 1a′**

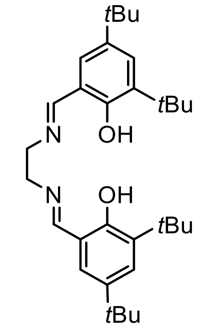

Following the general procedure and using 3,5-di-*tert*-butyl-2-hydroxybenzaldehyde (0.70 g, 3.00 mmol) and ethylenediamine (57.0 µL, 0.85 mmol) in EtOH (20 mL). Yield: 375 mg (89%). ^{1}**H NMR** (400 MHz; $CDCl_3$): δ 13.56 (s, 2H, OH), 8.31 (s, 2H, N=CH), 7.29 (d, 2H, $^4J_{HH}$ = 2.5, Ar), 6.99 (d, 2H, $^4J_{HH}$ = 2.5, Ar), 3.85 (s, 4H, CH_2), 1.36 (s, 18H, *t*Bu), 1.21 (s, 18H, *t*Bu). **FT-IR** (cm^{-1}): 2956.08 (C-H aromatic), 2867.71 (C-H aromatic), 1626.22 (C=N), 973.50 (=CH), 931.70, 909.83, 879.78, 829.61, 798.43, 773.44, 751.32, 729.52, 710.34. **M.p.** 160 °C. Data are consistent with the literature.[12]

2.1.8. **Preparation of 1b′**

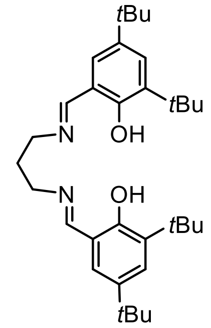

Following the general procedure and using 3,5-di-*tert*-butyl-2-hydroxybenzaldehyde (0.63 g, 2.69 mmol) and 1,3-diaminopropane (64.0 µL, 0.76 mmol) in EtOH (20 mL). Yield: 356 mg (92%). ^{1}**H NMR** (500 MHz; $CDCl_3$, 298 K): δ 13.83 (s, 2H, OH), 8.41 (d, 2H, $^4J_{HH}$ = 1.2, N=CH), 7.40 (d, 2H, $^4J_{HH}$ = 2.4, Ar), 7.10 (d, 2H, $^4J_{HH}$ = 2.4, Ar), 3.72 (td, 4H, $^3J_{HH}$ = 6.6, $^4J_{HH}$ = 1.2, N-CH_2), 2.14 (p, 2H, $^3J_{HH}$ = 6.6, CH_2), 1.47 (s, 18H, *t*Bu), 1.35 – 1.23 (m, 18H, *t*Bu). **FT-IR** (cm^{-1}): 2954.14 (C-H aromatic), 2867.63 (C-H aromatic), 1630.49 (C=N). **M.p.** 144 °C. Data are consistent with the literature.[13]

2.1.9. **Preparation of 1c′**

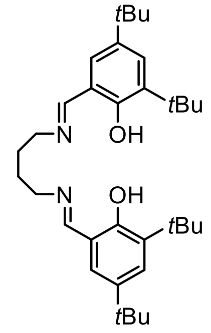

Following the general procedure and using 3,5-di-*tert*-butyl-2-hydroxybenzaldehyde (0.50 g, 2.13 mmol) and 1,4-diaminobutane (61.0 µL, 0.61 mmol) in EtOH (10 mL). Yield: 264 mg (83%). **^{1}H NMR** (400 MHz, $CDCl_3$): *δ* 13.86 (br s, 2H, OH), 8.36 (s, 2H, N=CH), 7.37 (d, $^4J_{HH}$ = 2.4, 2H, Ar), 7.07 (d, $^4J_{HH}$ = 2.4, 2H, Ar), 3.65 – 3.60 (m, 4H, NCH_2), 1.83 – 1.79 (m, 4H, CH_2), 1.44 (s, 18H, *t*Bu), 1.30 (s, 18H, *t*Bu). **FT-IR** (cm^{-1}): 2953 (C-H), 2869 (C-H), 1631 (C=N), 1465 (C=C), 1375 (C-N), 1251 (C-O). **ESI-MS** (positive ion, 4 kV): 521.4108, $[M+H]^+$ (calc. 521.4107) *m/z*. Data are consistent with the literature.[13]

2.1.10. **Preparation of 1d′**

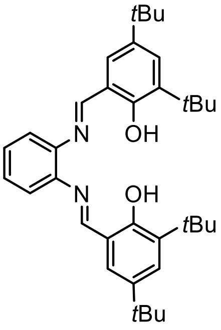

Following the general procedure and using 3,5-di-*tert*-butyl-2-hydroxybenzaldehyde (0.85 g, 3.61 mmol) and *o*-phenylendiamine (0.20 g, 1.80 mmol) in EtOH (20 mL). Yield: 458 mg (47%).**^{1}H NMR** (400 MHz, $CDCl_3$): *δ* 13.53 (br s, 2H, OH), 8.66 (s, 2H, N=CH), 7.44 (d, $^4J_{HH}$ = 2.9, 2H, Ar), 7.33 – 7.29 (m, 2H, Ar), 7.24 – 7.21 (m, 4H, Ar), 1.43 (s, 18H, *t*Bu), 1.32 (s, 18H, *t*Bu). Data are consistent with the literature. **FT-IR** (cm^{-1}): 2957 (C-H), 1617 (C=N), 1470 (C=C), 1361 (C-N), 1250 (C-O). **ESI-MS** (positive ion, 4 kV): 541.3808, $[M+H]^+$ (calc. 541.8806) *m/z*. Data are consistent with the literature.[14]

3. Synthesis of precatalysts

3.1. General Procedure

Ligand **1** and $Fe(OAc)_2$ were added to a flask (in air), EtOH was added and the suspension was refluxed for 2 hours. The resulting suspension was cooled to -30 °C and the solid collected by filtration. The solid residue was washed with cold ethanol (-30 °C, 3 x ~ 10 – 20 mL) and dried under vacuum.

3.1.1. **Preparation of 2a**

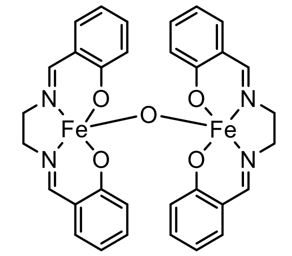

Following the general procedure and using **1a** (2.55 g, 9.50 mmol) and $Fe(OAc)_2$ (1.38 g, 7.93 mmol) and EtOH (100 mL). Isolated as a maroon powder. Yield: 2.27 g (87%). **^{1}H NMR** (500 MHz; $CDCl_3$): δ (all br.) 77.34, 47.98, 23.84, 19.50, 12.56, 9.82, 8.39, 7.31, 7.28 – 6.85, 3.97, 2.75, 1.34, 1.28, 0.45, -69.01. **FT-IR** (cm^{-1}): 2971.18 (C-H aromatic), 2907.71 (C-H aromatic), 1625.46 (C=N), 858.45 (Fe-O-Fe), 757.78 (=CH). **UV-Vis** (298 K, CH_3CN): $\lambda_{max,1}$ = 470 nm, $\varepsilon_1 = 6.08 \times 10^3$ $M^{-1}cm^{-1}$. **LIFDI-MS:** 660.0757, $[M]^+$ (calc. 660.0754) *m/z*. Data are consistent with the literature.[7]

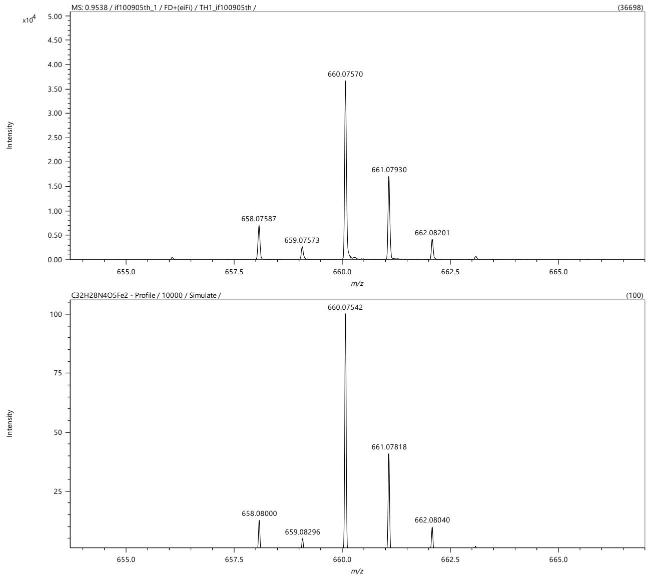

Figure S1. LIFDI-MS of **2a** (top) and simulated isotope pattern of **2a** (bottom).

3.1.2. **Preparation of 2b**

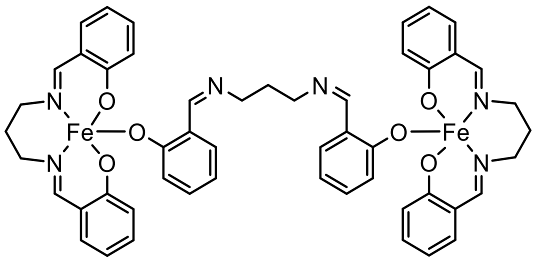

Following the general procedure and using **1b** (874 mg, 3.10 mmol) and $Fe(OAc)_2$ (430 mg, 2.47 mmol) and EtOH (30 mL). Isolated as a maroon powder. Yield: 564 mg (66%). **^{1}H NMR** (400 MHz; $CDCl_3$): featureless between δ 300 – (–)300. **FT-IR** (cm^{-1}): 2894.08 (aromatic C-H), 1613.87 (C=N), 854.29 (Fe-O-Fe), 759.36 (=CH). **UV-Vis** (298 K, CH_3CN): $\lambda_{max,1}$ = 291 nm, $\varepsilon_1 = 1.55 \times 10^4$ $M^{-1}cm^{-1}$; $\lambda_{max,2}$ = 314 nm, $\varepsilon_2 = 1.57 \times 10^4$ $M^{-1}cm^{-1}$; $\lambda_{max,3}$ = 420 nm, $\varepsilon_3 = 5.65 \times 10^3$ $M^{-1}cm^{-1}$, $\lambda_{max,4}$ = 483 nm, $\varepsilon_4 = 6.67 \times 10^3$ $M^{-1}cm^{-1}$.

LIFDI-MS: 952.2439, $[M]^+$ (calc. 952.2331) *m/z*. This compound has previously been characterised crystallographically.[15]

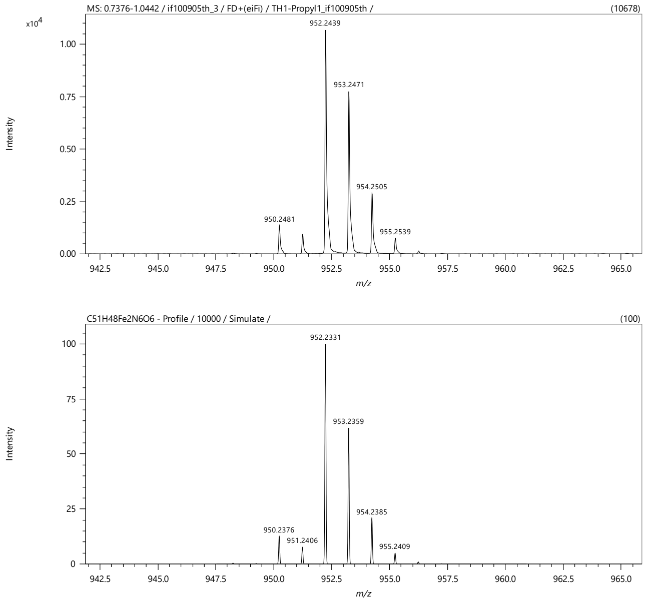

Figure S2. LIFDI-MS of **[Fe$_2$(1b)$_3$]** (top) and simulated isotope pattern of **[Fe$_2$(1b)$_3$]** (bottom).

3.1.3. **Preparation of 2c**

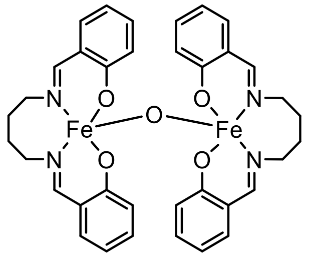

Following the general procedure and using **1c** (180 mg, 0.61 mmol) and $Fe(OAc)_2$ (88.0 mg, 0.51 mmol) and EtOH (30 mL). Isolated as a maroon powder. Yield: 152 mg (84%). **^{1}H NMR** ($CDCl_3$, 400 MHz): featureless between δ 300 – (–)300. **FT-IR** (cm^{-1}): 2924.25 (aromatic C-H), 1611.64 (C=N), 852.18 (Fe-O-Fe), 754.94 (=CH). **UV-Vis** (298 K, CH_3CN): $\lambda_{max,1}$ = 295 nm, $\varepsilon_1 = 1.05 \times 10^4$ M^{-1}cm^{-1}; $\lambda_{max,2}$ = 316 nm, $\varepsilon_2 = 1.09 \times 10^4$ M^{-1}cm^{-1}; $\lambda_{max,3}$ = 479 nm, $\varepsilon_3 = 3.44 \times 10^3$ M^{-1}cm^{-1}. **LIFDI-MS:** 716.1357, $[M]^+$ (calc. 716.1380) *m/z*.[16] A range of related isomers have been reported and characterised crystallographically.[17]

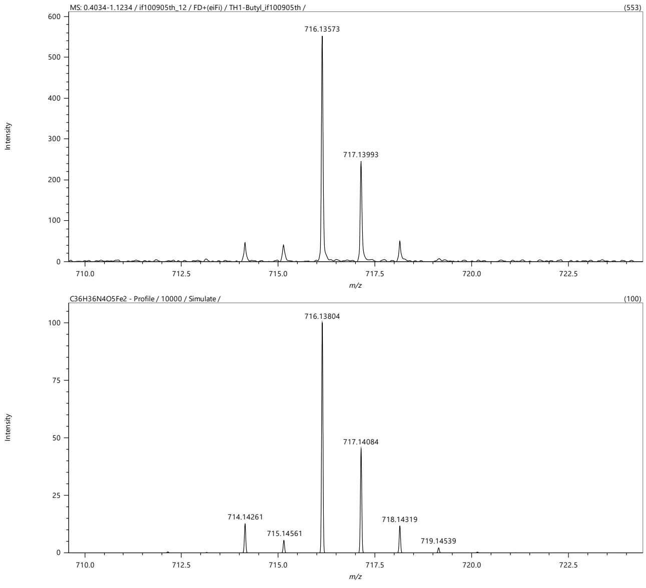

Figure S3. LIFDI-MS of **2c** (top) and simulated isotope pattern of **2c** (bottom).

3.1.4. Preparation of 2d

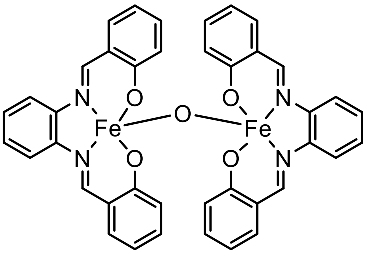

Following the general procedure and using **1d** (949 mg, 3.00 mmol) and $Fe(OAc)_2$ (435 mg, 2.50 mmol) and EtOH (100 mL). Isolated as a brown-red powder. Yield: 740 mg (78%). **^{1}H NMR** ($CDCl_3$, 500 MHz): *δ* (all br.) 82.91, 50.13, 30.14, 27.91, 12.65, 9.83, 9.61, 3.83, 2.64, 1.60, 0.89, -9.00, 71.47 **FT-IR** (cm^{-1}): 3012.65 (aromatic C-H), 1603.74 (C=N), 805.34 (Fe-O-Fe), 754.28 (=CH). **UV-Vis** (298 K, CH_3CN): $\lambda_{max,1}$ = 241 nm, $\varepsilon_1 = 2.04 \times 10^4\ M^{-1}cm^{-1}$; $\lambda_{max,2}$ = 295 nm, $\varepsilon_2 = 2.46 \times 10^4\ M^{-1}cm^{-1}$; $\lambda_{max,3}$ = 399 nm, $\varepsilon_3 = 9.46 \times 10^3\ M^{-1}cm^{-1}$. **LIFDI-MS:** 756.0721, $[M]^+$ (calc. 756.0755) *m/z*. Data are consistent with the literature.[18]

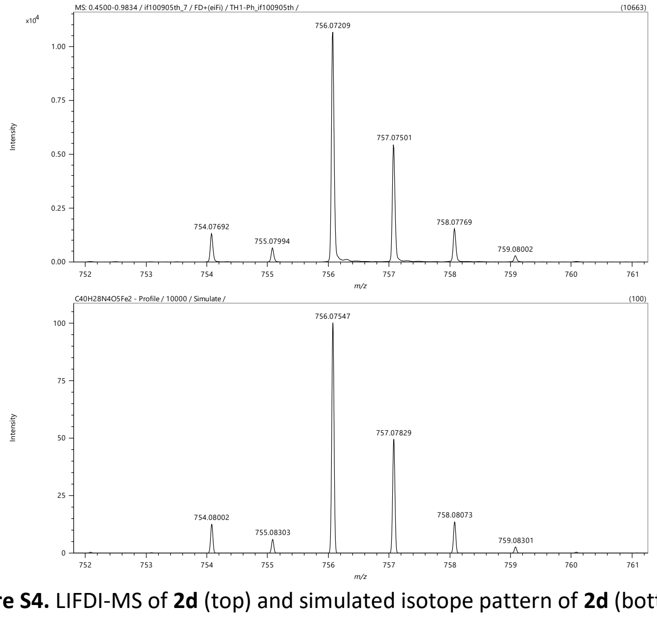

Figure S4. LIFDI-MS of **2d** (top) and simulated isotope pattern of **2d** (bottom).

3.1.5. **Preparation of 2e**

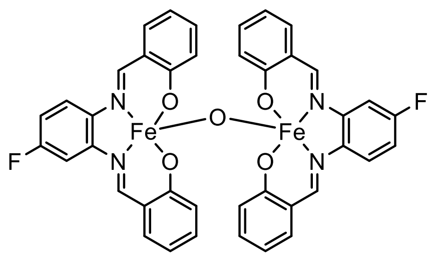

Following the general procedure and using **1e** (465 mg, 1.39 mmol) and $Fe(OAc)_2$ (280 mg, 1.26 mmol) and EtOH (20 mL). Isolated as a red powder. Yield: 385 mg (77%). **^{1}H NMR** ($CDCl_3$, 400 MHz): δ (all br.) 12.68, 9.94, 8.16, 6.14, 3.72, 2.78, 2.68, 0.42. **FT-IR** (cm^{-1}): 3041.32 (aromatic C-H), 1603.84 (C=N), 1312.24 (Ar–F), 849.68 (Fe-O-Fe), 755.10 (=CH). **UV-Vis** (298 K, CH_3CN): $\lambda_{max,1}$ = 231 nm, ε_1 = 1.67 × 10^4 $M^{-1}cm^{-1}$; $\lambda_{max,2}$ = 291 nm, ε_2 = 2.03 × 10^4 $M^{-1}cm^{-1}$; $\lambda_{max,3}$ = 408 nm, ε_3 = 7.64 × 10^3 $M^{-1}cm^{-1}$. **LIFDI-MS:** 792.0549, [*M*]$^+$ (calc. 792.0566) *m/z*.

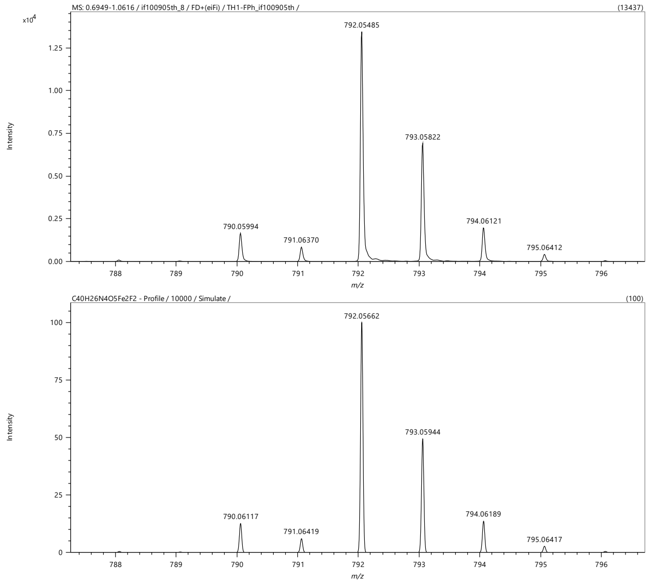

Figure S5. LIFDI-MS of **2e** (top) and simulated isotope pattern of **2e** (bottom).

3.1.6. **Preparation of 2f**

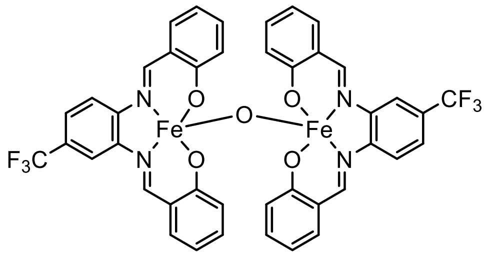

Following the general procedure and using **1f** (486 mg, 1.26 mmol) and $Fe(OAc)_2$ (200 mg, 1.15 mmol) and EtOH (20 mL). Isolated as an orange/red powder. Yield: 372 mg (72%). **^{1}H NMR** ($CDCl_3$, 400 MHz): δ (all br.) 12.75, 9.96, 9.39, 2.65. **FT-IR** (cm^{-1}): 3044.35 (aromatic C-H), 1601.23 (C=N), 1319.43 (C–F), 839.07 (Fe-O-Fe), 750.77 (=CH). **UV-Vis** (298 K, CH_3CN): $\lambda_{max,1}$ = 240 nm, ε_1 = 4.45 × 10^4 $M^{-1}cm^{-1}$; $\lambda_{max,2}$ = 290 nm, ε_2 = 5.55 × 10^4 $M^{-1}cm^{-1}$; $\lambda_{max,3}$ = 406 nm, ε_3 = 1.98 × 10^4 $M^{-1}cm^{-1}$. **LIFDI-MS:** 892.0454, [*M*]$^+$ (calc. 892.0503) *m/z*.

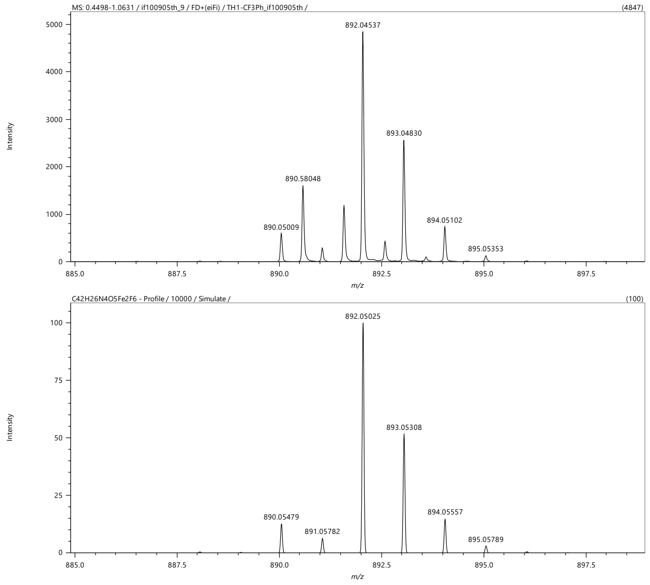

Figure S6. LIFDI-MS of **2f** (top) and simulated isotope pattern of **2f** (bottom).

3.1.7. **Preparation of 2a'**

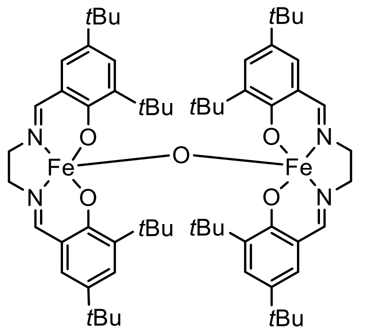

Following the general procedure and using **1a'** (360 mg, 0.73 mmol) and $Fe(OAc)_2$ (106 mg, 0.61 mmol) and propan-2-ol (20 mL). Isolated as a dark red powder. Yield: 230 mg (68%). **^{1}H NMR** (500 MHz; $CDCl_3$): featureless between δ 300 – (−)300. **FT-IR** (cm^{-1}): 2950.248 (aromatic C-H), 1622.71 (C=N), 837.02 (Fe-O-Fe), 747.01 (=CH). **UV-Vis** (298 K, CH_3CN): $\lambda_{max,1}$ = 301 nm, $\varepsilon_1 = 1.54 \times 10^4$ $M^{-1}cm^{-1}$; $\lambda_{max,2}$ = 334 nm, $\varepsilon_2 = 1.67 \times 10^4$ $M^{-1}cm^{-1}$; $\lambda_{max,3}$ = 445 nm, $\varepsilon_3 = 4.31 \times 10^3$ $M^{-1}cm^{-1}$, $\lambda_{max,4}$ = 535 nm, $\varepsilon_4 = 7.27 \times 10^3$ $M^{-1}cm^{-1}$. Data are consistent with the literature.[19]

3.1.8. **Preparation of [Fe(1b')OAc]**

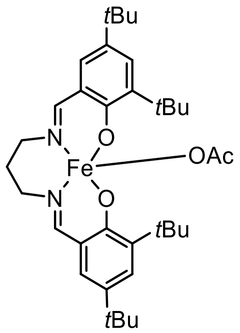

Following the general procedure and using **1b'** (262 mg, 0.52 mmol) and $Fe(OAc)_2$ (77.0 mg, 0.44 mmol) and propan-2-ol (20 mL). Isolated as a dark purple powder. Yield: 138 mg (55%). **^{1}H NMR** (400 MHz; $CDCl_3$): featureless between δ 300 – (−)300. **M.p.** 290 °C. **FT-IR** (cm^{-1}): 2954.29 (aromatic C-H),

1611.28 (C=N), 842.22 (Fe-O-Ac), 750.36 (=CH). **UV-Vis** (298 K, CH_3CN): $\lambda_{max,1}$ = 225 nm, $\varepsilon_1 = 1.24 \times 10^4$ $M^{-1}cm^{-1}$; $\lambda_{max,2}$ = 240 nm, $\varepsilon_2 = 1.24 \times 10^4$ $M^{-1}cm^{-1}$; $\lambda_{max,3}$ = 269 nm, $\varepsilon_3 = 8.52 \times 10^3$ $M^{-1}cm^{-1}$; $\lambda_{max,4}$ = 300 nm, $\varepsilon_4 = 3.38 \times 10^3$ $M^{-1}cm^{-1}$; $\lambda_{max,5}$ = 331 nm, $\varepsilon_5 = 3.47 \times 10^3$ $M^{-1}cm^{-1}$; $\lambda_{max,6}$ = 515 nm, $\varepsilon_6 = 1.04 \times 10^3$ $M^{-1}cm^{-1}$. **LIFDI-MS:** 619.3094, $[M]^+$ (calc. 619.3193) *m/z*. The title compound has previously been reported without any data alongside the μ-oxo analogue (**2b'**) for which the data are complementary to our observations.[20]

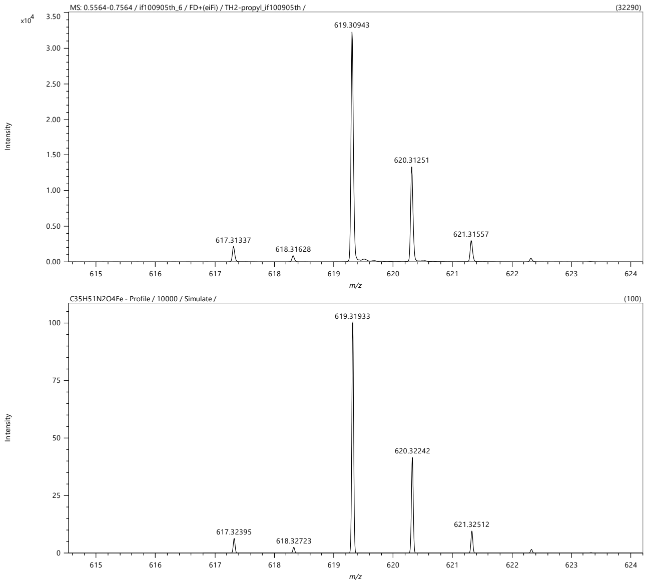

Figure S7. LIFDI-MS of [Fe(**1b'**)(OAc)] ("**2b'**", top) and simulated isotope pattern of [Fe(**1b'**)(OAc)] (bottom).

3.1.9. **Preparation of 2c'**

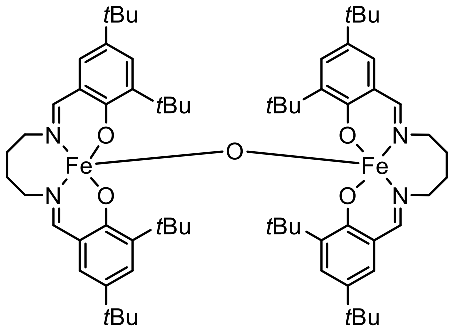

Following the general procedure and using **1c'** (190 mg, 0.36 mmol) and $Fe(OAc)_2$ (76.3 mg, 0.44 mmol) and EtOH (5 mL). Isolated as a brown-purple powder. Yield: 191 mg (90%). **^{1}H NMR** ($CDCl_3$, 400 MHz): featureless between δ 300 – (–)300. **FT-IR** (cm^{-1}): 2950.00 (aromatic C-H), 1608.20 (C=N), 839.89 (Fe-O-Fe). **UV-Vis** (298 K, CH_3CN): $\lambda_{max,1}$ = 267 nm, $\varepsilon_1 = 2.42 \times 10^4$ $M^{-1}cm^{-1}$; $\lambda_{max,2}$ = 303 nm, $\varepsilon_2 = 8.98 \times 10^3$ $M^{-1}cm^{-1}$; $\lambda_{max,3}$ = 331 nm, $\varepsilon_3 = 1.06 \times 10^4$ $M^{-1}cm^{-1}$, $\lambda_{max,4}$ = 530 nm, $\varepsilon_4 = 4.88 \times 10^3$ $M^{-1}cm^{-1}$. **LIFDI-MS:** 1164.6390, $[M]^+$ (calc. 1164.6391) *m/z*.

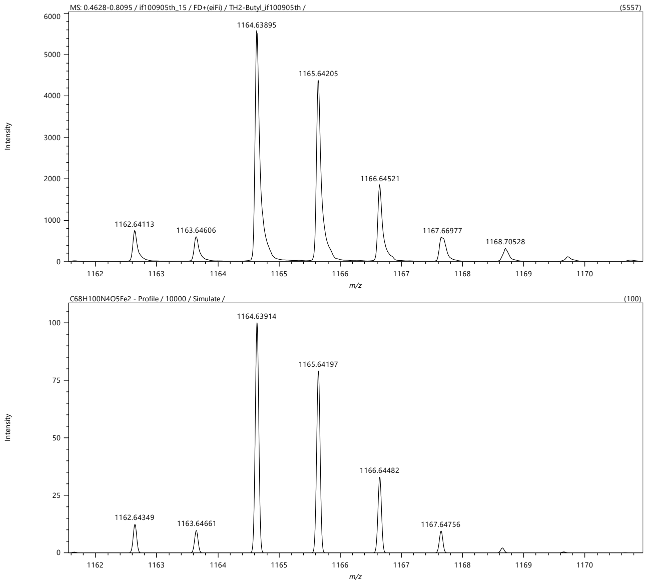

Figure S8. LIFDI-MS of **2f** (top) and simulated isotope pattern of **2f** (bottom).

3.1.10. **Preparation of 2d’**

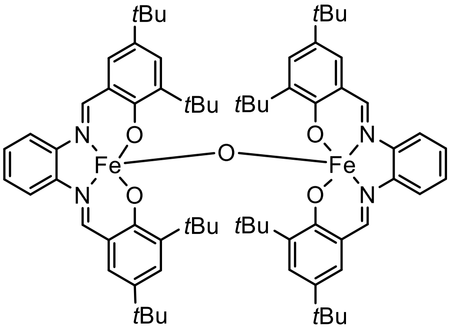

Following the general procedure and using **1d’** (372 mg, 0.69 mmol) and $Fe(OAc)_2$ (104 mg, 0.60 mmol) and EtOH (20 mL). Isolated as a red powder. Yield: 250 mg (69%). **^{1}H NMR** ($CDCl_3$, 500 MHz): *δ* (all br.) 11.88, 9.84, 9.00, 6.64, 0.18. **FT-IR** (cm^{-1}): 2952.67 (aromatic C-H), 2865.58 (aromatic C-H), 1598.97 (C=N), 835.98 (Fe-O-Fe), 739.82 (=CH). **UV-Vis** (298 K, CH_3CN): $\lambda_{max,1}$ = 244 nm, $\varepsilon_1 = 6.58 \times 10^3$ $M^{-1}cm^{-1}$; $\lambda_{max,2}$ = 302 nm, $\varepsilon_2 = 8.43 \times 10^3$ $M^{-1}cm^{-1}$; $\lambda_{max,3}$ = 442 nm, $\varepsilon_3 = 3.09 \times 10^3$ $M^{-1}cm^{-1}$. **LIFDI-MS:** 1204.5772, $[M]^+$ (calc. 1204.5766) *m/z*. Data are consistent with the literature.[19]

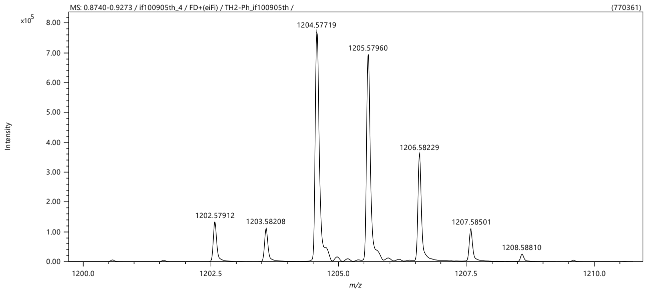

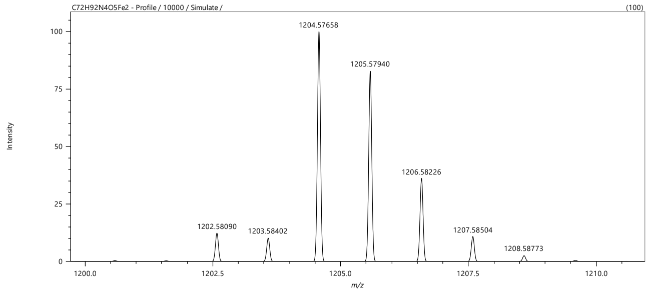

Figure S9. LIFDI-MS of **2d'** (top) and simulated isotope pattern of **2d'** (bottom).

3.1.11. Preparation of **5**

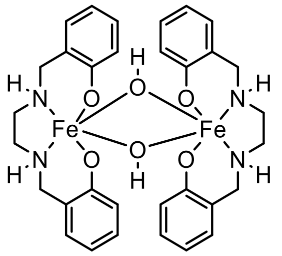

Ligand ^{H}salan (99.5 mg, 0.37 mmol) and $Fe(OAc)_2$ (64.0 mg, 0.37 mmol) were added to a flask (in air), EtOH (10 mL) was added and the suspension was refluxed for 2 hours. The resulting deep-red solution was concentrated to dryness and the residue recrystallised by slow diffusion of pentane into a concentrated DCM to give a red-purple amorphous solid. Yield: 112 mg (88%). Data are consistent with the literature.

^{1}H NMR ($CDCl_3$, 500 MHz): *δ* (all br.) 57.3, 50.1, 31.2, 2.4, -36.6. **UV-Vis** (298 K, CH_3CN): $\lambda_{max,1}$ = 232 nm, ε_1 = 2.00× 10^4 $M^{-1}cm^{-1}$; $\lambda_{max,2}$ = 277 nm, ε_2 = 1.51 × 10^3 $M^{-1}cm^{-1}$; $\lambda_{max,3}$ = 313 nm, ε_3 = 8.72 × 10^3 $M^{-1}cm^{-1}$; $\lambda_{max,4}$ = 479 nm, ε_4 = 5.51 × 10^3 $M^{-1}cm^{-1}$. Data is consistent with the literature.[21]

4. UV-vis spectra

4.1. R^1 = H

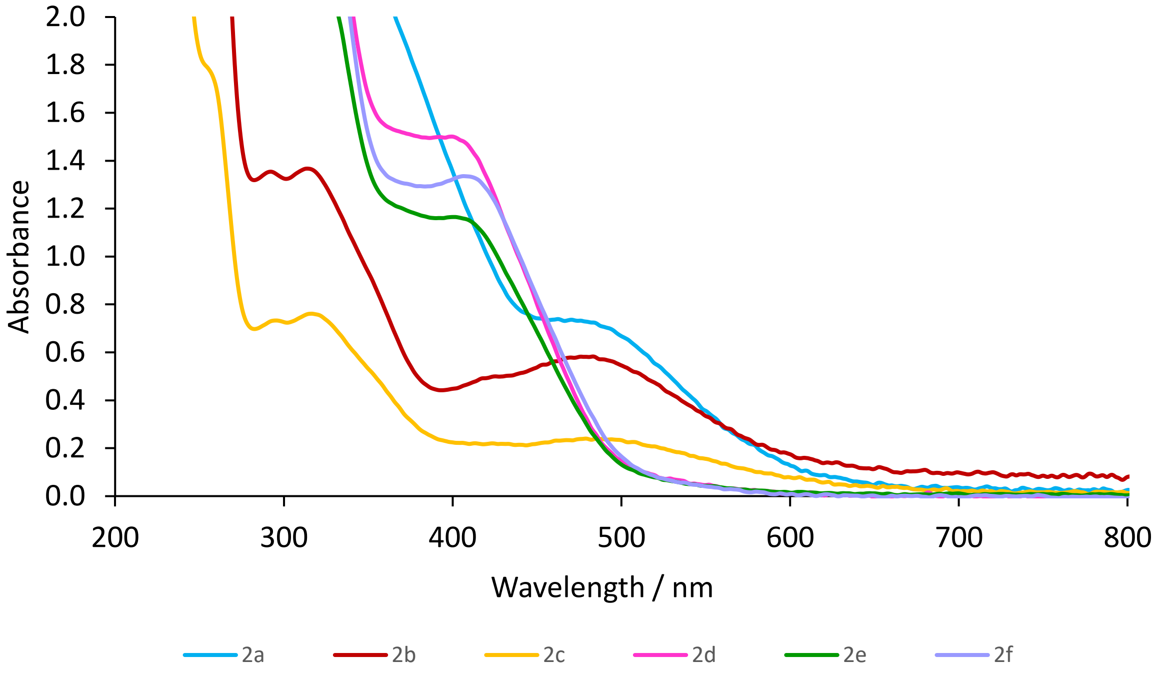

4.2. R^1 = tBu

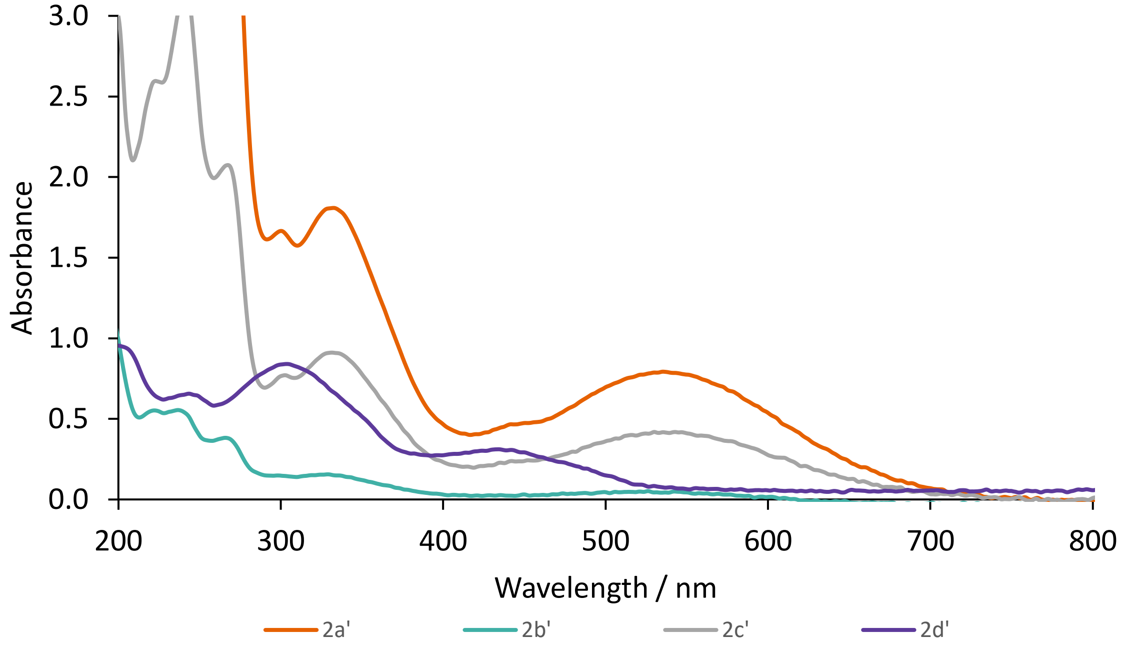

4.3. Comparison of all ethyl backbone [Fe] complexes

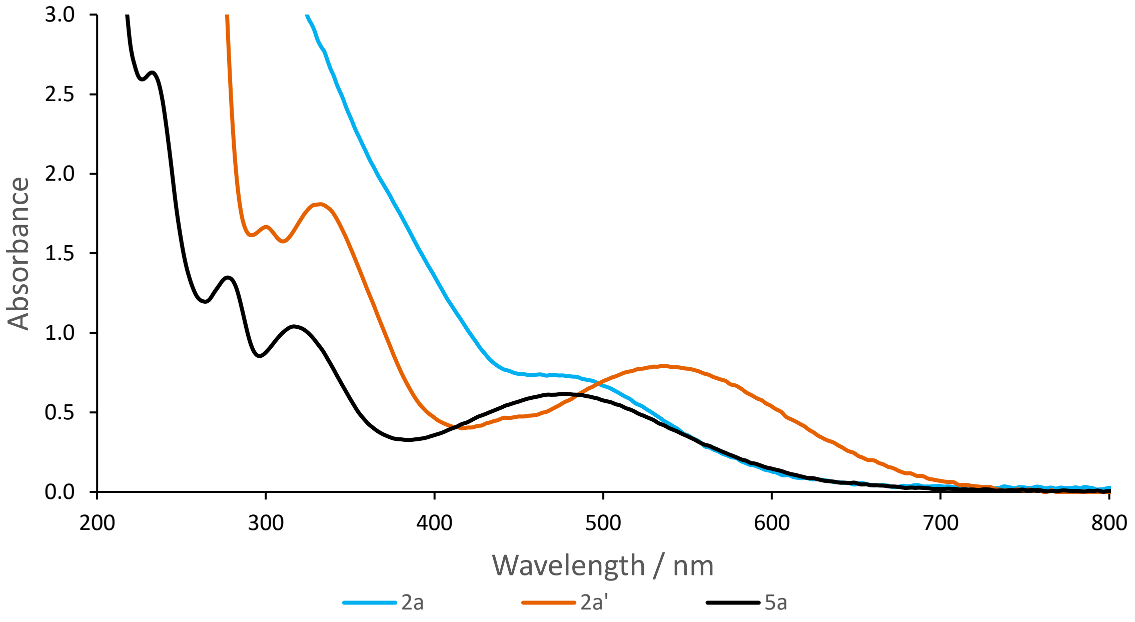

5. Kinetic experiments

5.1. General procedure for the trimerization of phenylacetylene

Precatalysts **2** (2.5 µmol, 1 mol%), phenylacetylene (27.5 µL, 0.25 mmol), 1,3,5-trimethoxybenzene (42.0 mg, 0.25 mmol) and CD_3CN (600 µL) were added a J Young valve NMR tube. To this, pinacolborane (14.5 µL, 0.10 mmol) was added and the kinetic experiment ran (^{1}H NMR, 400 MHz). The kinetic data was analysed by consumption of the alkynyl proton vs. the 1,3,5-trimethoxybenzene internal standard.

5.1.1. Precatalysts **2a** and **2a’**

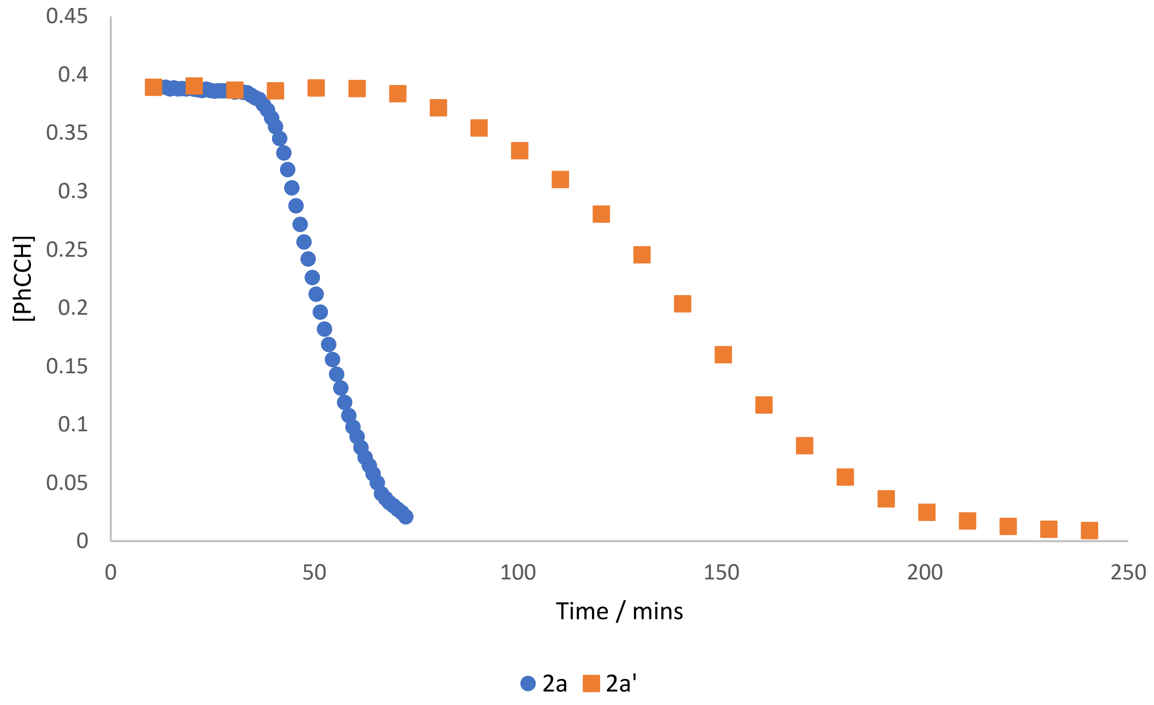

5.1.2. Precatalysts **2b** and **2b’**

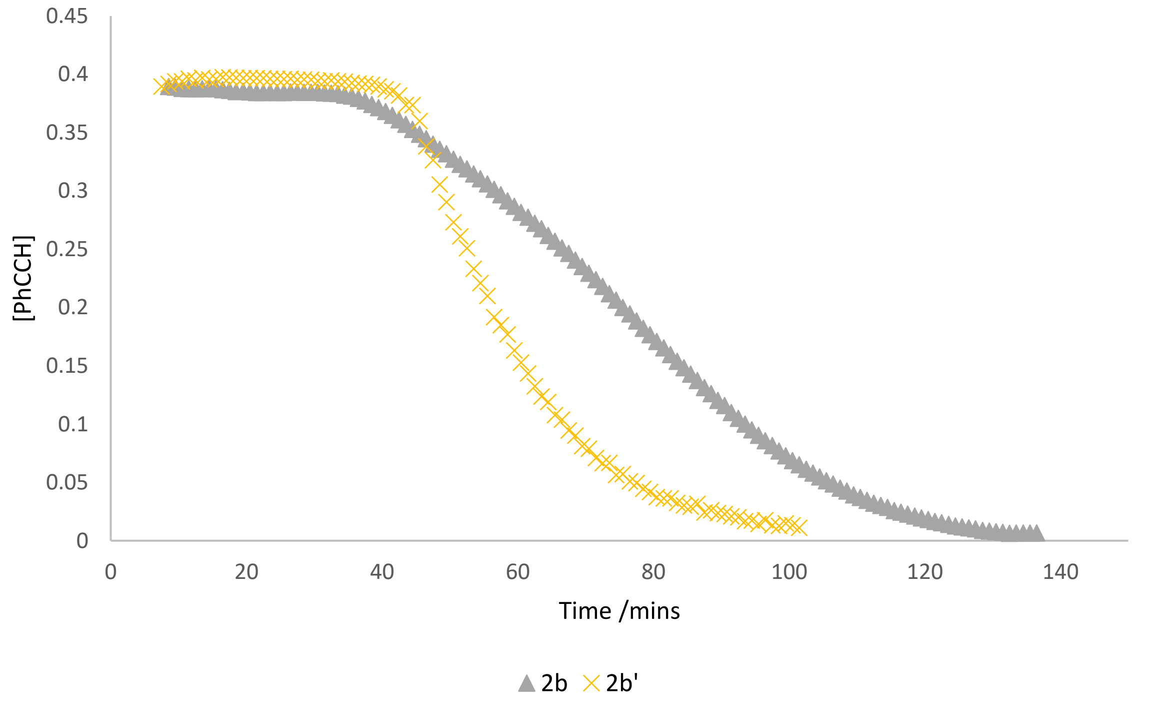

5.1.3. Precatalysts **2c** and **2c'**

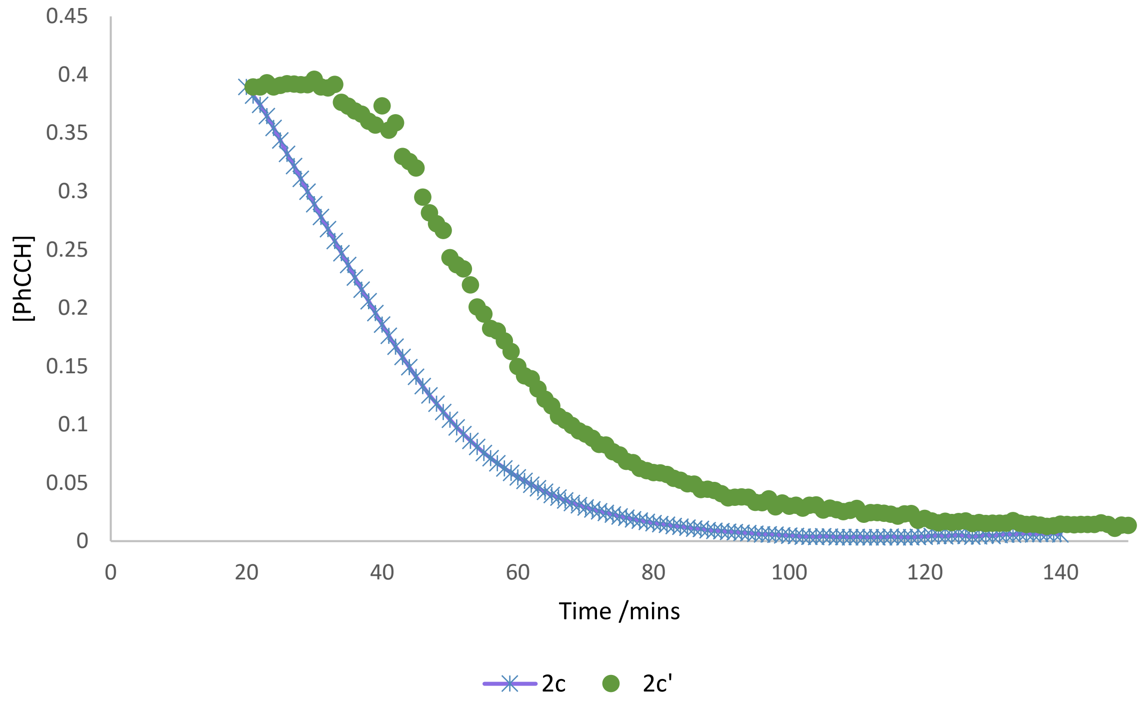

5.1.4. Precatalysts **2d** and **2d'**

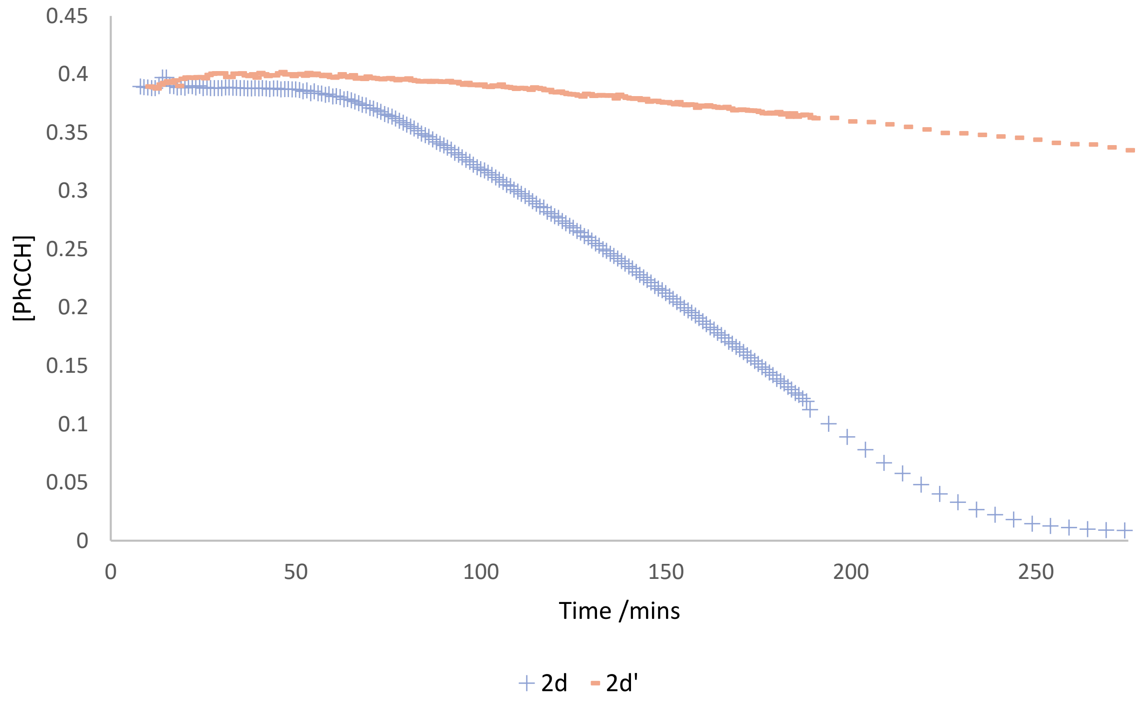

5.1.5. Precatalysts **2e** and **2f**

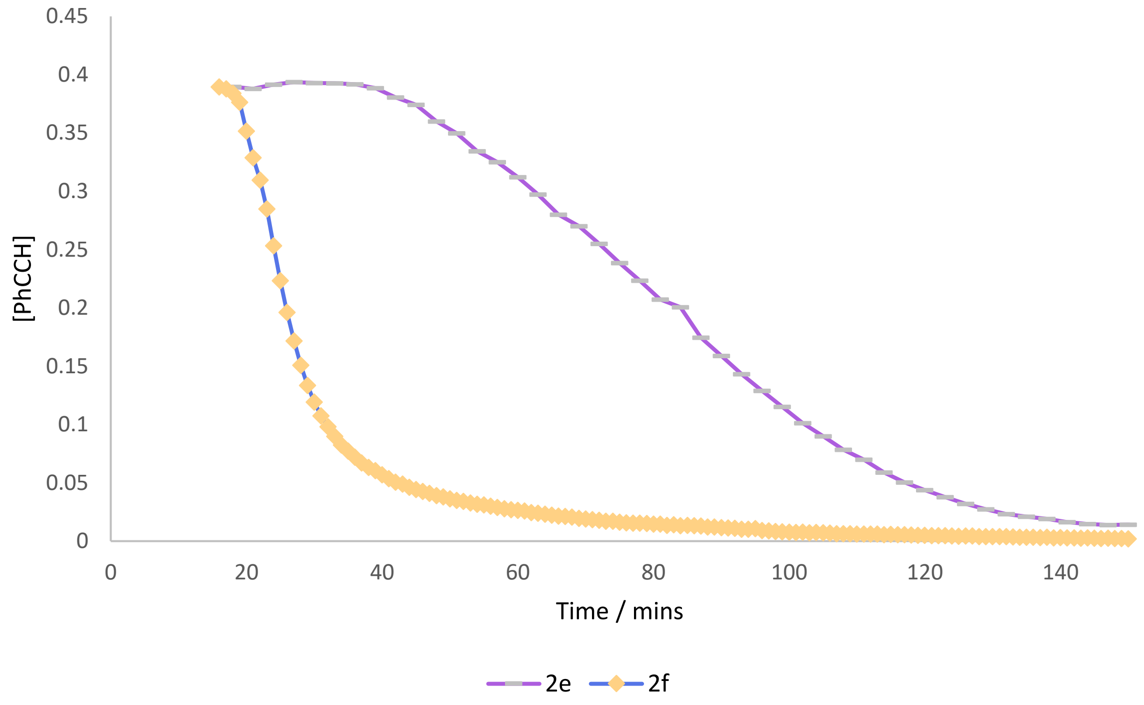

5.1.6. Backbone variations (**2**)

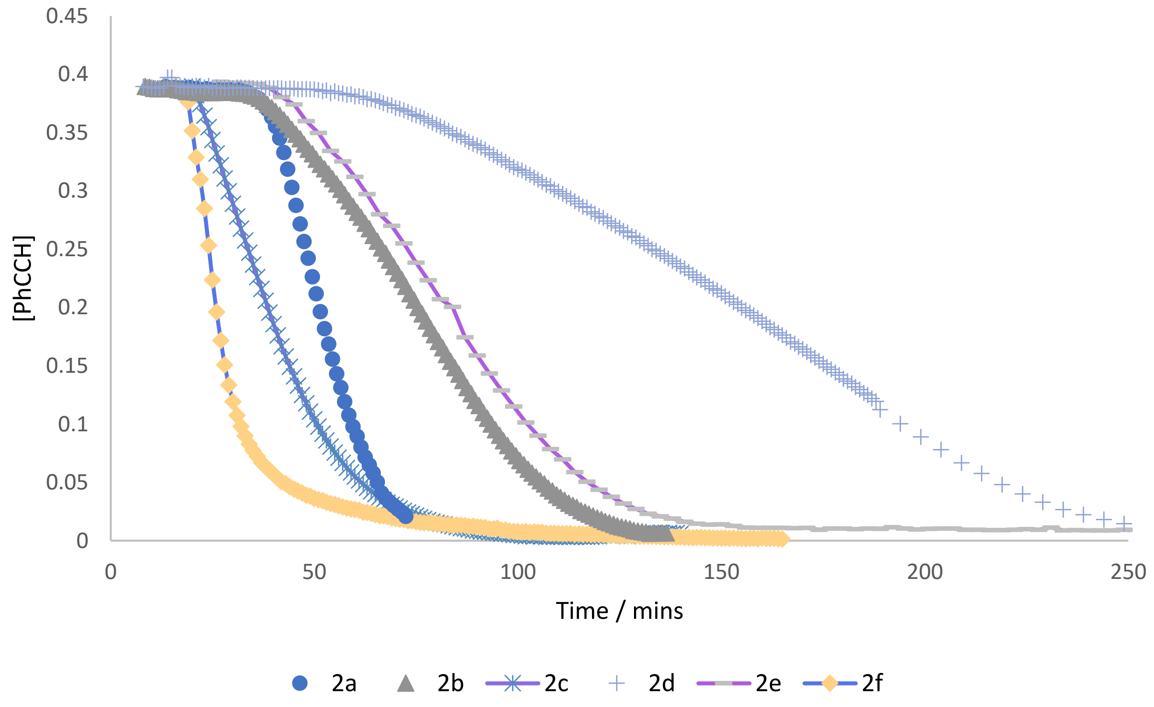

5.1.7. Backbone variation (**2'**)

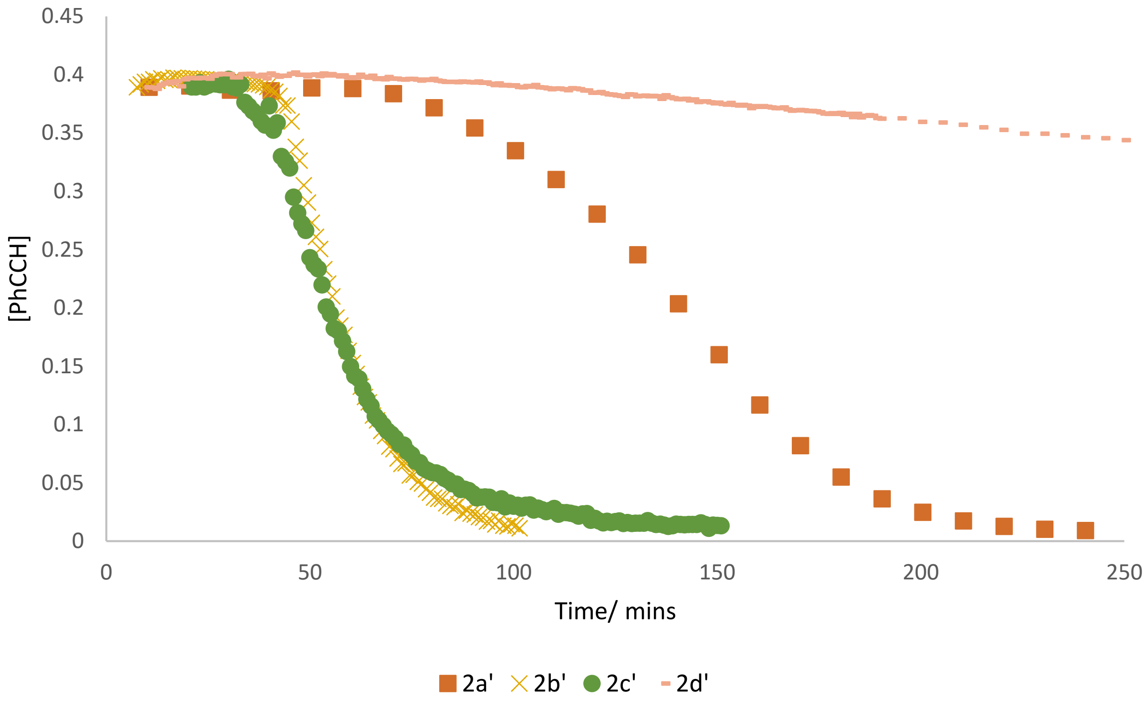

5.2. Trimerization of 4-*tert*-butylphenylacetylene

Precatalysts **2a**, **5** or **3a** (2.5 µmol, 1 mol%), 4-*tert*-butylphenylacetylene (46.5 µL, 0.25 mmol), 1,3,5-trimethoxybenzene (42.0 mg, 0.25 mmol) and CD_3CN (500 µL) were added a J Young NMR tube. To this, pinacolborane (14.5 µL, 0.10 mmol) was added and the kinetic experiment ran (^{1}H NMR, 400 MHz). The kinetic data was analysed by consumption of the alkynyl proton vs. the 1,3,5-trimethoxybenzene internal standard.

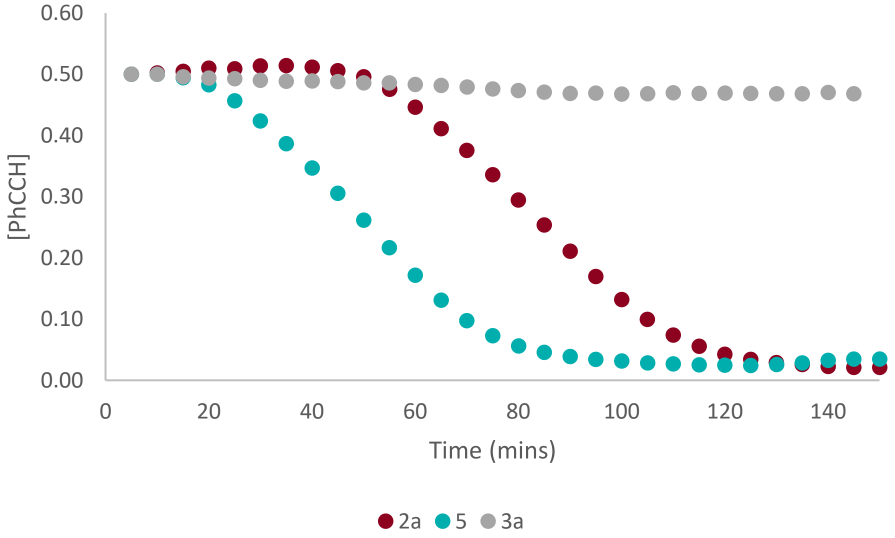

5.3. Catalysts inhibition experiments

Precatalysts **2a** or **2f** (2.5 μmol, 1 mol%), phenylacetylene (27.5 μL, 0.25 mmol), 1,3,5-trimethoxybenzene (14.0 mg, 0.08 mmol) and CD_3CN (600 μL) were added a J Young NMR tube. To this, pinacolborane (14.5 μL, 0.10 mmol) was added and the reaction was allowed to pre-mix for varying induction times before inhibitor (Hg (excess) or PMe_3 (0.25 μL, 2.5 μmol) was added. The kinetic data was analysed by consumption of the alkynyl proton vs. the 1,3,5-trimethoxybenzene internal standard.

5.3.1. Inhibition of **2a** with Hg

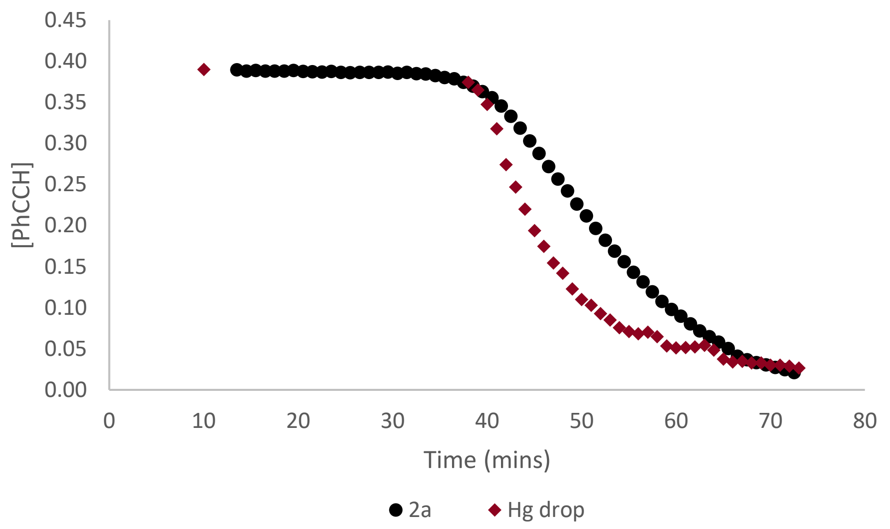

5.3.2. Inhibition of **2f** with Hg and PMe_3

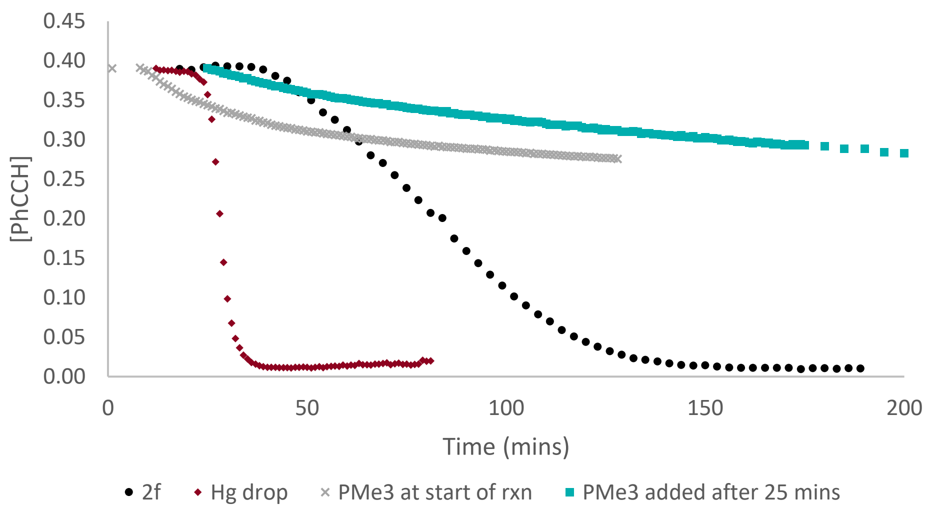

6. Trimerisation with 2a’

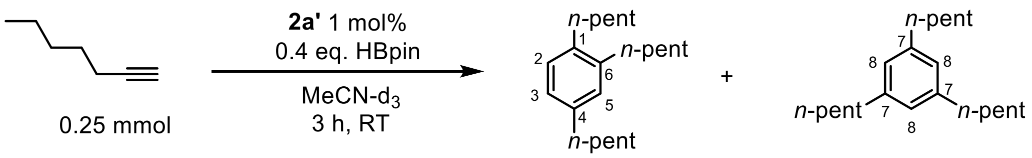

Precatalyst **2a’** (2.8 mg, 2.5 µmol), hept-1-yne (32.8 µL, 0.25 mmol), 1,3,5-trimethoxybenzene (21.0 mg, 0.12 mmol) and CD_3CN (600 µL) were added a J Young NMR tube. To this, pinacolborane (14.5 µL, 0.10 mmol) was added and monitored at RT after 3 h.

In-situ **^{1}H NMR** (CD_3CN, 400 MHz): δ 7.14 – 6.87 (m, 3H, $C^{2,3,5}$-*H*), 6.81 (s, (1,3,5 isomer, C^8-*H*)).[18]

Spectroscopic yield >99%. Isomeric ratio of 1,2,4 to 1,3,5: 45:55

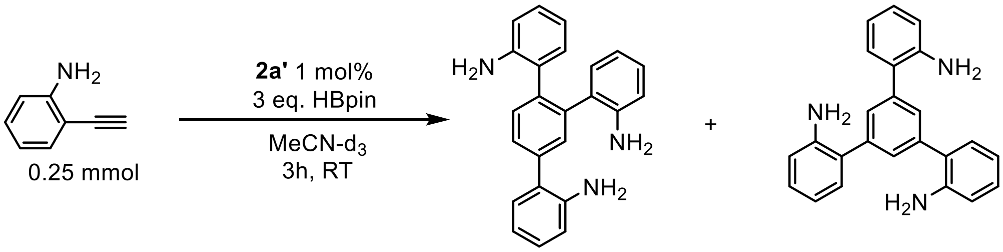

Precatalyst **2a’** (2.8 mg, 2.5 µmol), 2-ethynylaniline (28.4 µL, 0.25 mmol), 1,3,5-trimethoxybenzene (21.0 mg, 0.12 mmol) and CD_3CN (600 µL) were added a J Young NMR tube. To this, pinacolborane (109 µL, 0.75 mmol) was added and monitored at RT after 3 h. Solvent removed *in vacuo*, redissolved in minimal CH_2Cl_2 and taken through a silica plug. Crude ^{1}H NMR taken of product.

Spectroscopic yield >99%. Isomeric ratio of 1,2,4 to 1,3,5: 100:0

^{1}H NMR ($CDCl_3$, 400 MHz): δ 7.58-7.52 (m, 3H), 7.22 (d, $^3J_{HH}$ = 7.48, 1H), 7.17 (t, $^3J_{HH}$ = 7.71, 1H), 7.01 (q, $^3J_{HH}$ = 8.2, 2H), 6.97-6.89 (m, 2H), 6.84 (t, $^3J_{HH}$ = 7.93, 1H), 6.7 (d, $^3J_{HH}$ = 7.93, 1H), 6.65-6.57 (m, 4H).[18]

7. Stoichiometric reactions

7.1. General procedure for the treatment of **2** with HBpin

Precatalyst **2** (3.5 µmol) was added to a J Young NMR tube and CD_3CN or C_6D_6 (500 µL) and to this suspension pinacolborane (5.0 µL, 35.0 µmol) was added. The ^{1}H and ^{11}B{^{1}H} NMR spectra were recorded after 5 minutes, 1 hour and 48 hours.

7.1.1. **2a**

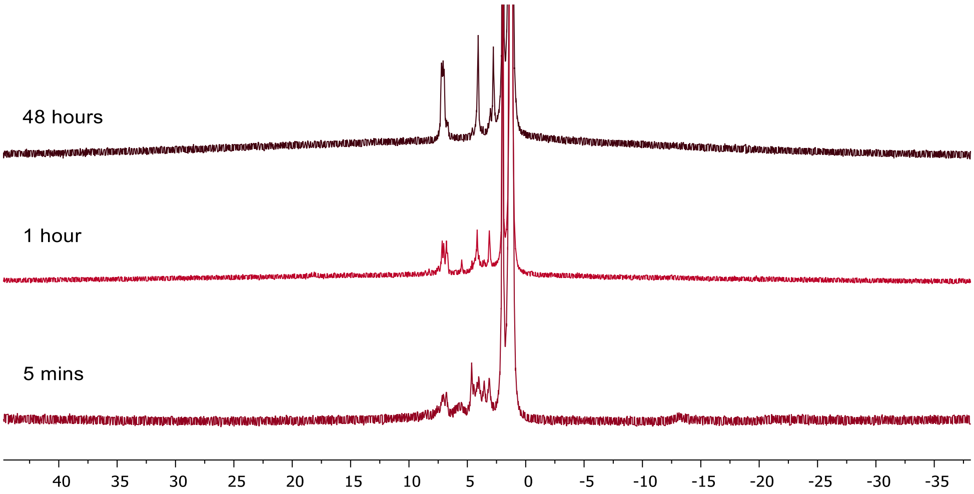

Figure S10. ^{1}H NMR stacked spectrum of **2a** with HBpin (CD_3CN, 400 MHz).

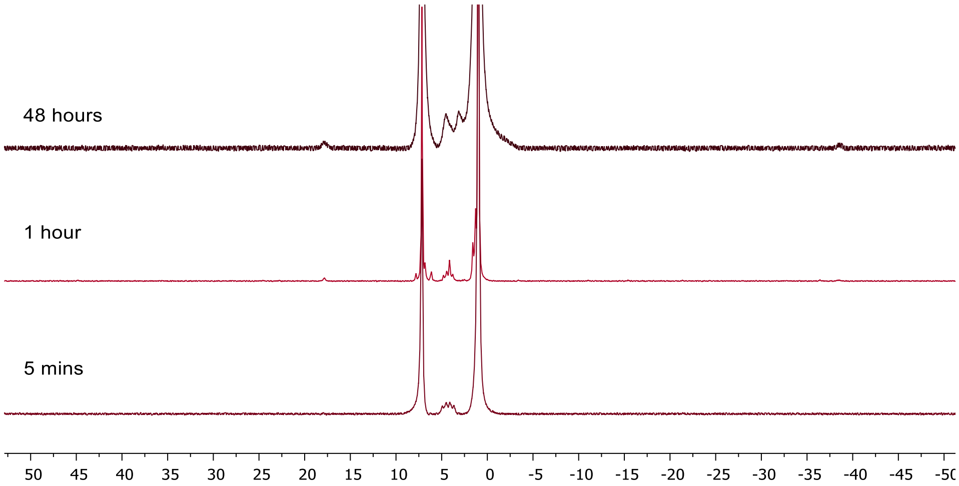

Figure S11. ^{1}H NMR stacked spectrum of **2a** with HBpin (C_6D_6, 400 MHz).

7.1.2. **2b**

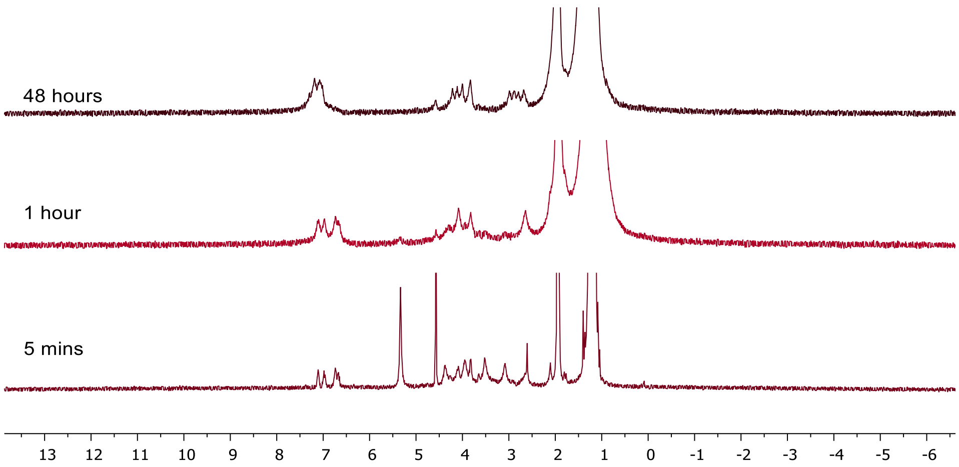

Figure S12. ^{1}H NMR stacked spectrum of **2b** with HBpin (CD_3CN, 400 MHz).

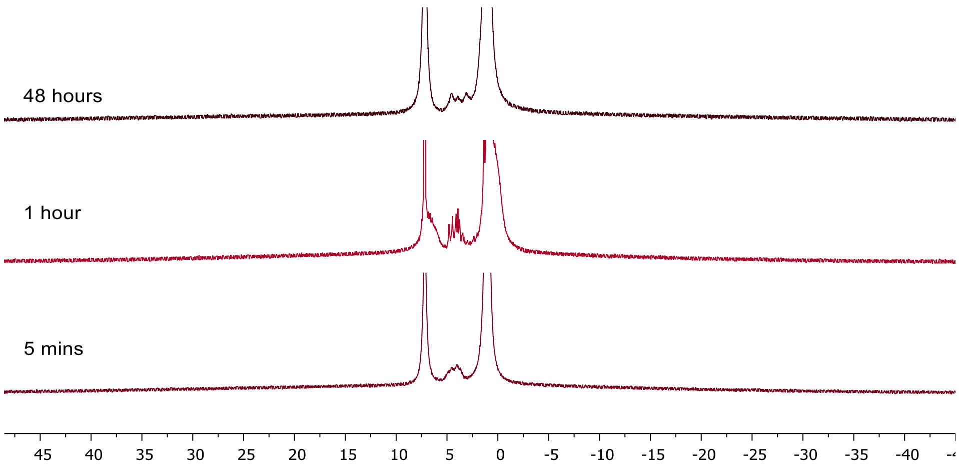

Figure S13. ^{1}H NMR stacked spectrum of **2b** with HBpin (C_6D_6, 400 MHz).

7.1.3. **2d**

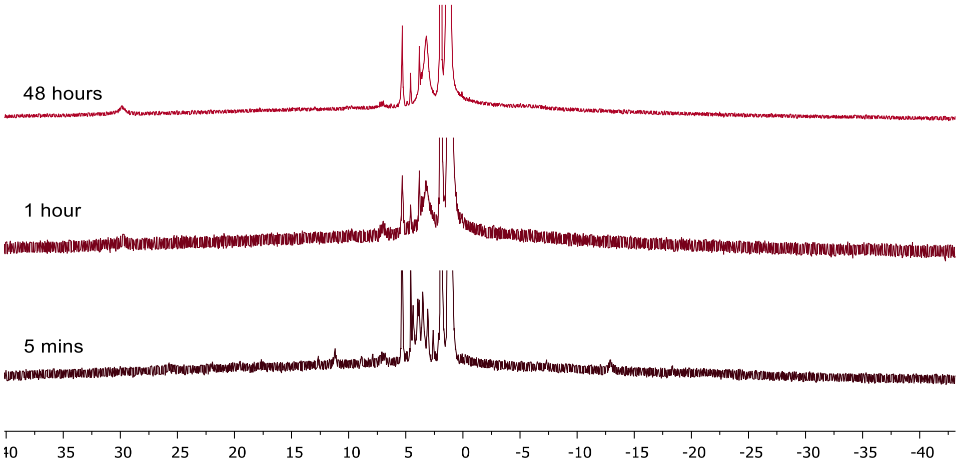

Figure S14. ^{1}H NMR stacked spectrum of **2d** with HBpin (CD_3CN, 400 MHz).

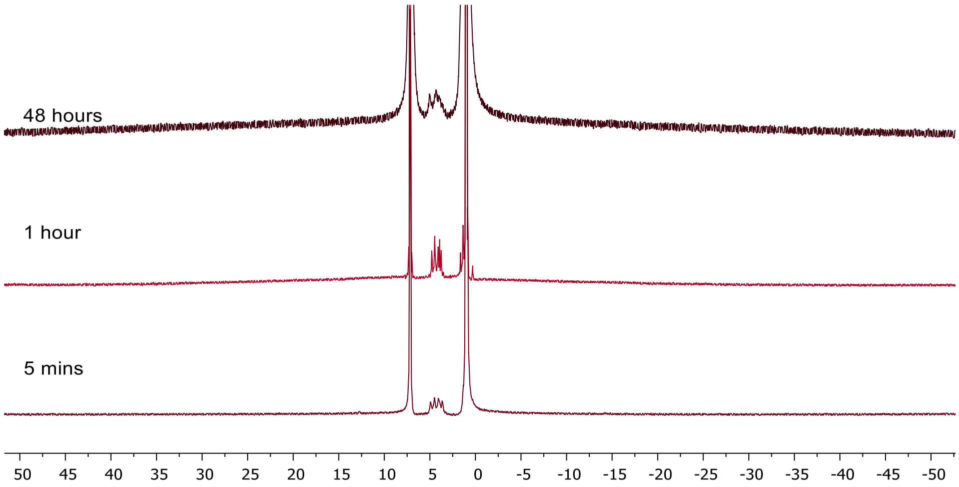

Figure S15. ^{1}H NMR stacked spectrum of **2d** with HBpin (C_6D_6, 400 MHz).

7.1.4. **2e**

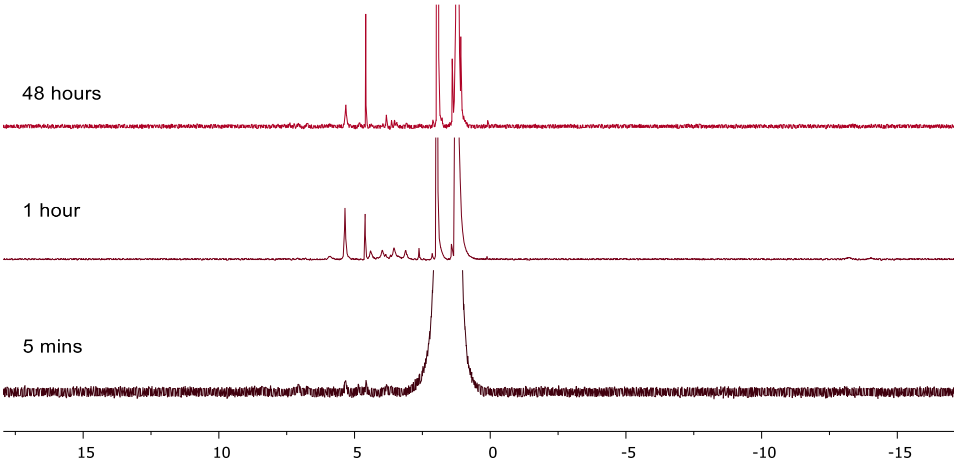

Figure S16. ^{1}H NMR stacked spectrum of **2e** with HBpin (CD_3CN, 400 MHz).

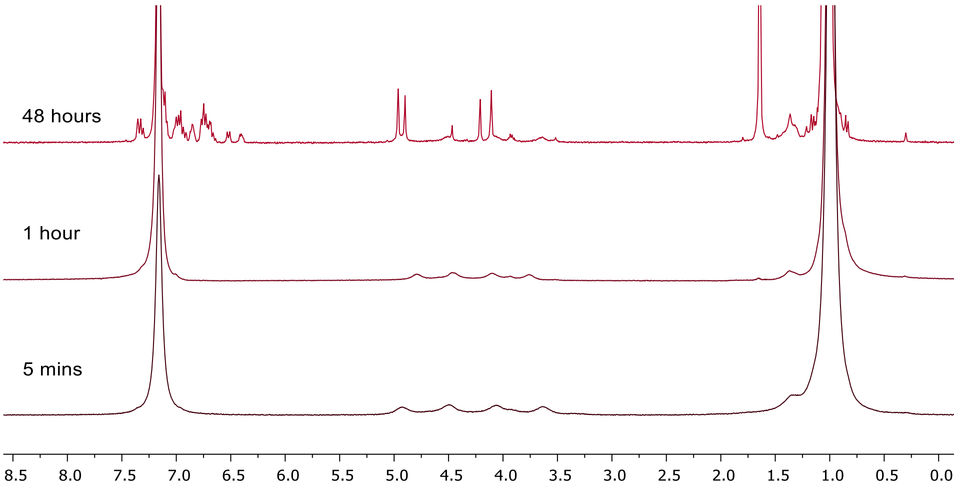

Figure S17. ^{1}H NMR stacked spectrum of **2e** with HBpin (C_6D_6, 400 MHz).

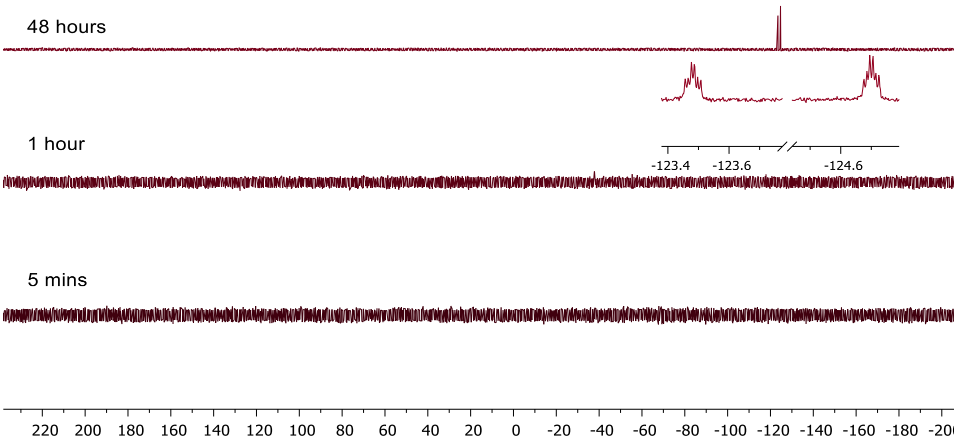

Figure S18. ^{19}F NMR stacked spectrum of **2e** with HBpin (C_6D_6, 400 MHz).

7.1.5. **2f**

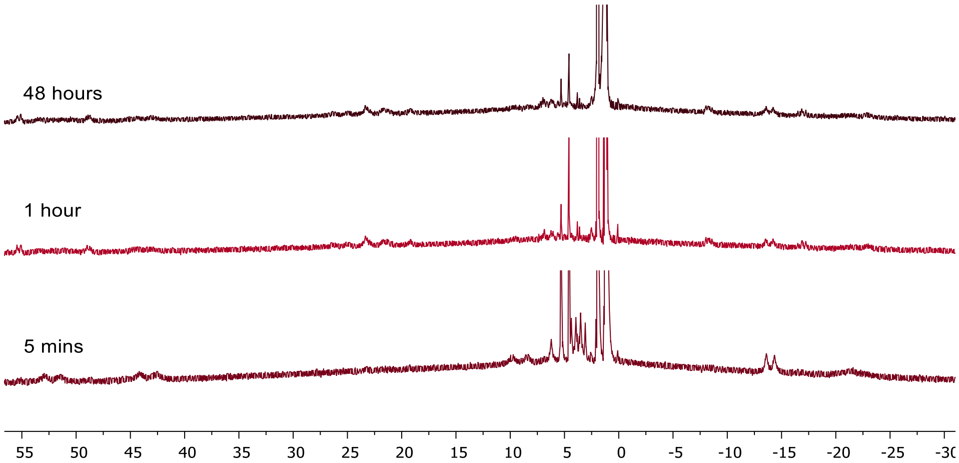

Figure S19. ^{1}H NMR stacked spectrum of **2f** with HBpin (CD_3CN, 400 MHz).

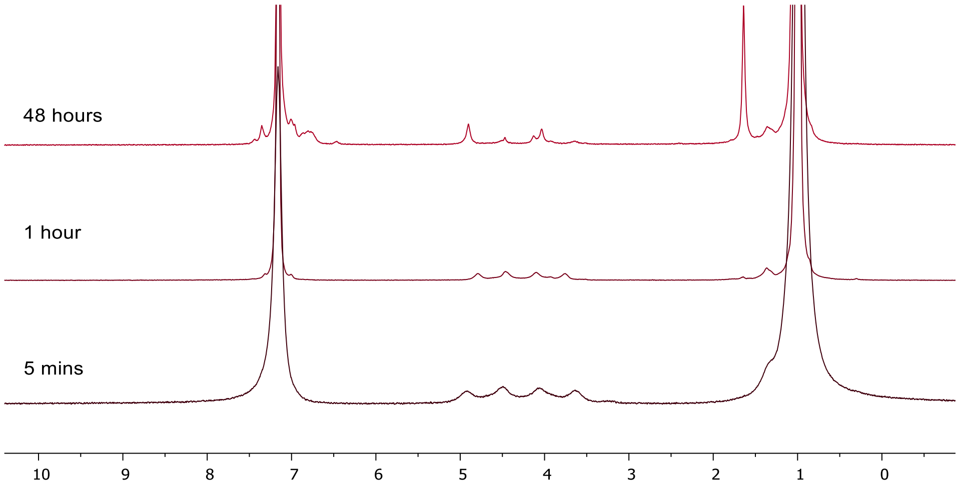

Figure S20. ^{1}H NMR stacked spectrum of **2f** with HBpin (C_6D_6, 400 MHz).

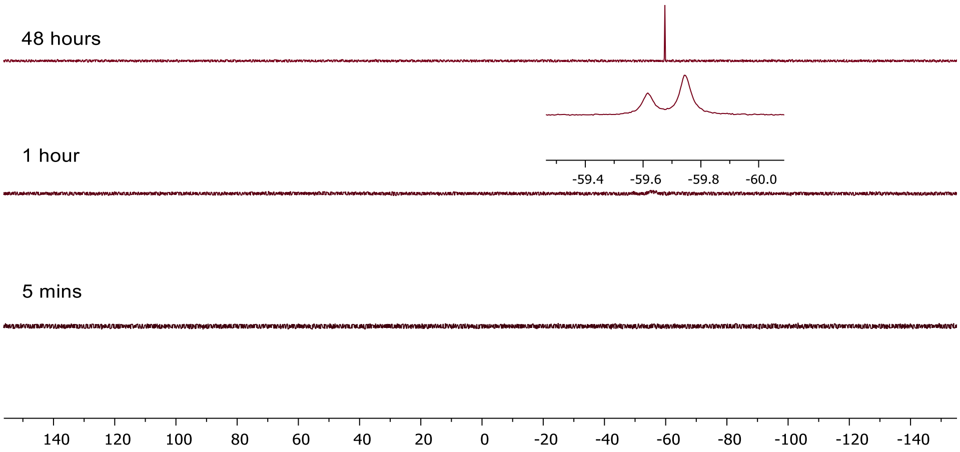

Figure S21. ^{19}F NMR stacked spectrum of **2f** with HBpin (C_6D_6, 400 MHz).

7.1.6. **2a′**

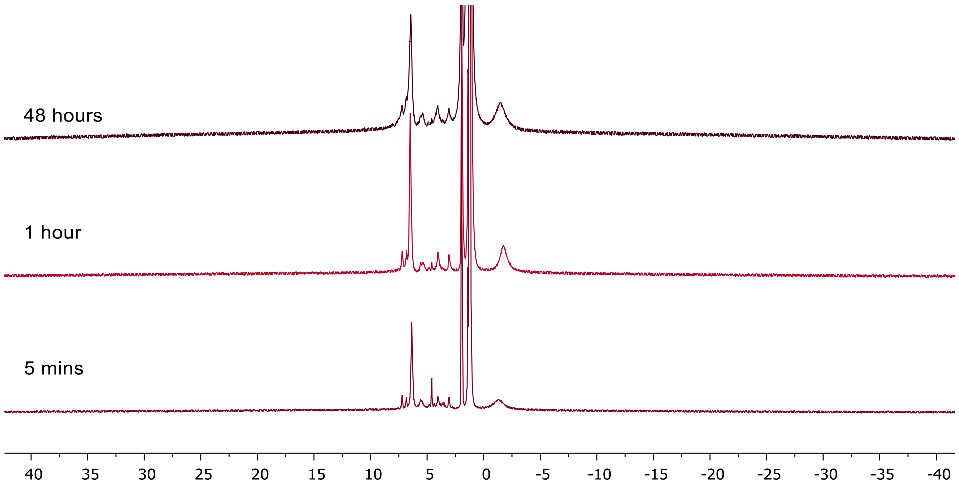

Figure S22. ^{1}H NMR stacked spectrum of **2a′** with HBpin (CD_3CN, 400 MHz).

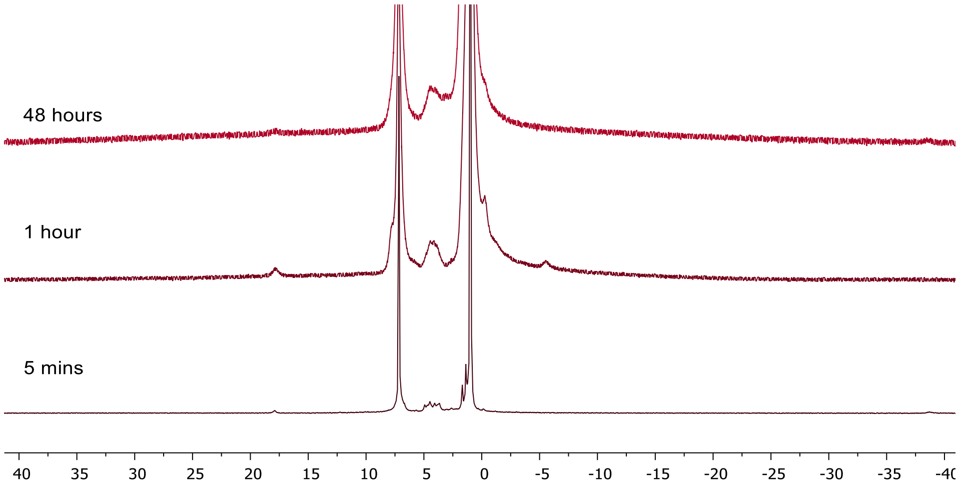

Figure S23. ^{1}H NMR stacked spectrum of **2a′** with HBpin (C_6D_6, 400 MHz).

7.1.7. **2b′**

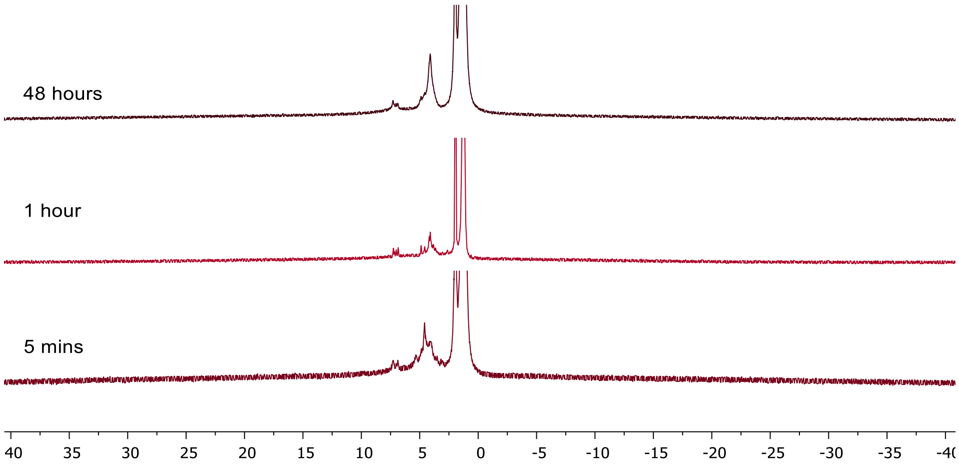

Figure S24. ^{1}H NMR stacked spectrum of **2b′** with HBpin (CD_3CN, 400 MHz).

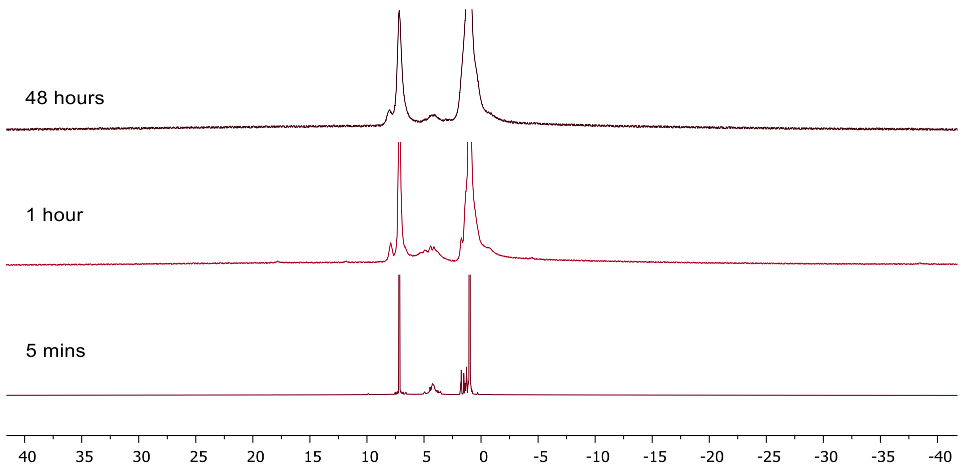

Figure S25. ^{1}H NMR stacked spectrum of **2b′** with HBpin (C_6D_6, 400 MHz).

7.1.8. **2c′**

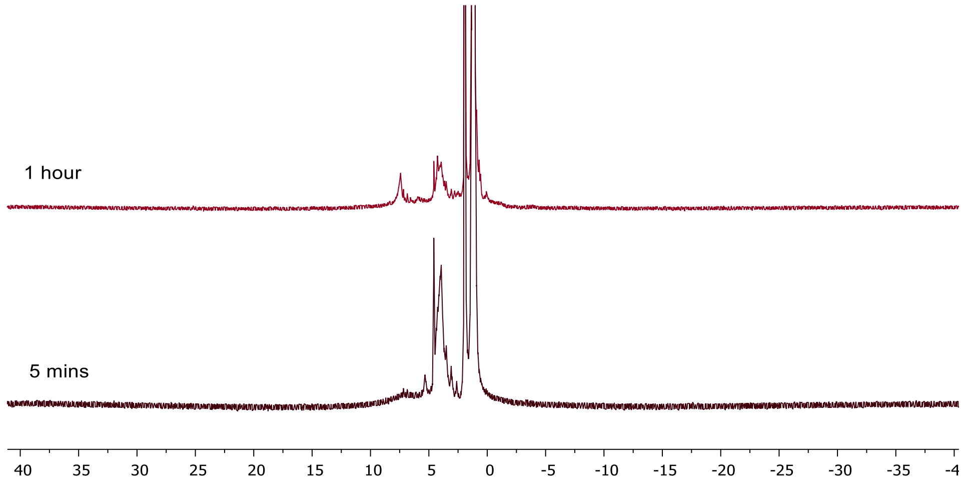

Figure S26. ^{1}H NMR stacked spectrum of **2c′** with HBpin (CD_3CN, 400 MHz).

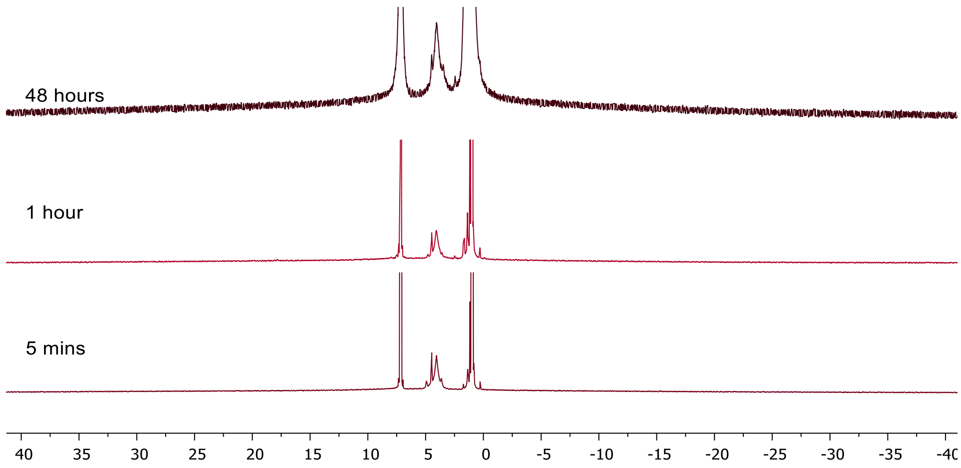

Figure S27. ^{1}H NMR stacked spectrum of **2c′** with HBpin (C_6D_6, 400 MHz).

7.1.9. **2d'**

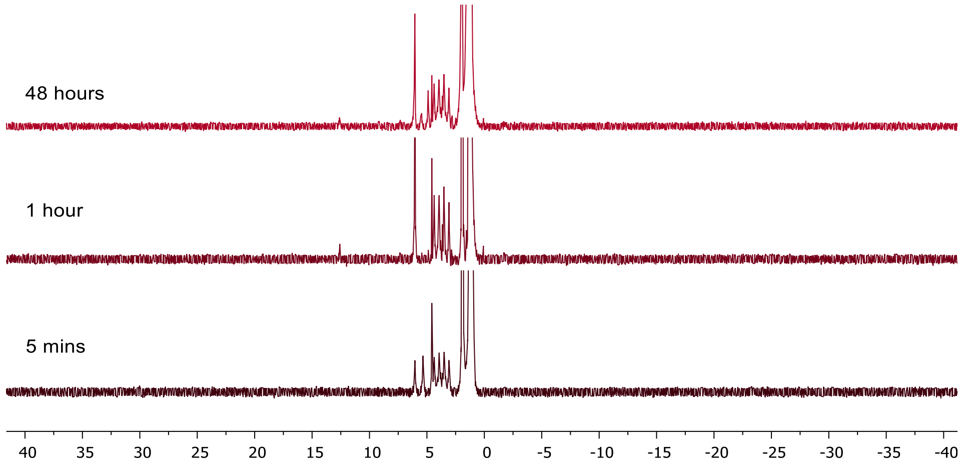

Figure S28. ^{1}H NMR stacked spectrum of **2d'** with HBpin (CD_3CN, 400 MHz).

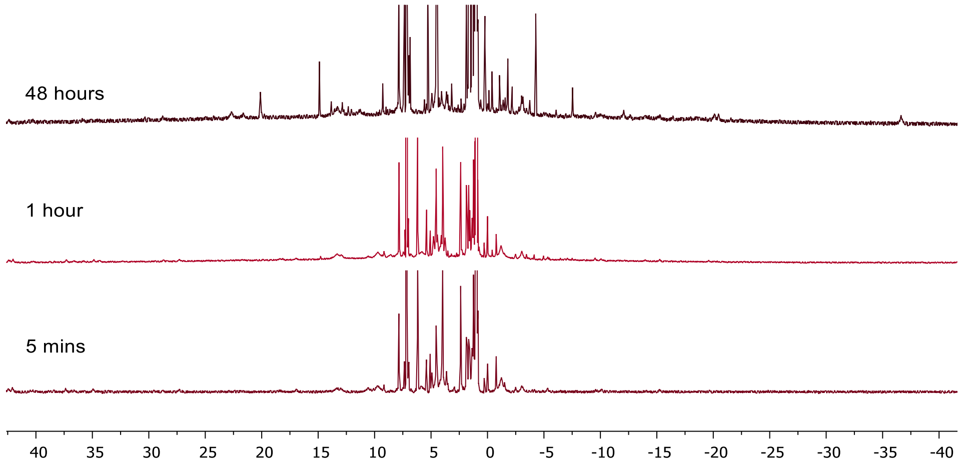

Figure S29. ^{1}H NMR stacked spectrum of **2d'** with HBpin (C_6D_6, 400 MHz).

7.1.10. **5**

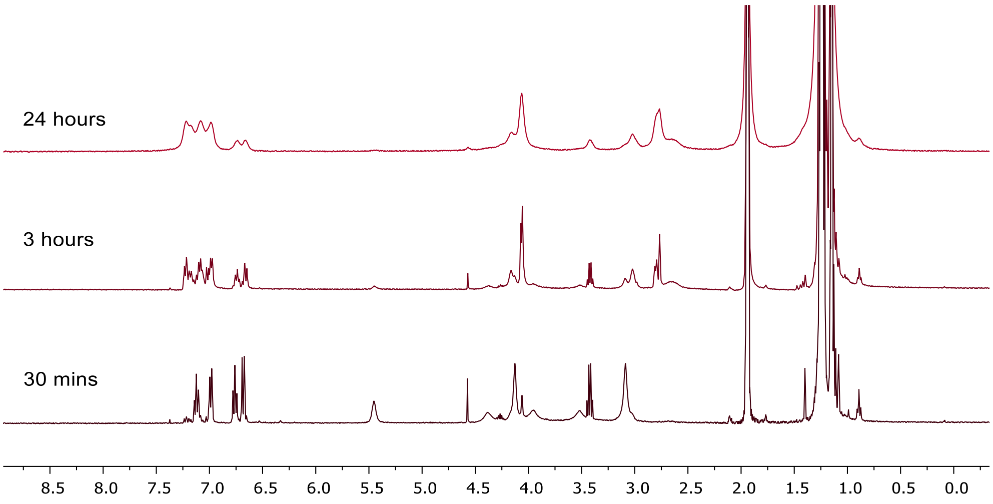

Figure S30. ^{1}H NMR stacked spectrum of **5** with HBpin (CD_3CN, 400 MHz).

7.2. Varying borane stoichiometry

7.2.1. Precatalyst **1e** with varying equivalents of HBpin

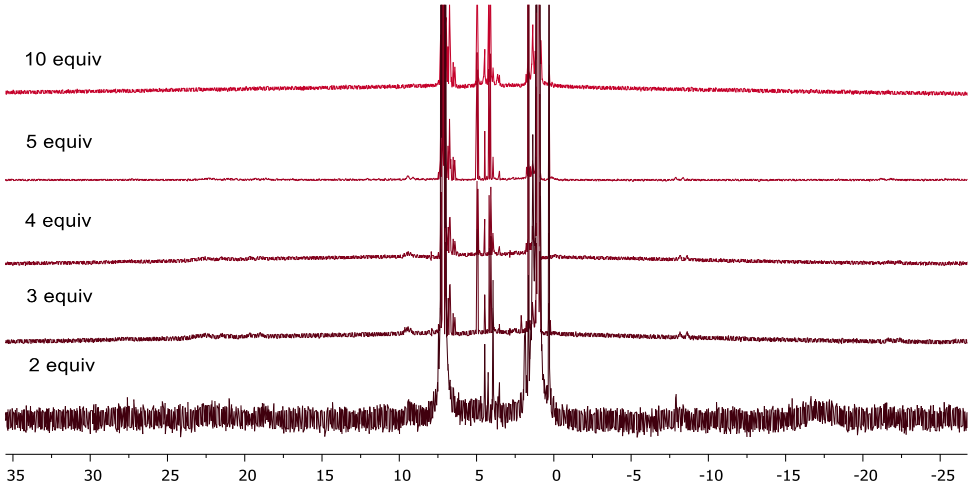

Figure S31. ^{1}H NMR stacked spectrum of **1e** treated with HBpin after 48 hours, indicating 4 equivalents of HBpin are required for full conversion to **3e**.

7.2.2. Precatalyst **1f** with varying equivalents of HBpin

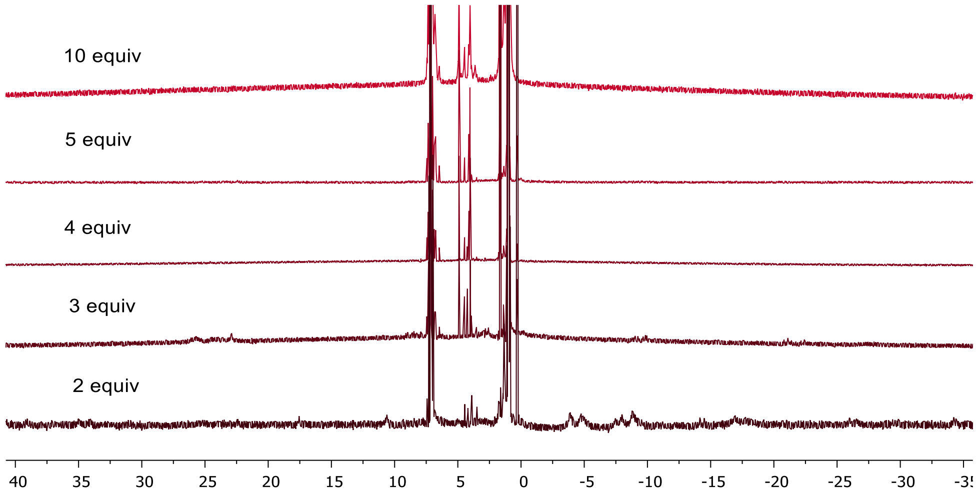

Figure S32. ^{1}H NMR stacked spectrum of **1f** treated with HBpin after 48 hours, indicating 4 equivalents of HBpin are required for full conversion to **3f**.

7.3. Alternative boranes

7.3.1. **2a** with HBCat

Precatalyst **2a** (10.0 mg, 15 µmol) was added to a J Young NMR tube and suspended in CD_3CN (600 µL). To the suspension of **2a**, HBcat (3.2 µL, 30 µmol) was added. The ^{1}H and ^{11}B{^{1}H} NMR spectra were recorded after 1 hour and 48 hours.

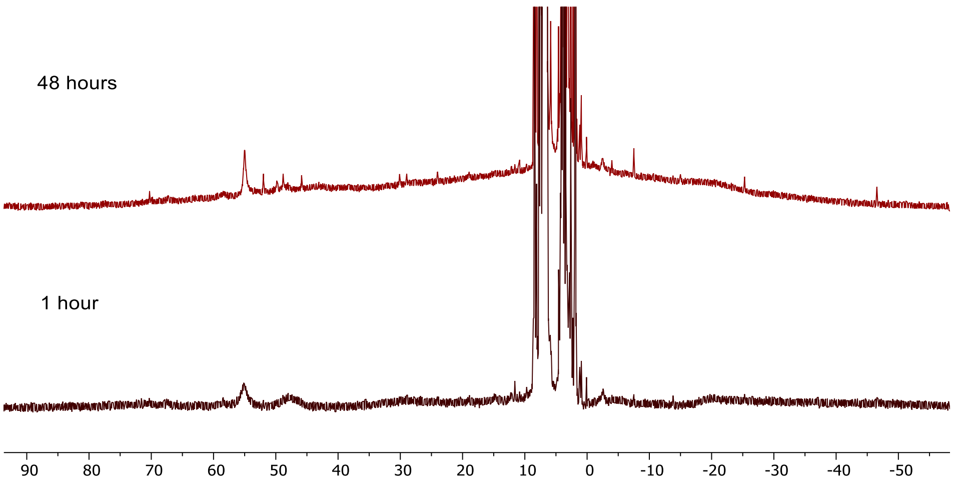

Figure S33. ^{1}H NMR stacked spectrum of **2a** with HBcat (CD_3CN, 400 MHz).

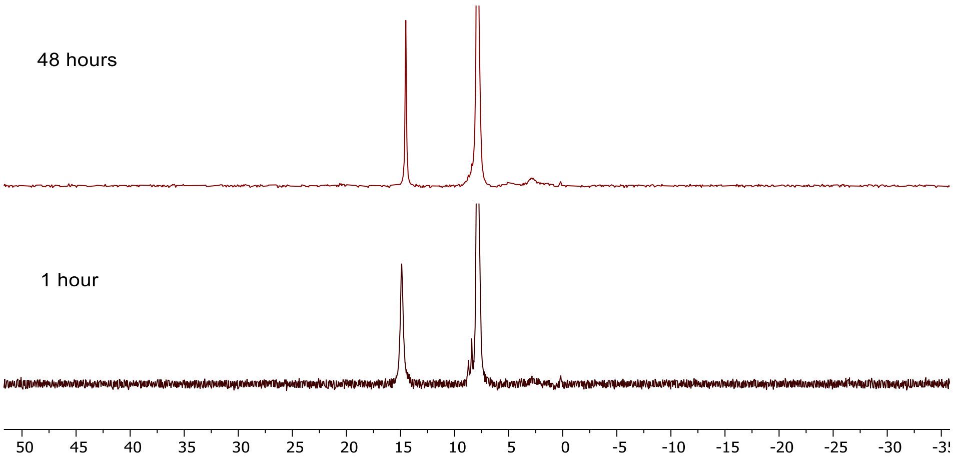

Figure S34. $^{11}B\{^{1}H\}$ NMR stacked spectrum of **2a** with HBcat (CD_3CN, 128 MHz).

7.3.2. **2a** with 9-BBN

Precatalyst **2a** (10.0 mg, 15 µmol) was added to a J Young NMR tube and suspended in CD_3CN (600 µL). To the suspension of **2a**, 9-BBN (3.8 mg, 15 µmol) was added. The ^{1}H and $^{11}B\{^{1}H\}$ NMR spectra were recorded after 1 hour and 48 hours.

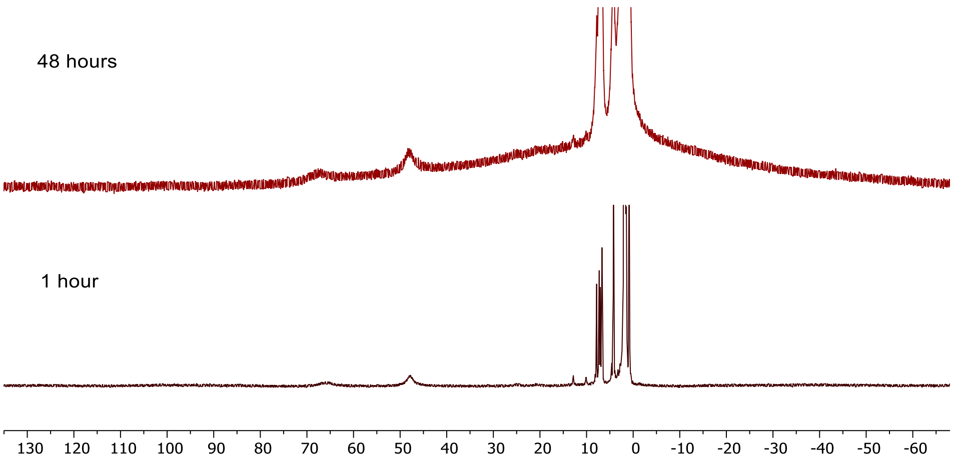

Figure S35. ^{1}H NMR stacked spectrum of **2a** with 9-BBN (CD_3CN, 400 MHz).

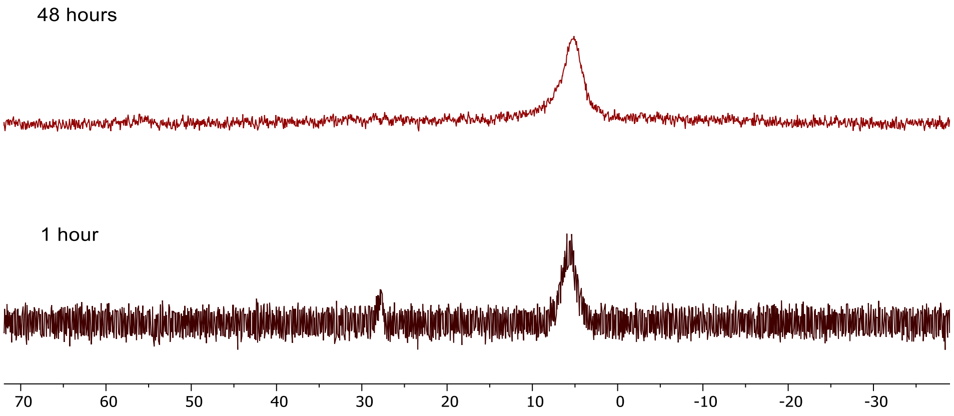

Figure S36. $^{11}B\{^{1}H\}$ NMR stacked spectrum of **2a** with HBcat (CD_3CN, 128 MHz).

7.3.3. **2a** with BH_3.SMe_2

Precatalyst **2a** (5.0 mg, 7.6 µmol) was added to a J Young NMR tube and suspended in CD_3CN (600 µL). To the suspension of **2a** BH_3.SMe_2 (3.6 µL, 38 µmol) was added. The ^{1}H and ^{11}B NMR spectra were recorded after 1 hour and 16 hours. After 48 hours Fe mirror was deposited, the reaction was filtered and ^{1}H and ^{11}B NMR spectrum were taken.

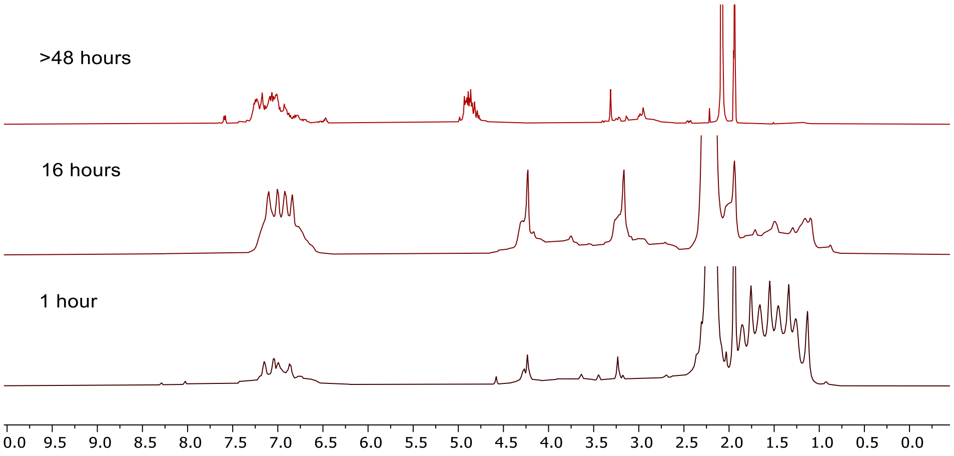

Figure S37. ^{1}H NMR stacked spectrum of **2a** with $BH_3.SMe_2$ (CD_3CN, 500 MHz).

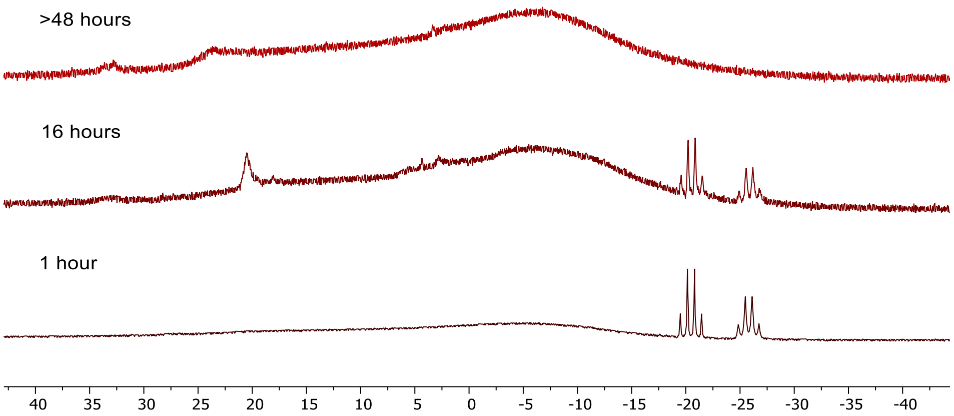

Figure S38. ^{11}B NMR stacked spectrum of **2a** with $BH_3.SMe_2$ (CD_3CN, 160 MHz).

7.3.4. **2e** with HBcat

Precatalyst **2e** (7.9 mg, 9.97 µmol) was added to a J Young NMR tube and suspended in C_6D_6 (500 µL). To the suspension of **2e**, HBcat (6.0 mg, 50.0 µmol). The ^{1}H and $^{11}B\{^{1}H\}$ and ^{19}F NMR spectra were recorded after 48 hours.

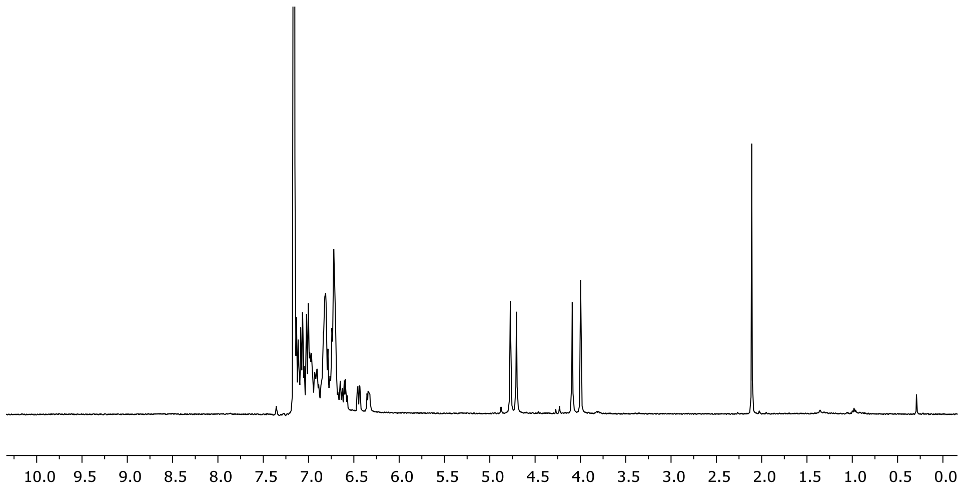

Figure S39. ^{1}H NMR spectrum of **2e** with HBcat (C_6D_6, 500 MHz).

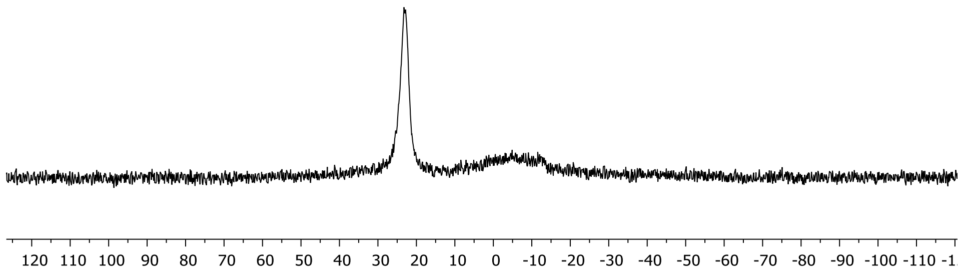

Figure S40. $^{11}B\{^{1}H\}$ NMR spectrum of **2e** with HBcat (C_6D_6, 160 MHz).

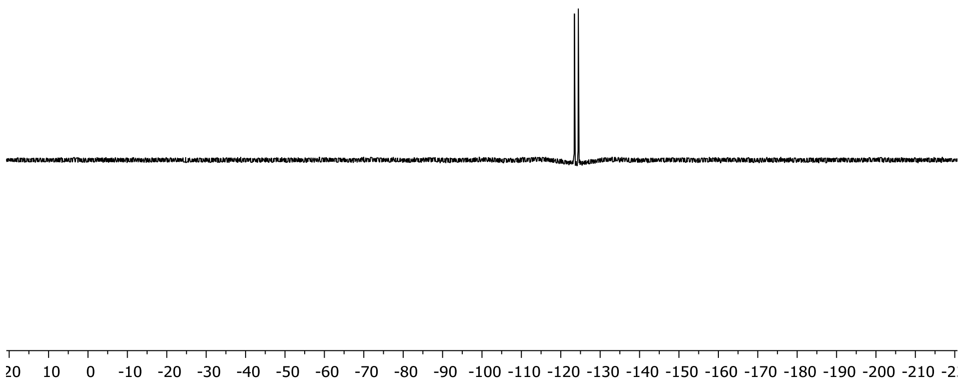

Figure S41. $^{19}F\{^{1}H\}$ NMR spectrum of **2e** with HBcat (C_6D_6, 470 MHz).

7.3.5. **2e** with 9-BBN

Precatalyst **2e** (7.9 mg, 9.97 µmol) was added to a J Young NMR tube and suspended in C_6D_6 (500 µL). To the suspension of **2e**, 9-BBN (6.1 mg, 25.0 µmol). The ^{1}H and $^{11}B\{^{1}H\}$ and ^{19}F NMR spectra were recorded after 48 hours.

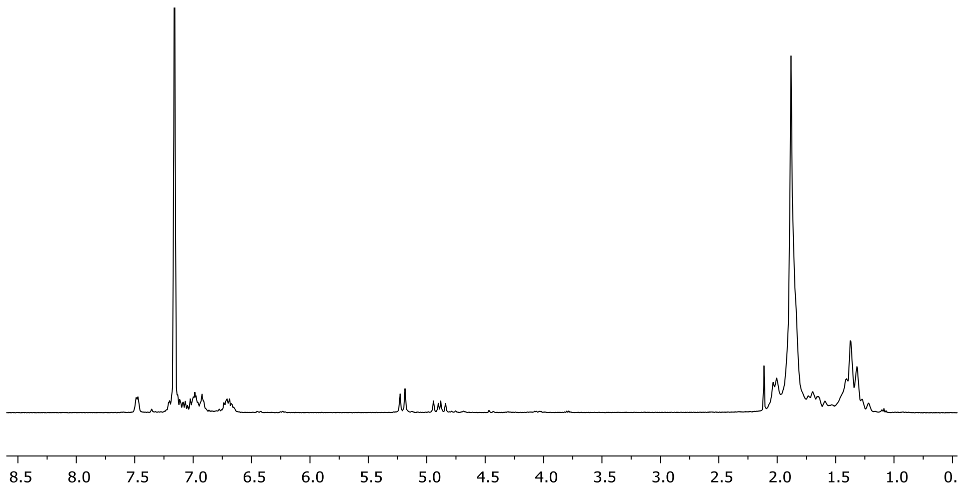

Figure S42. ^{1}H NMR spectrum of **2e** with 9-BBN (C_6D_6, 500 MHz).

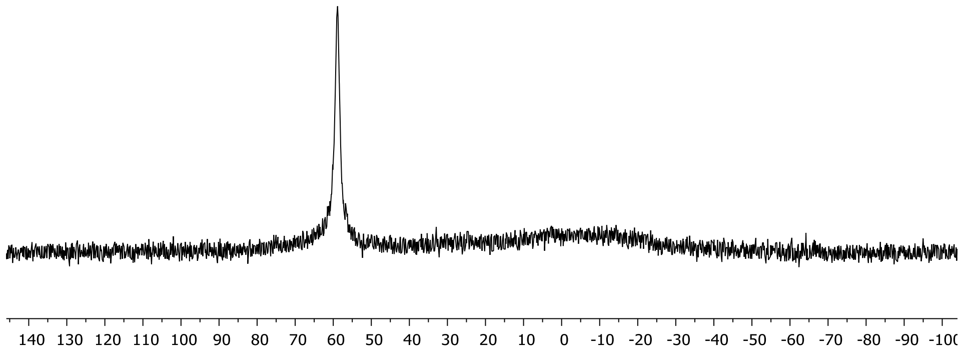

Figure S43. ^{11}B{^{1}H} NMR spectrum of **2e** with 9-BBN (C_6D_6, 160 MHz).

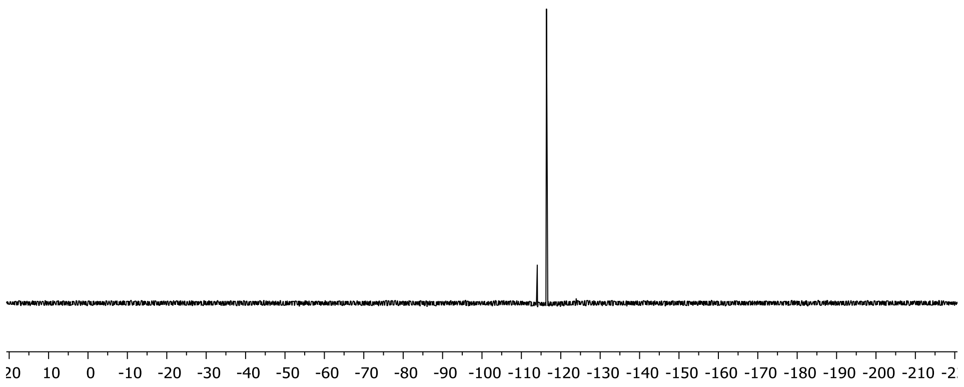

Figure S44. ^{19}F{^{1}H} NMR spectrum of **2e** with 9-BBN (C_6D_6, 470 MHz).

7.3.6. **2f** with HBcat

Precatalyst **2f** (8.9 mg, 9.97 µmol) was added to a J Young NMR tube and suspended in C_6D_6 (500 µL). To the suspension of **2f**, HBcat (6.0 mg, 50.0 µmol). The ^1H and $^{11}B\{^1H\}$ and ^{19}F NMR spectra were recorded after 48 hours.

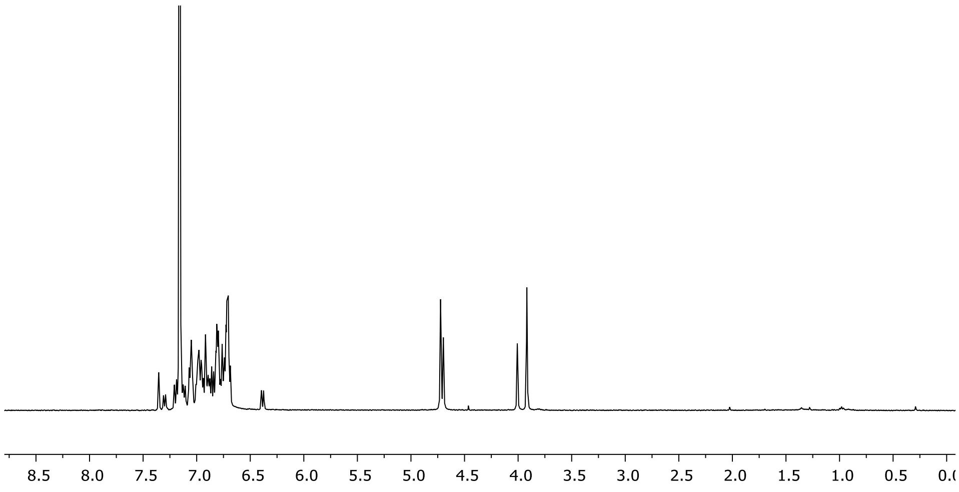

Figure S45. ^1H NMR spectrum of **2f** with HBcat (C_6D_6, 500 MHz).

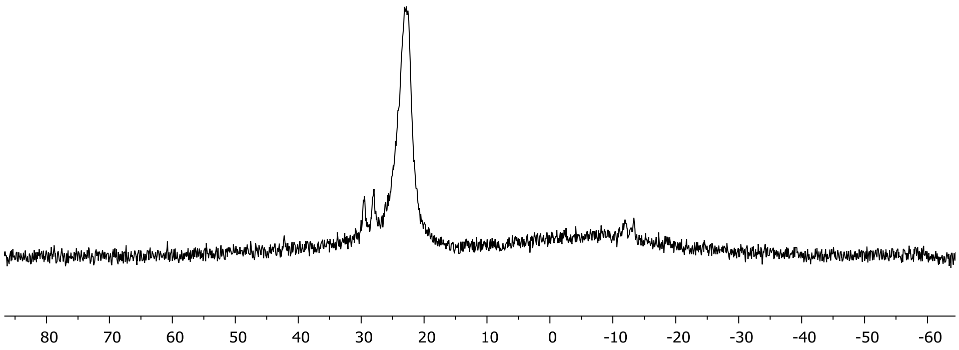

Figure S46. $^{11}B\{^1H\}$ NMR spectrum of **2f** with HBcat (C_6D_6, 160 MHz).

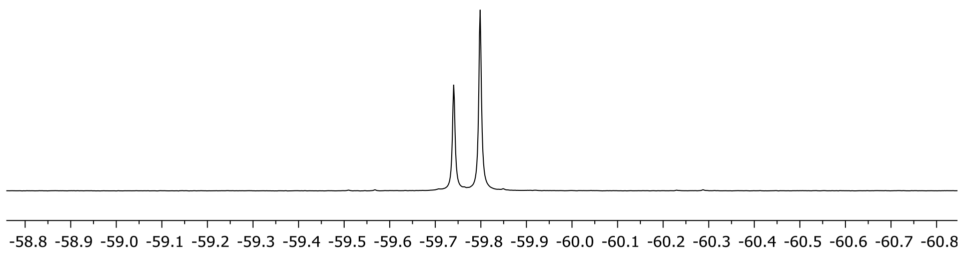

Figure S47. $^{19}F\{^1H\}$ NMR spectrum of **2f** with HBcat (C_6D_6, 470 MHz).

7.3.7. **2f** with 9-BBN

Precatalyst **2f** (8.9 mg, 9.97 µmol) was added to a J Young NMR tube and suspended in C_6D_6 (500 µL). To the suspension of **2f**, 9-BBN (6.1 mg, 25.0 µmol). The ^1H and $^{11}B\{^1H\}$ and ^{19}F NMR spectra were recorded after 48 hours.

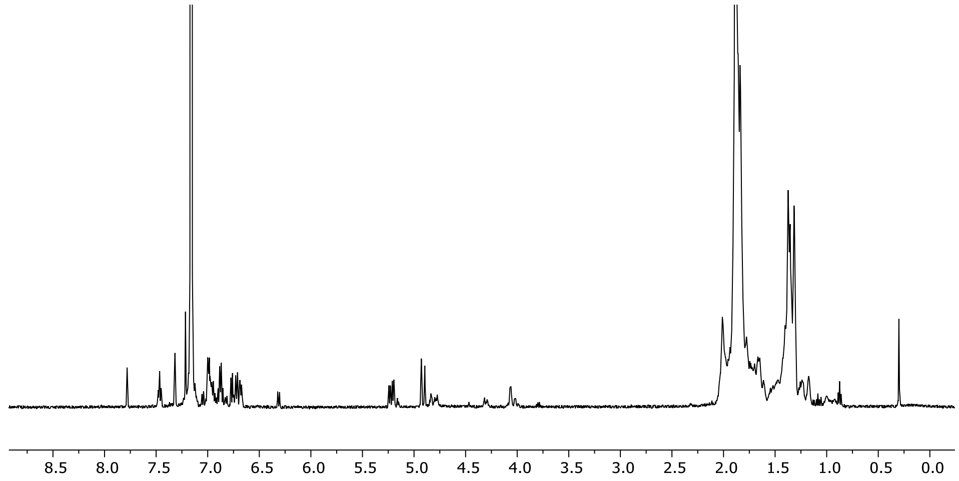

Figure S48. ^1H NMR spectrum of **2f** with 9-BBN (C_6D_6, 500 MHz).

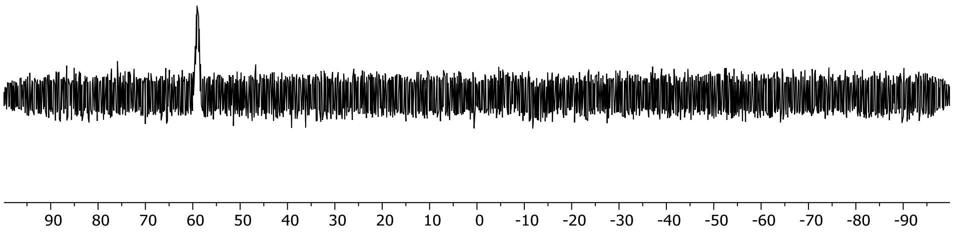

Figure S49. $^{11}B\{^1H\}$ NMR spectrum of **2f** with 9-BBN (C_6D_6, 160 MHz).

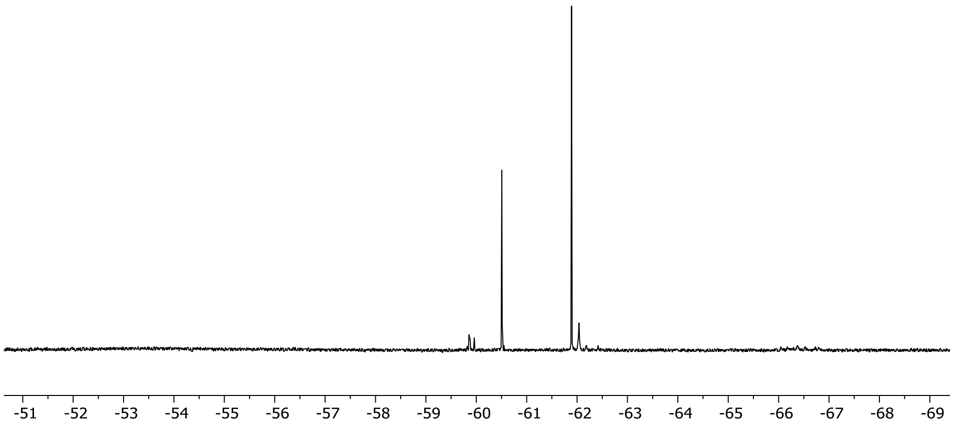

Figure S50. $^{19}F\{^1H\}$ NMR spectrum of **2f** with 9-BBN (C_6D_6, 470 MHz).

7.3.8. **2f** with $BH_3.SMe_2$

Precatalyst **2f** (6.8 mg, 7.6 µmol) was added to a J Young NMR tube and suspended in C_6D_6 (600 µL). To the suspension of **2f**, $BH_3.SMe_2$ (3.6 µL, 38 µmol) was added. The ^1H, ^{11}B and ^{19}F NMR spectra were recorded after 1 hour and 24 hours. After 48 hours Fe mirror was deposited, the reaction was filtered and ^1H spectrum was taken.

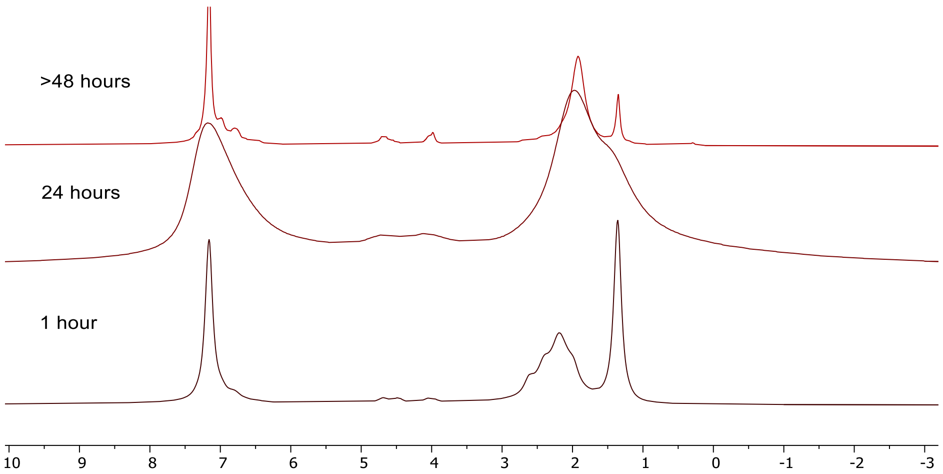

Figure S51. ^{1}H NMR stacked spectrum of **2f** with $BH_3.SMe_2$ (C_6D_6, 500 MHz).

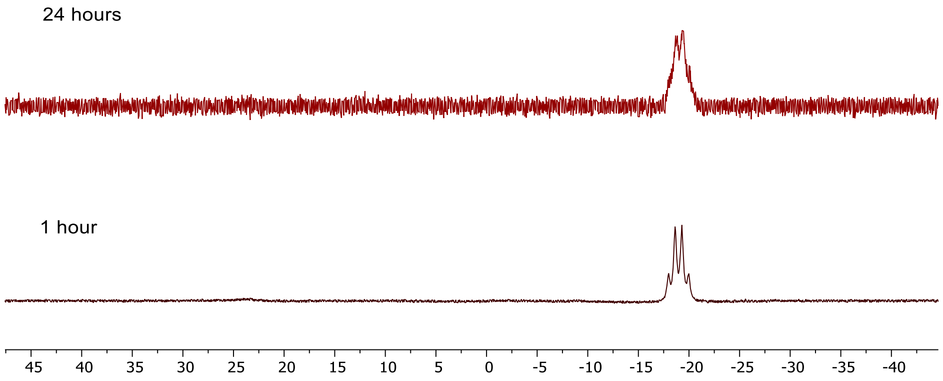

Figure S52. ^{11}B NMR stacked spectrum of **2f** with $BH_3.SMe_2$ (C_6D_6, 160 MHz).

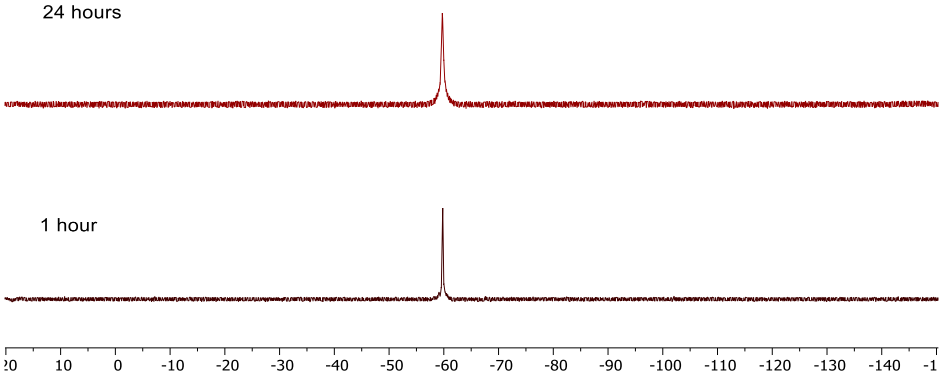

Figure S53. ^{19}F NMR stacked spectrum of **2f** with $BH_3.SMe_2$ (C_6D_6, 470 MHz).

7.4. Reaction with DBpin

7.4.1. Synthesis of neat DBpin

DBpin was synthesized by a modified literature procedure.[22] B_2pin_2 (1.00 g, 3.94 mmol) and Raney Nickel (5 mol%) were placed under an atmosphere of D_2 gas and the solids were agitated at 80 °C for 16 h. The resulting suspension was allowed to cool to room temperature and the flask was backfilled with D_2 again. The suspension was stirred at 80 °C for another 16 h, and the neat DBpin was collected by filtration. Yield: 0.92 g (91%). **^{1}H NMR** (C_6D_6, 400 MHz): δ 0.99 (s, 12H). **^{11}B NMR** (C_6D_6, 128 MHz): δ 28.4 (t, $^1J_{BD}$ = 21). Data are consistent with the literature.[23]

7.4.2. Reaction of **2a** with DBpin

Precatalyst **2a** (6.6 mg, 10.0 µmol) was added to a J Young NMR tube and CD_3CN (500 µL) and to this suspension pinacolborane (7.26 µL, 50.0 µmol) was added. The ^{1}H and ^{11}B{^{1}H} NMR spectra were recorded after 2 hours.

7.4.3. Reaction of **5** with DBpin

Precatalyst **5** (6.7 mg, 10.0 µmol) was added to a J Young NMR tube and CD_3CN (500 µL) and to this suspension pinacolborane (7.26 µL, 50.0 µmol) was added. The ^{1}H and ^{11}B{^{1}H} NMR spectra were recorded after 2 hours.

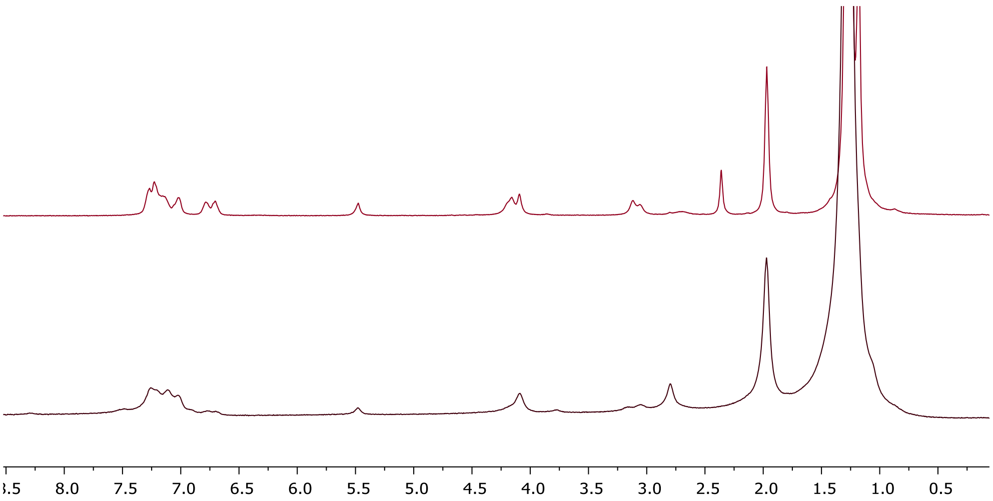

Figure S54. ^{1}H NMR of **2a** (bottom) and **5** (top) treated with HBpin (CD_3CN, 2h, RT).

7.5. Identification of **3**

As reported previously, the reaction of precatalyst **2a** with HBpin generates a diamagnetic complex as the major species in CD_3CN (**3a**) after 1 h at RT, *i.e.* after the induction period for **2a**. However, as shown above, in most cases the reaction of precatalysts **2** and HBpin results in broad paramagnetic signals in the ^{1}H NMR spectra that elude meaningful characterization. Moreover, we have demonstrated that diamagnetic species **3a** is inactive in catalysis. This likely indicates that on-cycle catalysis is dominated by paramagnetic intermediates. The only other precatalysts that form well-defined diamagnetic species upon treatment with HBpin in C_6D_6 are **2e** and **2f**, which generate **3e** and **3f** with BOB as the only unambiguously assigned by-product. The fact that species akin to **3a** *i.e.* **3e** and **3f** form in C_6D_6 (a solvent with does not facilitate catalysis is further evidence that **3a** is not the active species.

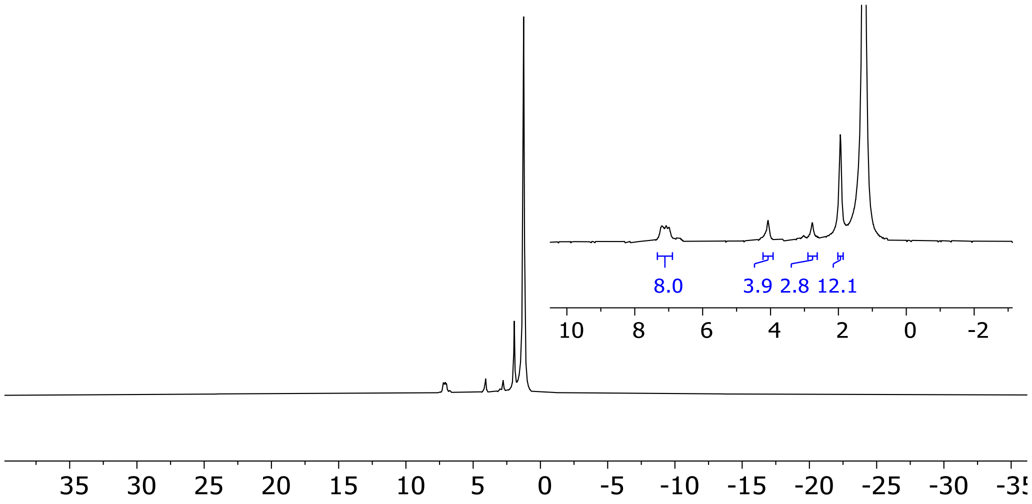

Figure S55. An example ^{1}H NMR spectrum showing diamagnetic species 3a (2a + 10 equiv. HBpin, 24h RT).

To gain further insight into the role of HBpin in catalysis, **2e** and **2f** were reacted in a stoichiometric fashion with HBcat and 9-BBN (in C_6D_6) after 72 h **3e-Bcat**, **3e-BBN**, **3f-Bcat** and **3f-BBN** were generated. The ^{1}H and $^{19}F\{^{1}H\}$ NMR spectra **3e-Bcat**, **3e-BBN**, **3f-Bcat** and **3f-BBN** bear a striking resemblance to that of diamagnetic species **3e** and **3f** (*vide infra*). It is again worth noting that cyclotrimerization catalysis is not mediated by HBcat or 9-BBN, further implying HBpin incorporation into the active species.

These *in situ* generated diamagnetic species (**3-Bcat/BBN**) have very simple ^{1}H NMR spectra, although contamination with $O(B(OR)_2)$ makes species identification difficult (only ligand-based signals are assignable). However, in all cases no diagnostic imine protons could be assigned, rather an additional methylene resonance is observed in the ^{1}H NMR spectra (~ δ 4 – 5 ppm). This observation indicates that the imine moieties in the salen framework are reduced over the course of the stoichiometric reaction to form an [Fe(II)(salan)] species. The diamagnetic complexes **3** were characterized by ^{1}H, $^{13}C\{^{1}H\}$ ^{1}H-^{1}H COSY, ^{1}H-^{13}C HSQC, ^{1}H-^{13}C HMBC, ^{1}H-^{11}B HMBC, and ^{19}F (where appropriate) NMR spectroscopy. These data suggest **3** corresponds to an Fe(II) species bearing a salan ligand in which the O-donor is bonded to the borane unit and the N-donor is formally an anionic donor.

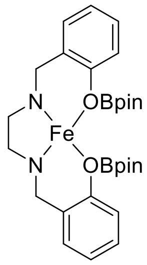

Figure S56. The speculated speciation of complex **3a**.

The independent synthesis of **3a** was attempted by treatment of ^{H}salan with 2 equivalents of HBpin followed by reaction with $Fe(OAc)_2$. However the resulting dehydrocoupled product formed from ^{H}salan and HBpin was found to be too insoluble to undergo any further reactivity with $Fe(OAc)_2$. The extensive NMR data collected for complexes **3a**, **3e** and **3f** are consistent with speciation as per **Figure S56**, though they cannot be assigned unambiguously, at present.

8. Detection and characterisation of catalytically relevant species

8.1. Stoichiometric LIFDI-MS reaction monitoring

CH_3CN is not compatible with LIFDI-MS, therefore the stoichiometric reaction **2a** (13.3 mg, 20.0 µmol) and HBpin (14.5 µL, 0.10 mmol) was conducted in PhCN and THF (500 µL) at ambient temperature in an inert atmosphere glove box. Aliquots were taken periodically, and the LIFDI-MS recorded.

8.1.1. Stoichiometric reaction in PhCN

i. 20 mins:

Reduction of the backbone was observed after 20 minutes by analysing the molecular ion fragment [Fe(salen)] (322 *m/z*). Further analysis of the isotope pattern shows a 50:40:10 ratio of salen:salalen:salan ligands.

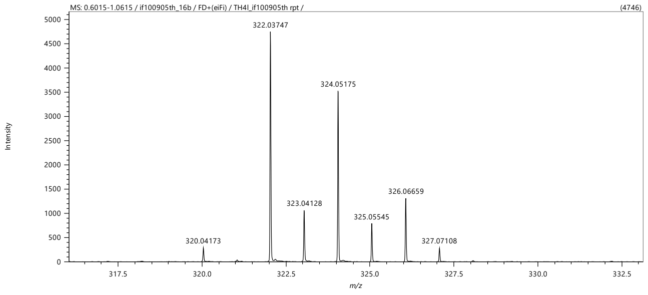

Figure S57. LIFDI-MS [Fe(salen)] region indicating backbone reduction.

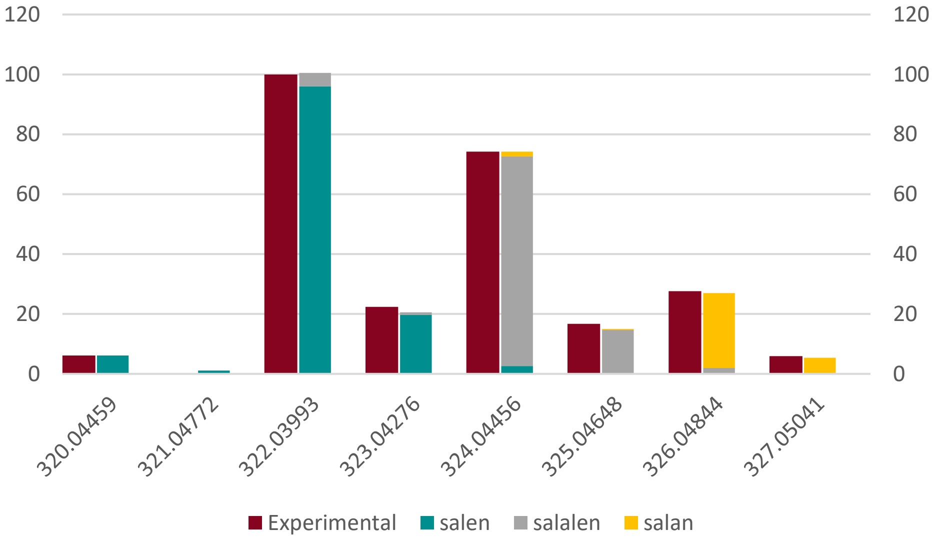

Figure S58. Ratios of salen:salalen:salan ligands.

ii. 1 hour:

[Fe(salen)(μ-H)(μ-H)Fe(salan)] (*m/z* = 650) and [Fe(salen)(μ-H)(μ-Bpin)Fe(salan)] (**6a**, *m/z* = 776) detected, indicating that reduction and boryl generation are consistent with the induction period timeframe.

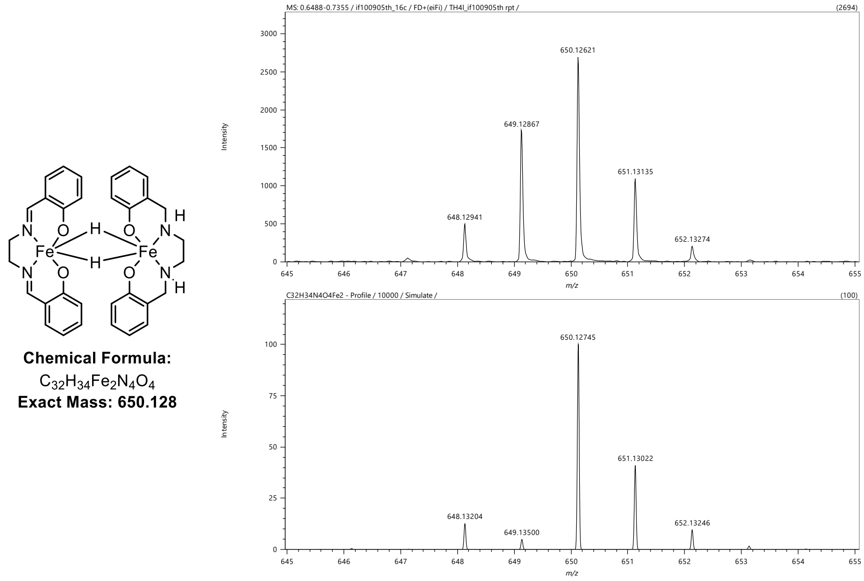

Figure S59. LIFDI-MS of [Fe(salen)(μ-H)(μ-H)Fe(salan)].

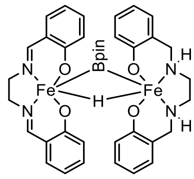

Chemical Formula: $C_{38}H_{45}BFe_2N_4O_6$
Exact Mass: 776.213

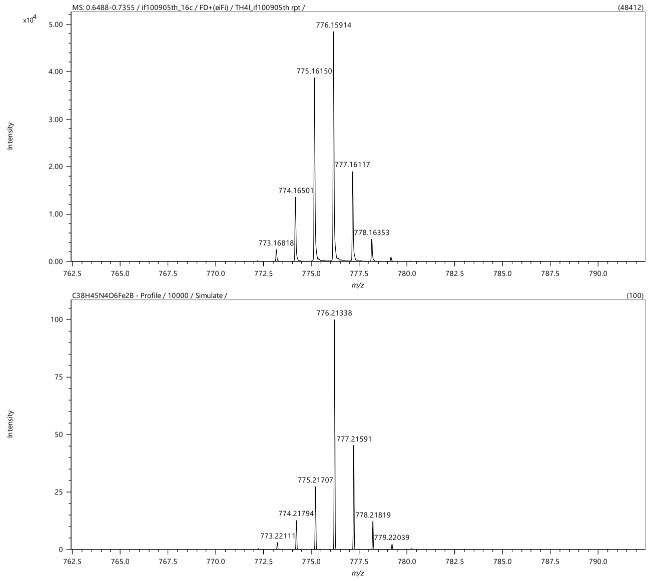

Figure S60. LIFDI-MS of **6a**.

iii. 48 hours

[Fe(salan)] molecular fragment is the dominant species, indicating significant backbone reduction:

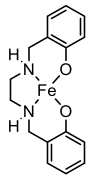

Chemical Formula: $C_{16}H_{18}FeN_2O_2$
Exact Mass: 326.072

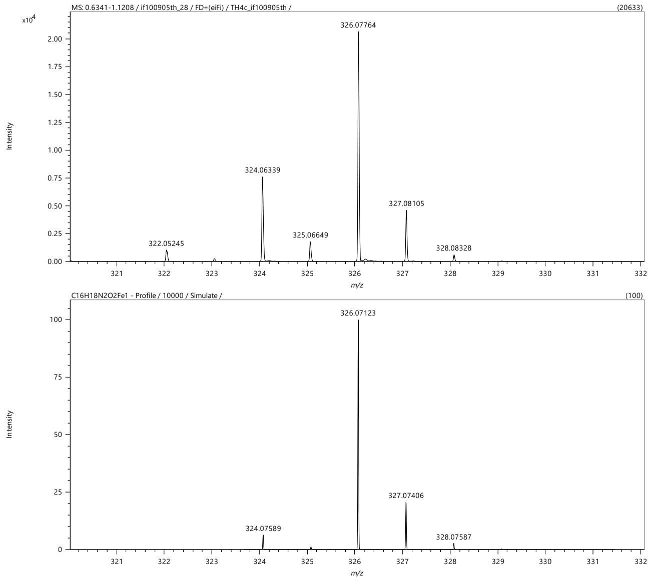

Figure S61. LIFDI-MS of [Fe(salan)] species.

8.1.2. Stoichiometric reaction of **2a** with HBpin in THF

i) *20 mins:*

[Fe(salen)(μ-H)(μ-H)Fe(salen)] (*m/z* = 646) and [Fe(salen)(μ-H)(μ-H)Fe(salan)] (*m/z* = 650) detected indicating facile Fe-H formation is concurrent with backbone reduction.

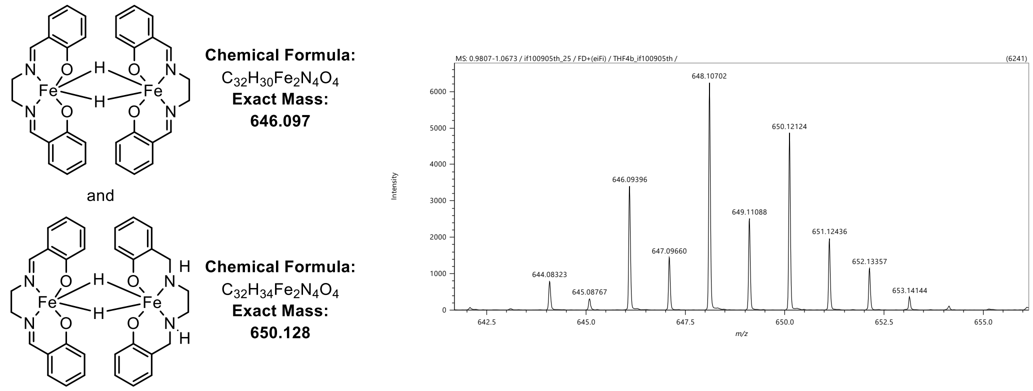

Figure S62. Selected LIFDI-MS of [Fe] species after 20 minutes.

ii) *1 hour:*

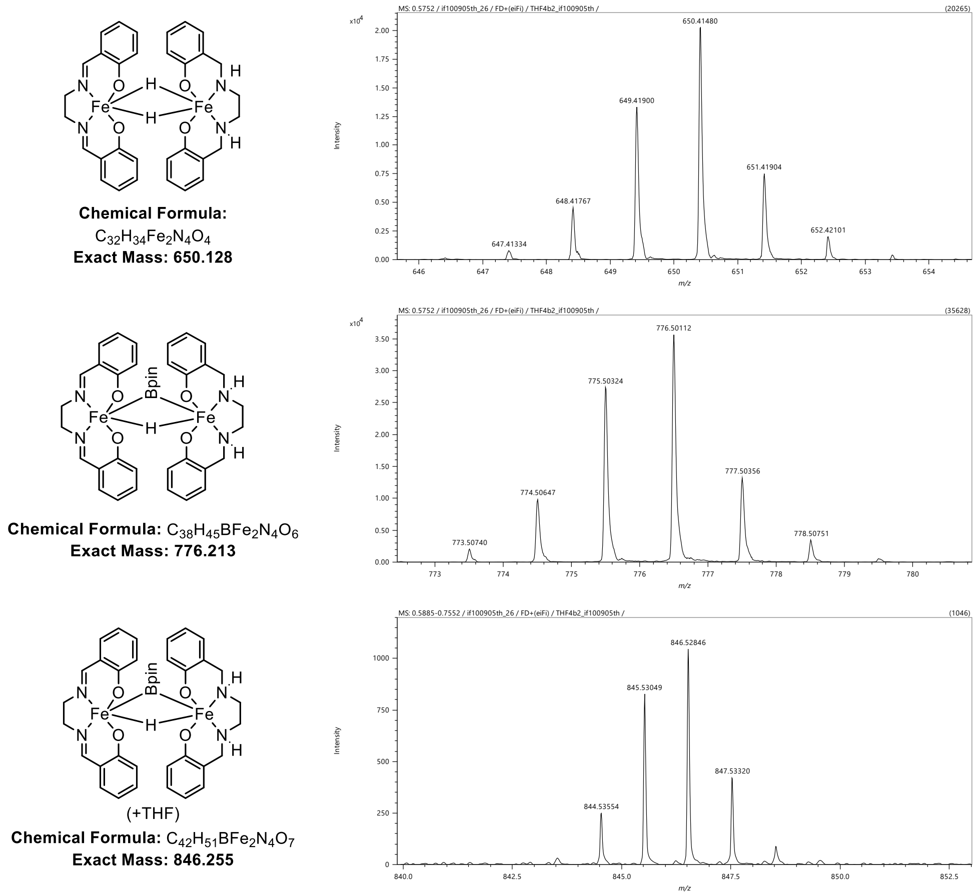

Figure S63. Selected LIFDI-MS of [Fe] species after 1 hour.

8.1.3. Stoichiometric reaction of **2a** with HBcat in THF

$O(Bcat)_2$ detected by LIFDI (m/z 254.0378). Reduction of backbone observed by LIFDI (m/z 322.0250 -> 326.1122 $[M]^+$). No Fe-H or Fe-boryl detected. Evidence for decomposition products (m/z 281.1280 and 508.1872) with partially and fully-reduced backbones. Also, evidence for BH_3 generation which mediates decomposition (*vide infra*).

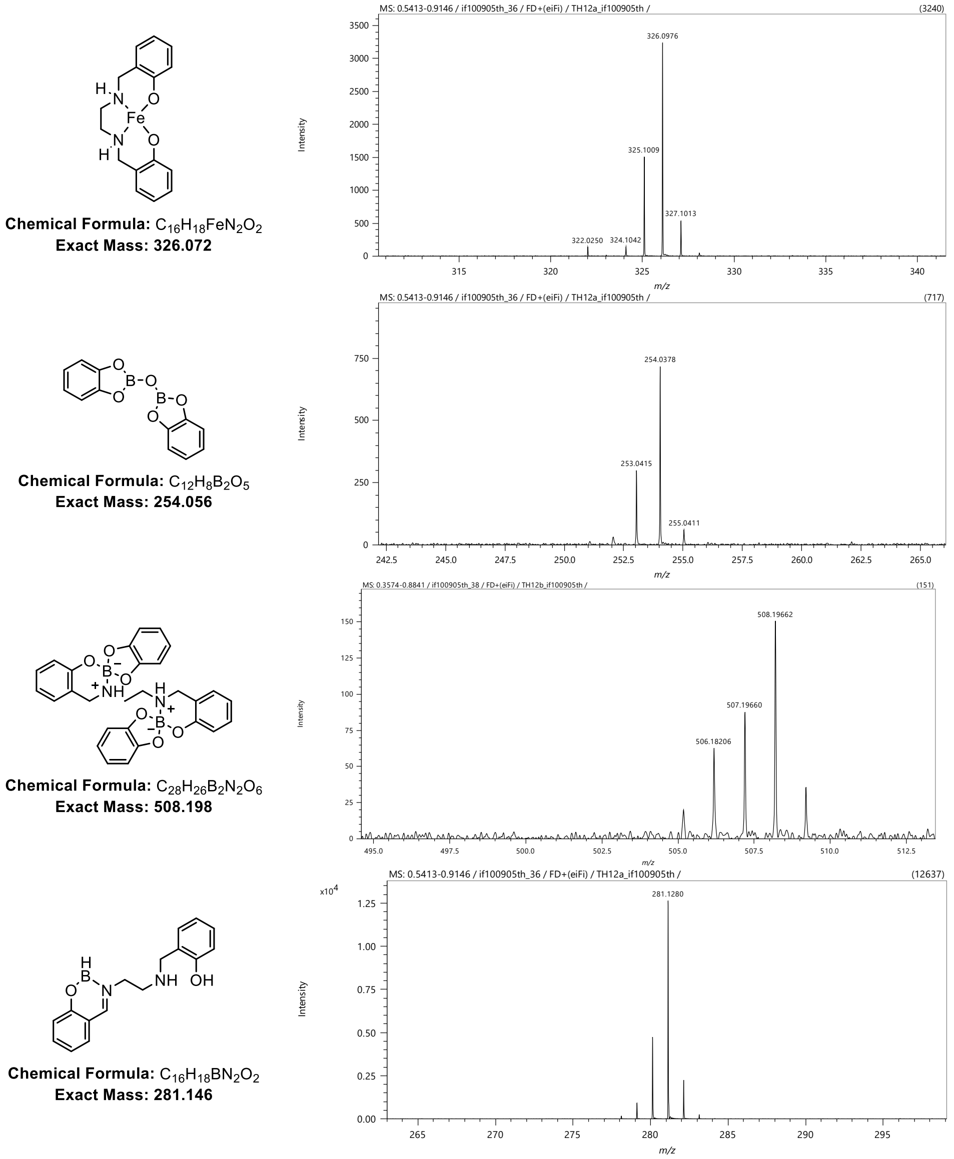

Figure S64. Selected LIFDI-MS of **2a** with HBCat after 1 hour (THF).

8.1.1. Stoichiometric reaction of **2a** with 9-BBN in THF

20 mins and 1 hour: Reduction of backbone observed by LIFDI (m/z 322.0351 -> 326.2587 [M]$^+$). Unknown Fe-containing species reducing on over the course of the timepoints (m/z 982.2437 -> m/z 988.1614) but does not correspond to an Fe-H or Fe boryl.

8.1.1. Stoichiometric reaction of **2f** with HBpin, HBcat or 9-BBN

In an inert atmosphere glovebox, solutions of **2f** (20 µmol) in toluene (500 µL) were treated with HBpin, HBcat or 9-BBN (0.1 mmol), the resulting suspensions were stirred at ambient temperature for ~ 48 hours and the LIFDI-MS recorded. No Fe-containing species could be identified, only a series of decomposition products could be tentatively assigned.

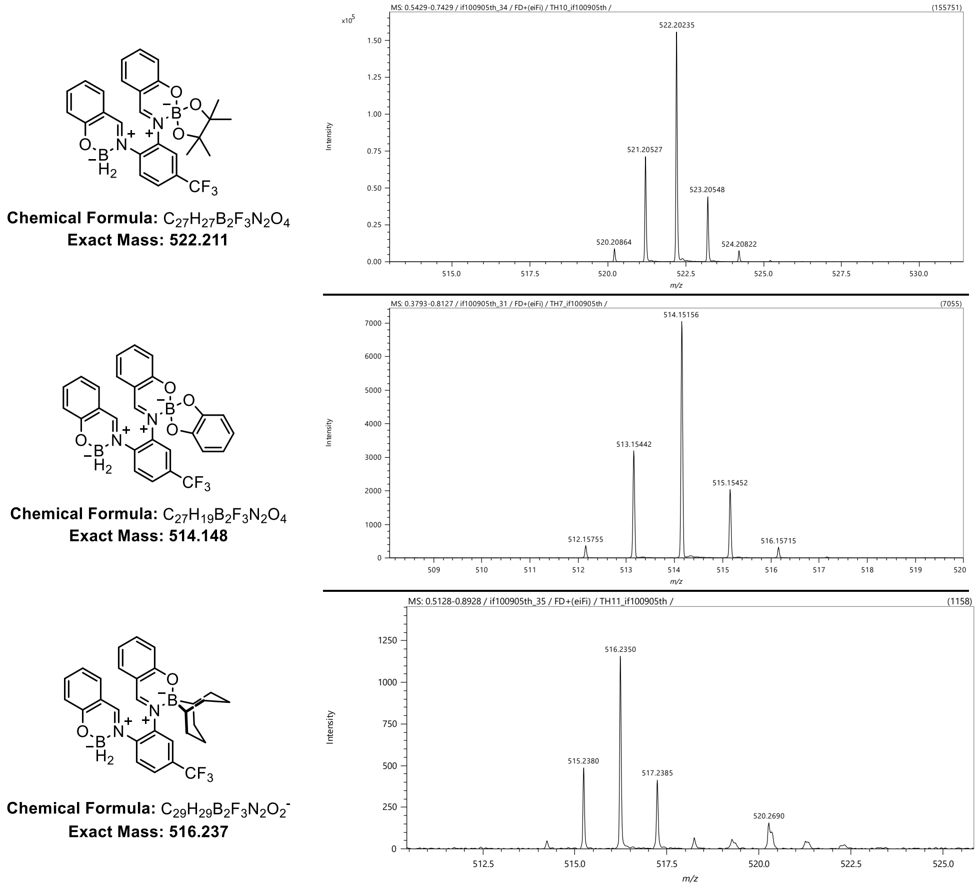

Figure S65. Selected LIFDI-MS data of **2f** with HBpin (top), HBcat (middle) and 9-BBN (bottom) (Toluene, 48 hours).

8.2. Catalytic monitoring with LIFDI-MS

8.2.1. Trimerisation of 4-tert-butylphenylacetylene

i. Backbone reduction by [Fe(salen)]:

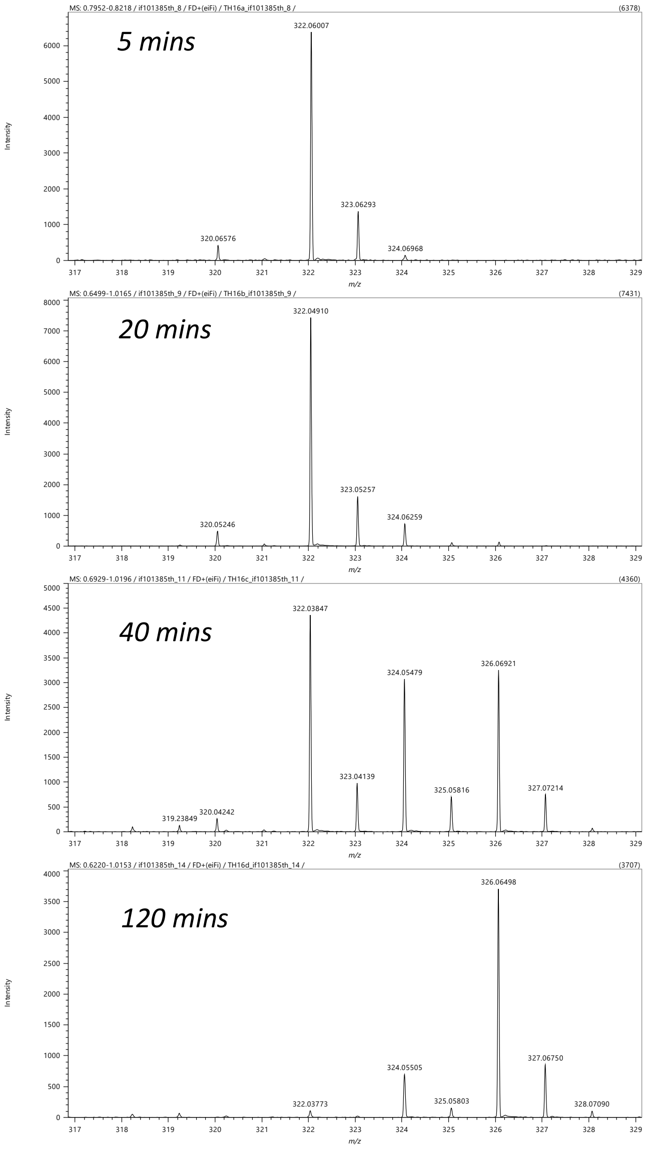

Figure S66. LIFDI-MS over timer demonstrating [Fe(salen)] reduction.

ii. Active species identification

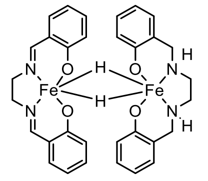

(+THF)
Chemical Formula:
$C_{36}H_{42}Fe_2N_4O_5$
Molecular Weight: 722.45

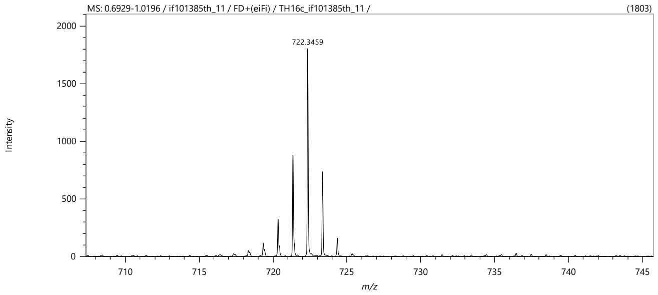

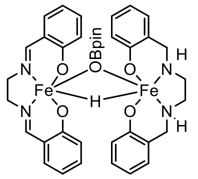

Chemical Formula: $C_{38}H_{43}BFe_2N_4O_7$
Exact Mass: 790.192
(±2H)

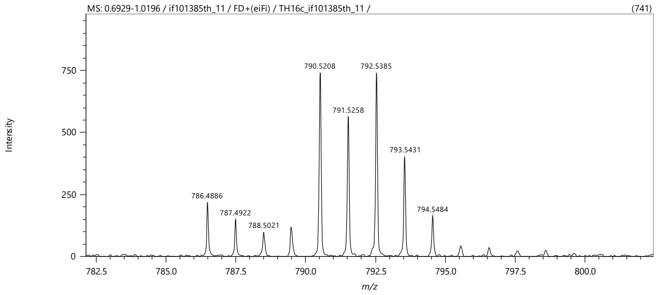

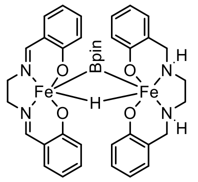

(+THF)
Chemical Formula: $C_{42}H_{53}BFe_2N_4O_7$
Exact Mass: 848.271

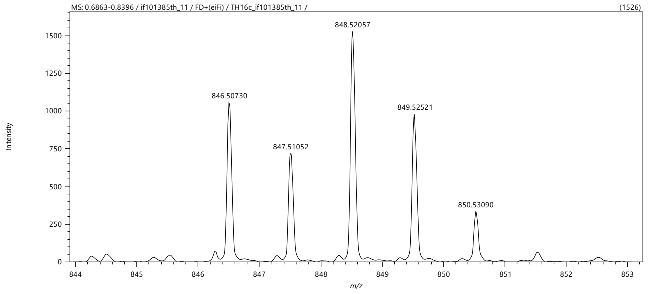

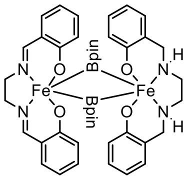

(+THF)
Chemical Formula: $C_{48}H_{64}B_2Fe_2N_4O_9$
Exact Mass: 974.356
(±2H, 4H)

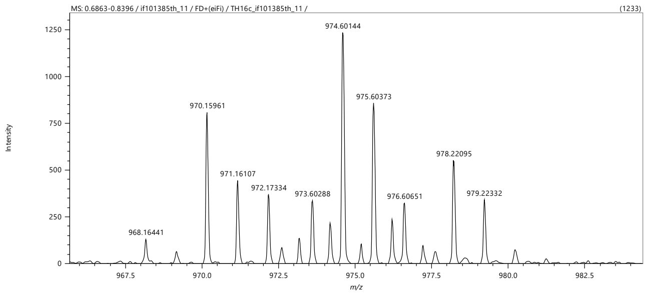

Figure S67. Selected LIFDI-MS identifying potential active species (t = 40 minutes).

Simulations:

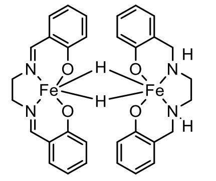

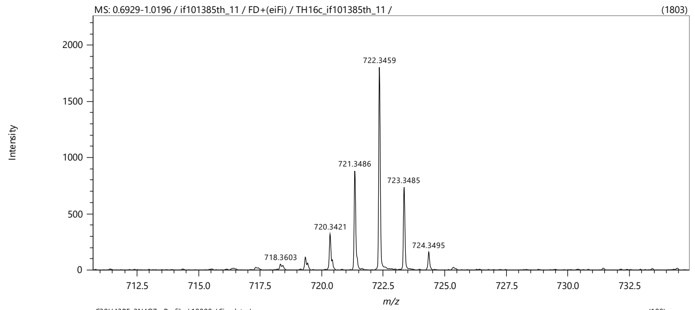

(+THF)
Chemical Formula: $C_{36}H_{42}Fe_2N_4O_5$
Molecular Weight: 722.45

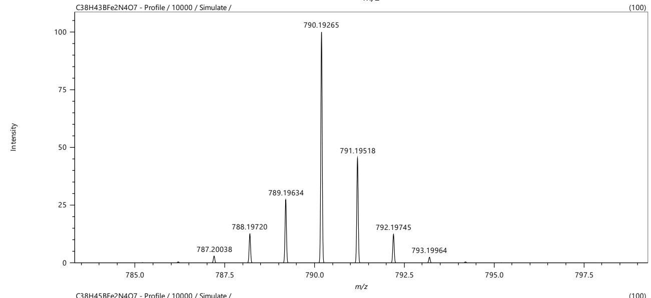

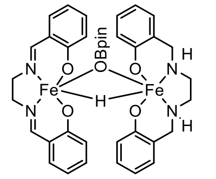

Chemical Formula: $C_{38}H_{43}BFe_2N_4O_7$
Exact Mass: 790.192

(±2H)

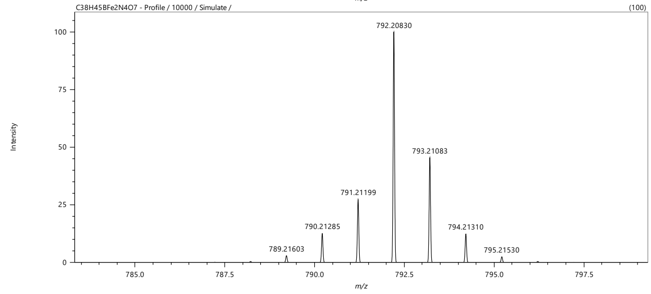

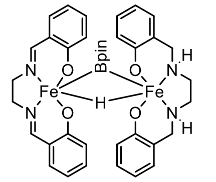

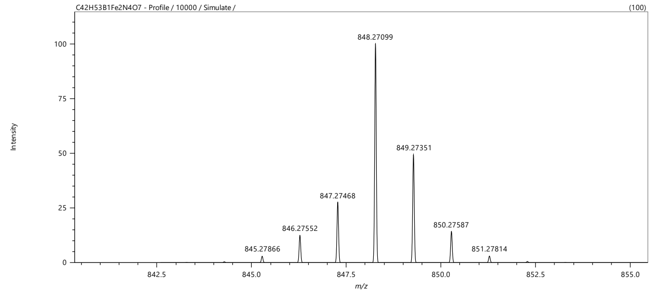

(+THF)
Chemical Formula: $C_{42}H_{53}BFe_2N_4O_7$
Exact Mass: 848.271

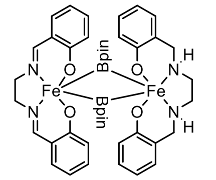

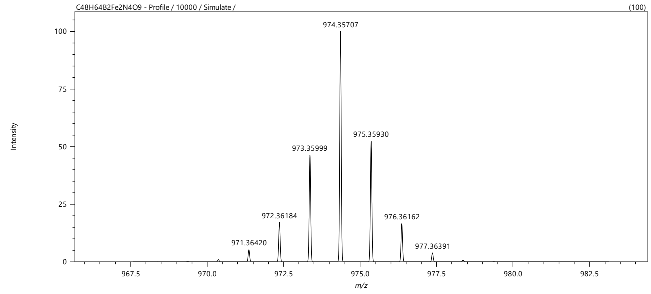

(+THF)
Chemical Formula: $C_{48}H_{64}B_2Fe_2N_4O_9$
Exact Mass: 974.356

Figure S68. Selected MS simulations for the Fe complexes identified by LIFDI-MS.

Alkyne insertion:

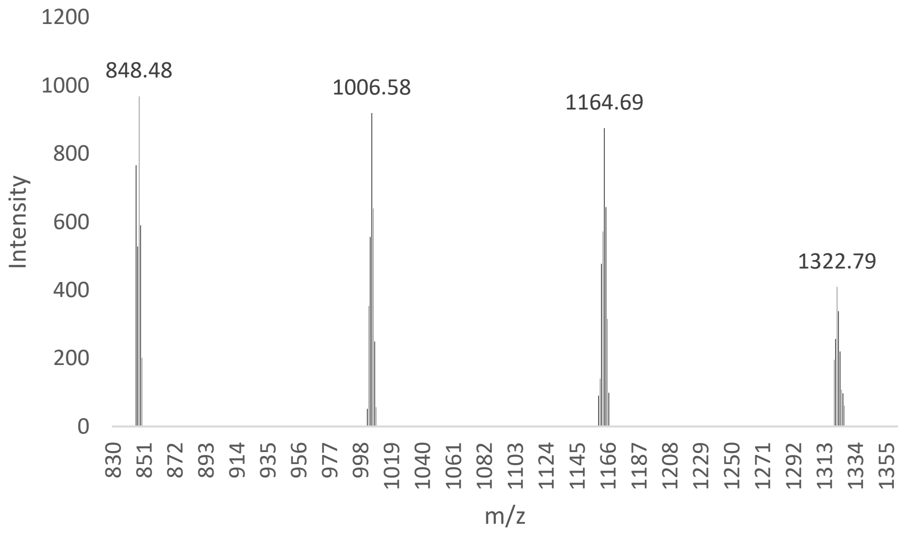

Figure S69. Selected *in situ* LIFDI-MS signals identified during the catalytic trimerization of 4-*tert*-butylphenylacetylene. Spacing of m/z 158 is consistent with additional units of alkyne (m/z 158.1). Data replotted from peak list tables for clarity.

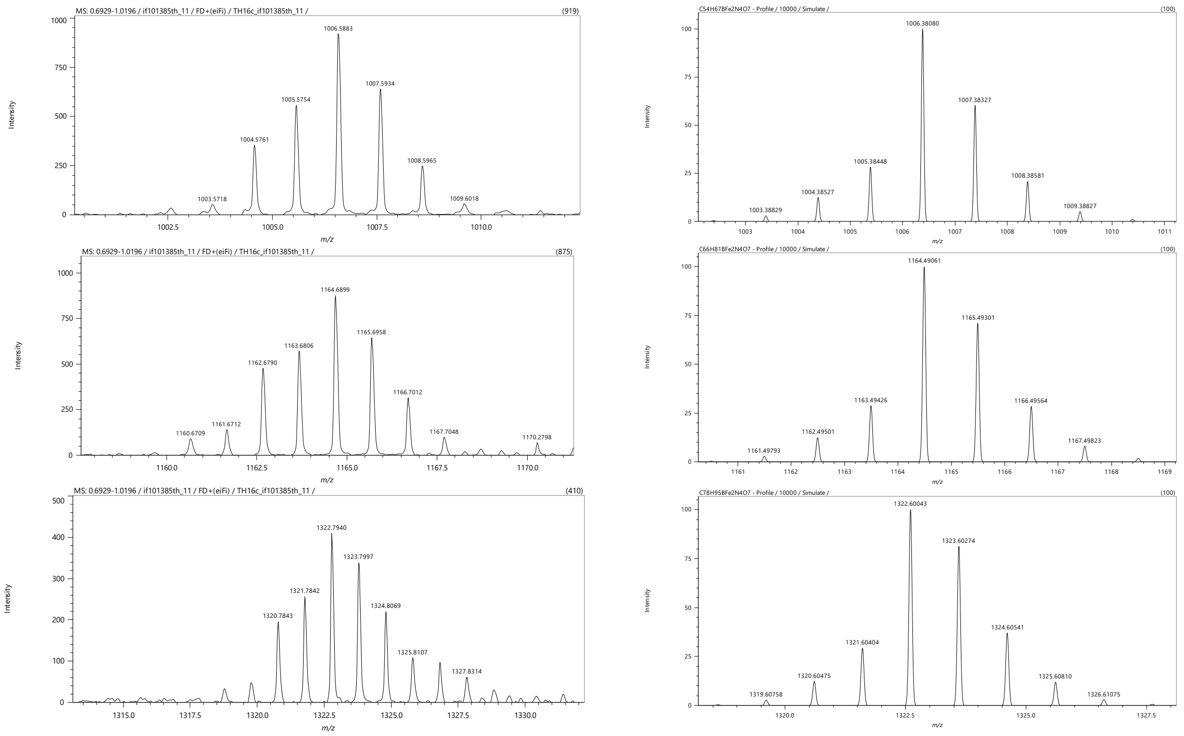

Figure S70. Selected LIFDI-MS highlighting alkyne insertion (left) and the corresponding MS simulations (right).

9. Attempts to generate 3a and [Fe(salan)Bpin] (8)

9.1. Attempt to generate **3**

^{H}Salan (2.7 mg, 10.0 µmol) was dissolved in C_6D_6 (500 µL) and treated with HBpin (2.9 µL, 20.0 µmol) resulting in the formation of a white precipitate and concomitant with the formation of H_2 bubbles. $Fe(OAc)_2$ (1.7 mg, 10.0 µmol) was added to the resulting suspension. No further productive reaction occurred up to 80 °C for several days.

9.2. Attempt to generate **3** or **8** from Fe(II)

^{H}Salan (2.7 mg, 10.0 µmol) and KBn (2.6, 20.0 µmol) were dissolved in CD_3CN or C_6D_6 (500 µL) resulting in the formation of a white precipitate which was subsequently reacted with $FeCl_2$ (1.3 mg, 10 µmol) to give an orange suspension. The orange suspension was treated separately with i) $O(Bpin)_2$, ii) HBpin, iii) B_2pin_2 and iv) 4-*tert*-butylphenylacetylene.

i) Reaction occurs but ^{1}H NMR is not consistent with **3a** – tentatively assigned as [Fe(^{H}salan)(BOB)].
ii) ^{1}H NMR consistent with the formation of **3a** as a mixture with unknown species.
iii) No reaction at RT, 50 °C or 80 °C.
iv) No trimerization occurs at RT, 50 °C or 80 °C.

9.3. Attempts to generate **8** from Fe(III)

9.3.1. Synthesis of [Fe(salan)Cl] (**7**)

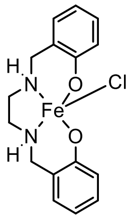

A solution of ^{H}salan (65.7 mg, 0.24 mmol) in ethanol (10 mL) was treated with $FeCl_3$ (39.1 mg, 0.24 mmol) and the resulting suspension was refluxed for 2 hours. The solution was cooled to ambient temperature and the volatiles removed in vacuo. The title compound was isolated as a purple microcrystalline solid following recrystallisation from DCM:pentane (1:20) at -30 °C. Yield: 82.7 mg (95%). Crystals suitable for single crystal X-ray crystallography were obtained by recrystallisation from DCM:pentane (1:20) at -30 °C.

^{1}H NMR (500 MHz; $CDCl_3$, 298 K): δ (all br.) 149.26, 133.16, 100.11, 90.05, 81.69, 73.95, 60.59, 49.37, 23.28, 21.83, 18.25, 17.79, 13.61, 12.38, −3.77, −5.76, −7.92, −8.55, −10.91.

LIFDI-MS: 361.0373, [*M*]$^{+}$ (calc. 361.0401) *m/z*.

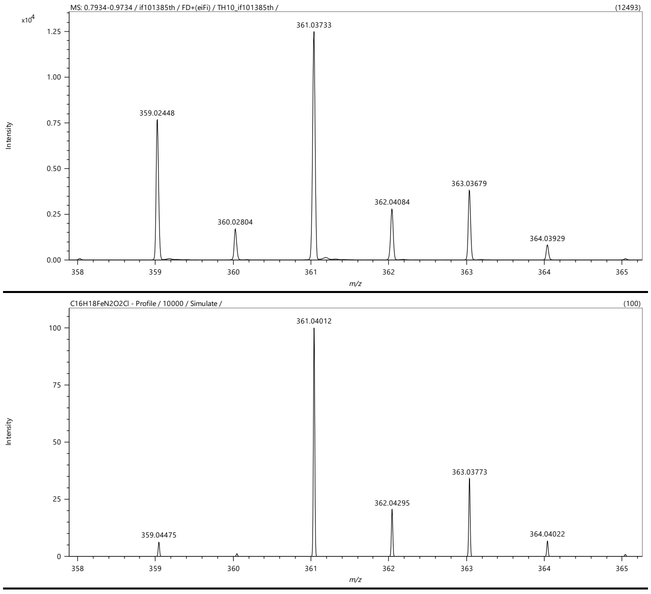

Figure S71. LIFDI-MS of **7** (top) and simulated isotope pattern (bottom).

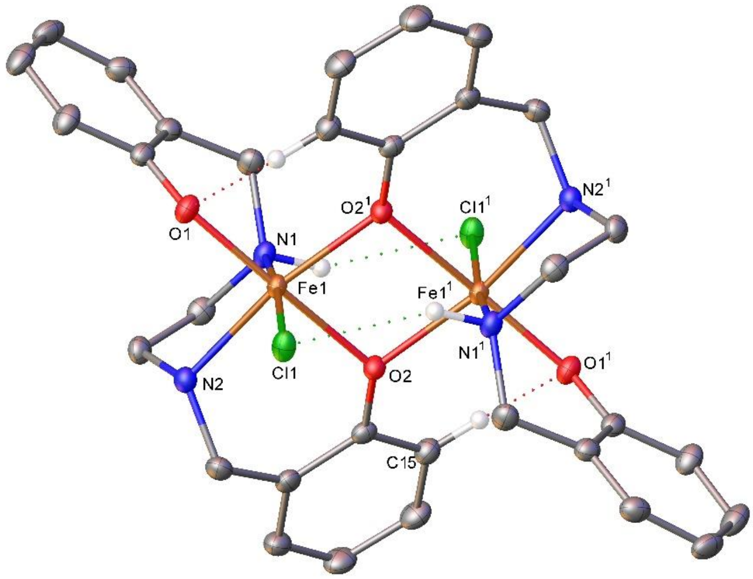

Figure S72. Molecular structure representation (50% ellipsoids) of compound **7**. Hydrogen atoms (H1 excepted) have been omitted for clarity. Symmetry operations: 1 1 − x, 1 − y, 1 − z.

9.3.2. Treatment of **7** with B_2pin_2 with KO^{t}Bu

A solution of **7** (3.60 mg, 9.96 µmol) in CD_3CN was treated with B_2pin_2 (2.50 mg, 9.84 µmol)) and KO*t*Bu (1.10 mg, 9.81 µmol). No reaction was observed up to 80 ˚C.

9.3.3. Treatment of **7** with nucleophilic Bpin (magnesium boryl)

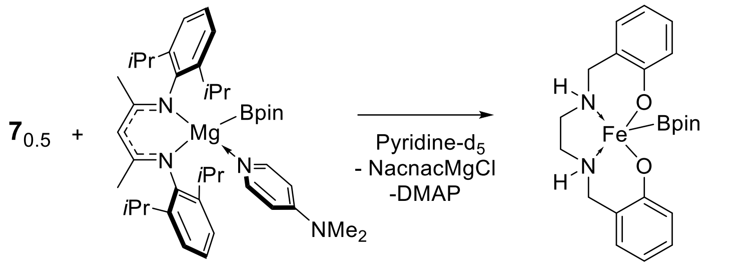

Postulated species

8

A J Young NMR tube was charged with **7** (3.6 mg, 9.96 µmol) and [Mg(^{Dipp}Nacnac)(Bpin)(DMAP)] (6.9 mg, 9.98 µmol), pyridine-d_5 was added and the solution rapidly (< 5 mins) turned deep red.

^{1}H NMR (500 MHz; py-d_5, 298 K): δ (all br.) 25.38, 23.16, 22.13, 21.25, 18.90, 18.73, 17.94, 16.76, 13.71, −3.54, −4.07, −5.09, −5.95, −6.32, −7.01, −8.01, −10.53. **^{11}B{^{1}H} NMR** (160 MHz; py-d_5, 298 K): featureless between δ 90 – (−)110.

^{1}H and ^{11}B NMR analysis of the *in-situ* reaction indicates the formation of [(DippNacnac)MgCl][24] and thus we can infer salt metathesis and formation of **8**. Isolation of **8** as red microcrystalline solid was achieved when the reaction was repeated in THF. In addition, **8** is incredibly insoluble in polar coordinating solvents tested; THF and MeCN.

9.3.4. Trimerization mediated by *in situ*-generated **8**

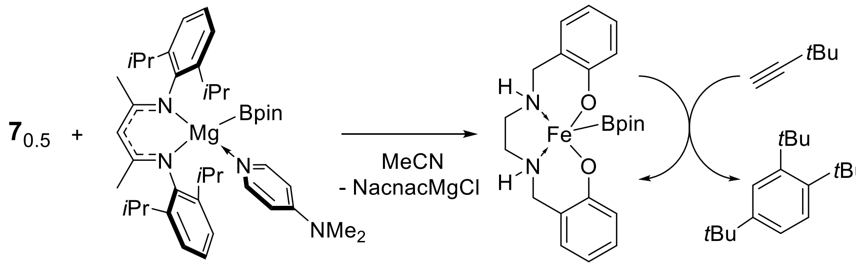

8

4-*tert*-butylphenylacetylene (46.5 µL, 0.25 mmol) was added to **7** (4.5 mg, 12.4 µmol) in MeCN-d_3 (500 µL), to this [Mg(^{Dipp}Nacnac)(Bpin)(DMAP)] (8.6 mg, 12.5 µmol) was added. The ^{1}H NMR spectrum was recorded after five minutes; the sample was heated at 80 °C for 16 hours and the ^{1}H NMR spectrum recorded again.

Cyclotrimerization occurs rapidly initially upon addition of [Mg(^{Dipp}Nacnac)(Bpin)(DMAP)] (< 5 minutes). However, the cyclotrimerization is concomitant with formation of an insoluble red precipitate (postulated as **8**). Due to the insolubility of **8**, the conversion plateaus at 28% at RT. However, after 16 hours at 80 °C the trimerization reaches 87% conversion by ^{1}H NMR spectroscopy. Control reaction using just [Mg(^{Dipp}Nacnac)(Bpin)(DMAP)] shows no cyclotrimerization product.

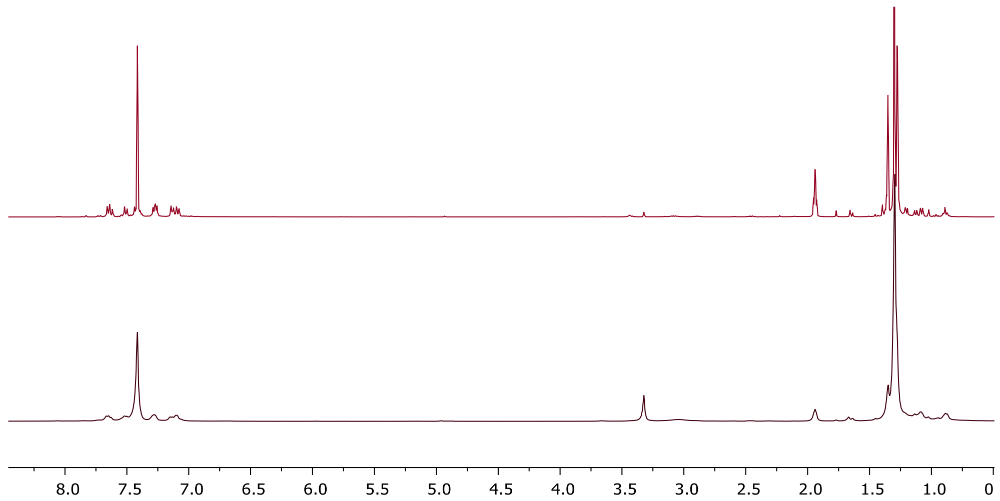

Figure S73. Trimerization mediated by *in situ*-generated **8** after 5 minutes (below) and 16 hours at 80 °C (above).

10. Potential modes of activation of precatalyst

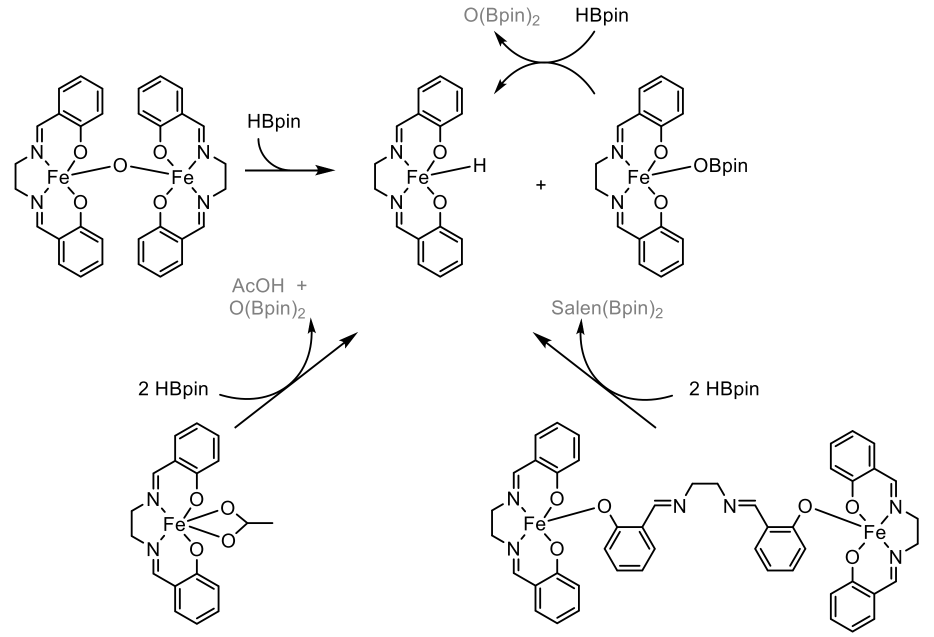

11. Single Crystal X-ray Diffraction Analysis

Data for **4a, 4b, 4c** and **7** were collected on an Agilent SuperNova diffractometer (using Cu-Kα radiation) while those for **4d** were obtained using an Agilent Xcalibur instrument and a Mo-Kα source. All experiments were conducted at 150 K, solved using SHELXT[25] and refined using SHELXL[26] via the Olex-2[27] interface. In the least-squares runs, distance and ADP restraints were employed, on merit, in disordered regions to assist convergence. Refinements were, otherwise, unremarkable except for the points outlined hereafter.

The asymmetric unit in **4a** comprises one molecule of the metal complex and a region of disordered solvent. The nitrogen bound hydrogens, in the former, were located and each refined at a distance of 0.98 Å from the relevant parent atom. The solvent was disordered to an extent that did not lend itself to ready modelling. Hence, it was treated using the solvent-mask algorithm available in Olex-2. An allowance has been made for two molecules of acetonitrile, per asymmetric unit, in the formula to account for this guest solvent. The data frames were also integrated to take account of non-merohedral sample twinning (45% approximately).

Half of a dimer molecule and a region of solvent constitute the symmetric unit in the structure of **4b**. The remainder of the main feature is generated *via* a crystallographic inversion centre. C34-36 were each modelled over two sites, in a 55:45 ratio. While the solvent was recognisable as one disordered toluene moiety, post modelling it, the electron density remained quite smeared. As such, this region of the electron density map was ultimately treated using the solvent mask algorithm in Olex-2, and allowance made for same in the formula as presented.

The asymmetric unit in the structure of **4c** comprises half of an iron complex, two molecules of benzene (based on C41 and C47, treated for 77:23 disorder) and a benzene molecule with half site occupancy (based on C53) which is crystallographically disordered with itself. Disorder in the main feature is confined to the positions of F1 (disordered with H3 in a 77:23 split) and F2 (disordered with H23 in an 83:17 ratio). During refinement, the benzene moieties were treated as rigid hexagons while hydrogens attached to N3 and N4 were readily located and refined at a distance of 0.90 Å from the parent atoms. While this crystal was not of optimal quality (as reflected in the estimated standard deviations that pertain to the *a* and *c* axes, as well as the R_{int} value), the structural characterisation is unambiguous.

In the structure of **4d**, the asymmetric unit comprises half of one dimer and three THF molecules. The electron density for two of the latter was very smeared and, ultimately, all guest solvent was treated using the solvent mask algorithm available in Olex-2, while making allowances for same in the formula. 50:50 disorder also prevailed for the fluorine atoms in the main feature and for the entire phenyl ring based on C28. The hydrogen atoms were manually added for C31 and C32, for completeness, albeit with multiple restraints because of their 25% site-occupancies. Intermolecular hydrogen-bonding contributes to the conformation of the dimer.

Half of a dimer constitutes the asymmetric unit in **7**. The remainder of the molecule arises by virtue of crystallographic inversion symmetry.

Crystallographic data for all compounds have been deposited with the Cambridge Crystallographic Data Centre as supplementary publications CCDC 2260432, 2260433, 2260599, 2260434 and 2260435 for **4a, 4b, 4c, 4d,** and **7**, respectively. Copies of these data can be obtained free of charge on application to CCDC, 12 Union Road, Cambridge CB2 1EZ, UK [fax(+44) 1223 336033, e-mail: deposit@ccdc.cam.ac.uk

Table S1. Crystal data and structure refinement for compounds **4a**, **4b**, **4c**, **4d** and **7**.

Identification code (CCDC)	**4a (2260432)**	**4b (2260433)**	**4c (2260599)**	**4d (2260434)**	**7 (2260435)**
Empirical formula	$C_{48}H_{66}B_2Fe_2N_6O_{10}$	$C_{49}H_{68}BFeN_2O_5$	$C_{55}H_{45}F_2Fe_2N_4O_{4.5}$	$C_{92}H_{104}F_3Fe_3N_6O_{14}$	$C_{16}H_{18}ClFeN_2O_2$
Formula weight	1020.38	831.71	983.65	1742.36	361.62
Crystal system	monoclinic	triclinic	monoclinic	monoclinic	monoclinic
Space group	$P2_1/n$	P-1	$C2/c$	$C2/c$	$P2_1/c$
a/ Å	13.3923(5)	13.1658(5)	23.6386(11)	13.5405(5)	8.62169(8)
b/ Å	18.0537(8)	14.0233(5)	20.0319(4)	22.5652(6)	20.3612(2)
c/ Å	21.4231(9)	14.4698(5)	20.8331(10)	27.5834(8)	8.86282(11)
α/ °	90	116.769(4)	90	90	90
β/ °	94.416(4)	95.895(3)	109.899(5)	93.293(3)	99.1657(10)
γ/ °	90	95.371(3)	90	90	90
U/ Å^3	5164.3(4)	2343.00(16)	9276.0(7)	8414.0(5)	1535.98(3)
Z	4	2	8	4	4
ρ_{calc}/ g cm^{-3}	1.312	1.179	1.409	1.375	1.564
μ/ mm^{-1}	4.997	2.933	5.522	0.586	9.543
F(000)	2152.0	894.0	4072.0	3660.0	748.0
Crystal size/ mm^3	0.095 × 0.077 × 0.021	0.158 × 0.085 × 0.062	0.182 × 0.073 × 0.054	0.51 × 0.4 × 0.279	0.192 × 0.116 × 0.064
2θ range for data collection/°	7.532 to 146.308	6.834 to 145.408	7.956 to 145.456	6.028 to 56.562	8.686 to 145.214
Index ranges	$-16 \le h \le 13$,<br>$-21 \le k \le 22$,<br>$-23 \le l \le 26$	$-16 \le h \le 16$,<br>$-17 \le k \le 13$,<br>$-16 \le l \le 17$	$-29 \le h \le 29$,<br>$-24 \le k \le 15$,<br>$-25 \le l \le 25$	$-17 \le h \le 16$,<br>$-30 \le k \le 30$,<br>$-34 \le l \le 36$	$-5 \le h \le 10$,<br>$-25 \le k \le 25$,<br>$-10 \le l \le 10$
Reflections collected	14051	23359	51751	35884	15113
Independent reflections, R_{int}	140510.0694*	92390.0584	9177, 0.0985	103270.0429	30440.0237
Data/restraints/parameters	14051/4/585	9239/16/510	9177/394/711	10327/88/463	3044/0/199
Goodness–of–fit on F^2	0.806	1.018	1.024	1.042	1.041
Final $R1$, $wR2$ [I>=2σ(I)]	0.0597, 0.1294	0.0482, 0.1155	0.0582, 0.1265	0.0789, 0.2431	0.0231, 0.0611
Final $R1$, $wR2$ [all data]	0.1189, 0.1428	0.0616, 0.1235	0.0582, 0.1265	0.0950, 0.2586	0.0240, 0.0618
Largest diff. peak/hole/ e Å^{-3}	0.43/−0.38	0.38/−0.33	0.47/−0.39	0.90/−0.46	0.27/−0.34

*R_{int} pertaining to HKLF5 file (based on comparison of scale factors arising from the component analysis for each domain and overlapping reflections which contribute to each twinned component).

12. Quantum-Chemical Studies

12.1. Computational Methods

Quantum chemical calculations were carried out with the Gaussian 16[28] and ORCA 5[29,30] programs. With Gaussian 16, molecular geometries were optimzed at the density functional theory (DFT) level employing the generalized gradient approximation (GGA) via the PBE[31,32] functional in conjunction with the D3 atom-pairwise dispersion correction without damping[33] and an implicit polarizable continuum solvent model[34] utilizing acetonitrile as the solvent. The split-valence double-zeta def2-SVP[35] basis set was used together with the corresponding auxiliary Coulomb-fitting basis set of Weigend.[36] At this GGA DFT level, abbreviated as PBE-D3(PCM)/def2-SVP, frequency calculations were performed on the optimized stationary points to characterize minima and transition structures, and to extract thermal contributions to enthalpies and Gibbs energies at 298.15 K. Single-point energy calculations were performed at the hybrid DFT level with 25 % admixture of Fock-exchange via the PBE0[37,38] functional employing the triple-zeta valence polarized def2-TZVP basis set[35] and the same dispersion and solvent corrections as above for improved relative energies. The final relative Gibbs energies are thus reported at the PBE0-D3(PCM)/def2-TZVP // PBE-D3(PCM)/def2-SVP level.

To ascertain relative energies for selected species, correlated *ab initio* calculations were performed with ORCA 5 utilizing the domain-based local pair natural orbital approximation for coupled-cluster calculations including single and double excitations and semicanonical perturbative triple excitations, DLPNO-CCSD(T).[39] The one-particle space was described with the triple-zeta def2-TZVPP and quadruple-zeta def2-QZVPP basis sets[35] on all atoms. The corresponding auxiliary Coulomb[36] and correlation[40] fitting basis sets were used throughout together with the RIJCOSX[41] algorithm. The reference and correlation energies were extrapolated to the complete basis set (CBS) limit, CBS(T,Q),[42] according to

$$E_{\text{ref}}(\text{CBS}) = E_{\text{ref}}(Q) + \frac{E_{\text{ref}}(Q) - E_{\text{ref}}(T)}{\exp(\alpha\,(\sqrt{4} - \sqrt{3})) - 1}$$

$$E_{\text{corr}}(\text{CBS}) = \frac{3^{\beta}\,E_{\text{corr}}(T) - 4^{\beta}\,E_{\text{corr}}(Q)}{3^{\beta} - 4^{\beta}}$$

with cardinal numbers 3 for T and 4 for Q, and α = 7.88, β = 2.97.

The `NormalPNO` settings in ORCA convention were employed with pair natural orbital (PNO) thresholds set to `TCutPNO` = 10^{-5}, 10^{-6}, or 10^{-7}, in order to arrive at an extrapolated PNO limit, PNO(5,6) or PNO(6,7),[43] for the correlation energies according to

$$E_{\text{corr}}(\text{PNO}) = E_{\text{corr}}(\text{PNO-}X) + F_{\text{PNO}} \times (E_{\text{corr}}(\text{PNO-}Y) - E_{\text{corr}}(\text{PNO-}X))$$

where F_{PNO} = 1.5, and PNO-X and PNO-Y correspond to `TCutPNO` thresholds differing by one order of magnitude with $X < Y$.

12.2. Spin-State Energetics: Fe^{II}(salen)

According to DLPNO-CCSD(T) reference calculations, the square planar iron(II) complex Fe^{II}(salen) is a high-spin quintet species, clearly separated from the intermediate-spin triplet and the low-spin singlet electromers (Table S2). Spin-state energetics are close to convergence already with the PNO(5,6) extrapolation sequence, and the overall picture does not change with the computationally much more demanding PNO(6,7) approach. A DFT single-point energy benchmark with selected functionals, performed without polarized continuum solvent model for consistency with the DLPNO-CCSD(T) reference, shows that, next to the heavily parametrized meta-GGA functional M06L, the hybrid functionals with a Fock-exchange admixture of 25 % or more can reproduce this trend in relative energies (Table S4). The PBE0-D3/def2-TZVP level appears an appropriate choice in this context.

Table S2: Spin-state energetics (E_{rel} in kcal mol^{-1}) for Fe^{II}(salen) computed at the DLPNO-CCSD(T) / def2-nZVPP level (n = T, Q), extrapolated to the complete basis set CBS(T,Q) and pair natural orbital PNO(X,Y) limits. Molecular geometries are optimized with PBE-D3(PCM)/def2-SVP.

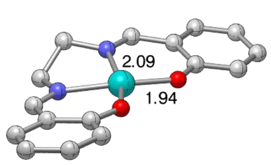

Fe^{II}(salen) ^{5}**HS**

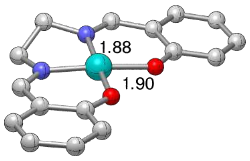

Fe^{II}(salen) ^{3}**IS**

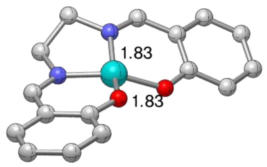

Fe^{II}(salen) ^{1}**LS**

	TCutPNO = 10^{-5}			TCutPNO = 10^{-6}			TCutPNO = 10^{-7}			PNO(5,6)	PNO(6,7)
	n = T	n = Q	CBS(T,Q)	n = T	n = Q	CBS(T,Q)	n = T	n = Q	CBS(T,Q)	CBS(T,Q)	CBS(T,Q)
^{5}**HS**	0.0	0.0	0.0	0.0	0.0	0.0	0.0	0.0	0.0	0.0	0.0
^{3}**IS**	22.8	22.1	19.2	19.0	18.0	14.9	16.4	15.6	12.5	12.7	11.3
^{1}**LS**	65.8	64.3	61.7	60.9	59.0	56.1	54.5	53.0	50.4	53.3	47.5

Table S3: Total energies (Hartree) for spin-state energetics of Fe^{II}(salen) corresponding to Table S2; Hartree–Fock reference energies, E_{tot}(ref), final total energies, E_{tot}(CCSD(T)), and the $\mathcal{T}_1$ diagnostic for singles amplitudes are given.

	def2-TZVPP			def2-QZVPP			CBS(T,Q)	
	E_{tot}(ref)	E_{tot}(CCSD(T))	$\mathcal{T}_1$	E_{tot}(ref)	E_{tot}(CCSD(T))	$\mathcal{T}_1$	E_{tot}(ref)	E_{tot}(CCSD(T))
	TCutPNO = 10^{-5}							
^{5}**HS**	−2135.517 305	−2139.624 615	0.010	−2135.583 168	−2139.931 673	0.010	−2135.592 240	−2140.119 407
^{3}**IS**	−2135.438 293	−2139.588 266	0.012	−2135.497 744	−2139.896 398	0.011	−2135.505 933	−2140.088 794
^{1}**LS**	−2135.350 157	−2139.519 799	0.013	−2135.411 923	−2139.829 172	0.012	−2135.420 431	−2140.021 092
	TCutPNO = 10^{-6}							
^{5}**HS**	−2135.517 305	−2139.653 716	0.013	−2135.583 168	−2139.950 041	0.013	−2135.592 240	−2140.129 824
^{3}**IS**	−2135.438 294	−2139.623 373	0.015	−2135.497 744	−2139.921 311	0.015	−2135.505 933	−2140.106 157
^{1}**LS**	−2135.350 157	−2139.556 751	0.017	−2135.411 923	−2139.855 997	0.016	−2135.420 431	−2140.040 416
	TCutPNO = 10^{-7}							
^{5}**HS**	−2135.517 305	−2139.666 249	0.014	−2135.583 166	−2139.960 663	0.014	−2135.592 238	−2140.139 034
^{3}**IS**	−2135.438 293	−2139.640 059	0.017	−2135.497 744	−2139.935 860	0.017	−2135.505 933	−2140.119 122
^{1}**LS**	−2135.350 157	−2139.579 469	0.022	−2135.411 923	−2139.876 223	0.020	−2135.420 431	−2140.058 795

Table S4: DFT-D3/def2-TZVP single-point energy benchmark at PBE-D3(PCM)/def2-SVP geometries computed with selected functionals in vacuo (without implict solvent correction) for the spin-state energetics (E_{rel} in kcal mol^{-1}) of Fe^{II}(salen). Extrapolated DLPNO-CCSD(T) reference values in blue color.

**DLPNO-CCSD(T)**	^{5}**HS**	^{3}**IS**	^{1}**LS**
CBS(T,Q)/PNO(5,6)	0.0	12.7	53.3
CBS(T,Q)/PNO(6,7)	0.0	11.3	47.5
**Functional**			
PBE-D3	0.0	−12.1	2.4
PBE0-D3	0.0	4.4	39.2
BLYP-D3	0.0	−11.4	2.4
B3LYP-D3	0.0	0.4	29.9
M06L-D3	0.0	4.7	29.5
M06-D3	0.0	12.5	43.4
M06-2X-D3	0.0	22.7	65.6
TPSS-D3	0.0	−13.8	3.7
TPSSh-D3	0.0	−7.2	19.0
TPSS0-D3	0.0	2.6	39.0

Table S5: Total energies (Hartree) for spin-state energetics of Fe^{II}(salen) corresponding to Table S4, computed in vacuo; extrapolated DLPNO-CCSD(T) reference values in blue color.

**DLPNO-CCSD(T)**	^{5}**HS**	^{3}**IS**	^{1}**LS**
CBS(T,Q)/PNO(5,6)	−2140.135 033	−2140.114 839	−2140.050 078
CBS(T,Q)/PNO(6,7)	−2140.143 641	−2140.125 605	−2140.067 985
**Functional**			
PBE-D3	−2140.951 729	−2140.970 950	−2140.947 922
PBE0-D3	−2141.004 048	−2140.997 117	−2140.941 654
BLYP-D3	−2142.029 272	−2142.047 386	−2142.025 490
B3LYP-D3	−2142.297 046	−2142.296 384	−2142.249 404
M06L-D3	−2142.075 141	−2142.067 697	−2142.028 197
M06-D3	−2141.555 725	−2141.535 870	−2141.486 560
M06-2X-D3	−2141.841 106	−2141.804 921	−2141.736 613
TPSS-D3	−2142.455 727	−2142.477 654	−2142.449 883
TPSSh-D3	−2142.326 419	−2142.337 957	−2142.296 109
TPSS0-D3	−2142.147 034	−2142.142 922	−2142.084 931

12.3. Isomers of Species A

For the Fe-boryl hydride species **A**, DFT computations predict two closed-shell singlet isomers ^{1}**A** and ^{1}**A-1**, which persist independent of functional choice or basis set as compiled in Table S6. Isomer ^{1}**A-1** features a shorter Fe–O^{Bpin} distance (also cf. the overlay of the isomers in Figure S74) and is energetically favored over ^{1}**A**. This energetic preference is confirmed by DLPNO-CCSD(T)/CBS(T,Q) calculations using the PNO(5,6) extrapolation sequence (Table S7). However, a quintet high-spin electromer, ^{5}**A-1**, is clearly more stable than its singlet low-spin ^{1}**A-1** or triplet intermediate-spin ^{3}**A-1** congeners. Moreover, a 1,2-hydrogen shift from ^{5}**A-1** to yield species ^{5}**B** is exergonic by more than 30 kcal mol^{-1} (Table S9).

Table S6: Selected distances (Å) for the two closed-shell singlet isomers of **A**, i.e., ^{1}**A** and ^{1}**A-1**. Geometries are optimized at DFT-D3(PCM,Acetonitrile) levels with D3 dispersion correction and implicit solvent model using the def2-SVP and def2-TZVP basis sets as specified in the Table. The first two entries for each isomer are computed in full analogy to Ref. 18 using mixed ZORA-def2-TZVP(-f) and ZORA-def2-SVP basis sets in conjunction with the ZORA Hamiltonian; these calculations were carried out with the newer ORCA5 program employing the SHARK integral package and applying the tightest available integration grid (`DefGrid3` in ORCA convention).

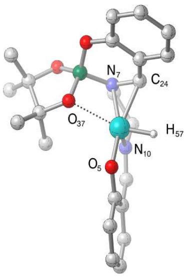

^{1}**A**

**DFT-Level**	Fe–O(5)	Fe–N(10)	Fe–N(7)	Fe–C(24)	N(7)–C(24)	Fe–O(37)	Fe–H(57)
BP86 / ZORA	1.828	1.832	2.055	2.093	1.387	2.506	1.480
PBE / ZORA	1.829	1.830	2.044	2.076	1.389	2.515	1.481
BP86 / def2SVP	1.837	1.833	2.065	2.118	1.388	2.458	1.483
BP86 / def2TZVP	1.836	1.836	2.050	2.111	1.382	2.490	1.484
PBE / def2SVP	1.839	1.832	2.057	2.098	1.388	2.467	1.482
PBE / def2TZVP	1.837	1.834	2.042	2.091	1.383	2.502	1.483
PBE0 / def2SVP	1.858	1.888	2.033	2.071	1.367	2.491	1.472
PBE0 / def2TZVP	1.851	1.878	2.028	2.075	1.359	2.521	1.475

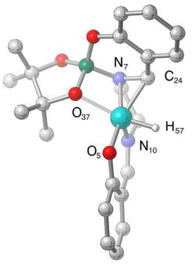

^{1}**A-1**

**DFT-Level**	Fe–O(5)	Fe–N(10)	Fe–N(7)	Fe–C(24)	N(7)–C(24)	Fe–O(37)	Fe–H(57)
BP86 / ZORA	1.839	1.818	1.973	1.997	1.392	2.124	1.473
PBE / ZORA	1.841	1.817	1.972	1.993	1.392	2.139	1.473
BP86 / def2SVP	1.845	1.820	1.978	1.998	1.393	2.122	1.473
BP86 / def2TZVP	1.846	1.825	1.980	2.011	1.386	2.151	1.478
PBE / def2SVP	1.845	1.819	1.976	1.995	1.392	2.138	1.472
PBE / def2TZVP	1.845	1.823	1.978	2.004	1.386	2.173	1.477
PBE0 / def2SVP	1.857	1.863	1.959	1.972	1.373	2.125	1.465
PBE0 / def2TZVP	1.856	1.860	1.962	1.983	1.366	2.154	1.469

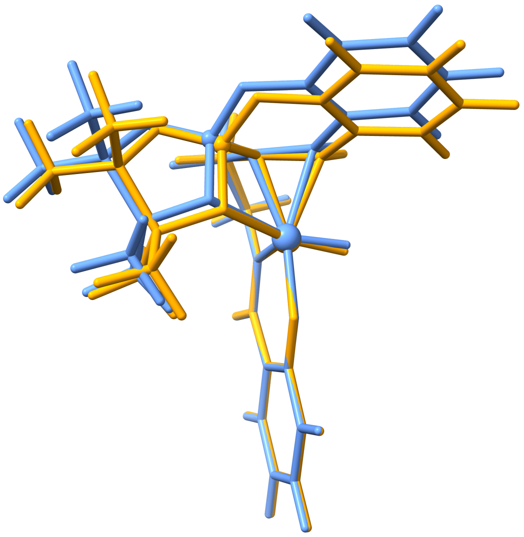

Figure S74: Overlay for the two closed-shell singlet isomers of **A**, i.e., ^{1}**A** (blue) and ^{1}**A-1** (orange). The overlay minimizes the mean square distances between the atom positions of iron and the consecutive O, C, C, C, N atoms of the lower salen arm (RMSD: 0.011 Å). Geometries are optimized with PBE-D3(PCM,Acetonitrile)/def2-SVP.

Table S7: Relative energies (kcal mol^{-1}) of closed-shell singlet isomers ^{1}**A** and ^{1}**A-1** corresponding to Table S6.

DFT-Level	$\Delta_r E(^1\mathbf{A} \rightarrow {}^1\mathbf{A\text{-}1})$	$\Delta_r G^{298}(^1\mathbf{A} \rightarrow {}^1\mathbf{A\text{-}1})$
BP86 / ZORA	−3.03	−3.13
PBE / ZORA	−3.67	−3.24
BP86 / def2SVP	−3.16	−3.38
BP86 / def2TZVP	−1.65	−1.95
PBE / def2SVP	−3.93	−3.63
PBE / def2TZVP	−2.43	−2.28
PBE0 / def2SVP	−4.20	−3.56
PBE0 / def2TZVP	−2.88	−2.32
DLPNO-CCSD(T)/CBS(T,Q)[a]	−8.86	−8.56

[a]DLPNO-CCSD(T)/def2{T,Q}ZVPP , *TCutPNO* = $\{10^{-5}, 10^{-6}\}$
extrapolated to the CBS(T,Q) and PNO(5,6) limits,
computed at RI-PBE-D3(PCM)/def2SVP geometries.

Table S8: Total energies and Gibbs energy corrections (Hartree) of closed-shell singlet isomers ^{1}**A** and ^{1}**A-1** corresponding to Table S7.

DFT-Level	Species	E_{tot}	G^{298}_{corr}
BP86 / ZORA	^{1}**A**	−2568.832 574	0.401 419
	^{1}**A-1**	−2568.837 396	0.401 588
PBE / ZORA	^{1}**A**	−2566.756 810	0.401 150
	^{1}**A-1**	−2566.762 662	0.400 466
BP86 / def2SVP	^{1}**A**	−2552.984 394	0.397 530
	^{1}**A-1**	−2552.989 432	0.397 177
BP86 / def2TZVP	^{1}**A**	−2554.554 410	0.396 594
	^{1}**A-1**	−2554.557 040	0.396 117
PBE / def2SVP	^{1}**A**	−2550.944 826	0.396 982
	^{1}**A-1**	−2550.951 084	0.397 457
PBE / def2TZVP	^{1}**A**	−2552.500 152	0.395 396
	^{1}**A-1**	−2552.504 023	0.395 632
PBE0 / def2SVP	^{1}**A**	−2551.014 625	0.413 766
	^{1}**A-1**	−2551.021 319	0.414 779
PBE0 / def2TZVP	^{1}**A**	−2552.557 656	0.412 211
	^{1}**A-1**	−2552.562 243	0.413 096
DLPNO-CCSD(T)/CBS(T,Q)[a]	^{1}**A**	−2551.466 025	0.396 982
	^{1}**A-1**	−2551.480 142	0.397 457

[a]DLPNO-CCSD(T)/def2{T,Q}ZVPP , *TCutPNO* = $\{10^{-5}, 10^{-6}\}$
extrapolated to the CBS(T,Q) and PNO(5,6) limits,
computed at RI-PBE-D3(PCM)/def2SVP geometries.

Table S9: Relative Gibbs energies (kcal mol^{-1}) of low-spin singlet, intermediate-spin triplet, and high-spin quintet electromers for species **A-1**, the transition structure **TS(A-1,B)**, and the product of a 1,2-hydrogen shift **B**. Molecular geometries are optimized at the GGA DFT level PBE-D3(PCM)/def2-SVP, and further single-point energy calculations are carried out at the PBE0-D3(PCM)/def2-TZVP hybrid DFT and the DLPNO-CCSD(T)/CBS(T,Q) levels; the latter DLPNO scheme was extrapolated to the PNO(5,6) limit. Energies are given relative to the corresponding ^{1}**A-1** electromer.

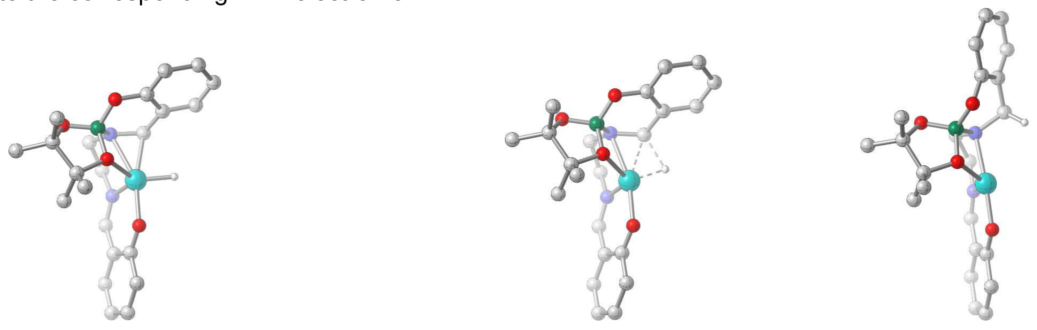

^{5}**A-1** ^{5}**TS(A-1,B)** ^{5}**B**

	$\Delta_r G^{298}$(w.r.t. ^{1}**A-1**)		
	**A-1**	**TS(A-1,B)**	**B**
**PBE-D3(PCM)/def2-SVP**			
^{1}LS	0.0	7.9	7.9
^{3}IS	8.1	7.9	−13.1
^{5}HS	8.8	10.0	−10.1
**PBE0-D3(PCM)/def2-TZVP // PBE-D3(PCM)/def2-SVP**			
^{1}LS	0.0	−5.1	−10.9
^{3}IS	−7.2	−8.0	−41.2
^{5}HS	−18.8	−21.6	−55.0
**DLPNO-CCSD(T)/CBS(T,Q) // PBE-D3(PCM)/def2-SVP**			
^{1}LS	0.0	1.5	−6.4
^{3}IS	−0.3	−1.5	−35.6
^{5}HS	−21.2	−22.0	−54.3

Table S10: Total energies and Gibbs energy corrections (Hartree) for species **A-1**, the transition structure **TS(A-1,B)**, and the product of a 1,2-hydrogen shift **B** corresponding to Table S9

	**A-1**		**TS(A-1,B)**		**B**	
**PBE-D3(PCM)/def2-SVP**						
	E_{tot}	G_{corr}	E_{tot}	G_{corr}	E_{tot}	G_{corr}
^{1}LS	−2550.951 084	0.397 457	−2550.935 658[a]	0.394 553	−2550.939 643	0.398 608
^{3}IS	−2550.935 187	0.394 399	−2550.934 982[b]	0.393 986	−2550.971 934	0.397 503
^{5}HS	−2550.929 873	0.390 266	−2550.927 024[c]	0.389 260	−2550.963 941	0.394 301
**PBE0-D3(PCM)/def2-TZVP // PBE-D3(PCM)/def2-SVP**						
^{1}LS	−2552.548 683		−2552.553 820[d]		−2552.567 225	
^{3}IS	−2552.557 025		−2552.557 970		−2552.614 342	
^{5}HS	−2552.571 430		−2552.574 873		−2552.633 100	
**DLPNO-CCSD(T)/CBS(T,Q) // PBE-D3(PCM)/def2-SVP**						
^{1}LS	−2551.480 142		−2551.474 878		−2551.491 551	
^{3}IS	−2551.477 551		−2551.479 033		−2551.536 951	
^{5}HS	−2551.506 733		−2551.507 030		−2551.563 528	

[a]TS optimized as broken-symmetry singlet species with $\langle S^2 \rangle$ = 0.45 and ν_{imag} = 605 i cm^{-1}.
[b]ν_{imag} = 262 i cm^{-1}.
[c]ν_{imag} = 606 i cm^{-1}.
[d]TS single point energy computed as broken-symmetry singlet with $\langle S^2 \rangle$ = 0.70.

12.4. Coordination Sphere of 2a–2d′

To quantify the structural flexibility of the iron coordination sphere in **2a**–**2d′**, the molecular geometries of the corresponding mono-metallic congeners **2a-mono**–**2d′-mono** were computed. Figure S75 shows the structures in their most stable high-spin configuration. The two largest valence angles around the metal center, α and β (degrees), are used for the evaluation of the coordination parameter τ_4' (see Table S11), where τ_4' = 0 relates to a square-planar and τ_4' = 1 to a tetrahedral environment.[44]

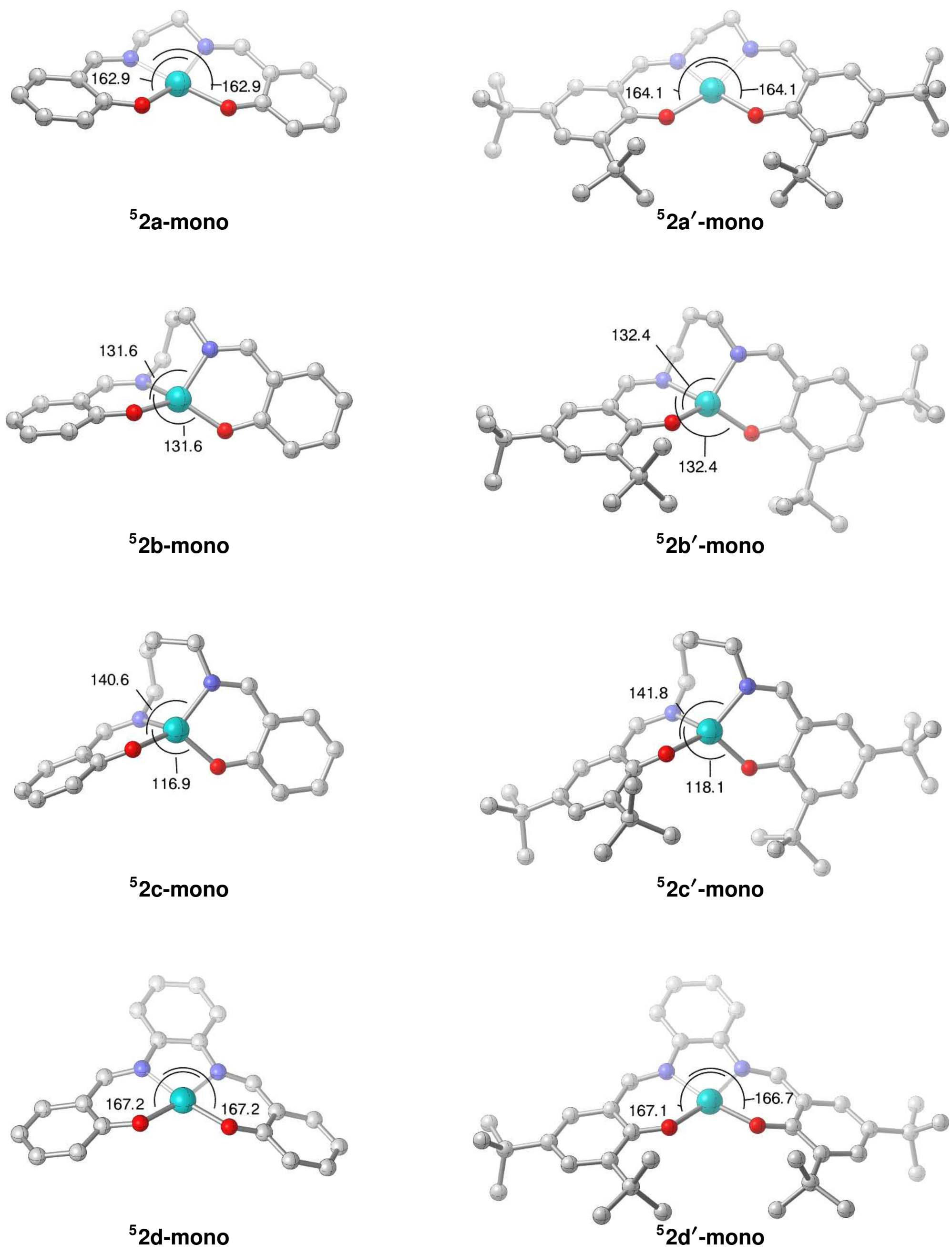

Figure S75: Optimized molecular structures (PBE-D3(PCM)/def2-SVP) for high-spin mono-metallic congeners of **2a**–**2d′**.

Table S11: Structural environment parameters and energies for mono-metallic congeners of **2a–2d′**. The largest valence angles α and β (degrees) around the iron center and the structural index parameter τ_4' are given along with total energies, Gibbs energy corrections (Hartree), and relative Energies (kcal mol^{-1}).

**PBE0-D3(PCM)/def2-TZVP // PBE-D3(PCM)/def2-SVP**							
	α(O–Fe–N)	β(O–Fe–N)	τ_4'	E_{tot}	G_{corr}	E_{rel}	G_{rel}
^{5}**2a-mono**	162.9	162.9	0.24	−2141.027 249	0.215 265	0.0	0.0
^{3}**2a-mono**	175.2	175.2	0.07	−2141.018 352	0.218 825	5.6	7.8
^{1}**2a-mono**	156.3	156.3	0.34	−2140.958 374	0.220 322	43.2	46.4
^{5}**2b-mono**	131.6	131.6	0.69	−2180.308 633	0.242 334	0.0	0.0
^{3}**2b-mono**	149.0	149.0	0.44	−2180.287 154	0.244 800	13.5	15.0
^{1}**2b-mono**	136.2	136.2	0.62	−2180.239 410	0.246 782	43.4	46.2
^{5}**2c-mono**	116.9	140.6	0.65	−2219.589 837	0.268 686	0.0	0.0
^{3}**2c-mono**	138.7	147.3	0.50	−2219.562 120	0.270 304	17.4	18.4
^{1}**2c-mono**	109.6	155.3	0.53	−2219.526 386	0.273 114	39.8	42.6
^{5}**2d-mono**	167.2	167.2	0.18	−2293.338 232	0.234 739	0.0	0.0
^{3}**2d-mono**	179.8	179.8	0.00	−2293.329 763	0.237 486	5.3	7.0
^{1}**2d-mono**	173.8	173.8	0.09	−2293.269 450	0.237 739	43.2	45.0
^{5}**2a′-mono**	164.1	164.1	0.23	−2769.507 859	0.623 241	0.0	0.0
^{3}**2a′-mono**	174.4	174.4	0.08	−2769.500 900	0.628 813	4.4	7.9
^{1}**2a′-mono**	163.0	163.0	0.24	−2769.442 977	0.630 274	40.7	45.1
^{5}**2b′-mono**	132.4	132.4	0.67	−2808.788 979	0.650 672	0.0	0.0
^{3}**2b′-mono**	151.6	151.6	0.40	−2808.770 146	0.654 565	11.8	14.3
^{1}**2b′-mono**	139.4	139.4	0.58	−2808.722 552	0.655 850	41.7	44.9
^{5}**2c′-mono**	118.1	141.8	0.64	−2848.069 456	0.677 127	0.0	0.0
^{3}**2c′-mono**	142.7	150.8	0.45	−2848.044 745	0.680 863	15.5	17.9
^{1}**2c′-mono**	110.4	157.9	0.50	−2848.008 470	0.681 431	38.3	41.0
^{5}**2d′-mono**	166.7	167.1	0.18	−2921.820 433	0.642 142	0.0	0.0
^{3}**2d′-mono**	174.9	174.9	0.07	−2921.813 430	0.648 076	4.4	8.1
^{1}**2d′-mono**	168.8	168.8	0.16	−2921.750 349	0.649 847	44.0	48.8

12.5. Regioselectivity for 1,2,4- and 1,3,5-triphenylbenzene

DLPNO-CCSD(T)/CBS(T,Q) calculations show that the 1,2,4- and 1,3,5-isomers of triphenylbenzene are isoenergetic, i.e., $\Delta G(1,3,5 \rightarrow 1,2,4) = 0.6\,\text{kcal}\,\text{mol}^{-1}$, see Table S12. The TightPNO setting was employed with PNO thresholds set to values of 10^{-7} and 10^{-8}, where the former is already very close to convergence in conjunction with the quadruple-zeta basis set. Calculations for the diphenyl-diene isomers in Figure S76 are thus reported for DLPNO-CCSD(T)/def2-QZVPP with `TCutPNO` $=10^{-7}$. An analysis of the relative energies of the various conformers of the dimerization isomers, i.e. 1,4-, 1,3-, and 2,3-diphenyl-dienes, shows that the linear and coplanar conformation is lowest in energy (Table S13). Other than may perhaps be expected, stabilization by dispersion between the phenyl rings in a π-stacked arrangement appears to be offset by the greater strain in the diene backbone. The fact that the co-planar **1,4-a** regioisomer lies $1.7\,\text{kcal}\,\text{mol}^{-1}$ below the most stable 1,3-regioisomer **1,3-a** is, notwithstanding, in favor of formation of the 1,2,4-triphenylbenzene product.

Table S12: Total energies (Hartree) for two regioisomers of triphenylbenzene; Hartree–Fock reference energies, E_{tot}(ref), final total energies, E_{tot}(CCSD(T)), and the $\mathcal{T}_1$ diagnostic for singles amplitudes are given, along with relative energies ($\text{kcal}\,\text{mol}^{-1}$) for the isomerization 1,3,5-triphenylbenzene $\rightarrow$ 1,2,4-triphenylbenzene. Geometries and Gibbs contributions computed with PBE-D3(PCM)/def2-SVP.

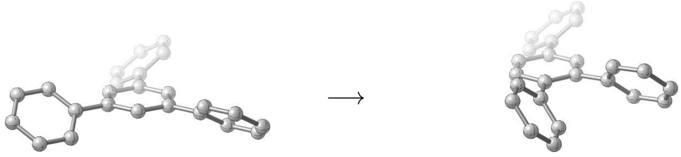

**DLPNO-CCSD(T)/def2-*n*ZVPP (TightPNO, UseFullLMP2Guess=True) // PBE-D3(PCM)/def2-SVP**									
basis set	`TCutPNO`	$E_{\text{tot}}^{\text{ref}}$	$E_{\text{tot}}^{\text{CCSD(T)}}$	$\mathcal{T}_1$	$E_{\text{tot}}^{\text{ref}}$	$E_{\text{tot}}^{\text{CCSD(T)}}$	$\mathcal{T}_1$	$E_{\text{rel}}^{\text{CCSD(T)}}$	$G_{\text{rel}}^{\text{CCSD(T)}}$
		**1,3,5-triphenylbenzene**			**1,2,4-triphenylbenzene**				
def2-TZVPP	10^{-7}	−919.646 045	−923.673 139	0.010	−919.639 337	−923.671 199	0.010	1.22	0.35
def2-TZVPP	10^{-8}	−919.646 045	−923.675 380	0.010	−919.639 337	−923.673 408	0.010	1.24	0.37
def2-QZVPP	10^{-7}	−919.682 215	−923.914 598	0.010	−919.675 482	−923.912 377	0.010	1.39	0.52
def2-QZVPP	10^{-8}	−919.682 215	−923.915 767	0.011	−919.675 482	−923.913 593	0.010	1.36	0.49
CBS(T,Q)	PNO(7,8)	−919.687 196	−924.072 209	0.011	−919.680 461	−924.069 953	0.010	1.42	0.55

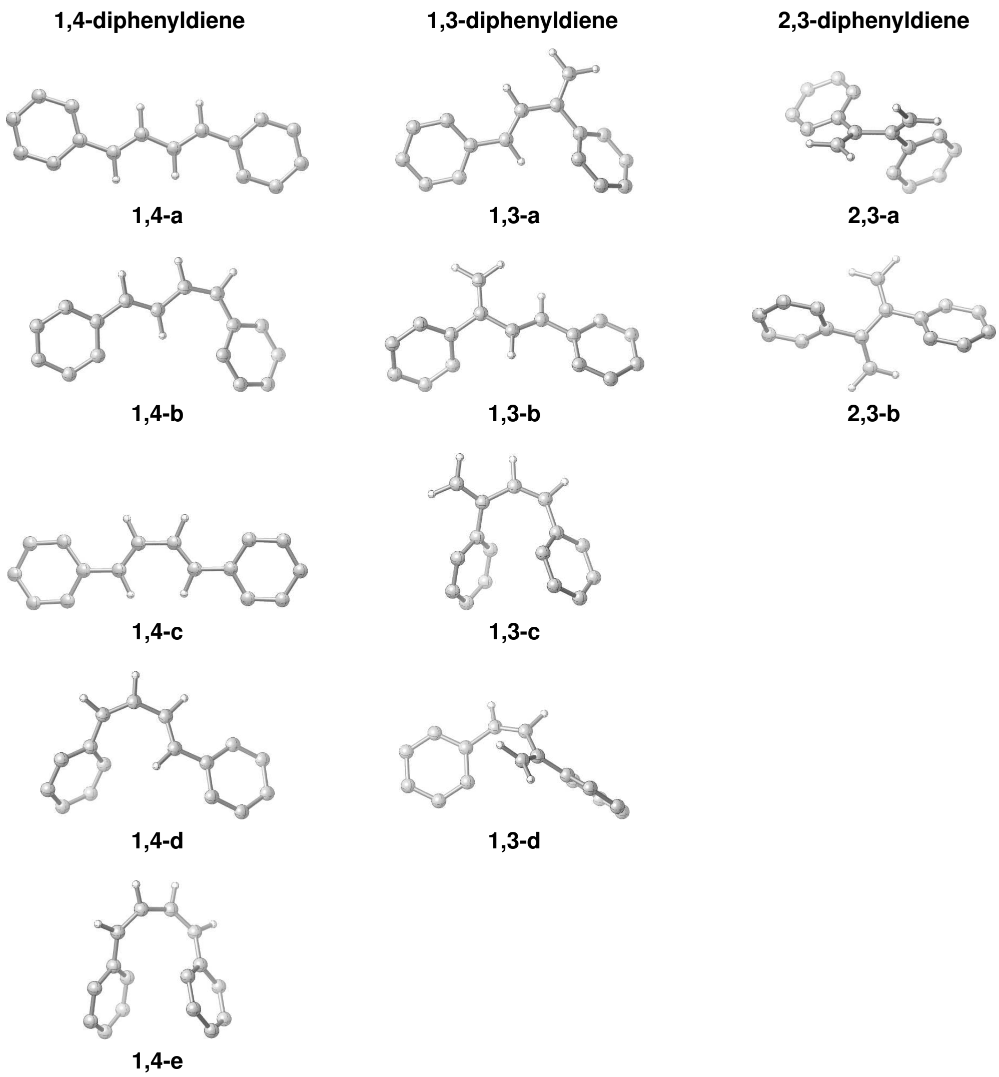

Figure S76: Optimized molecular structures (PBE-D3(PCM)/def2-SVP) for various conformers of 1,4-, 1,3-, and 2,3-diphenyl-dienes.

Table S13: Total energies (Hartree) for various conformers of 1,4-, 1,3-, and 2,3-diphenyl-dienes; Hartree–Fock reference energies, E_{tot}(ref), final total energies, E_{tot}(CCSD(T)), and the $\mathcal{T}_1$ diagnostic for singles amplitudes are given, along with relative energies (kcal mol^{-1}) with respect to the most stable diphenyldiene. Geometries and Gibbs contributions computed with PBE-D3(PCM)/def2-SVP.

**DLPNO-CCSD(T)/def2-QZVPP // PBE-D3(PCM)/def2-SVP (TightPNO, TCutPNO=10^{-7}, UseFullLMP2Guess=True)**					
	$E_{\mathrm{tot}}^{\mathrm{ref}}$	$E_{\mathrm{tot}}^{\mathrm{CCSD(T)}}$	$\mathcal{T}_1$	$E_{\mathrm{rel}}^{\mathrm{CCSD(T)}}$	$G_{\mathrm{rel}}^{\mathrm{CCSD(T)}}$
**1,4-a**	−614.252 500	−617.104 389	0.011	0.00	0.00
**1,4-b**	−614.247 206	−617.101 053	0.011	2.09	2.08
**1,4-c**	−614.244 749	−617.098 305	0.011	3.82	3.51
**1,4-d**	−614.238 792	−617.094 395	0.011	6.27	5.85
**1,4-e**	−614.229 257	−617.093 303	0.010	6.96	8.05
**1,3-a**	−614.245 680	−617.101 390	0.011	1.88	1.66
**1,3-b**	−614.243 290	−617.098 732	0.011	3.55	3.16
**1,3-c**	−614.233 478	−617.096 699	0.010	4.83	5.20
**1,3-d**	−614.237 462	−617.094 905	0.010	5.95	5.28
**2,3-a**	−614.237 935	−617.098 891	0.010	3.45	3.76
**2,3-b**	−614.237 392	−617.097 843	0.010	4.11	4.47

Table S14: DFT results for regioisomers of triphenylbenzene and diphenyldiene. Total energies and Gibbs energy corrections (Hartree) computed with PBE0-D3(PCM)/def2-TZVP // PBE-D3(PCM)/def2-SVP, along with corresponding relative energies (kcal mol^{-1}) with respect to the lowest-energy regioisomer. Term symbols for irreducible representation and point groups are also given.

**PBE0-D3(PCM)/def2-TZVP // PBE-D3(PCM)/def2-SVP**						
Species	irrep	PG	E_{tot}	G_{corr}^{298}	E_{rel}	G_{rel}^{298}
**1,3,5**	$^1A_1$	(D_3)	−924.647 647	0.287 379	0.0	0.0
**1,2,4**	^1A	(C_1)	−924.644 317	0.285 991	2.1	1.2
**1,4-a**	^1A_g	(C_{2h})	−617.592 552	0.200 030	0.0	0.0
**1,4-b**	^1A	(C_1)	−617.587 617	0.200 019	3.1	3.1
**1,4-c**	^1A	(C_2)	−617.586 021	0.199 546	4.1	3.8
**1,4-d**	^1A	(C_1)	−617.579 551	0.199 361	8.2	7.7
**1,4-e**	^1A	(C_1)	−617.577 488	0.201 770	9.5	10.5
**1,3-a**	^1A	(C_1)	−617.584 360	0.199 681	5.1	4.9
**1,3-b**	^1A	(C_1)	−617.581 528	0.199 406	6.9	6.5
**1,3-c**	^1A	(C_1)	−617.578 210	0.200 618	9.0	9.4
**1,3-d**	^1A	(C_1)	−617.575 878	0.198 953	10.5	9.8
**2,3-a**	^1A	(C_2)	−617.578 215	0.200 529	9.0	9.3
**2,3-b**	^1A	(C_2)	−617.576 704	0.200 590	9.9	10.3

13. References

(1) Voronova, K.; Purgel, M.; Udvardy, A.; Bényei, A. C.; Kathó, Á.; Joó, F., Hydrogenation and Redox Isomerization of Allylic Alcohols Catalyzed by a New Water-Soluble Pd-tetrahydrosalen Complex. *Organometallics* **2013**, *32* (15), 4391–4401.

(2) Li, P.; Liu, Y.; Wang, L.; Xiao, J.; Tao, M., Copper(II)-Schiff Base Complex-Functionalized Polyacrylonitrile Fiber as a Green Efficient Heterogeneous Catalyst for One-Pot Multicomponent Syntheses of 1,2,3-Triazoles and Propargylamines. *Adv. Synth. Catal.* **2018**, *360* (8), 1673–1684.

(3) Broere, D. L. J.; Čorić, I.; Brosnahan, A.; Holland, P. L., Quantitation of the THF Content in $Fe[N(SiMe_3)_2]_2 \cdot x$THF. *Inorg. Chem.* **2017**, *56* (6), 3140–3143.

(4) Johnson, S. A.; Kiernicki, J. J.; Fanwick, P. E.; Bart, S. C., New Benzylpotassium Reagents and Their Utility for the Synthesis of Homoleptic Uranium(IV) Benzyl Derivatives. *Organometallics* **2015**, *34* (12), 2889–2895.

(5) Hawkeswood, S.; Stephan, D. W., Syntheses and reactions of the bis-boryloxide $O(Bpin)_2$ (pin = $O_2C_2Me_4$). *Dalton Trans.* **2005**, (12), 2182–2187.

(6) Pécharman, A.-F.; Colebatch, A. L.; Hill, M. S.; McMullin, C. L.; Mahon, M. F.; Weetman, C., Easy access to nucleophilic boron through diborane to magnesium boryl metathesis. *Nat. Commun.* **2017**, *8* (1), 15 022.

(7) Gallagher, K. J.; Espinal-Viguri, M.; Mahon, M. F.; Webster, R. L., A Study of Two Highly Active, Air-Stable Iron(III)-μ-Oxo Precatalysts: Synthetic Scope of Hydrophosphination using Phenyl- and Diphenylphosphine. *Adv. Synth. Catal.* **2016**, *358* (15), 2460–2468.

(8) Sarac, K.; Orek, C.; Cetin, A.; Dastan, T.; Koparir, P.; Dastan, S. D.; Koparir, M., Synthesis and in vitro antioxidant evaluation of new bis(α-aminoalkyl)phosphinic acid derivatives. *Phosphorus, Sulfur, Silicon Relat. Elem.* **2016**, *191* (9), 1284–1289.

(9) You, Z.-L.; Lu, Y.; Zhang, N.; Ding, B.-W.; Sun, H.; Hou, P.; Wang, C., Preparation and structural characterization of hetero-dinuclear Schiff base copper(II)-zinc(II) complexes and their inhibition studies on Helicobacter pylori urease. *Polyhedron* **2011**, *30* (13), 2186–2194.

(10) Chapman, M. R.; Henkelis, S. E.; Kapur, N.; Nguyen, B. N.; Willans, C. E., A Straightforward Electrochemical Approach to Imine- and Amine-bisphenolate Metal Complexes with Facile Control Over Metal Oxidation State. *ChemistryOpen* **2016**, *5* (4), 351–356.

(11) Würtenberger, I.; Follia, V.; Lerch, F.; Cwikla, C.; Fahrner, N.; Kalchschmidt, C.; Flögel, B.; Kircher, B.; Gust, R., Fluorinated Fe(III) Salophene Complexes: Optimization of Tumor Cell Specific Activity and Utilization of Fluorine Labeling for in Vitro Analysis. *J. Med. Chem.* **2015**, *58* (2), 588–597.

(12) Bhunia, A.; Gotthardt, M. A.; Yadav, M.; Gamer, M. T.; Eichhöfer, A.; Kleist, W.; Roesky, P. W., Salen-Based Coordination Polymers of Manganese and the Rare-Earth Elements: Synthesis and Catalytic Aerobic Epoxidation of Olefins. *Chem. Eur. J.* **2013**, *19* (6), 1986–1995.

(13) Kasumov, V. T.; Köksal, F., Synthesis, spectroscopy, and electrochemistry of copper(II) complexes with N,N'-bis(3,5-di-t-butylsalicylideneimine)polymethylenediamine ligands. *Spectrochim. Acta Part A* **2005**, *61* (1), 225–231.

(14) Kirk, S. M.; Kociok-Köhn, G.; Jones, M. D., Zirconium vs Aluminum Salalen Initiators for the Production of Biopolymers. *Organometallics* **2016**, *35* (22), 3837–3843.

(15) Elmali, A.; Atakol, O.; Svoboda, I.; Fuess, H., Crystal structure of μ-(N,N'-1,3-propyl-disalicylaldiminato)-bis(N,N-1,3-propyl-disalicylaldiminato)-di-iron(III) ethanol solvate, $(OC_6H_4CHN)_2C_3H_6(Fe(OC_6H_4CHN)_2C_3H_6)_2(C_2H_5OH)$. *Z. Kristallogr. – Cryst. Mater.* **1993**, *203* (1-2), 271–274.

(16) Higher order **$[Fe_2(1c)_3]$** also observed in spectrum by LIFDI-MS.

(17) Negoro, S.; Asada, H.; Fujiwara, M.; Matsushita, T., Preparation and characterization of three types of dinuclear iron(III) complexes with H_2salbn, N,N'-disalicylidene-1,4-diaminobutane. *Inorg. Chem. Commun.* **2003**, *6* (4), 357–360.

(18) Provis-Evans, C. B.; Lau, S.; Krewald, V.; Webster, R. L., Regioselective Alkyne Cyclotrimerization with an In Situ-Generated [Fe(II)H(salen)]•Bpin Catalyst. *ACS Catal.* **2020**, *10* (17), 10 157–10 168.

(19) Edulji, S. K.; Nguyen, S. T., Catalytic Olefin Cyclopropanation Using μ-Oxo-bis[(salen)iron(III)] Complexes. *Organometallics* **2003**, *22* (17), 3374–3381.

(20) Angaridis, P.; Kampf, J. W.; Pecoraro, V. L., Multinuclear Fe(III) Complexes with Polydentate Ligands of the Family of Dicarboxyimidazoles: Nuclearity- and Topology-Controlled Syntheses and Magneto-Structural Correlations. *Inorg. Chem.* **2005**, *44* (10), 3626–3635.

(21) Borer, L.; Thalken, L.; Ceccarelli, C.; Glick, M.; Zhang, J. H.; Reiff, W. M., Synthesis and characterization of a hydroxyl-bridged iron(III) dimer of N,N'-ethylenebis(salicylamine). *Inorg. Chem.* **1983**, *22* (12), 1719–1724.

(22) Braunschweig, H.; Guethlein, F.; Mailänder, L.; Marder, T. B., Synthesis of Catechol-, Pinacol-, and Neopentylglycolborane through the Heterogeneous Catalytic B–B Hydrogenolysis of Diboranes(4). *Chem. Eur. J.* **2013**, *19* (44), 14 831–14 835.

(23) Wei, C. S.; Jiménez-Hoyos, C. A.; Videa, M. F.; Hartwig, J. F.; Hall, M. B., Origins of the Selectivity for Borylation of Primary over Secondary C–H Bonds Catalyzed by Cp*-Rhodium Complexes. *J. Am. Chem. Soc.* **2010**, *132* (9), 3078–3091.

(24) Dove, A. P.; Gibson, V. C.; Hormnirun, P.; Marshall, E. L.; Segal, J. A.; White, A. J. P.; Williams, D. J., Low coordinate magnesium chemistry supported by a bulky β-diketiminate ligand. *Dalton Trans.* **2003**, (15), 3088–3097.

(25) Sheldrick, G. M., *SHELXT* — Integrated space-group and crystal-structure determination. *Acta Cryst. A* **2015**, *71* (1), 3–8.

(26) Sheldrick, G. M., Crystal structure refinement with *SHELXL*. *Acta Cryst. C* **2015**, *71* (1), 3–8.

(27) Dolomanov, O. V.; Bourhis, L. J.; Gildea, R. J.; Howard, J. A. K.; Puschmann, H., OLEX2: a complete structure solution, refinement and analysis program. *J. Appl. Crystallogr.* **2009**, *42* (2), 339–341.

(28) Gaussian 16, Revision C.01. Frisch, M. J.; Trucks, G. W.; Schlegel, H. B.; Scuseria, G. E.; Robb, M. A.; Cheeseman, J. R.; Scalmani, G.; Barone, V.; Petersson, G. A.; Nakatsuji, H.; Li, X.; Caricato, M.; Marenich, A. V.; Bloino, J.; Janesko, B. G.; Gomperts, R.; Mennucci, B.; Hratchian, H. P.; Ortiz, J. V.; Izmaylov, A. F.; Sonnenberg, J. L.; Williams-Young, D.; Ding, F.; Lipparini, F.; Egidi, F.; Goings, J.; Peng, B.; Petrone, A.; Henderson, T.; Ranasinghe, D.; Zakrzewski, V. G.; Gao, J.; Rega, N.; Zheng, G.; Liang, W.; Hada, M.; Ehara, M.; Toyota, K.; Fukuda, R.; Hasegawa, J.; Ishida, M.; Nakajima, T.; Honda, Y.; Kitao, O.; Nakai, H.; Vreven, T.; Throssell, K.; Montgomery, Jr., J. A.; Peralta, J. E.; Ogliaro, F.; Bearpark, M. J.; Heyd, J. J.; Brothers, E. N.; Kudin, K. N.; Staroverov, V. N.; Keith, T. A.; Kobayashi, R.; Normand, J.; Raghavachari, K.; Rendell, A. P.; Burant, J. C.; Iyengar, S. S.; Tomasi, J.; Cossi, M.; Millam, J. M.; Klene, M.; Adamo, C.; Cammi, R.; Ochterski, J. W.; Martin, R. L.; Morokuma, K.; Farkas, Ö.; Foresman, J. B.; Fox, D. J. (Gaussian, Inc., Wallingford, CT), **2019**, see `http://www.gaussian.com`.

(29) Neese, F., Software update: The ORCA program system – Version 5.0. *WIREs Comput. Mol. Sci.* **2022**, *12* (5), e1606.

(30) ORCA version 5.0.4, an *ab initio*, DFT and semiempirical SCF-MO package. Neese, F.; *Technical Directorship* F. Wennmohs; *with contributions from* D. Aravena; Atanasov, M.; Auer, A. A.; Becker, U.; Bistoni, G.; Bykov, D.; Chilkuri, V. G.; Datta, D.; Dutta, A. K.; Ehlert, S.; Ganyushin, D.; Garcia, M.; Guo, Y.; Hansen, A.; Helmich-Paris, B.; Huntington, L.; Izsák, R.; Kettner, M.; Kollmar, C.; Kossmann, S.; Krupička, M.; Lang, L.; Lechner, M.; Lenk, D.; Liakos, D. G.; Manganas, D.; Pantazis, D. A.; Papadopoulos, A.; Petrenko, T.; Pinski, P.; Pracht, P.; Reimann, C.; Retegan, M.; Riplinger, C.; Risthaus, T.; Roemelt, M.; Saitow, M.; Sandhöfer, B.; Schapiro, I.; Sen, A.; Sivalingam, K.; de Souza, B.; Stoychev, G.; Van den Heuvel, W.; Wezisla, B.; *and with contributions from collaborators* M. Kállay; Grimme, S.; Valeev, E.; Chan, G.; Pittner, J.; Brehm, M.; Goerigk, L.; Åsgeirsson, V.; Ungur, L. (Max-Planck-Institut für Kohlenforschung, Mülheim a. d. Ruhr, Germany), **2023**, see `https://orcaforum.kofo.mpg.de/`.

(31) Perdew, J. P.; Burke, K.; Ernzerhof, M., Generalized Gradient Approximation Made Simple. *Phys. Rev. Lett.* **1996**, *77* (18), 3865–3868.

(32) Perdew, J. P.; Burke, K.; Ernzerhof, M., Erratum: Generalized Gradient Approximation Made Simple [Phys. Rev. Lett. 77, 3865 (1996)]. *Phys. Rev. Lett.* **1997**, *78* (7), 1396–1396.

(33) Grimme, S.; Antony, J.; Ehrlich, S.; Krieg, H., A consistent and accurate *ab initio* parametrization of density functional dispersion correction (DFT-D) for the 94 elements H–Pu. *J. Chem. Phys.* **2010**, *132* (15), 154 104.

(34) Tomasi, J.; Mennucci, B.; Cammi, R., Quantum Mechanical Continuum Solvation Models. *Chem. Rev.* **2005**, *105* (8), 2999–3094.

(35) Weigend, F.; Ahlrichs, R., Balanced basis sets of split valence, triple zeta valence and quadruple zeta valence quality for H to Rn: Design and assessment of accuracy. *Phys. Chem. Chem. Phys.* **2005**, *7* (18), 3297–3305.

(36) Weigend, F., Accurate Coulomb-fitting basis sets for H to Rn. *Phys. Chem. Chem. Phys.* **2006**, *8* (9), 1057–1065.

(37) Perdew, J. P.; Ernzerhof, M.; Burke, K., Rationale for mixing exact exchange with density functional approximations. *J. Chem. Phys.* **1996**, *105* (22), 9982–9985.

(38) Adamo, C.; Barone, V., Toward reliable density functional methods without adjustable parameters: The PBE0 model. *J. Chem. Phys.* **1999**, *110* (13), 6158–6170.

(39) Riplinger, C.; Pinski, P.; Becker, U.; Valeev, E. F.; Neese, F., Sparse maps — A systematic infrastructure for reduced-scaling electronic structure methods. II. Linear scaling domain based pair natural orbital coupled cluster theory. *J. Chem. Phys.* **2016**, *144* (2), 024 109.

(40) Hellweg, A.; Hättig, C.; Höfener, S.; Klopper, W., Optimized accurate auxiliary basis sets for RI-MP2 and RI-CC2 calculations for the atoms Rb to Rn. *Theor. Chem. Acc.* **2007**, *117* (4), 587–597.

(41) Neese, F.; Wennmohs, F.; Hansen, A.; Becker, U., Efficient, approximate and parallel Hartree–Fock and hybrid DFT calculations. A 'chain-of-spheres' algorithm for the Hartree–Fock exchange. *Chem. Phys.* **2009**, *356* (1–3), 98–109.

(42) Neese, F.; Valeev, E. F., Revisiting the Atomic Natural Orbital Approach for Basis Sets: Robust Systematic Basis Sets for Explicitly Correlated and Conventional Correlated *ab initio* Methods?. *J. Chem. Theory Comput.* **2011**, *7* (1), 33–43.

(43) Altun, A.; Neese, F.; Bistoni, G., Extrapolation to the Limit of a Complete Pair Natural Orbital Space in Local Coupled-Cluster Calculations. *J. Chem. Theory Comput.* **2020**, *16* (10), 6142–6149.

(44) Okuniewski, A.; Rosiak, D.; Chojnacki, J.; Becker, B., Coordination polymers and molecular structures among complexes of mercury(II) halides with selected 1-benzoylthioureas. *Polyhedron* **2015**, *90*, 47–57.

A. Appendix

Cartesian coordinates of optimized geometries (Å)

35

^{5}Fe^{II}(salen) (^{5}B, C_2): E_{tot}(UPBE-D3(PCM=Acetonitrile)/def2-SVP) = -2139.85867752 $\langle S^2 \rangle$ = 6.0832

Fe	0.000000000000	0.000000000000	0.073145577311
O	0.064760102313	1.553902154977	1.228282850705
O	-0.064760102313	-1.553902154977	1.228282850705
N	0.283280476450	-1.307060519211	-1.527405648381
N	-0.283280476450	1.307060519211	-1.527405648381
C	-0.300888391162	2.615405363520	-1.490601388792
H	-0.443932959216	3.173713312771	-2.442712128343
C	-0.139897459748	3.421794225793	-0.306084429362
C	-0.158194745886	4.838799074217	-0.446731807183
H	-0.301375710022	5.258759685771	-1.456261213947
C	0.000000000000	5.688606411302	0.644676171686
H	-0.017278676025	6.780213676818	0.510272888546
C	0.184586174999	5.125992686568	1.931820777305
H	0.310635416070	5.786658538827	2.804528016220
C	0.207743816653	3.744395988754	2.109399135607
H	0.349184384766	3.299705491799	3.106457273230
C	0.046378641276	2.842098701533	1.011143358238
C	-0.046378641276	-2.842098701533	1.011143358238
C	-0.207743816653	-3.744395988754	2.109399135607
H	-0.349184384766	-3.299705491799	3.106457273230
C	-0.184586174999	-5.125992686568	1.931820777305
H	-0.310635416070	-5.786658538827	2.804528016220
C	0.000000000000	-5.688606411302	0.644676171686
H	0.017278676025	-6.780213676818	0.510272888546
C	0.158194745886	-4.838799074217	-0.446731807183
H	0.301375710022	-5.258759685771	-1.456261213947
C	0.139897459748	-3.421794225793	-0.306084429362
C	0.300888391162	-2.615405363520	-1.490601388792
H	0.443932959216	-3.173713312771	-2.442712128343
C	0.458397050191	-0.614029445480	-2.796880173151
H	0.246273628431	-1.270487598721	-3.669810848907
H	1.515176558006	-0.273507737203	-2.878239624915
C	-0.458397050191	0.614029445480	-2.796880173151
H	-0.246273628431	1.270487598721	-3.669810848907
H	-1.515176558006	0.273507737203	-2.878239624915

35

^{3}Fe^{II}(salen) (^{3}A, C_2): E_{tot}(UPBE-D3(PCM=Acetonitrile)/def2-SVP) = -2139.87515212 $\langle S^2 \rangle$ = 2.0562

Fe	0.000000000000	0.000000000000	0.225534357033
O	0.000000000000	1.298165354407	-1.155186280352
O	0.000000000000	-1.298165354407	-1.155186280352
N	-0.157641072886	-1.270548628033	1.607209732095
N	0.157641072886	1.270548628033	1.607209732095
C	0.171481336796	2.582574918097	1.481273595423
H	0.277458503243	3.190247107765	2.402311911018
C	0.066169274494	3.293324946298	0.241532036317
C	0.052464581559	4.719584402833	0.263555292723
H	0.117253227917	5.225654704780	1.240792620307
C	-0.040021038935	5.467083438245	-0.904933633392
H	-0.050130353377	6.566311843112	-0.865551178232
C	-0.120126390964	4.792536947893	-2.150714529838
H	-0.193063125206	5.374440236133	-3.083355047242
C	-0.106584040063	3.402384139079	-2.210597147858
H	-0.166842655835	2.870751861329	-3.172563045694
C	-0.013227800262	2.599353315333	-1.029322366403
C	0.013227800262	-2.599353315333	-1.029322366403
C	0.106584040063	-3.402384139079	-2.210597147858
H	0.166842655835	-2.870751861329	-3.172563045694
C	0.120126390964	-4.792536947893	-2.150714529838
H	0.193063125206	-5.374440236133	-3.083355047242
C	0.040021038935	-5.467083438245	-0.904933633392
H	0.050130353377	-6.566311843112	-0.865551178232
C	-0.052464581559	-4.719584402833	0.263555292723
H	-0.117253227917	-5.225654704780	1.240792620307
C	-0.066169274494	-3.293324946298	0.241532036317
C	-0.171481336796	-2.582574918097	1.481273595423
H	-0.277458503243	-3.190247107765	2.402311911018

C	-0.370916497985	-0.664932010661	2.929483800500
H	-0.027677448675	-1.330422144616	3.749322725740
H	-1.460694426422	-0.488961573167	3.066455206005
C	0.370916497985	0.664932010661	2.929483800500
H	0.027677448675	1.330422144616	3.749322725740
H	1.460694426422	0.488961573167	3.066455206005

35

^{1}Fe^{II}(salen) (^{1}A, C_2): E_{tot}(RPBE-D3(PCM=Acetonitrile)/def2-SVP) = -2139.84639286 $\langle S^2 \rangle$ = 0.0000

Fe	0.000000000000	0.000000000000	0.187419511151
O	-0.246477573802	1.329324184407	-1.051904988374
O	0.246477573802	-1.329324184407	-1.051904988374
N	-0.482475606721	-1.165446923010	1.513381724898
N	0.482475606721	1.165446923010	1.513381724898
C	0.688353666540	2.475559816869	1.427786655710
H	0.984138468088	3.016810410088	2.346319318706
C	0.506202852191	3.239787924185	0.233932994093
C	0.752377337171	4.643757338664	0.233146274676
H	1.135048743276	5.110021388702	1.155272921686
C	0.514130548304	5.419110245107	-0.897337927514
H	0.716101497262	6.500474733333	-0.878789786501
C	0.000000000000	4.811706891474	-2.071935569959
H	-0.191330282998	5.424176591085	-2.966737074235
C	-0.266923503868	3.445091294385	-2.101243745553
H	-0.667680328342	2.956145183135	-3.001976770639
C	-0.004841962754	2.619617839797	-0.968950000819
C	0.004841962754	-2.619617839797	-0.968950000819
C	0.266923503868	-3.445091294385	-2.101243745553
H	0.667680328342	-2.956145183135	-3.001976770639
C	0.000000000000	-4.811706891474	-2.071935569959
H	0.191330282998	-5.424176591085	-2.966737074235
C	-0.514130548304	-5.419110245107	-0.897337927514
H	-0.716101497262	-6.500474733333	-0.878789786501
C	-0.752377337171	-4.643757338664	0.233146274676
H	-1.135048743276	-5.110021388702	1.155272921686
C	-0.506202852191	-3.239787924185	0.233932994093
C	-0.688353666540	-2.475559816869	1.427786655710
H	-0.984138468088	-3.016810410088	2.346319318706
C	-0.558964467930	-0.519243255389	2.833446221867
H	-0.450826160993	-1.255162655433	3.657261166733
H	-1.544102227499	-0.016123004720	2.940694996983
C	0.558964467930	0.519243255389	2.833446221867
H	0.450826160993	1.255162655433	3.657261166733
H	1.544102227499	0.016123004720	2.940694996983

57

^{1}A_BP86/ZORA E_{tot} = -2568.83257374 $\langle S^2 \rangle$ = 0.0000

Fe	4.961977	4.582795	3.374773
O	2.804197	5.572276	6.262444
C	7.459436	3.440852	2.777683
H	8.233244	2.686555	2.988576
O	5.666383	5.667411	2.082783
C	7.708010	4.381383	1.711200
N	4.438684	3.950163	5.259070
C	8.912125	4.266749	0.958739
H	9.603081	3.448528	1.202459
N	6.379025	3.432509	3.529495
C	9.221977	5.162067	-0.066122
H	10.152985	5.050667	-0.634601
C	8.324400	6.216064	-0.362863
H	8.558612	6.925406	-1.167144
C	7.136171	6.363587	0.358784
H	6.435006	7.178245	0.139784
C	6.797810	5.457691	1.398418
C	6.197518	2.362180	4.549449
H	7.170346	1.945027	4.863158
H	5.612841	1.553289	4.073571
C	5.452953	2.970875	5.739199
H	4.961937	2.192820	6.350530
H	6.142137	3.538536	6.382302
C	3.457359	3.526141	4.375273
C	2.199213	4.275643	4.304258
C	1.227424	3.977786	3.316781
H	1.455340	3.200114	2.575814
C	0.002667	4.654287	3.286709
H	-0.740120	4.416701	2.515743

C	-0.271009	5.639890	4.261722
H	-1.230380	6.172776	4.247950
C	0.673950	5.948169	5.250974
H	0.471500	6.707717	6.016072
C	1.919788	5.283198	5.277953
H	3.431873	2.459345	4.110503
B	4.254248	5.373348	6.017197
O	4.768699	6.407038	5.081618
O	5.027526	5.448825	7.218987
C	5.947020	7.022628	5.698259
C	5.688582	6.748658	7.231717
C	4.728119	7.775036	7.858125
C	6.961448	6.635135	8.072816
C	5.973533	8.501033	5.305651
C	7.210356	6.322523	5.176074
H	6.174563	8.594055	4.223574
H	5.011409	8.993096	5.521448
H	7.214963	6.365346	4.073561
H	5.208303	8.762605	7.973410
H	3.816902	7.890781	7.246843
H	7.233819	5.261712	5.474411
H	8.126640	6.813669	5.546710
H	6.774162	9.031405	5.852206
H	4.429173	7.416983	8.858955
H	7.555561	7.563961	8.002470
H	7.586361	5.789102	7.742955
H	6.696460	6.475610	9.133131
H	4.461717	3.648713	2.340996

57

[1]A_PBE/ZORA E_{tot} = -2566.75680952 $\langle S^2 \rangle$ = 0.0000

Fe	4.982539	4.596752	3.367313
O	2.820315	5.591876	6.231355
C	7.448793	3.411552	2.739591
H	8.212245	2.644374	2.941865
O	5.692744	5.669466	2.067165
C	7.697227	4.340534	1.665380
N	4.457915	3.968716	5.240053
C	8.884263	4.196292	0.893228
H	9.559711	3.363146	1.129353
N	6.381494	3.425910	3.509906
C	9.196339	5.080251	-0.139066
H	10.114351	4.945905	-0.723354
C	8.318863	6.152839	-0.423020
H	8.555009	6.854342	-1.233519
C	7.148242	6.329833	0.318228
H	6.463024	7.160202	0.107901
C	6.806858	5.435347	1.365852
C	6.202825	2.365988	4.537639
H	7.178330	1.954805	4.851156
H	5.621169	1.547295	4.073762
C	5.462298	2.982807	5.723059
H	4.963684	2.208923	6.334128
H	6.154723	3.543029	6.369635
C	3.481583	3.549220	4.346354
C	2.216698	4.287023	4.281293
C	1.239409	3.976129	3.304472
H	1.466502	3.196595	2.565118
C	0.008506	4.639254	3.282463
H	-0.738548	4.390614	2.519255
C	-0.266694	5.624358	4.255626
H	-1.231590	6.147228	4.248601
C	0.682138	5.944203	5.235274
H	0.477026	6.703230	6.000241
C	1.934852	5.292453	5.254514
H	3.460719	2.482694	4.080044
B	4.273644	5.386022	6.003896
O	4.809204	6.423186	5.087824
O	5.025899	5.450333	7.219832
C	5.954163	7.059525	5.740054
C	5.673976	6.753614	7.265594
C	4.701215	7.761648	7.900540
C	6.933101	6.634967	8.125336
C	5.947082	8.544160	5.372729
C	7.248894	6.409772	5.232059
H	6.154315	8.660304	4.294118
H	4.972377	9.010199	5.589040

H	7.277646	6.477335	4.131039
H	5.173847	8.749854	8.037182
H	3.792774	7.885746	7.287276
H	7.306568	5.344985	5.512570
H	8.140486	6.923315	5.631131
H	6.730340	9.086770	5.931864
H	4.397920	7.386076	8.893468
H	7.512933	7.574763	8.098858
H	7.580576	5.811579	7.782643
H	6.650251	6.437645	9.174438
H	4.467250	3.673722	2.330193

57

^{1}A_BP86/SVP E_{tot}(RBP86-D3(Acetonitrile)/def2SVP/W06) = -2552.98439387 $\langle S^2 \rangle$ = 0.0000

Fe	0.458907	-0.884113	0.210847
O	-3.067758	0.230849	-0.065962
C	2.867329	-0.347008	1.568211
H	3.395591	-0.082739	2.503516
O	1.757105	-0.897175	-1.089365
C	3.664609	-0.532447	0.380393
N	-1.081918	-0.201046	1.403977
C	5.081556	-0.412258	0.471007
H	5.527959	-0.197344	1.456025
N	1.550873	-0.452062	1.618449
C	5.901219	-0.561338	-0.648504
H	6.993743	-0.468508	-0.553260
C	5.312406	-0.831256	-1.908053
H	5.950725	-0.951158	-2.798256
C	3.925883	-0.949579	-2.036433
H	3.456379	-1.154720	-3.010783
C	3.068777	-0.812340	-0.908712
C	0.859139	-0.327859	2.926518
H	1.453360	0.278693	3.640539
H	0.763049	-1.349531	3.353167
C	-0.510945	0.306721	2.676975
H	-1.210285	0.104098	3.514707
H	-0.428315	1.402404	2.556234
C	-1.264368	-1.566422	1.236009
C	-2.289795	-2.034743	0.302017
C	-2.457697	-3.415518	0.031924
H	-1.769693	-4.132396	0.509128
C	-3.473337	-3.864531	-0.819627
H	-3.590951	-4.939716	-1.023858
C	-4.349573	-2.925577	-1.409590
H	-5.154281	-3.269535	-2.078929
C	-4.204865	-1.554886	-1.154707
H	-4.881413	-0.812451	-1.605120
C	-3.170607	-1.086447	-0.310835
H	-1.011512	-2.226852	2.084179
B	-1.783620	0.828432	0.365282
O	-0.852161	0.940658	-0.786208
O	-1.927912	2.133562	0.924097
C	-0.374745	2.311168	-0.857473
C	-1.512685	3.091354	-0.077279
C	-2.722070	3.412711	-0.975746
C	-1.031972	4.360426	0.630013
C	-0.225022	2.694427	-2.331649
C	0.990493	2.397640	-0.156955
H	0.598896	2.107433	-2.786423
H	-1.149964	2.482874	-2.900529
H	1.673551	1.654158	-0.613396
H	-2.492394	4.210733	-1.710261
H	-3.062015	2.509509	-1.519656
H	0.904258	2.157845	0.920234
H	1.445311	3.402847	-0.260575
H	0.020444	3.771400	-2.435685
H	-3.557683	3.758742	-0.334216
H	-0.594691	5.075062	-0.097111
H	-0.272974	4.130450	1.402073
H	-1.887269	4.859183	1.130020
H	0.807424	-2.280528	0.567299

57

^{1}A_BP86/TZVP E_{tot}(RBP86-D3(Acetonitrile)/def2TZVP/W06) = -2554.55441041 $\langle S^2 \rangle$ = 0.0000

Fe	0.431515	-0.944349	0.221872
O	-3.041390	0.271460	-0.102305

C	2.842234	-0.379576	1.558249
H	3.369949	-0.105682	2.479211
O	1.725859	-0.989390	-1.079883
C	3.628712	-0.568046	0.371461
N	-1.078046	-0.195095	1.389249
C	5.036047	-0.418080	0.452192
H	5.478512	-0.184438	1.423907
N	1.535508	-0.490040	1.616558
C	5.847633	-0.560100	-0.663551
H	6.928501	-0.443344	-0.576846
C	5.260305	-0.853214	-1.910394
H	5.890301	-0.966649	-2.795116
C	3.884006	-1.002831	-2.028815
H	3.420542	-1.226352	-2.991269
C	3.036889	-0.872191	-0.903231
C	0.849441	-0.348952	2.924877
H	1.448815	0.252276	3.623549
H	0.738681	-1.358892	3.349161
C	-0.506650	0.303796	2.668818
H	-1.209630	0.107954	3.491513
H	-0.403794	1.387862	2.551343
C	-1.311296	-1.549565	1.247304
C	-2.325106	-2.001630	0.302921
C	-2.518976	-3.375022	0.050149
H	-1.864447	-4.094736	0.547704
C	-3.520001	-3.808761	-0.812839
H	-3.658463	-4.873854	-1.003005
C	-4.354088	-2.862267	-1.433650
H	-5.143919	-3.193863	-2.110570
C	-4.183682	-1.499248	-1.195716
H	-4.827997	-0.756516	-1.669342
C	-3.164094	-1.049057	-0.338260
H	-1.086145	-2.195598	2.100305
B	-1.752277	0.849561	0.347472
O	-0.804025	0.966805	-0.787686
O	-1.899753	2.155875	0.915652
C	-0.312027	2.336993	-0.833965
C	-1.452132	3.118664	-0.075132
C	-2.639150	3.455184	-0.990204
C	-0.975527	4.374220	0.649053
C	-0.130444	2.732604	-2.297165
C	1.037563	2.401702	-0.110712
H	0.690468	2.148060	-2.737346
H	-1.040307	2.540574	-2.879064
H	1.715056	1.661688	-0.559686
H	-2.381835	4.239973	-1.716020
H	-2.980434	2.563690	-1.533723
H	0.931596	2.157654	0.953982
H	1.497042	3.396127	-0.200355
H	0.125326	3.799487	-2.381042
H	-3.470430	3.817521	-0.368756
H	-0.523288	5.082720	-0.060753
H	-0.237154	4.132379	1.424169
H	-1.830030	4.871570	1.130527
H	0.739372	-2.344137	0.606275

57

^{1}A_PBE/SVP E_{tot}(RPBE-D3(Acetonitrile)/def2SVP/W06) = -2550.94482626 $\langle S^2 \rangle$ = 0.0000

Fe	0.500372	-0.850670	0.194863
O	-3.059271	0.135063	-0.054000
C	2.896995	-0.304770	1.564306
H	3.419797	-0.046701	2.503730
O	1.804214	-0.822083	-1.101703
C	3.699668	-0.470658	0.380055
N	-1.050325	-0.230739	1.395120
C	5.113705	-0.345454	0.480284
H	5.551896	-0.144555	1.471102
N	1.582062	-0.420510	1.609924
C	5.939144	-0.472367	-0.635322
H	7.030000	-0.376615	-0.532586
C	5.359034	-0.722942	-1.900661
H	6.002193	-0.825085	-2.788725
C	3.975610	-0.844772	-2.038559
H	3.513307	-1.035496	-3.018570
C	3.112144	-0.731058	-0.914827
C	0.889886	-0.314263	2.915854
H	1.469766	0.309976	3.625474

H	0.824137	-1.335110	3.349918
C	-0.495964	0.280243	2.670795
H	-1.187979	0.047591	3.506488
H	-0.448801	1.379396	2.565102
C	-1.175827	-1.600474	1.208583
C	-2.198970	-2.099896	0.289049
C	-2.328426	-3.483238	0.019244
H	-1.610896	-4.179163	0.482441
C	-3.344063	-3.962912	-0.812421
H	-3.430969	-5.040393	-1.016070
C	-4.260436	-3.053147	-1.381870
H	-5.066294	-3.421434	-2.035577
C	-4.155709	-1.681258	-1.125699
H	-4.865218	-0.960905	-1.559535
C	-3.121846	-1.181101	-0.301583
H	-0.894562	-2.257286	2.049985
B	-1.797183	0.779879	0.375395
O	-0.881125	0.946595	-0.779239
O	-1.994403	2.071675	0.947442
C	-0.495250	2.341231	-0.868219
C	-1.653977	3.055105	-0.052888
C	-2.897163	3.327048	-0.917833
C	-1.225407	4.340811	0.654698
C	-0.418551	2.725225	-2.346660
C	0.884423	2.519768	-0.218935
H	0.417178	2.179467	-2.828901
H	-1.350180	2.464920	-2.881977
H	1.599058	1.823315	-0.699275
H	-2.722230	4.139255	-1.650506
H	-3.213552	2.416529	-1.462099
H	0.858613	2.289112	0.863235
H	1.267129	3.551270	-0.345684
H	-0.231519	3.811498	-2.465551
H	-3.730204	3.632789	-0.254172
H	-0.859784	5.091647	-0.074180
H	-0.427274	4.150629	1.396330
H	-2.091236	4.779026	1.190234
H	0.882273	-2.243625	0.526313

57

^{1}A_PBE/TZVP E_{tot}(RPBE-D3(Acetonitrile)/def2TZVP/W06) = -2552.50015182 $\langle S^2 \rangle$ = 0.0000

Fe	0.484897	-0.901480	0.202853
O	-3.026711	0.144271	-0.115904
C	2.877133	-0.310382	1.552503
H	3.397013	-0.035090	2.477359
O	1.784821	-0.904674	-1.095130
C	3.671806	-0.485514	0.371488
N	-1.044906	-0.223962	1.373183
C	5.076222	-0.329429	0.465199
H	5.507743	-0.102415	1.443105
N	1.571811	-0.435685	1.604255
C	5.897197	-0.457801	-0.643515
H	6.976541	-0.337440	-0.546587
C	5.322026	-0.741629	-1.896410
H	5.959574	-0.844601	-2.776707
C	3.948744	-0.895868	-2.027668
H	3.495474	-1.113037	-2.996260
C	3.091839	-0.780184	-0.909083
C	0.882960	-0.309012	2.909376
H	1.462361	0.320638	3.599574
H	0.810991	-1.316163	3.349153
C	-0.494837	0.290626	2.652924
H	-1.189633	0.063742	3.474876
H	-0.435594	1.378978	2.544657
C	-1.206334	-1.588934	1.223253
C	-2.214764	-2.090960	0.299309
C	-2.361373	-3.472334	0.065606
H	-1.674565	-4.162021	0.562001
C	-3.358012	-3.953594	-0.774821
H	-3.459612	-5.025228	-0.949462
C	-4.235972	-3.047594	-1.392062
H	-5.023582	-3.416440	-2.051758
C	-4.113952	-1.678208	-1.171712
H	-4.794749	-0.966588	-1.641866
C	-3.099406	-1.179362	-0.336184
H	-0.943334	-2.222982	2.074221
B	-1.773717	0.791012	0.344830

O	-0.832724	0.982403	-0.783412
O	-2.004202	2.079413	0.925595
C	-0.455154	2.384903	-0.841031
C	-1.636740	3.078538	-0.057632
C	-2.852893	3.351763	-0.952979
C	-1.240287	4.355456	0.675593
C	-0.338280	2.788421	-2.308030
C	0.901561	2.566284	-0.154185
H	0.508322	2.258971	-2.768198
H	-1.247021	2.535515	-2.867319
H	1.631880	1.892813	-0.623836
H	-2.652032	4.157580	-1.672850
H	-3.151396	2.450327	-1.504134
H	0.851124	2.322315	0.914772
H	1.267960	3.597089	-0.257401
H	-0.155097	3.868775	-2.402540
H	-3.695391	3.660178	-0.318209
H	-0.864670	5.110299	-0.030357
H	-0.465737	4.163448	1.428329
H	-2.118961	4.775733	1.185249
H	0.836932	-2.295862	0.564956

57

^{1}A_PBE0/SVP E_{tot}(RPBE0-D3(Acetonitrile)/def2SVP/W06) = -2551.01462500 $\langle S^2 \rangle$ = 0.0000

Fe	0.477800	-0.878482	0.144269
O	-3.063035	0.163395	0.006235
C	2.885701	-0.344470	1.557274
H	3.417610	-0.109514	2.490645
O	1.837290	-0.847111	-1.121380
C	3.690857	-0.478872	0.372411
N	-1.022614	-0.243211	1.360664
C	5.090884	-0.333758	0.498214
H	5.506259	-0.137047	1.491030
N	1.599837	-0.473125	1.607250
C	5.931077	-0.435644	-0.594511
H	7.010700	-0.324022	-0.477795
C	5.370481	-0.683341	-1.860388
H	6.022290	-0.765523	-2.734623
C	4.003645	-0.825500	-2.019616
H	3.564461	-1.013938	-3.001793
C	3.118483	-0.733581	-0.917086
C	0.898058	-0.386896	2.885753
H	1.467010	0.193520	3.628182
H	0.789687	-1.410612	3.281956
C	-0.460390	0.246062	2.629991
H	-1.160128	0.038746	3.454319
H	-0.375714	1.334478	2.529208
C	-1.172233	-1.588758	1.174718
C	-2.215394	-2.062932	0.263435
C	-2.352146	-3.428246	-0.031630
H	-1.627334	-4.130776	0.389280
C	-3.385383	-3.883228	-0.838829
H	-3.480549	-4.947110	-1.065423
C	-4.307982	-2.962457	-1.353505
H	-5.128402	-3.309565	-1.986990
C	-4.192205	-1.607447	-1.069828
H	-4.906325	-0.880672	-1.462991
C	-3.140112	-1.133920	-0.269596
H	-0.912343	-2.249068	2.008493
B	-1.789279	0.784419	0.376624
O	-0.923886	0.954377	-0.795183
O	-1.937641	2.063228	0.968008
C	-0.504757	2.319339	-0.863615
C	-1.611860	3.041633	-0.016617
C	-2.864697	3.340055	-0.839492
C	-1.140432	4.305152	0.681915
C	-0.442725	2.739103	-2.322516
C	0.881935	2.438719	-0.237685
H	0.364620	2.189084	-2.829166
H	-1.383398	2.516677	-2.842536
H	1.557416	1.731947	-0.741774
H	-2.695960	4.148070	-1.565987
H	-3.205595	2.443222	-1.375613
H	0.861260	2.183350	0.831083
H	1.296053	3.451404	-0.344589
H	-0.232415	3.815482	-2.414181
H	-3.667939	3.651911	-0.155570

H	-0.779127	5.045584	-0.047646
H	-0.333982	4.092348	1.396080
H	-1.976514	4.755008	1.238037
H	0.704738	-2.297364	0.464629

57

[1]A_PBE0/TZVP E_{tot}(RPBE0-D3(Acetonitrile)/def2TZVP) = -2552.55765603 $\langle S^2 \rangle$ = 0.0000

Fe	0.448866	-0.949184	0.156791
O	-3.016992	0.213948	-0.082691
C	2.849899	-0.383859	1.546325
H	3.378940	-0.132689	2.466498
O	1.796129	-0.959509	-1.111762
C	3.645454	-0.529020	0.362946
N	-1.032753	-0.231984	1.341272
C	5.035623	-0.355770	0.479284
H	5.446743	-0.135805	1.459672
N	1.572052	-0.519449	1.599598
C	5.868850	-0.458010	-0.609490
H	6.937814	-0.323776	-0.500660
C	5.310130	-0.735069	-1.862178
H	5.954177	-0.817104	-2.731302
C	3.952408	-0.906912	-2.011783
H	3.519351	-1.118303	-2.982719
C	3.077078	-0.814679	-0.911824
C	0.874452	-0.412009	2.877438
H	1.451864	0.159123	3.607675
H	0.741789	-1.423853	3.272206
C	-0.463995	0.251182	2.612114
H	-1.168884	0.062971	3.424901
H	-0.348611	1.327329	2.508526
C	-1.239517	-1.567086	1.193180
C	-2.269967	-2.028624	0.273142
C	-2.450228	-3.391016	0.022920
H	-1.774369	-4.099949	0.490218
C	-3.466299	-3.830787	-0.800807
H	-3.596887	-4.888846	-0.992033
C	-4.325669	-2.896878	-1.379924
H	-5.129388	-3.231512	-2.026371
C	-4.166493	-1.544944	-1.140867
H	-4.832571	-0.813580	-1.583732
C	-3.130894	-1.089614	-0.321495
H	-1.004687	-2.209979	2.034959
B	-1.748314	0.814639	0.339237
O	-0.839347	0.998433	-0.793338
O	-1.916900	2.089187	0.943383
C	-0.410871	2.363405	-0.825123
C	-1.537460	3.080331	-0.013417
C	-2.749858	3.410194	-0.876805
C	-1.080876	4.323252	0.722825
C	-0.295959	2.803957	-2.271273
C	0.952790	2.469181	-0.157049
H	0.520239	2.261417	-2.753709
H	-1.213791	2.601168	-2.822170
H	1.639694	1.776750	-0.648382
H	-2.533799	4.214890	-1.582287
H	-3.085093	2.533347	-1.432678
H	0.902667	2.202664	0.899198
H	1.362707	3.477431	-0.239321
H	-0.076749	3.872573	-2.334476
H	-3.565040	3.733677	-0.226234
H	-0.686297	5.063731	0.022958
H	-0.309311	4.090994	1.456861
H	-1.928048	4.771014	1.246956
H	0.638787	-2.367892	0.513007

57

[1]A-1_BP86/ZORA E_{tot} = -2568.83739595 $\langle S^2 \rangle$ = 0.0000

Fe	0.345266	-0.736715	0.342014
O	-3.041741	0.173798	0.008474
C	2.804230	-0.245616	1.564405
H	3.383928	0.013928	2.464122
O	1.544372	-0.762566	-1.052637
C	3.529328	-0.464641	0.335712
N	-1.102404	-0.167937	1.556461
C	4.952035	-0.389731	0.358657
H	5.446590	-0.189975	1.319048

N	1.500190	-0.325571	1.684310
C	5.715821	-0.564908	-0.796379
H	6.809991	-0.507847	-0.750819
C	5.061112	-0.813123	-2.026897
H	5.649804	-0.952184	-2.943120
C	3.666547	-0.884155	-2.088911
H	3.149972	-1.071466	-3.038596
C	2.868075	-0.722663	-0.922682
C	0.866553	-0.213557	3.019426
H	1.467767	0.425001	3.690668
H	0.829429	-1.229532	3.455439
C	-0.534753	0.361431	2.818820
H	-1.199853	0.120067	3.667119
H	-0.497863	1.455762	2.700578
C	-1.180046	-1.541588	1.348040
C	-2.133784	-2.047111	0.354609
C	-2.189588	-3.427630	0.038317
H	-1.486137	-4.108138	0.536312
C	-3.123190	-3.917412	-0.882429
H	-3.154185	-4.987708	-1.119603
C	-4.029816	-3.024607	-1.496406
H	-4.768002	-3.401167	-2.216025
C	-3.995634	-1.654900	-1.196548
H	-4.695091	-0.950607	-1.663496
C	-3.045704	-1.151371	-0.281841
H	-0.926328	-2.197479	2.192775
B	-1.780626	0.789607	0.476894
O	-0.757669	0.799924	-0.623972
O	-1.952402	2.139374	0.906145
C	-0.331088	2.199209	-0.835510
C	-1.540517	2.995895	-0.203023
C	-2.721348	3.145948	-1.178943
C	-1.156554	4.363106	0.366426
C	-0.132347	2.410179	-2.336162
C	0.985479	2.438722	-0.088791
H	0.741565	1.828359	-2.678105
H	-1.013650	2.081829	-2.910246
H	1.741539	1.719029	-0.441391
H	-2.488583	3.866062	-1.982828
H	-2.992842	2.178885	-1.634179
H	0.861828	2.304472	0.998098
H	1.364080	3.457461	-0.279892
H	0.054836	3.477573	-2.552111
H	-3.596542	3.521646	-0.620514
H	-0.712309	4.998947	-0.420223
H	-0.435161	4.266074	1.193967
H	-2.057945	4.871919	0.751376
H	0.805737	-2.101657	0.647868

57

^{1}**A-1_PBE/ZORA** E_{tot} = -2566.76266185 $\langle S^2 \rangle$ = 0.0000

Fe	0.363231	-0.729550	0.328972
O	-3.022347	0.167087	-0.012541
C	2.816924	-0.275305	1.572605
H	3.392647	-0.027951	2.478228
O	1.574789	-0.761178	-1.056362
C	3.548968	-0.498882	0.350760
N	-1.087801	-0.165505	1.539519
C	4.971196	-0.445727	0.388376
H	5.457304	-0.258237	1.355411
N	1.510823	-0.337614	1.682203
C	5.743385	-0.626424	-0.758689
H	6.837685	-0.586359	-0.701838
C	5.097849	-0.857881	-1.995851
H	5.693398	-1.001160	-2.906898
C	3.704248	-0.907195	-2.072330
H	3.195662	-1.081781	-3.028729
C	2.896462	-0.739840	-0.914218
C	0.871282	-0.221493	3.011805
H	1.473267	0.415352	3.683974
H	0.828526	-1.235231	3.453474
C	-0.525076	0.358036	2.805062
H	-1.194901	0.116069	3.649828
H	-0.485204	1.453212	2.693451
C	-1.158760	-1.539425	1.328085
C	-2.118010	-2.050883	0.344475
C	-2.182722	-3.433303	0.042261

H	-1.480961	-4.113274	0.543461
C	-3.123525	-3.927450	-0.866920
H	-3.161143	-4.999878	-1.092870
C	-4.029302	-3.037294	-1.483005
H	-4.773914	-3.417354	-2.194043
C	-3.987808	-1.666516	-1.195934
H	-4.688763	-0.964714	-1.664485
C	-3.030213	-1.157862	-0.292548
H	-0.897347	-2.193672	2.171575
B	-1.767696	0.792407	0.462485
O	-0.744440	0.826114	-0.634606
O	-1.957892	2.136679	0.901750
C	-0.351581	2.231866	-0.854083
C	-1.563302	3.009266	-0.197534
C	-2.753140	3.170676	-1.159081
C	-1.186105	4.371202	0.387350
C	-0.188152	2.447409	-2.358106
C	0.977065	2.499701	-0.140054
H	0.683864	1.876142	-2.721965
H	-1.078272	2.113416	-2.914894
H	1.738564	1.791864	-0.505969
H	-2.528539	3.900440	-1.956219
H	-3.032983	2.211874	-1.626174
H	0.885351	2.377800	0.951786
H	1.333120	3.522966	-0.348773
H	-0.014672	3.516191	-2.577567
H	-3.622095	3.542276	-0.588508
H	-0.770152	5.029754	-0.395734
H	-0.444767	4.272685	1.196642
H	-2.086543	4.858659	0.800601
H	0.814888	-2.099561	0.626186

57

[1]**A-1_BP86/SVP** E_{tot}(RBP86-D3(Acetonitrile)/def2SVP/W06) = -2552.98943232 $\langle S^2 \rangle$ = 0.0000

Fe	0.342836	-0.734645	0.325973
O	-3.012679	0.224166	-0.057178
C	2.802775	-0.282326	1.577030
H	3.377750	-0.027705	2.487518
O	1.577959	-0.795510	-1.043624
C	3.545268	-0.525641	0.364343
N	-1.112693	-0.144829	1.528897
C	4.969149	-0.482078	0.407292
H	5.457874	-0.282007	1.375332
N	1.490617	-0.333682	1.680890
C	5.744602	-0.688609	-0.734468
H	6.843081	-0.655742	-0.674501
C	5.100508	-0.938325	-1.970986
H	5.701386	-1.103188	-2.880073
C	3.706423	-0.978585	-2.053217
H	3.195687	-1.166013	-3.010468
C	2.889890	-0.784952	-0.900967
C	0.845873	-0.205395	3.004489
H	1.448717	0.427168	3.688693
H	0.788834	-1.220675	3.453700
C	-0.546844	0.387235	2.787260
H	-1.222534	0.166999	3.639699
H	-0.499216	1.484835	2.657530
C	-1.202504	-1.519070	1.320013
C	-2.175894	-2.013866	0.339994
C	-2.279060	-3.397672	0.053238
H	-1.590565	-4.093220	0.560347
C	-3.235388	-3.877549	-0.849193
H	-3.302540	-4.954960	-1.064285
C	-4.118588	-2.968719	-1.473941
H	-4.878080	-3.337723	-2.181583
C	-4.037168	-1.595370	-1.204588
H	-4.718760	-0.874499	-1.681546
C	-3.063305	-1.097864	-0.308903
H	-0.964358	-2.180417	2.171875
B	-1.758125	0.825450	0.435216
O	-0.718069	0.824261	-0.646275
O	-1.902475	2.173038	0.866648
C	-0.276194	2.202610	-0.867875
C	-1.468855	3.026147	-0.220815
C	-2.643627	3.221518	-1.198252
C	-1.048793	4.376249	0.364758
C	-0.085112	2.405240	-2.371003

C	1.053010	2.418763	-0.133425
H	0.766144	1.786188	-2.719029
H	-0.984803	2.102001	-2.938523
H	1.792219	1.676953	-0.490377
H	-2.393926	3.945147	-1.999994
H	-2.944971	2.261260	-1.660035
H	0.933935	2.284936	0.958787
H	1.457729	3.432081	-0.325236
H	0.139631	3.467650	-2.597882
H	-3.512461	3.614126	-0.632348
H	-0.593739	5.020091	-0.415369
H	-0.323883	4.251054	1.191351
H	-1.938763	4.901350	0.767331
H	0.782161	-2.100670	0.657913

57

^{1}**A-1_BP86/TZVP** E_{tot}(RBP86-D3(Acetonitrile)/def2TZVP/W06) = -2554.55704044 $\langle S^2 \rangle$ = 0.0000

Fe	0.314338	-0.803599	0.315360
O	-2.989280	0.299127	-0.093419
C	2.775709	-0.360876	1.567873
H	3.346401	-0.116667	2.471864
O	1.556077	-0.889601	-1.047704
C	3.514222	-0.594274	0.358230
N	-1.120303	-0.143464	1.510361
C	4.930060	-0.532018	0.401783
H	5.410551	-0.330507	1.362730
N	1.471339	-0.404123	1.669209
C	5.704261	-0.720378	-0.733685
H	6.792182	-0.672106	-0.674025
C	5.067144	-0.971010	-1.965351
H	5.665235	-1.120334	-2.866955
C	3.681789	-1.031916	-2.047945
H	3.181358	-1.221115	-2.999406
C	2.868410	-0.856184	-0.900764
C	0.830096	-0.269020	2.993959
H	1.443312	0.342849	3.671652
H	0.746963	-1.277902	3.428099
C	-0.542996	0.359758	2.777988
H	-1.222793	0.145435	3.615358
H	-0.462600	1.446938	2.662004
C	-1.281735	-1.506545	1.316128
C	-2.254780	-1.964108	0.328294
C	-2.409736	-3.338248	0.052411
H	-1.765285	-4.051229	0.572290
C	-3.363982	-3.782012	-0.857559
H	-3.472972	-4.847709	-1.063003
C	-4.190861	-2.846564	-1.503590
H	-4.945325	-3.186766	-2.215581
C	-4.057100	-1.482923	-1.245908
H	-4.694403	-0.747127	-1.739354
C	-3.085278	-1.024158	-0.340350
H	-1.070977	-2.164474	2.163589
B	-1.720475	0.859061	0.420254
O	-0.665323	0.854114	-0.642673
O	-1.849278	2.208122	0.869959
C	-0.186538	2.229075	-0.827372
C	-1.368999	3.066814	-0.200776
C	-2.515230	3.298447	-1.197177
C	-0.934645	4.395910	0.409879
C	0.044510	2.451616	-2.318220
C	1.127359	2.400955	-0.063589
H	0.885941	1.828234	-2.652786
H	-0.841297	2.180734	-2.905627
H	1.848886	1.652555	-0.415960
H	-2.226173	4.012015	-1.982047
H	-2.828744	2.357360	-1.667891
H	0.983426	2.256334	1.014663
H	1.553401	3.399519	-0.233295
H	0.293437	3.504714	-2.517023
H	-3.375692	3.712481	-0.652670
H	-0.455604	5.032872	-0.348247
H	-0.232647	4.244587	1.239429
H	-1.815495	4.928596	0.796255
H	0.703579	-2.186056	0.663462

57
[1]A-1_PBE/SVP E_{tot}(RPBE-D3(Acetonitrile)/def2SVP/W06) = -2550.95108367 $\langle S^2 \rangle$ = 0.0000

Fe	0.375851	-0.715959	0.306613
O	-3.006028	0.157554	-0.051906
C	2.823973	-0.263879	1.573621
H	3.393242	-0.018131	2.489324
O	1.617999	-0.741940	-1.057927
C	3.573041	-0.487298	0.363849
N	-1.090153	-0.175766	1.516634
C	4.994619	-0.440943	0.417141
H	5.474300	-0.255231	1.391697
N	1.512427	-0.323823	1.671674
C	5.777421	-0.627111	-0.720766
H	6.874648	-0.592888	-0.652702
C	5.143212	-0.857549	-1.963813
H	5.750184	-1.006104	-2.870787
C	3.751628	-0.899613	-2.056150
H	3.248760	-1.072541	-3.019569
C	2.927340	-0.727260	-0.908026
C	0.864751	-0.215121	2.992204
H	1.456516	0.424764	3.678220
H	0.827319	-1.231450	3.440605
C	-0.536621	0.351378	2.779495
H	-1.207605	0.106941	3.628696
H	-0.512954	1.451376	2.666760
C	-1.145342	-1.548582	1.292079
C	-2.121882	-2.061070	0.326661
C	-2.206393	-3.444713	0.041099
H	-1.499843	-4.129038	0.537248
C	-3.167142	-3.940267	-0.845208
H	-3.219015	-5.017956	-1.059460
C	-4.074577	-3.047999	-1.454317
H	-4.838203	-3.429489	-2.149730
C	-4.013448	-1.675821	-1.184596
H	-4.715538	-0.967503	-1.649163
C	-3.034997	-1.161690	-0.305173
H	-0.880088	-2.210656	2.134424
B	-1.765715	0.788522	0.438753
O	-0.737961	0.837735	-0.650355
O	-1.948198	2.124092	0.890701
C	-0.360268	2.231126	-0.875873
C	-1.562795	3.007278	-0.186028
C	-2.762211	3.194440	-1.131651
C	-1.170615	4.356067	0.417964
C	-0.227283	2.449460	-2.382127
C	0.983571	2.499162	-0.189720
H	0.630250	1.861228	-2.764703
H	-1.135106	2.123323	-2.921931
H	1.739021	1.788880	-0.574811
H	-2.546970	3.936367	-1.925358
H	-3.058772	2.239272	-1.605230
H	0.914563	2.368465	0.906918
H	1.339950	3.526692	-0.397025
H	-0.042135	3.518228	-2.610926
H	-3.624075	3.561246	-0.539783
H	-0.767578	5.036882	-0.358146
H	-0.413908	4.240082	1.215850
H	-2.064520	4.836926	0.862682
H	0.835684	-2.079718	0.614967

57
[1]A-1_PBE/TZVP E_{tot}(RPBE-D3(Acetonitrile)/def2TZVP/W06) = -2552.50402324 $\langle S^2 \rangle$ = 0.0000

Fe	0.353331	-0.782780	0.299293
O	-2.982221	0.207805	-0.098983
C	2.799772	-0.311708	1.562324
H	3.363863	-0.063666	2.469174
O	1.601132	-0.842283	-1.058841
C	3.545824	-0.534369	0.357639
N	-1.097199	-0.170117	1.496816
C	4.959304	-0.461150	0.411241
H	5.429710	-0.258876	1.376733
N	1.495972	-0.370545	1.658742
C	5.742252	-0.640027	-0.717965
H	6.829077	-0.583868	-0.650503
C	5.116093	-0.891518	-1.953305
H	5.721198	-1.033905	-2.851049
C	3.733151	-0.962614	-2.045659

H	3.241845	-1.153395	-3.001395
C	2.910774	-0.796809	-0.904797
C	0.852331	-0.247380	2.980380
H	1.450663	0.384112	3.653050
H	0.797632	-1.254186	3.424252
C	-0.535503	0.342359	2.765133
H	-1.208783	0.104110	3.601520
H	-0.487651	1.432467	2.658944
C	-1.211543	-1.537159	1.299092
C	-2.180619	-2.028244	0.325732
C	-2.304166	-3.406813	0.062375
H	-1.637436	-4.099843	0.580930
C	-3.256273	-3.881809	-0.831792
H	-3.340443	-4.951375	-1.027245
C	-4.113495	-2.973859	-1.474159
H	-4.867263	-3.338527	-2.174368
C	-4.012570	-1.607061	-1.227459
H	-4.675968	-0.892529	-1.717586
C	-3.042972	-1.116011	-0.337348
H	-0.970436	-2.187596	2.143911
B	-1.736065	0.815644	0.415974
O	-0.688629	0.872116	-0.649004
O	-1.924210	2.152080	0.880953
C	-0.288932	2.268094	-0.839165
C	-1.495481	3.045583	-0.177895
C	-2.663476	3.256260	-1.151162
C	-1.107744	4.379411	0.451331
C	-0.111402	2.509014	-2.334083
C	1.036518	2.510212	-0.117293
H	0.745782	1.925976	-2.699062
H	-1.000911	2.205368	-2.898691
H	1.786886	1.801874	-0.491789
H	-2.410466	3.991358	-1.927870
H	-2.958443	2.316426	-1.635231
H	0.939855	2.369376	0.966787
H	1.403027	3.528785	-0.304449
H	0.087944	3.571750	-2.534888
H	-3.526582	3.635982	-0.586821
H	-0.684762	5.058945	-0.302454
H	-0.374851	4.246921	1.256523
H	-2.002221	4.857650	0.874794
H	0.771408	-2.158793	0.634393

57

^{1}**A-1_PBE0/SVP** E_{tot}(RPBE0-D3(Acetonitrile)/def2SVP) = -2551.02131915 $\langle S^2 \rangle$ = 0.0000

Fe	0.309696	-0.779556	0.294087
O	-2.965261	0.254444	-0.069524
C	2.777467	-0.313053	1.561405
H	3.363223	-0.078890	2.462200
O	1.588607	-0.869233	-1.049117
C	3.518840	-0.539558	0.349682
N	-1.098891	-0.156219	1.504034
C	4.928991	-0.466649	0.409636
H	5.396540	-0.248294	1.374472
N	1.493705	-0.368850	1.672865
C	5.715474	-0.665010	-0.709475
H	6.803559	-0.607966	-0.642581
C	5.086667	-0.940299	-1.937505
H	5.693777	-1.099290	-2.833257
C	3.709067	-1.013279	-2.032868
H	3.218584	-1.222208	-2.986384
C	2.874549	-0.822153	-0.900747
C	0.847616	-0.250969	2.971786
H	1.442061	0.353185	3.674698
H	0.763155	-1.263728	3.401082
C	-0.525274	0.359581	2.749692
H	-1.199292	0.153874	3.595378
H	-0.458447	1.447897	2.628171
C	-1.217071	-1.509915	1.306731
C	-2.200437	-1.981605	0.328494
C	-2.338751	-3.351132	0.051413
H	-1.675526	-4.057973	0.557876
C	-3.298925	-3.805584	-0.841713
H	-3.395691	-4.873031	-1.050239
C	-4.146750	-2.881036	-1.466037
H	-4.909828	-3.228094	-2.167458
C	-4.027588	-1.521784	-1.204905

H	-4.682875	-0.790446	-1.682850
C	-3.049687	-1.051211	-0.315224
H	-1.008842	-2.165824	2.157708
B	-1.718911	0.833119	0.424355
O	-0.682026	0.852919	-0.635954
O	-1.849459	2.165763	0.873308
C	-0.254606	2.208675	-0.851195
C	-1.424064	3.023518	-0.185266
C	-2.595259	3.245536	-1.142263
C	-0.992954	4.352615	0.410363
C	-0.086197	2.431282	-2.343622
C	1.077838	2.417852	-0.143774
H	0.749482	1.817551	-2.710911
H	-0.989303	2.142621	-2.896256
H	1.798019	1.673027	-0.508799
H	-2.344486	3.969866	-1.930483
H	-2.912166	2.303579	-1.610361
H	0.978192	2.292318	0.942985
H	1.484658	3.419056	-0.343599
H	0.139735	3.486384	-2.559684
H	-3.446121	3.640519	-0.567755
H	-0.554974	5.004854	-0.359983
H	-0.258521	4.213396	1.214270
H	-1.868219	4.866640	0.834547
H	0.643494	-2.156769	0.663525

57

[1]A-1_PBE0/TZVP E_{tot}(RPBE0-D3(Acetonitrile)/def2TZVP) = -2552.56224342 $\langle S^2 \rangle$ = 0.0000

Fe	0.291192	-0.831867	0.285435
O	-2.944050	0.307047	-0.103414
C	2.754469	-0.374626	1.553514
H	3.334836	-0.147703	2.448929
O	1.570837	-0.948235	-1.053857
C	3.491900	-0.592373	0.343989
N	-1.105029	-0.155422	1.485696
C	4.893729	-0.502166	0.404870
H	5.352934	-0.283245	1.364087
N	1.477139	-0.428573	1.659996
C	5.679858	-0.683577	-0.708318
H	6.758269	-0.612650	-0.641008
C	5.058346	-0.959237	-1.931966
H	5.663022	-1.103592	-2.821249
C	3.688750	-1.051182	-2.027897
H	3.208504	-1.261216	-2.976882
C	2.856983	-0.877795	-0.900723
C	0.833110	-0.311818	2.959535
H	1.435489	0.269579	3.661352
H	0.723089	-1.320477	3.369926
C	-0.520876	0.333741	2.738795
H	-1.198487	0.134381	3.571745
H	-0.422081	1.412147	2.630297
C	-1.277222	-1.497325	1.300504
C	-2.257884	-1.943696	0.317274
C	-2.434017	-3.304400	0.052126
H	-1.804497	-4.021189	0.569426
C	-3.390825	-3.733493	-0.845104
H	-3.518560	-4.790452	-1.044345
C	-4.195174	-2.792013	-1.487070
H	-4.952773	-3.118844	-2.190583
C	-4.037715	-1.441658	-1.238124
H	-4.659200	-0.702448	-1.730057
C	-3.062580	-0.998868	-0.342636
H	-1.085171	-2.149631	2.145305
B	-1.689009	0.855636	0.407256
O	-0.640753	0.876658	-0.637532
O	-1.815133	2.186410	0.874330
C	-0.188746	2.232704	-0.816603
C	-1.351176	3.053190	-0.163959
C	-2.495685	3.312873	-1.137464
C	-0.910028	4.361970	0.458991
C	0.010505	2.483988	-2.297340
C	1.131653	2.411962	-0.087316
H	0.846052	1.881304	-2.659167
H	-0.877716	2.217661	-2.869069
H	1.844736	1.673097	-0.455130
H	-2.209242	4.033975	-1.905347
H	-2.821740	2.391917	-1.621734

```
H        1.016513     2.270623     0.987578
H        1.545046     3.406172    -0.264594
H        0.244296     3.535423    -2.480815
H       -3.340933     3.722104    -0.580423
H       -0.451648     5.011877    -0.289987
H       -0.195622     4.199124     1.265528
H       -1.778851     4.880139     0.870285
H        0.581548    -2.222022     0.661820
```

57

[1]TS(A-1,B) E_{tot}(UPBE-D3(Acetonitrile)/def2SVP/W06) = -2550.93565760 $\langle S^2 \rangle$ = 0.4509 ν_{imag} = 604.55 i cm^{-1}

```
Fe       0.551007    -0.399273     0.150429
O       -3.084812     0.012685     0.167036
C        2.988068    -0.629902     1.564846
H        3.531267    -0.684042     2.528246
O        1.882257    -0.197266    -1.125070
C        3.775863    -0.555228     0.364601
N       -0.942606    -0.392358     1.395493
C        5.193611    -0.655386     0.463815
H        5.636359    -0.814165     1.460513
N        1.669076    -0.622232     1.595730
C        6.014863    -0.563563    -0.656839
H        7.106619    -0.650811    -0.554929
C        5.428775    -0.351920    -1.928457
H        6.068604    -0.275198    -2.821665
C        4.046212    -0.237309    -2.062639
H        3.579636    -0.067575    -3.044827
C        3.178124    -0.339056    -0.936275
C        0.963369    -0.778323     2.885312
H        1.532734    -0.312564     3.715973
H        0.889596    -1.863499     3.116924
C       -0.425941    -0.144904     2.753052
H       -1.119890    -0.536202     3.527986
H       -0.374581     0.955827     2.861282
C       -0.887786    -1.707145     0.852298
C       -2.046754    -2.162899     0.033498
C       -2.121884    -3.495084    -0.422393
H       -1.288423    -4.181570    -0.200334
C       -3.236305    -3.951957    -1.136988
H       -3.280772    -4.993891    -1.486759
C       -4.301545    -3.065730    -1.393003
H       -5.186550    -3.413791    -1.948019
C       -4.244365    -1.738931    -0.949990
H       -5.066493    -1.033528    -1.142748
C       -3.113598    -1.265619    -0.246234
H       -0.570094    -2.479372     1.581799
B       -1.799722     0.631715     0.541390
O       -0.938571     0.846259    -0.694246
O       -1.955450     1.903285     1.155663
C       -0.682874     2.288735    -0.806864
C       -1.815078     2.900839     0.122499
C       -3.155449     3.068024    -0.616213
C       -1.435781     4.223646     0.789928
C       -0.782149     2.669214    -2.282215
C        0.721374     2.604884    -0.278000
H        0.046577     2.195299    -2.844947
H       -1.736398     2.330058    -2.725170
H        1.475464     2.061386    -0.875498
H       -3.116446     3.900995    -1.345253
H       -3.448547     2.141894    -1.145099
H        0.829952     2.303132     0.784377
H        0.940927     3.687417    -0.351261
H       -0.700570     3.767238    -2.411734
H       -3.943105     3.296840     0.128679
H       -1.196837     4.998757     0.034620
H       -0.568628     4.103866     1.465330
H       -2.289967     4.588967     1.394085
H        0.244804    -1.894843     0.032284
```

57

[1]B E_{tot}(RPBE-D3(Acetonitrile)/def2SVP/W06) = -2550.93964302 $\langle S^2 \rangle$ = 0.0000

```
Fe       0.758324    -0.679160    -0.408429
O       -2.448138     0.351711    -1.074779
C        2.833618    -1.289195     1.343139
H        3.164076    -1.672737     2.326834
O        2.278988    -0.567554    -1.408828
```

C	3.846591	-0.929077	0.399797
N	-1.037075	-0.951852	0.516527
C	5.220003	-0.959106	0.788250
H	5.462861	-1.223746	1.830085
N	1.520868	-1.222002	1.123524
C	6.238179	-0.665439	-0.112358
H	7.288831	-0.689025	0.213431
C	5.915491	-0.341558	-1.455604
H	6.718847	-0.109217	-2.172046
C	4.587724	-0.315816	-1.876111
H	4.320022	-0.069480	-2.914885
C	3.522297	-0.593091	-0.972039
C	0.597913	-1.759666	2.145417
H	1.002623	-1.613348	3.169547
H	0.497320	-2.854895	1.981444
C	-0.738178	-1.054171	1.962895
H	-1.551250	-1.580837	2.506044
H	-0.689938	-0.025180	2.366618
C	-1.811539	-2.093327	-0.037367
C	-3.261920	-1.737778	-0.240960
C	-4.343536	-2.590341	0.028612
H	-4.151261	-3.576771	0.481529
C	-5.657869	-2.200863	-0.288311
H	-6.500497	-2.875669	-0.074641
C	-5.886388	-0.946501	-0.881599
H	-6.912317	-0.636784	-1.135542
C	-4.816584	-0.074555	-1.143621
H	-4.980043	0.915337	-1.595937
C	-3.502536	-0.464979	-0.820429
H	-1.693659	-2.984724	0.610502
B	-1.452637	0.467604	0.007044
O	-0.142285	0.981435	-0.588188
O	-1.765322	1.439925	1.021734
C	0.189785	2.300048	-0.032180
C	-1.195706	2.703544	0.641771
C	-2.139761	3.420095	-0.342137
C	-1.034072	3.556259	1.901753
C	0.642785	3.184862	-1.190788
C	1.325955	2.127488	0.977031
H	1.597285	2.798815	-1.600767
H	-0.101777	3.201063	-2.007185
H	2.187664	1.641106	0.480730
H	-1.782918	4.440115	-0.585287
H	-2.263565	2.847399	-1.279974
H	1.020993	1.494192	1.830568
H	1.657370	3.110166	1.363883
H	0.811431	4.223853	-0.844382
H	-3.137265	3.506059	0.132620
H	-0.503977	4.503550	1.678074
H	-0.479114	3.015731	2.690707
H	-2.034958	3.811652	2.302754
H	-1.366021	-2.364647	-1.021621

57

^{3}A-1 E_{tot}(UPBE-D3(Acetonitrile)/def2SVP/W06) = -2550.93518698 $\langle S^2 \rangle$ = 2.0592

Fe	0.453948	-0.862738	-0.020943
O	-3.045389	0.440086	0.035532
C	2.893281	-0.744575	1.574836
H	3.414897	-0.736354	2.553986
O	1.900401	-0.859380	-1.222481
C	3.724715	-0.738091	0.403074
N	-1.022533	-0.376392	1.277219
C	5.138181	-0.669811	0.579512
H	5.531759	-0.621890	1.607630
N	1.581721	-0.760814	1.572360
C	6.010366	-0.659753	-0.502488
H	7.097116	-0.605830	-0.343557
C	5.478805	-0.722821	-1.815211
H	6.159060	-0.716064	-2.681456
C	4.105741	-0.797590	-2.027551
H	3.685290	-0.846656	-3.043039
C	3.181390	-0.806092	-0.937989
C	0.836311	-0.843304	2.837177
H	1.403369	-0.411649	3.688876
H	0.667505	-1.917841	3.070471
C	-0.484258	-0.109498	2.635501
H	-1.236476	-0.398783	3.398008

H	-0.342110	0.985646	2.689384
C	-1.319709	-1.671867	0.869608
C	-2.536976	-1.920463	0.089824
C	-2.935209	-3.233858	-0.244437
H	-2.280914	-4.073018	0.041813
C	-4.133255	-3.473431	-0.924760
H	-4.429231	-4.501985	-1.178939
C	-4.960118	-2.385494	-1.275506
H	-5.906632	-2.562588	-1.809381
C	-4.586262	-1.076465	-0.951476
H	-5.219583	-0.216277	-1.215238
C	-3.370111	-0.824794	-0.276562
H	-1.001558	-2.488664	1.542599
B	-1.680477	0.827821	0.429714
O	-0.784524	0.967938	-0.759079
O	-1.644131	2.072444	1.123255
C	-0.250540	2.325831	-0.774498
C	-1.265035	3.091270	0.175057
C	-2.521796	3.571555	-0.572457
C	-0.647162	4.259908	0.943426
C	-0.231315	2.811489	-2.223374
C	1.176237	2.308732	-0.212875
H	0.505938	2.222113	-2.804473
H	-1.219773	2.693454	-2.703965
H	1.791252	1.609314	-0.810369
H	-2.298543	4.419540	-1.249159
H	-2.979122	2.754315	-1.162004
H	1.191968	1.970159	0.841547
H	1.646056	3.309839	-0.265632
H	0.065040	3.878349	-2.277343
H	-3.268277	3.910706	0.172816
H	-0.242096	5.022619	0.248688
H	0.165722	3.922322	1.612791
H	-1.423550	4.745511	1.567622
H	-0.022217	-2.207714	-0.673594

57

³TS(A-1,B) E_{tot}(UPBE-D3(Acetonitrile)/def2SVP/W06) = -2550.93498155 $\langle S^2 \rangle$ = 2.0510 ν_{imag} = 262.00 i cm^{-1}

Fe	0.479971	-0.837653	-0.002266
O	-3.061241	0.438802	0.104790
C	2.912160	-0.712653	1.578264
H	3.435437	-0.704650	2.556310
O	1.912377	-0.918197	-1.211251
C	3.738808	-0.706552	0.402728
N	-1.009060	-0.394975	1.283361
C	5.151661	-0.607606	0.570962
H	5.548153	-0.528951	1.596070
N	1.599025	-0.732876	1.577914
C	6.019827	-0.604925	-0.514364
H	7.105746	-0.524731	-0.360616
C	5.485870	-0.710788	-1.823364
H	6.162943	-0.710794	-2.692116
C	4.113873	-0.819743	-2.028161
H	3.691428	-0.904167	-3.040538
C	3.193718	-0.819276	-0.935466
C	0.854820	-0.847019	2.842744
H	1.412566	-0.410484	3.698446
H	0.715903	-1.928478	3.063550
C	-0.487192	-0.147421	2.651832
H	-1.232119	-0.475656	3.405823
H	-0.374749	0.949872	2.729278
C	-1.304932	-1.690973	0.849838
C	-2.535364	-1.919266	0.074486
C	-2.928137	-3.222921	-0.299847
H	-2.263102	-4.066395	-0.053217
C	-4.134567	-3.449598	-0.970371
H	-4.425429	-4.471267	-1.256231
C	-4.975053	-2.357445	-1.270772
H	-5.928110	-2.523565	-1.796501
C	-4.605566	-1.057494	-0.907898
H	-5.248693	-0.193603	-1.133150
C	-3.380919	-0.818437	-0.243285
H	-1.022025	-2.507668	1.538530
B	-1.679938	0.812768	0.453750
O	-0.825146	0.941924	-0.768648
O	-1.606963	2.057624	1.143458
C	-0.294860	2.301286	-0.814510

C	-1.262282	3.073130	0.179293
C	-2.547317	3.564006	-0.511202
C	-0.602835	4.236954	0.920387
C	-0.346502	2.781489	-2.264593
C	1.157633	2.286050	-0.323581
H	0.362001	2.190970	-2.879308
H	-1.357120	2.661166	-2.696038
H	1.743163	1.586590	-0.950528
H	-2.347540	4.409476	-1.198265
H	-3.038477	2.750361	-1.077921
H	1.224543	1.948002	0.729281
H	1.624630	3.286930	-0.400409
H	-0.054055	3.848366	-2.336919
H	-3.255909	3.910380	0.267046
H	-0.225020	4.998662	0.209402
H	0.237417	3.893431	1.551857
H	-1.347308	4.725940	1.579829
H	-0.042866	-2.265650	-0.417918

57

^{3}B E_{tot}(UPBE-D3(Acetonitrile)/def2SVP/W06) = -2550.97193449 $\langle S^2 \rangle$ = 2.0522

Fe	0.833274	-0.427166	-0.404682
O	-2.671782	0.480369	-0.887884
C	2.825462	-1.707779	1.134819
H	3.110131	-2.339833	1.998549
O	2.444658	0.020838	-1.216875
C	3.891296	-1.153087	0.352009
N	-0.953477	-0.916105	0.310458
C	5.239653	-1.442801	0.721130
H	5.411700	-2.089410	1.596925
N	1.534895	-1.519470	0.912159
C	6.319746	-0.935324	0.007987
H	7.349338	-1.173463	0.313474
C	6.080112	-0.106301	-1.117405
H	6.928783	0.301546	-1.688867
C	4.779191	0.199774	-1.508091
H	4.579823	0.842348	-2.379134
C	3.649293	-0.303936	-0.797267
C	0.531025	-2.189056	1.760642
H	0.899841	-2.319628	2.799877
H	0.341708	-3.206032	1.350782
C	-0.733520	-1.333799	1.713268
H	-1.613443	-1.881553	2.116089
H	-0.603844	-0.411746	2.311530
C	-1.599749	-1.967339	-0.525018
C	-3.097601	-1.830143	-0.476156
C	-4.008755	-2.878453	-0.282864
H	-3.629008	-3.898557	-0.109295
C	-5.393665	-2.632645	-0.326610
H	-6.106307	-3.457646	-0.176872
C	-5.859551	-1.327167	-0.565883
H	-6.942418	-1.129755	-0.604428
C	-4.958344	-0.264336	-0.745926
H	-5.311073	0.762780	-0.924170
C	-3.571912	-0.510891	-0.696124
H	-1.259169	-2.974849	-0.216695
B	-1.488370	0.536995	0.004118
O	-0.345556	1.190793	-0.752504
O	-1.660974	1.346871	1.177445
C	-0.065630	2.506935	-0.160362
C	-1.291623	2.694759	0.845190
C	-2.489630	3.413946	0.195196
C	-0.920959	3.417505	2.142978
C	-0.005038	3.524844	-1.298202
C	1.287653	2.447043	0.554887
H	0.869935	3.311020	-1.943930
H	-0.913517	3.487411	-1.926437
H	2.073156	2.153557	-0.166609
H	-2.274736	4.485258	0.013482
H	-2.786166	2.937948	-0.757227
H	1.281629	1.708573	1.382248
H	1.557631	3.433696	0.978106
H	0.106598	4.553064	-0.899691
H	-3.353124	3.347151	0.886643
H	-0.534810	4.436245	1.939897
H	-0.161405	2.857087	2.718755
H	-1.824397	3.515629	2.777205

H	-1.265549	-1.821936	-1.578050

57

^{5}A-1 E_{tot}(UPBE-D3(Acetonitrile)/def2SVP/W06) = -2550.92987286 $\langle S^2 \rangle$ = 6.0658

Fe	0.292733	-0.921672	-0.174002
O	-3.038400	0.561345	0.072870
C	2.819084	-0.901416	1.526927
H	3.325677	-0.889517	2.516117
O	1.914122	-0.770112	-1.248704
C	3.690624	-0.822802	0.385428
N	-1.176002	-0.342189	1.447020
C	5.096081	-0.784998	0.613493
H	5.458208	-0.837800	1.653138
N	1.512657	-0.972938	1.511305
C	6.001821	-0.685206	-0.437276
H	7.083692	-0.660733	-0.242161
C	5.510432	-0.612622	-1.765054
H	6.219530	-0.531081	-2.604140
C	4.143741	-0.641628	-2.027738
H	3.756540	-0.580974	-3.055818
C	3.187589	-0.753944	-0.970874
C	0.791578	-1.050022	2.779324
H	1.424876	-0.745094	3.639516
H	0.502976	-2.109583	2.955838
C	-0.455727	-0.161816	2.709148
H	-1.122793	-0.383323	3.570895
H	-0.179785	0.908451	2.764695
C	-1.413316	-1.607987	0.938913
C	-2.599611	-1.810640	0.097344
C	-3.010113	-3.104614	-0.295736
H	-2.383403	-3.965398	-0.012814
C	-4.184196	-3.295382	-1.028070
H	-4.493023	-4.308851	-1.324040
C	-4.973037	-2.177987	-1.380247
H	-5.899877	-2.318975	-1.957542
C	-4.583990	-0.888246	-1.005671
H	-5.183890	-0.005727	-1.273466
C	-3.390428	-0.685279	-0.274580
H	-1.107471	-2.467625	1.558659
B	-1.675575	0.859343	0.561714
O	-0.705666	0.895846	-0.614185
O	-1.582721	2.134528	1.182296
C	-0.110013	2.236246	-0.666863
C	-1.151448	3.079519	0.180028
C	-2.359153	3.534368	-0.658998
C	-0.545443	4.288252	0.894284
C	0.018385	2.632784	-2.135175
C	1.271402	2.211593	-0.004348
H	0.757157	1.972227	-2.631569
H	-0.944887	2.534521	-2.668315
H	1.915280	1.478834	-0.521330
H	-2.080839	4.342178	-1.363901
H	-2.799642	2.696297	-1.230631
H	1.204917	1.928752	1.063606
H	1.759430	3.202462	-0.073791
H	0.373722	3.678372	-2.230613
H	-3.138089	3.925015	0.025220
H	-0.083437	4.988438	0.170047
H	0.218684	3.983485	1.632776
H	-1.343525	4.834729	1.434981
H	-0.054055	-2.358305	-0.771923

57

^{5}TS(A-1,B) E_{tot}(UPBE-D3(Acetonitrile)/def2SVP/W06) = -2550.92702413 $\langle S^2 \rangle$ = 6.0553 ν_{imag} = 605.82 i cm^{-1}

Fe	0.370980	-0.840608	-0.191659
O	-3.041954	0.562168	0.045293
C	2.841596	-0.885986	1.533037
H	3.338842	-0.886586	2.527146
O	1.970553	-0.861192	-1.274197
C	3.723350	-0.816011	0.395839
N	-1.189644	-0.382641	1.402246
C	5.125159	-0.752178	0.640373
H	5.472804	-0.766067	1.686282
N	1.534739	-0.941623	1.499746
C	6.046138	-0.674862	-0.399529
H	7.124307	-0.627107	-0.188434

C	5.574904	-0.658176	-1.735554
H	6.294621	-0.595941	-2.567194
C	4.211929	-0.719726	-2.014983
H	3.839068	-0.705288	-3.050155
C	3.241576	-0.804828	-0.970301
C	0.788585	-1.073042	2.748061
H	1.394154	-0.773779	3.630840
H	0.535881	-2.147769	2.887476
C	-0.491444	-0.230869	2.680249
H	-1.157715	-0.507125	3.527815
H	-0.253974	0.845751	2.777766
C	-1.415488	-1.648138	0.856191
C	-2.658310	-1.823354	0.070017
C	-3.110474	-3.106130	-0.305435
H	-2.494891	-3.981908	-0.042684
C	-4.315838	-3.273933	-0.994970
H	-4.657295	-4.281091	-1.276319
C	-5.089973	-2.140084	-1.320425
H	-6.040868	-2.259451	-1.862590
C	-4.655980	-0.859195	-0.964210
H	-5.243909	0.036712	-1.214032
C	-3.432124	-0.680360	-0.276502
H	-1.160975	-2.501238	1.508836
B	-1.673994	0.831640	0.541604
O	-0.713407	0.901112	-0.651193
O	-1.565870	2.094295	1.190353
C	-0.128803	2.245780	-0.685411
C	-1.146038	3.066139	0.212436
C	-2.367195	3.560283	-0.584329
C	-0.513445	4.245318	0.953707
C	-0.046003	2.682470	-2.146082
C	1.273985	2.204813	-0.069025
H	0.681232	2.041247	-2.683503
H	-1.024185	2.592354	-2.652771
H	1.902902	1.488966	-0.630161
H	-2.097281	4.386222	-1.271329
H	-2.831038	2.744007	-1.168818
H	1.242872	1.888937	0.991356
H	1.761621	3.196885	-0.125492
H	0.300040	3.732644	-2.224181
H	-3.125000	3.938306	0.130175
H	-0.059847	4.965812	0.244171
H	0.263309	3.908586	1.664805
H	-1.294324	4.779983	1.530196
H	-0.332754	-2.264243	-0.409016

57

^{5}B E_{tot}(UPBE-D3(Acetonitrile)/def2SVP/W06) = -2550.96394117 $\langle S^2 \rangle$ = 6.0551

Fe	0.874605	-0.508657	-0.733364
O	-2.681352	0.422415	-1.005023
C	2.908341	-1.596993	1.184227
H	3.183524	-2.155500	2.105135
O	2.640694	-0.135981	-1.367014
C	4.006537	-1.049121	0.423278
N	-1.021103	-0.991407	0.227874
C	5.326740	-1.225644	0.926827
H	5.451559	-1.771639	1.876456
N	1.640240	-1.482589	0.875281
C	6.442996	-0.730568	0.257144
H	7.451947	-0.879621	0.669083
C	6.261162	-0.033233	-0.961102
H	7.135130	0.364072	-1.501277
C	4.984447	0.155810	-1.488415
H	4.832576	0.695695	-2.435451
C	3.821759	-0.339495	-0.826785
C	0.618469	-2.137903	1.687975
H	0.950315	-2.277457	2.740679
H	0.437397	-3.154249	1.268852
C	-0.668137	-1.308023	1.624801
H	-1.496880	-1.845464	2.138084
H	-0.525634	-0.349882	2.160686
C	-1.796427	-2.065129	-0.445464
C	-3.277858	-1.799618	-0.374104
C	-4.265865	-2.746246	-0.065926
H	-3.965150	-3.773275	0.199263
C	-5.628344	-2.395036	-0.110281
H	-6.399894	-3.141426	0.132311

C	-5.996103	-1.086094	-0.470105
H	-7.060718	-0.806361	-0.510276
C	-5.017380	-0.122605	-0.768013
H	-5.291561	0.908238	-1.039332
C	-3.653661	-0.473690	-0.714668
H	-1.533569	-3.055151	-0.022001
B	-1.525905	0.467363	-0.068377
O	-0.384112	1.178112	-0.772555
O	-1.761115	1.259232	1.116759
C	-0.076887	2.423487	-0.073584
C	-1.357484	2.608775	0.849557
C	-2.496569	3.359916	0.133142
C	-1.061653	3.298277	2.184058
C	0.134778	3.518693	-1.117421
C	1.211448	2.215836	0.733511
H	1.043352	3.297591	-1.712903
H	-0.722149	3.588267	-1.812100
H	2.021161	1.895303	0.047291
H	-2.261649	4.434448	0.000624
H	-2.720913	2.913433	-0.853149
H	1.082827	1.434312	1.507796
H	1.538270	3.150639	1.228756
H	0.277257	4.504314	-0.630417
H	-3.411354	3.279847	0.753755
H	-0.640748	4.311694	2.027113
H	-0.353457	2.710450	2.797115
H	-2.002344	3.405365	2.760254
H	-1.498248	-2.096020	-1.518324

35

^{5}**2a-mono** E_{tot}(UPBE-D3(Acetonitrile)/def2SVP/W06) = -2139.85867749 $\langle S^2 \rangle$ = 6.0832

Fe	-0.000000	0.000000	0.073146
O	0.064760	1.553902	1.228283
O	-0.064760	-1.553902	1.228283
N	0.283280	-1.307061	-1.527406
N	-0.283280	1.307061	-1.527406
C	-0.300888	2.615405	-1.490601
H	-0.443933	3.173713	-2.442712
C	-0.139897	3.421794	-0.306084
C	-0.158195	4.838799	-0.446732
H	-0.301376	5.258760	-1.456261
C	0.000000	5.688606	0.644676
H	-0.017279	6.780214	0.510273
C	0.184586	5.125993	1.931821
H	0.310635	5.786659	2.804528
C	0.207744	3.744396	2.109399
H	0.349184	3.299705	3.106457
C	0.046379	2.842099	1.011143
C	-0.046379	-2.842099	1.011143
C	-0.207744	-3.744396	2.109399
H	-0.349184	-3.299705	3.106457
C	-0.184586	-5.125993	1.931821
H	-0.310635	-5.786659	2.804528
C	-0.000000	-5.688606	0.644676
H	0.017279	-6.780214	0.510273
C	0.158195	-4.838799	-0.446732
H	0.301376	-5.258760	-1.456261
C	0.139897	-3.421794	-0.306084
C	0.300888	-2.615405	-1.490601
H	0.443933	-3.173713	-2.442712
C	0.458397	-0.614029	-2.796880
H	0.246274	-1.270488	-3.669811
H	1.515177	-0.273508	-2.878240
C	-0.458397	0.614029	-2.796880
H	-0.246274	1.270488	-3.669811
H	-1.515177	0.273508	-2.878240

38

^{5}**2b-mono** E_{tot}(UPBE-D3(Acetonitrile)/def2SVP/W06) = -2179.09540009 $\langle S^2 \rangle$ = 6.0777

Fe	0.000000	-0.000000	0.266925
O	0.472619	1.613738	1.207637
O	-0.472619	-1.613738	1.207637
N	0.977880	-1.063377	-1.159300
N	-0.977880	1.063377	-1.159300
C	-1.135750	2.363867	-1.166559
H	-1.609720	2.831853	-2.057672

```
C        -0.740978     3.264112    -0.109503
C        -1.116219     4.633323    -0.224710
H        -1.706357     4.936665    -1.105272
C        -0.763766     5.578651     0.734652
C         0.000000     5.166748     1.853715
H         0.288755     5.903195     2.620625
C         0.397707     3.838989     1.995772
C         0.050594     2.841092     1.032803
C        -0.050594    -2.841092     1.032803
C        -0.397707    -3.838989     1.995772
C        -0.000000    -5.166748     1.853715
H        -0.288755    -5.903195     2.620625
C         0.763766    -5.578651     0.734652
C         1.116219    -4.633323    -0.224710
H         1.706357    -4.936665    -1.105272
C         0.740978    -3.264112    -0.109503
C         1.135750    -2.363867    -1.166559
H         1.609720    -2.831853    -2.057672
C         1.272602    -0.288280    -2.362311
H         2.022682    -0.805347    -3.001188
H         1.722845     0.672808    -2.037211
C        -1.272602     0.288280    -2.362311
H        -2.022682     0.805347    -3.001188
H        -1.722845    -0.672808    -2.037211
H         0.998150     3.513337     2.858941
H        -1.070748     6.629006     0.623816
H        -0.998150    -3.513337     2.858941
H         1.070748    -6.629006     0.623816
C        -0.000000     0.000000    -3.195194
H        -0.219435    -0.864411    -3.855636
H         0.219435     0.864411    -3.855636
```

41

^{5}2c-mono E_{tot}(UPBE-D3(Acetonitrile)/def2SVP/W06) = -2218.32916912 $\langle S^2 \rangle$ = 6.0809

```
Fe        0.001919    -0.092836     0.467719
O         1.287264    -1.410634    -0.092256
O        -1.636100    -0.793170     1.197873
N        -1.018357     0.876408    -0.995710
N         1.424540     1.292469     0.862335
C         2.698497     1.095651     0.598850
H         3.386173     1.958708     0.732001
C         3.326215    -0.121507     0.149295
C         4.744748    -0.118591     0.005105
H         5.288688     0.810141     0.244380
C         5.445396    -1.244180    -0.417319
C         4.727019    -2.427854    -0.718673
H         5.268810    -3.326375    -1.054735
C         3.339998    -2.468819    -0.599293
C         2.589834    -1.330204    -0.169251
C        -2.801117    -0.927378     0.611973
C        -3.785394    -1.776142     1.205883
C        -5.047357    -1.944138     0.639126
H        -5.775043    -2.611933     1.127334
C        -5.404268    -1.264286    -0.550449
C        -4.471533    -0.422487    -1.150366
H        -4.733808     0.121415    -2.072878
C        -3.166895    -0.235730    -0.611161
C        -2.279758     0.681017    -1.287356
H        -2.727163     1.272908    -2.116450
C        -0.296719     1.976236    -1.631074
H        -0.691458     2.172369    -2.653415
H         0.763449     1.670540    -1.740188
C         1.027786     2.632102     1.314700
H         1.830764     3.364309     1.069464
H         0.940564     2.610110     2.423926
H         2.773263    -3.381936    -0.837290
H         6.540492    -1.215734    -0.516138
H        -3.504297    -2.296529     2.134171
H        -6.403460    -1.395888    -0.990927
C        -0.401843     3.271243    -0.787965
H        -1.381712     3.744355    -1.011827
H         0.376604     3.980697    -1.142566
C        -0.311180     3.104302     0.738098
H        -0.534545     4.085262     1.207209
H        -1.120387     2.424194     1.085199
```

39

^{5}2d-mono E_{tot}(UPBE-D3(Acetonitrile)/def2SVP/W06) = -2292.01259574 $\langle S^2 \rangle$ = 6.0815

Fe	0.566579	-0.405948	0.000000
O	1.744827	-0.585308	1.516957
O	1.744827	-0.585308	-1.516957
N	-0.998055	-0.038457	-1.323157
N	-0.998055	-0.038457	1.323157
C	-0.840399	0.325890	2.584104
H	-1.713281	0.735263	3.133103
C	0.381387	0.263484	3.331272
C	0.355314	0.681031	4.696296
H	-0.603024	1.031550	5.113539
C	1.492102	0.647335	5.495427
C	2.714122	0.191275	4.938213
H	3.621103	0.160135	5.562720
C	2.783622	-0.216585	3.608811
C	1.634096	-0.198575	2.758924
C	1.634096	-0.198575	-2.758924
C	2.783622	-0.216585	-3.608811
C	2.714122	0.191275	-4.938213
H	3.621103	0.160135	-5.562720
C	1.492102	0.647335	-5.495427
C	0.355314	0.681031	-4.696296
H	-0.603024	1.031550	-5.113539
C	0.381387	0.263484	-3.331272
C	-0.840399	0.325890	-2.584104
H	-1.713281	0.735263	-3.133103
C	-2.265267	-0.046602	-0.715115
C	-2.265267	-0.046602	0.715115
H	3.728642	-0.567531	3.167154
H	1.445690	0.969306	6.546102
H	3.728642	-0.567531	-3.167154
H	1.445690	0.969306	-6.546102
C	-3.493508	-0.113368	1.407540
C	-3.493508	-0.113368	-1.407540
C	-4.704909	-0.155430	0.703410
C	-4.704909	-0.155430	-0.703410
H	-3.502105	-0.158635	2.506121
H	-3.502105	-0.158635	-2.506121
H	-5.654146	-0.211832	1.257174
H	-5.654146	-0.211832	-1.257174

83

^{5}2a′-mono E_{tot}(UPBE-D3(Acetonitrile)/def2SVP/W06) = -2767.55653133 $\langle S^2 \rangle$ = 6.0836

Fe	0.000000	0.000000	0.946702
O	-0.029869	1.542393	-0.202791
O	0.029869	-1.542393	-0.202791
N	-0.265631	-1.312014	2.529282
N	0.265631	1.312014	2.529282
C	0.293929	2.621175	2.472099
H	0.431491	3.189602	3.419323
C	0.160883	3.422861	1.278930
C	0.196382	4.839029	1.427632
H	0.320852	5.234920	2.446727
C	0.079914	5.699249	0.339114
C	-0.075933	5.098886	-0.943880
H	-0.165935	5.769186	-1.809805
C	-0.119730	3.721014	-1.172632
C	0.000000	2.838765	-0.033753
C	-0.000000	-2.838765	-0.033753
C	0.119730	-3.721014	-1.172632
C	0.075933	-5.098886	-0.943880
H	0.165935	-5.769186	-1.809805
C	-0.079914	-5.699249	0.339114
C	-0.196382	-4.839029	1.427632
H	-0.320852	-5.234920	2.446727
C	-0.160883	-3.422861	1.278930
C	-0.293929	-2.621175	2.472099
H	-0.431491	-3.189602	3.419323
C	-0.441927	-0.627181	3.803185
H	-0.204136	-1.280663	4.672136
H	-1.506808	-0.315874	3.898657
C	0.441927	0.627181	3.803185
H	0.204136	1.280663	4.672136
H	1.506808	0.315874	3.898657
C	-0.287657	3.132362	-2.590020
C	-1.579650	2.279202	-2.653150

C	0.937628	2.250364	-2.940215
H	1.869996	2.852102	-2.929224
H	1.047264	1.418298	-2.220155
H	0.821422	1.821066	-3.957733
H	-1.545467	1.452595	-1.919417
H	-1.701620	1.844038	-3.667451
H	-2.472552	2.902971	-2.440638
C	-0.397268	4.232692	-3.663432
H	0.509808	4.870149	-3.702690
H	-0.516932	3.763730	-4.661188
H	-1.274431	4.891587	-3.498722
C	0.113204	7.234595	0.459594
C	1.295482	7.789528	-0.370759
C	-1.212868	7.821209	-0.081314
C	0.287443	7.695993	1.918171
H	2.261501	7.391537	0.002141
H	1.209629	7.520462	-1.442835
H	1.331370	8.897147	-0.305307
H	-1.380519	7.552360	-1.143736
H	-1.204943	8.929229	-0.013431
H	-2.078192	7.447100	0.503409
H	0.306077	8.803801	1.963476
H	-0.545909	7.349474	2.562792
H	1.237521	7.328107	2.356466
C	0.287657	-3.132362	-2.590020
C	0.397268	-4.232692	-3.663432
C	-0.937628	-2.250364	-2.940215
C	1.579650	-2.279202	-2.653150
H	1.274431	-4.891587	-3.498722
H	-0.509808	-4.870149	-3.702690
H	0.516932	-3.763730	-4.661188
H	-1.869996	-2.852102	-2.929224
H	-0.821422	-1.821066	-3.957733
H	-1.047264	-1.418298	-2.220155
H	1.701620	-1.844038	-3.667451
H	1.545467	-1.452595	-1.919417
H	2.472552	-2.902971	-2.440638
C	-0.113204	-7.234595	0.459594
C	-1.295482	-7.789528	-0.370759
C	1.212868	-7.821209	-0.081314
C	-0.287443	-7.695993	1.918171
H	-2.261501	-7.391537	0.002141
H	-1.209629	-7.520462	-1.442835
H	-1.331370	-8.897147	-0.305307
H	1.380519	-7.552360	-1.143736
H	1.204943	-8.929229	-0.013431
H	2.078192	-7.447100	0.503409
H	-0.306077	-8.803801	1.963476
H	0.545909	-7.349474	2.562792
H	-1.237521	-7.328107	2.356466

86

^{5}2b′-mono E_{tot}(UPBE-D3(Acetonitrile)/def2SVP/W06) = -2806.79197965 $\langle S^2 \rangle$ = 6.0744

Fe	-0.000000	0.000000	0.736880
O	-0.467121	1.610901	-0.193349
O	0.467121	-1.610901	-0.193349
N	-0.955711	-1.085346	2.158969
N	0.955711	1.085346	2.158969
C	1.117581	2.385956	2.137194
H	1.587281	2.869143	3.022758
C	0.761872	3.269953	1.051879
C	1.181010	4.627340	1.155364
H	1.739164	4.913147	2.059407
C	0.914678	5.562165	0.158851
C	0.201567	5.097228	-0.982436
H	-0.000532	5.821336	-1.783580
C	-0.259353	3.788300	-1.151065
C	-0.000000	2.832842	-0.098163
C	-0.000000	-2.832842	-0.098163
C	0.259353	-3.788300	-1.151065
C	-0.201567	-5.097228	-0.982436
H	0.000532	-5.821336	-1.783580
C	-0.914678	-5.562165	0.158851
C	-1.181010	-4.627340	1.155364
H	-1.739164	-4.913147	2.059407
C	-0.761872	-3.269953	1.051879
C	-1.117581	-2.385956	2.137194

H	-1.587281	-2.869143	3.022758
C	-1.263732	-0.327487	3.368606
H	-1.995874	-0.869032	4.008498
H	-1.743990	0.621704	3.050423
C	1.263732	0.327487	3.368606
H	1.995874	0.869032	4.008498
H	1.743990	-0.621704	3.050423
C	-1.021575	3.353332	-2.421043
C	-2.450591	2.892272	-2.040792
C	-0.260004	2.195868	-3.115840
H	0.753341	2.525131	-3.425881
H	-0.153796	1.324991	-2.442584
H	-0.805417	1.871062	-4.026737
H	-2.418464	2.047738	-1.328060
H	-3.003049	2.568518	-2.947941
H	-3.018655	3.724224	-1.575338
C	-1.154544	4.502802	-3.439177
H	-0.166849	4.876722	-3.778703
H	-1.698665	4.139914	-4.334689
H	-1.725962	5.360957	-3.029781
C	1.351607	7.037178	0.234291
C	2.285823	7.358897	-0.956802
C	0.100919	7.946075	0.164889
C	2.105491	7.349663	1.539905
H	3.197118	6.727057	-0.926387
H	1.786678	7.186720	-1.931686
H	2.603073	8.422265	-0.926509
H	-0.469661	7.792448	-0.773195
H	0.394144	9.015922	0.207238
H	-0.582938	7.743002	1.014442
H	2.399917	8.418565	1.558645
H	1.477132	7.160710	2.434114
H	3.031492	6.746915	1.636260
C	1.021575	-3.353332	-2.421043
C	1.154544	-4.502802	-3.439177
C	0.260004	-2.195868	-3.115840
C	2.450591	-2.892272	-2.040792
H	1.725962	-5.360957	-3.029781
H	0.166849	-4.876722	-3.778703
H	1.698665	-4.139914	-4.334689
H	-0.753341	-2.525131	-3.425881
H	0.805417	-1.871062	-4.026737
H	0.153796	-1.324991	-2.442584
H	3.003049	-2.568518	-2.947941
H	2.418464	-2.047738	-1.328060
H	3.018655	-3.724224	-1.575338
C	-1.351607	-7.037178	0.234291
C	-2.285823	-7.358897	-0.956802
C	-0.100919	-7.946075	0.164889
C	-2.105491	-7.349663	1.539905
H	-3.197118	-6.727057	-0.926387
H	-1.786678	-7.186720	-1.931686
H	-2.603073	-8.422265	-0.926509
H	0.469661	-7.792448	-0.773195
H	-0.394144	-9.015922	0.207238
H	0.582938	-7.743002	1.014442
H	-2.399917	-8.418565	1.558645
H	-1.477132	-7.160710	2.434114
H	-3.031492	-6.746915	1.636260
C	-0.000000	0.000000	4.199756
H	0.246684	-0.856772	4.860625
H	-0.246684	0.856772	4.860625

89

^{5}2c′-mono E_{tot}(UPBE-D3(Acetonitrile)/def2SVP/W06) = -2846.02536974 $\langle S^2 \rangle$ = 6.0787

Fe	0.059084	1.061171	0.553011
O	1.284117	-0.303365	-0.001191
O	-1.614714	0.409476	1.227931
N	-0.923651	2.105710	-0.883247
N	1.545254	2.351504	0.984731
C	2.808412	2.080971	0.730831
H	3.542458	2.901795	0.882457
C	3.381324	0.834805	0.280988
C	4.798138	0.783341	0.172215
H	5.359506	1.695933	0.432696
C	5.475860	-0.365547	-0.235862
C	4.679787	-1.499235	-0.549505

H	5.192282	-2.413232	-0.870651
C	3.280434	-1.527140	-0.479697
C	2.592720	-0.331199	-0.059174
C	-2.724737	0.181022	0.562603
C	-3.652884	-0.824427	1.024872
C	-4.856200	-0.980616	0.330114
H	-5.559959	-1.745605	0.685877
C	-5.235960	-0.217509	-0.810020
C	-4.319574	0.726503	-1.265632
H	-4.537833	1.339974	-2.152494
C	-3.063245	0.930100	-0.627415
C	-2.176384	1.909419	-1.211652
H	-2.609381	2.534081	-2.024466
C	-0.186651	3.221096	-1.468834
H	-0.583088	3.474963	-2.478031
H	0.867170	2.899876	-1.599587
C	1.222960	3.704128	1.459336
H	2.042249	4.406743	1.182611
H	1.185215	3.678490	2.571415
C	-0.255637	4.474317	-0.564500
H	-1.236291	4.967063	-0.737422
H	0.520623	5.192416	-0.906460
C	-0.121692	4.231825	0.947759
H	-0.310747	5.192963	1.469908
H	-0.934417	3.550424	1.286363
C	-3.296983	-1.704775	2.241368
C	-3.141401	-0.818570	3.501964
C	-1.975167	-2.463751	1.959210
C	-4.385833	-2.754773	2.536470
H	-4.094388	-0.299507	3.734045
H	-2.353801	-0.056456	3.357003
H	-2.871921	-1.443552	4.379306
H	-1.146176	-1.762609	1.750799
H	-1.696459	-3.088224	2.833809
H	-2.088364	-3.136594	1.083984
H	-4.078792	-3.368024	3.407866
H	-4.542873	-3.445170	1.682461
H	-5.360742	-2.288429	2.786806
C	-6.599989	-0.473086	-1.478091
C	-6.830300	0.448172	-2.690060
C	-7.729587	-0.219023	-0.451307
C	-6.667690	-1.941434	-1.962356
H	-6.066334	0.291679	-3.478674
H	-6.814096	1.519271	-2.402642
H	-7.821732	0.238152	-3.140099
H	-7.710754	0.831157	-0.094372
H	-8.722505	-0.408868	-0.909966
H	-7.638470	-0.879436	0.434536
H	-7.648030	-2.148983	-2.440009
H	-6.544920	-2.658513	-1.125755
H	-5.871538	-2.149265	-2.706357
C	2.479505	-2.796735	-0.840128
C	1.527143	-2.497126	-2.024808
C	1.660083	-3.262858	0.389208
C	3.396814	-3.961936	-1.260481
H	2.103887	-2.193533	-2.923008
H	0.819606	-1.686107	-1.771849
H	0.943655	-3.405009	-2.286017
H	0.957067	-2.477641	0.722720
H	1.073877	-4.171836	0.137663
H	2.332507	-3.515350	1.235089
H	2.776465	-4.847361	-1.507296
H	4.092993	-4.260210	-0.449964
H	3.998869	-3.717428	-2.159558
C	7.016039	-0.374872	-0.330125
C	7.617181	-0.061089	1.060730
C	7.474626	0.702600	-1.341453
C	7.563041	-1.737724	-0.795506
H	7.307756	-0.822156	1.806235
H	7.292238	0.930610	1.434750
H	8.726465	-0.055955	1.014807
H	7.143678	1.716726	-1.039799
H	8.582036	0.719606	-1.418909
H	7.062693	0.498278	-2.351037
H	8.669928	-1.698535	-0.848949
H	7.190552	-2.011690	-1.803583
H	7.292280	-2.553996	-0.095198

87

^{5}2d′-mono E_{tot}(UPBE-D3(Acetonitrile)/def2SVP/W06) = -2919.71128645 $\langle S^2 \rangle$ = 6.0818

Fe	0.000938	0.595290	0.438690
O	1.520087	-0.578785	0.480228
O	-1.528801	-0.565305	0.542949
N	-1.320006	2.183021	0.233091
N	1.331405	2.179556	0.249828
C	2.612786	2.052198	-0.056808
H	3.181707	2.961942	-0.338048
C	3.371008	0.835585	-0.087454
C	4.757629	0.937856	-0.412490
H	5.154139	1.944431	-0.611886
C	5.584513	-0.177604	-0.470438
C	4.982000	-1.442996	-0.204704
H	5.626884	-2.330976	-0.254140
C	3.633404	-1.630148	0.106318
C	2.786845	-0.460802	0.182486
C	-2.789915	-0.448309	0.220887
C	-3.646540	-1.612195	0.178011
C	-4.986286	-1.427164	-0.170724
H	-5.637955	-2.311162	-0.197442
C	-5.571980	-0.169074	-0.500845
C	-4.737467	0.941530	-0.466969
H	-5.121470	1.942991	-0.711471
C	-3.358977	0.841485	-0.108541
C	-2.595361	2.054888	-0.097088
H	-3.153548	2.960608	-0.411490
C	-0.709260	3.445392	0.332858
C	0.722436	3.443887	0.346104
C	3.040476	-3.034617	0.344239
C	1.961889	-3.316511	-0.731107
C	2.410600	-3.117374	1.757079
H	3.176961	-2.936371	2.539046
H	1.602606	-2.372853	1.878364
H	1.985352	-4.129096	1.925133
H	1.161075	-2.555135	-0.699447
H	1.501025	-4.312650	-0.563545
H	2.410716	-3.313803	-1.745801
C	4.109808	-4.139636	0.242529
H	4.911571	-4.015605	0.999215
H	3.636052	-5.126629	0.418477
H	4.583631	-4.173831	-0.759837
C	7.085921	-0.109137	-0.805787
C	7.901497	-0.685016	0.376693
C	7.365425	-0.945147	-2.077920
C	7.554863	1.334835	-1.061478
H	7.726064	-0.097761	1.301298
H	7.633653	-1.739671	0.589109
H	8.987799	-0.654683	0.150547
H	7.080079	-2.008233	-1.945512
H	8.445838	-0.917802	-2.331036
H	6.798878	-0.547443	-2.944752
H	8.638154	1.342737	-1.297864
H	7.023034	1.795613	-1.918725
H	7.402261	1.981423	-0.173402
C	-3.075081	-3.009282	0.497957
C	-4.151552	-4.108765	0.413196
C	-1.963665	-3.352669	-0.524531
C	-2.493100	-3.028723	1.933470
H	-4.975837	-3.943283	1.137017
H	-4.593409	-4.185312	-0.601388
H	-3.693353	-5.090410	0.649778
H	-2.375055	-3.385826	-1.554505
H	-1.526752	-4.348242	-0.299094
H	-1.151487	-2.603690	-0.494564
H	-2.079094	-4.033052	2.162830
H	-1.685110	-2.282714	2.046805
H	-3.283754	-2.808001	2.680222
C	-7.064963	-0.102039	-0.872120
C	-7.323260	-0.984495	-2.117033
C	-7.911726	-0.625116	0.312980
C	-7.515649	1.334555	-1.193784
H	-6.734550	-0.624672	-2.985637
H	-7.049461	-2.043684	-1.937425
H	-8.397587	-0.959033	-2.395074
H	-7.658333	-1.672927	0.571875
H	-8.992418	-0.594547	0.061405

H	-7.751539	-0.003922	1.217969
H	-8.593158	1.341570	-1.455262
H	-7.377858	2.013740	-0.327891
H	-6.960513	1.757922	-2.055538
C	1.412448	4.664986	0.508993
C	-1.400066	4.668046	0.481538
C	0.709471	5.871553	0.630531
C	-0.697061	5.873098	0.616422
H	2.510511	4.672245	0.564706
H	-2.498966	4.676684	0.516905
H	1.263697	6.813493	0.759325
H	-1.251632	6.816292	0.734130

35

^{3}2a-mono E_{tot}(UPBE0-D3(Acetonitrile)/def2TZVP/W06) = -2141.01835217 $\langle S^2 \rangle$ = 2.0350

Fe	-0.000000	0.000000	0.225527
O	-0.000000	1.298167	-1.155190
O	-0.000000	-1.298167	-1.155190
N	-0.157348	-1.270587	1.607206
N	0.157348	1.270587	1.607206
C	0.170785	2.582617	1.481283
H	0.276530	3.190315	2.402332
C	0.065307	3.293343	0.241540
C	0.051063	4.719597	0.263573
H	0.115547	5.225685	1.240821
C	-0.041585	5.467068	-0.904922
H	-0.052137	6.566292	-0.865532
C	-0.121279	4.792500	-2.150717
H	-0.194335	5.374381	-3.083361
C	-0.107189	3.402352	-2.210608
H	-0.167133	2.870703	-3.172584
C	-0.013693	2.599350	-1.029325
C	0.013693	-2.599350	-1.029325
C	0.107189	-3.402352	-2.210608
H	0.167133	-2.870703	-3.172584
C	0.121279	-4.792500	-2.150717
H	0.194335	-5.374381	-3.083361
C	0.041585	-5.467068	-0.904922
H	0.052137	-6.566292	-0.865532
C	-0.051063	-4.719597	0.263573
H	-0.115547	-5.225685	1.240821
C	-0.065307	-3.293343	0.241540
C	-0.170785	-2.582617	1.481283
H	-0.276530	-3.190315	2.402332
C	-0.370765	-0.665016	2.929474
H	-0.027379	-1.330423	3.749320
H	-1.460583	-0.489292	3.066441
C	0.370765	0.665016	2.929474
H	0.027379	1.330423	3.749320
H	1.460583	0.489292	3.066441

38

^{3}2b-mono E_{tot}(UPBE0-D3(Acetonitrile)/def2TZVP/W06) = -2180.28715408 $\langle S^2 \rangle$ = 2.0225

Fe	-0.000000	-0.000000	-0.059661
O	0.352686	1.307330	1.258898
O	-0.352686	-1.307330	1.258898
N	0.656258	-1.222404	-1.351098
N	-0.656258	1.222404	-1.351098
C	-0.644278	2.533106	-1.229864
H	-1.009349	3.131496	-2.089503
C	-0.241633	3.265755	-0.062341
C	-0.330337	4.688260	-0.089948
H	-0.674417	5.170415	-1.019558
C	-0.000000	5.463382	1.017053
C	0.429196	4.818538	2.203797
H	0.693756	5.420309	3.087874
C	0.523578	3.430356	2.268430
C	0.208643	2.603747	1.145787
C	-0.208643	-2.603747	1.145787
C	-0.523578	-3.430356	2.268430
C	-0.429196	-4.818538	2.203797
H	-0.693756	-5.420309	3.087874
C	0.000000	-5.463382	1.017053
C	0.330337	-4.688260	-0.089948
H	0.674417	-5.170415	-1.019558
C	0.241633	-3.265755	-0.062341

C	0.644278	-2.533106	-1.229864
H	1.009349	-3.131496	-2.089503
C	1.136980	-0.615103	-2.596046
H	1.699061	-1.356907	-3.202435
H	1.854527	0.181136	-2.309120
C	-1.136980	0.615103	-2.596046
H	-1.699061	1.356907	-3.202435
H	-1.854527	-0.181136	-2.309120
H	0.859608	2.922656	3.185313
H	-0.074480	6.559839	0.973865
H	-0.859608	-2.922656	3.185313
H	0.074480	-6.559839	0.973865
C	-0.000000	-0.000000	-3.436469
H	-0.446090	-0.771880	-4.096147
H	0.446090	0.771880	-4.096147

41

³2c-mono E_{tot}(UPBE0-D3(Acetonitrile)/def2TZVP/W06) = -2219.56212037 $\langle S^2 \rangle$ = 2.0269

Fe	-0.038626	0.038098	-0.192960
O	-1.247260	-1.395536	0.032134
O	1.294791	-1.140959	-0.802817
N	1.133588	1.088589	0.858089
N	-1.364084	1.354190	-0.645796
C	-2.649130	1.143830	-0.429831
H	-3.335133	1.998160	-0.605408
C	-3.285543	-0.084804	-0.043936
C	-4.702515	-0.090745	0.112805
H	-5.245529	0.859706	-0.016761
C	-5.399932	-1.257077	0.412291
C	-4.682647	-2.470678	0.554702
H	-5.223374	-3.400901	0.791139
C	-3.298092	-2.503807	0.402042
C	-2.551098	-1.320181	0.118212
C	2.548502	-1.202803	-0.430634
C	3.368123	-2.260450	-0.931873
C	4.712853	-2.365451	-0.584565
H	5.315570	-3.190193	-0.996999
C	5.312377	-1.425970	0.291957
C	4.539061	-0.389975	0.805012
H	4.987670	0.349888	1.487912
C	3.160643	-0.247658	0.468379
C	2.417461	0.836002	1.040323
H	2.978988	1.515577	1.713162
C	0.603691	2.332647	1.432150
H	1.129199	2.573836	2.381521
H	-0.462279	2.164938	1.681525
C	-1.023683	2.659877	-1.255588
H	-1.748644	3.427381	-0.902990
H	-1.179500	2.557509	-2.351813
H	-2.732243	-3.440867	0.515947
H	-6.493271	-1.238877	0.530306
H	2.894822	-2.985283	-1.611501
H	6.374412	-1.516909	0.563308
C	0.753859	3.498495	0.436238
H	1.805123	3.856655	0.463231
H	0.122710	4.343631	0.785409
C	0.404687	3.137011	-1.012614
H	0.562869	4.029674	-1.652353
H	1.114417	2.368453	-1.389710

39

³2d-mono E_{tot}(UPBE0-D3(Acetonitrile)/def2TZVP/W06) = -2293.32976286 $\langle S^2 \rangle$ = 2.0333

Fe	0.000000	0.000000	0.386154
O	-0.000000	1.275367	1.783741
O	-0.000000	-1.275367	1.783741
N	0.000000	-1.283723	-1.011980
N	0.000000	1.283723	-1.011980
C	-0.000000	2.602503	-0.841266
H	-0.000000	3.247597	-1.737934
C	-0.000000	3.282393	0.410448
C	-0.000000	4.713023	0.409214
H	-0.000000	5.232060	-0.563097
C	-0.000000	5.442341	1.589514
C	-0.000000	4.751160	2.831271
H	-0.000000	5.322374	3.773156
C	-0.000000	3.361837	2.873999

C	-0.000000	2.574911	1.678012
C	-0.000000	-2.574911	1.678012
C	-0.000000	-3.361837	2.873999
C	-0.000000	-4.751160	2.831271
H	-0.000000	-5.322374	3.773156
C	-0.000000	-5.442341	1.589514
C	-0.000000	-4.713023	0.409214
H	-0.000000	-5.232060	-0.563097
C	-0.000000	-3.282393	0.410448
C	-0.000000	-2.602503	-0.841266
H	-0.000000	-3.247597	-1.737934
C	0.000000	-0.710692	-2.310703
C	0.000000	0.710692	-2.310703
H	-0.000000	2.817423	3.830416
H	-0.000000	6.541937	1.566366
H	-0.000000	-2.817423	3.830416
H	-0.000000	-6.541937	1.566366
C	-0.000000	1.410119	-3.533470
C	-0.000000	-1.410119	-3.533470
C	-0.000000	0.702995	-4.744530
C	-0.000000	-0.702995	-4.744530
H	-0.000000	2.508435	-3.554547
H	-0.000000	-2.508435	-3.554547
H	-0.000000	1.256450	-5.695364
H	-0.000000	-1.256450	-5.695364

83

^{3}2a′-mono E_{tot}(UPBE0-D3(Acetonitrile)/def2TZVP/W06) = -2769.50089993 $\langle S^2 \rangle$ = 2.0339

Fe	0.000000	-0.000000	1.278935
O	0.004458	1.320983	-0.083636
O	-0.004458	-1.320983	-0.083636
N	-0.182742	-1.265874	2.654859
N	0.182742	1.265874	2.654859
C	0.237619	2.576758	2.523725
H	0.374739	3.178198	3.444857
C	0.131454	3.302105	1.292889
C	0.160957	4.726967	1.358331
H	0.272811	5.183126	2.353094
C	0.050089	5.515414	0.219709
C	-0.105087	4.831728	-1.022234
H	-0.205782	5.445603	-1.927599
C	-0.139364	3.443640	-1.168048
C	0.000000	2.626457	0.021287
C	-0.000000	-2.626457	0.021287
C	0.139364	-3.443640	-1.168048
C	0.105087	-4.831728	-1.022234
H	0.205782	-5.445603	-1.927599
C	-0.050089	-5.515414	0.219709
C	-0.160957	-4.726967	1.358331
H	-0.272811	-5.183126	2.353094
C	-0.131454	-3.302105	1.292889
C	-0.237619	-2.576758	2.523725
H	-0.374739	-3.178198	3.444857
C	-0.385418	-0.656812	3.977224
H	-0.056568	-1.329831	4.796996
H	-1.471172	-0.457003	4.114688
C	0.385418	0.656812	3.977224
H	0.056568	1.329831	4.796996
H	1.471172	0.457003	4.114688
C	-0.329346	2.785121	-2.550153
C	-1.611343	1.916019	-2.531792
C	0.903922	1.910928	-2.883412
H	1.818316	2.536220	-2.951189
H	1.060181	1.138798	-2.109651
H	0.764133	1.403442	-3.861248
H	-1.551912	1.141936	-1.745442
H	-1.748130	1.411665	-3.511290
H	-2.505762	2.545451	-2.343583
C	-0.485487	3.825400	-3.676544
H	0.410277	4.472048	-3.777453
H	-0.625014	3.299906	-4.643103
H	-1.368126	4.479908	-3.523707
C	0.082317	7.054796	0.240451
C	1.270137	7.553113	-0.617468
C	-1.240008	7.605146	-0.345792
C	0.248017	7.609277	1.667144
H	2.233602	7.181234	-0.212423

H	1.191031	7.212375	-1.669527
H	1.305757	8.662623	-0.626204
H	-1.401047	7.269056	-1.389950
H	-1.231827	8.715197	-0.349109
H	-2.109227	7.269443	0.256172
H	0.268356	8.717656	1.641025
H	-0.590119	7.306210	2.327280
H	1.194535	7.268904	2.134389
C	0.329346	-2.785121	-2.550153
C	0.485487	-3.825400	-3.676544
C	-0.903922	-1.910928	-2.883412
C	1.611343	-1.916019	-2.531792
H	1.368126	-4.479908	-3.523707
H	-0.410277	-4.472048	-3.777453
H	0.625014	-3.299906	-4.643103
H	-1.818316	-2.536220	-2.951189
H	-0.764133	-1.403442	-3.861248
H	-1.060181	-1.138798	-2.109651
H	1.748130	-1.411665	-3.511290
H	1.551912	-1.141936	-1.745442
H	2.505762	-2.545451	-2.343583
C	-0.082317	-7.054796	0.240451
C	-1.270137	-7.553113	-0.617468
C	1.240008	-7.605146	-0.345792
C	-0.248017	-7.609277	1.667144
H	-2.233602	-7.181234	-0.212423
H	-1.191031	-7.212375	-1.669527
H	-1.305757	-8.662623	-0.626204
H	1.401047	-7.269056	-1.389950
H	1.231827	-8.715197	-0.349109
H	2.109227	-7.269443	0.256172
H	-0.268356	-8.717656	1.641025
H	0.590119	-7.306210	2.327280
H	-1.194535	-7.268904	2.134389

86

[3]2b′-mono E_{tot}(UPBE0-D3(Acetonitrile)/def2TZVP/W06) = -2808.77014589 $\langle S^2 \rangle$ = 2.0218

Fe	-0.000000	0.000000	1.167829
O	-0.217020	1.324852	-0.157400
O	0.217020	-1.324852	-0.157400
N	-0.701961	-1.197662	2.455338
N	0.701961	1.197662	2.455338
C	0.743652	2.507482	2.322527
H	1.108913	3.094636	3.190149
C	0.419209	3.257612	1.141950
C	0.584920	4.672508	1.196235
H	0.901432	5.106611	2.156072
C	0.368433	5.479521	0.084536
C	-0.009524	4.821566	-1.121366
H	-0.168394	5.447121	-2.010335
C	-0.187373	3.441638	-1.254060
C	0.000000	2.615361	-0.081812
C	-0.000000	-2.615361	-0.081812
C	0.187373	-3.441638	-1.254060
C	0.009524	-4.821566	-1.121366
H	0.168394	-5.447121	-2.010335
C	-0.368433	-5.479521	0.084536
C	-0.584920	-4.672508	1.196235
H	-0.901432	-5.106611	2.156072
C	-0.419209	-3.257612	1.141950
C	-0.743652	-2.507482	2.322527
H	-1.108913	-3.094636	3.190149
C	-1.154061	-0.584819	3.708178
H	-1.730008	-1.315768	4.315112
H	-1.855122	0.229976	3.432218
C	1.154061	0.584819	3.708178
H	1.730008	1.315768	4.315112
H	1.855122	-0.229976	3.432218
C	-0.560229	2.800967	-2.607449
C	-1.912682	2.055373	-2.489569
C	0.557269	1.811843	-3.023274
H	1.523845	2.343684	-3.143473
H	0.681421	1.013307	-2.269592
H	0.306788	1.335871	-3.994502
H	-1.865535	1.269249	-1.714306
H	-2.172163	1.579967	-3.458846
H	-2.727306	2.762361	-2.228106

C	-0.700271	3.850868	-3.727050
H	0.242534	4.409991	-3.897929
H	-0.960263	3.341105	-4.676905
H	-1.503608	4.585940	-3.514450
C	0.531955	7.010681	0.096789
C	1.623921	7.418607	-0.921278
C	-0.810216	7.672411	-0.297680
C	0.942336	7.535620	1.484759
H	2.600548	6.963728	-0.656941
H	1.368879	7.098221	-1.951551
H	1.750302	8.521360	-0.935209
H	-1.144650	7.358721	-1.307104
H	-0.710981	8.777999	-0.306405
H	-1.610094	7.404500	0.422732
H	1.045785	8.639205	1.454796
H	0.185527	7.291842	2.258015
H	1.916276	7.118318	1.812435
C	0.560229	-2.800967	-2.607449
C	0.700271	-3.850868	-3.727050
C	-0.557269	-1.811843	-3.023274
C	1.912682	-2.055373	-2.489569
H	1.503608	-4.585940	-3.514450
H	-0.242534	-4.409991	-3.897929
H	0.960263	-3.341105	-4.676905
H	-1.523845	-2.343684	-3.143473
H	-0.306788	-1.335871	-3.994502
H	-0.681421	-1.013307	-2.269592
H	2.172163	-1.579967	-3.458846
H	1.865535	-1.269249	-1.714306
H	2.727306	-2.762361	-2.228106
C	-0.531955	-7.010681	0.096789
C	-1.623921	-7.418607	-0.921278
C	0.810216	-7.672411	-0.297680
C	-0.942336	-7.535620	1.484759
H	-2.600548	-6.963728	-0.656941
H	-1.368879	-7.098221	-1.951551
H	-1.750302	-8.521360	-0.935209
H	1.144650	-7.358721	-1.307104
H	0.710981	-8.777999	-0.306405
H	1.610094	-7.404500	0.422732
H	-1.045785	-8.639205	1.454796
H	-0.185527	-7.291842	2.258015
H	-1.916276	-7.118318	1.812435
C	-0.000000	0.000000	4.546323
H	0.425525	-0.783013	5.206588
H	-0.425525	0.783013	5.206588

89

[3]2c′-mono E_{tot}(UPBE0-D3(Acetonitrile)/def2TZVP/W06) = -2848.04474497 $\langle S^2 \rangle$ = 2.0247

Fe	0.059760	1.089604	0.300532
O	1.282208	-0.335474	0.084439
O	-1.323117	-0.149050	0.605201
N	-1.045160	2.284969	-0.666056
N	1.375298	2.329074	0.953352
C	2.660811	2.136638	0.728359
H	3.343720	2.972859	0.985844
C	3.310953	0.948091	0.244694
C	4.722482	0.992762	0.087683
H	5.225689	1.954935	0.277666
C	5.462854	-0.130455	-0.282886
C	4.738736	-1.337218	-0.468158
H	5.301965	-2.236041	-0.743846
C	3.349600	-1.461472	-0.320213
C	2.591436	-0.280923	0.010304
C	-2.578862	-0.127073	0.229296
C	-3.446233	-1.239881	0.547052
C	-4.786322	-1.158005	0.159630
H	-5.446569	-1.997807	0.415469
C	-5.361264	-0.063235	-0.549144
C	-4.512077	0.988494	-0.874033
H	-4.882343	1.863377	-1.428398
C	-3.135205	0.984308	-0.502656
C	-2.333135	2.103499	-0.898939
H	-2.854982	2.890390	-1.481142
C	-0.468066	3.571571	-1.080305
H	-0.947838	3.922534	-2.019620
H	0.603882	3.407843	-1.304510

C	1.031956	3.562610	1.695123
H	1.793952	4.343634	1.473938
H	1.126860	3.327745	2.777981
C	-0.637465	4.632000	0.024268
H	-1.676533	5.023384	-0.012738
H	0.031062	5.489459	-0.205055
C	-0.368609	4.111707	1.441390
H	-0.527289	4.939918	2.162744
H	-1.120747	3.336943	1.706478
C	-2.891600	-2.478022	1.280734
C	-2.314859	-2.067994	2.658445
C	-1.782357	-3.120654	0.412360
C	-3.977799	-3.543225	1.526833
H	-3.106912	-1.627523	3.299132
H	-1.502414	-1.326943	2.545839
H	-1.909839	-2.958919	3.182851
H	-0.964988	-2.403187	0.217746
H	-1.353464	-4.005934	0.927168
H	-2.194579	-3.459566	-0.560725
H	-3.527562	-4.411902	2.048720
H	-4.419587	-3.917758	0.580764
H	-4.801383	-3.162396	2.165268
C	-6.856125	-0.097556	-0.917422
C	-7.290257	1.164824	-1.684554
C	-7.699617	-0.196501	0.376337
C	-7.137867	-1.330623	-1.809149
H	-6.736124	1.278649	-2.638521
H	-7.136545	2.085204	-1.085089
H	-8.369653	1.103560	-1.930951
H	-7.523897	0.681632	1.031018
H	-8.782102	-0.233467	0.133198
H	-7.456197	-1.107634	0.959187
H	-8.214103	-1.379069	-2.076690
H	-6.877611	-2.279123	-1.297571
H	-6.551509	-1.280814	-2.749501
C	2.639602	-2.819423	-0.498074
C	1.599094	-2.731499	-1.642076
C	1.944266	-3.197240	0.833678
C	3.625410	-3.951129	-0.849058
H	2.099421	-2.504063	-2.606308
H	0.851290	-1.943838	-1.438989
H	1.069625	-3.701093	-1.753133
H	1.216353	-2.422772	1.136727
H	1.402333	-4.160183	0.726680
H	2.694293	-3.315025	1.642997
H	3.067076	-4.903354	-0.955455
H	4.390139	-4.101770	-0.059680
H	4.151349	-3.766233	-1.808223
C	6.992709	-0.038428	-0.458696
C	7.637237	0.409849	0.874613
C	7.318856	0.998054	-1.560261
C	7.614136	-1.387992	-0.865800
H	7.421645	-0.318854	1.682787
H	7.260447	1.399763	1.201832
H	8.739432	0.488838	0.767796
H	6.929208	2.004260	-1.305844
H	8.416661	1.087633	-1.699200
H	6.873114	0.697657	-2.530711
H	8.710691	-1.274576	-0.984961
H	7.210517	-1.755674	-1.831169
H	7.442091	-2.171648	-0.100089

87

^{3}2d′-mono E_{tot}(UPBE0-D3(Acetonitrile)/def2TZVP/W06) = -2921.81343020 $\langle S^2 \rangle$ = 2.0325

Fe	0.000000	0.871163	0.000000
O	1.291959	-0.502875	0.154227
O	-1.291959	-0.502875	-0.154227
N	-1.281330	2.262756	0.013500
N	1.281330	2.262756	-0.013500
C	2.597958	2.084457	-0.082907
H	3.238823	2.979763	-0.174061
C	3.287077	0.838767	-0.039525
C	4.714507	0.875183	-0.126352
H	5.185888	1.861606	-0.246641
C	5.481107	-0.278471	-0.059116
C	4.779179	-1.509905	0.118212
H	5.379541	-2.427217	0.184939

C	3.392799	-1.630492	0.212553
C	2.595010	-0.424669	0.109821
C	-2.595010	-0.424669	-0.109821
C	-3.392799	-1.630492	-0.212553
C	-4.779179	-1.509905	-0.118212
H	-5.379541	-2.427217	-0.184939
C	-5.481107	-0.278471	0.059116
C	-4.714507	0.875183	0.126352
H	-5.185888	1.861606	0.246641
C	-3.287077	0.838767	0.039525
C	-2.597958	2.084457	0.082907
H	-3.238823	2.979763	0.174061
C	-0.711035	3.561347	0.002686
C	0.711035	3.561347	-0.002686
C	2.715108	-2.999651	0.423256
C	1.762552	-3.299505	-0.759647
C	1.924091	-2.976494	1.754806
H	2.609384	-2.813610	2.612300
H	1.165029	-2.173131	1.751730
H	1.405643	-3.945596	1.910241
H	0.973885	-2.530667	-0.837871
H	1.273281	-4.285746	-0.616904
H	2.325925	-3.333858	-1.715289
C	3.739207	-4.148383	0.505608
H	4.446864	-4.021036	1.350452
H	3.202502	-5.105690	0.664008
H	4.330228	-4.250240	-0.427731
C	7.017122	-0.290252	-0.160260
C	7.612840	-0.898405	1.132146
C	7.441139	-1.149894	-1.375457
C	7.593849	1.125572	-0.341800
H	7.329810	-0.296030	2.019575
H	7.262524	-1.936593	1.301250
H	8.720932	-0.925019	1.073302
H	7.082379	-2.195265	-1.287889
H	8.547339	-1.181643	-1.461490
H	7.034588	-0.729653	-2.318133
H	8.699237	1.075806	-0.412837
H	7.221106	1.606748	-1.268931
H	7.343789	1.785721	0.513608
C	-2.715108	-2.999651	-0.423256
C	-3.739207	-4.148383	-0.505608
C	-1.924091	-2.976494	-1.754806
C	-1.762552	-3.299505	0.759647
H	-4.330228	-4.250240	0.427731
H	-4.446864	-4.021036	-1.350452
H	-3.202502	-5.105690	-0.664008
H	-2.609384	-2.813610	-2.612300
H	-1.405643	-3.945596	-1.910241
H	-1.165029	-2.173131	-1.751730
H	-1.273281	-4.285746	0.616904
H	-0.973885	-2.530667	0.837872
H	-2.325925	-3.333858	1.715289
C	-7.017122	-0.290252	0.160260
C	-7.612840	-0.898405	-1.132147
C	-7.441139	-1.149894	1.375456
C	-7.593849	1.125572	0.341800
H	-7.329810	-0.296030	-2.019575
H	-7.262524	-1.936593	-1.301250
H	-8.720932	-0.925019	-1.073302
H	-7.082379	-2.195265	1.287889
H	-8.547339	-1.181643	1.461490
H	-7.034588	-0.729653	2.318133
H	-8.699237	1.075806	0.412837
H	-7.221106	1.606748	1.268931
H	-7.343789	1.785721	-0.513608
C	1.410640	4.783976	0.008123
C	-1.410640	4.783976	-0.008123
C	0.703043	5.995155	0.005728
C	-0.703043	5.995155	-0.005728
H	2.509043	4.803743	0.021471
H	-2.509043	4.803743	-0.021471
H	1.256244	6.946181	0.012433
H	-1.256244	6.946181	-0.012433

35

^{1}2a-mono E_{tot}(RPBE0-D3(Acetonitrile)/def2TZVP/W06) = -2140.95837376 $\langle S^2 \rangle$ = 0.0000

Fe	-0.000000	0.000000	0.187428
O	-0.246463	1.329316	-1.051909
O	0.246463	-1.329316	-1.051909
N	-0.482455	-1.165456	1.513391
N	0.482455	1.165456	1.513391
C	0.688322	2.475570	1.427793
H	0.984094	3.016827	2.346326
C	0.506179	3.239792	0.233934
C	0.752340	4.643763	0.233146
H	1.134993	5.110034	1.155277
C	0.514105	5.419109	-0.897346
H	0.716066	6.500476	-0.878798
C	-0.000000	4.811696	-2.071950
H	-0.191321	5.424161	-2.966757
C	-0.266910	3.445078	-2.101257
H	-0.667646	2.956125	-3.001995
C	-0.004841	2.619612	-0.968955
C	0.004841	-2.619612	-0.968955
C	0.266910	-3.445078	-2.101257
H	0.667646	-2.956125	-3.001995
C	-0.000000	-4.811696	-2.071950
H	0.191321	-5.424161	-2.966757
C	-0.514105	-5.419109	-0.897346
H	-0.716066	-6.500476	-0.878798
C	-0.752340	-4.643763	0.233146
H	-1.134993	-5.110034	1.155277
C	-0.506179	-3.239792	0.233934
C	-0.688322	-2.475570	1.427793
H	-0.984094	-3.016827	2.346326
C	-0.558953	-0.519256	2.833456
H	-0.450796	-1.255174	3.657270
H	-1.544101	-0.016158	2.940709
C	0.558953	0.519256	2.833456
H	0.450796	1.255174	3.657270
H	1.544101	0.016158	2.940709

38

[1]**2b-mono** E_{tot}(RPBE0-D3(Acetonitrile)/def2TZVP/W06) = -2180.23940979 $\langle S^2 \rangle$ = 0.0000

Fe	0.000000	-0.000000	0.065765
O	0.359771	1.436480	1.138438
O	-0.359771	-1.436480	1.138438
N	0.917160	-0.988940	-1.166802
N	-0.917160	0.988940	-1.166802
C	-1.273828	2.267091	-1.081868
H	-1.911628	2.676615	-1.887426
C	-0.856161	3.160768	-0.045645
C	-1.239997	4.532751	-0.077301
H	-1.894026	4.876185	-0.894964
C	-0.796679	5.430882	0.889947
C	0.059259	4.980574	1.925577
H	0.412214	5.687594	2.692446
C	0.458041	3.645079	1.983110
C	-0.000000	2.702650	1.021326
C	-0.000000	-2.702650	1.021326
C	-0.458041	-3.645079	1.983110
C	-0.059259	-4.980574	1.925577
H	-0.412214	-5.687594	2.692446
C	0.796679	-5.430882	0.889947
C	1.239997	-4.532751	-0.077301
H	1.894026	-4.876185	-0.894964
C	0.856161	-3.160768	-0.045645
C	1.273828	-2.267091	-1.081868
H	1.911628	-2.676615	-1.887426
C	1.256786	-0.247348	-2.387889
H	2.025562	-0.789825	-2.977054
H	1.697697	0.721679	-2.079265
C	-1.256786	0.247348	-2.387889
H	-2.025562	0.789825	-2.977054
H	-1.697697	-0.721679	-2.079265
H	1.119155	3.277255	2.782356
H	-1.104591	6.486295	0.847302
H	-1.119155	-3.277255	2.782356
H	1.104591	-6.486295	0.847302
C	-0.000000	0.000000	-3.246129
H	-0.192897	-0.872000	-3.903375
H	0.192897	0.872000	-3.903375

41

[1]2c-mono E_{tot}(RPBE0-D3(Acetonitrile)/def2TZVP/W06) = -2219.52638614 $\langle S^2 \rangle$ = 0.0000

Fe	0.021969	0.167528	-0.636530
O	-0.985816	-1.290512	-0.211208
O	1.542908	-0.718595	-1.224741
N	0.780111	0.851144	0.876879
N	-1.429085	1.333887	-0.791457
C	-2.704876	1.017860	-0.635509
H	-3.465525	1.796222	-0.840292
C	-3.182329	-0.254118	-0.175034
C	-4.571367	-0.442504	0.077143
H	-5.253392	0.405269	-0.098892
C	-5.070086	-1.657179	0.541757
C	-4.177406	-2.733407	0.768794
H	-4.561676	-3.697846	1.136502
C	-2.811296	-2.583744	0.531815
C	-2.278543	-1.354617	0.047070
C	2.700567	-0.864153	-0.626850
C	3.740009	-1.609081	-1.258109
C	4.970167	-1.800518	-0.632815
H	5.751448	-2.389500	-1.138663
C	5.222703	-1.248544	0.649564
C	4.232181	-0.504536	1.282835
H	4.422216	-0.059865	2.273240
C	2.961500	-0.284927	0.672666
C	1.989931	0.532991	1.330265
H	2.283604	0.967911	2.304694
C	0.008008	1.839458	1.645469
H	0.368122	1.863429	2.696132
H	-1.045864	1.505230	1.670100
C	-1.132753	2.727115	-1.182077
H	-2.005670	3.370198	-0.934000
H	-1.005650	2.766996	-2.285913
H	-2.106094	-3.411924	0.699357
H	-6.146571	-1.779057	0.733091
H	3.528713	-2.035870	-2.250444
H	6.196930	-1.403632	1.136592
C	0.128579	3.248599	1.022655
H	1.074360	3.712293	1.374078
H	-0.697552	3.875041	1.422019
C	0.126182	3.281221	-0.511714
H	0.247574	4.331037	-0.850767
H	1.022890	2.746489	-0.894958

39

[1]2d-mono E_{tot}(RPBE0-D3(Acetonitrile)/def2TZVP/W06) = -2293.26944962 $\langle S^2 \rangle$ = 0.0000

Fe	0.374148	-0.193120	-0.000000
O	1.758445	-0.210118	1.283825
O	1.758445	-0.210118	-1.283825
N	-1.007158	0.028234	-1.285991
N	-1.007158	0.028234	1.285991
C	-0.838455	0.210257	2.590330
H	-1.726736	0.417212	3.214758
C	0.413210	0.156971	3.272896
C	0.426681	0.326997	4.691658
H	-0.533729	0.507860	5.201653
C	1.604331	0.262106	5.424628
C	2.828533	0.019529	4.745514
H	3.768039	-0.036135	5.318107
C	2.858872	-0.149155	3.365876
C	1.666119	-0.078829	2.575412
C	1.666119	-0.078829	-2.575412
C	2.858872	-0.149155	-3.365876
C	2.828533	0.019529	-4.745514
H	3.768039	-0.036135	-5.318107
C	1.604331	0.262106	-5.424628
C	0.426681	0.326997	-4.691658
H	-0.533729	0.507860	-5.201653
C	0.413210	0.156971	-3.272896
C	-0.838455	0.210257	-2.590330
H	-1.726736	0.417212	-3.214758
C	-2.299409	-0.011066	-0.712192
C	-2.299409	-0.011066	0.712192
H	3.803540	-0.333131	2.832037
H	1.590971	0.393259	6.516520
H	3.803540	-0.333131	-2.832037
H	1.590971	0.393259	-6.516520

C	-3.522424	-0.070808	1.411438
C	-3.522424	-0.070808	-1.411438
C	-4.731403	-0.114313	0.704023
C	-4.731403	-0.114313	-0.704023
H	-3.538211	-0.100353	2.509691
H	-3.538211	-0.100353	-2.509691
H	-5.681588	-0.164984	1.256399
H	-5.681588	-0.164984	-1.256399

83

^{1}2a′-mono E_{tot}(RPBE0-D3(Acetonitrile)/def2TZVP/W06) = -2769.44297664 $\langle S^2 \rangle$ = 0.0000

Fe	0.000007	-1.278517	0.000294
O	-1.301755	0.003125	-0.213486
O	1.301793	0.003151	0.213883
N	1.218628	-2.600775	-0.320426
N	-1.218702	-2.600795	0.320750
C	-2.543885	-2.501751	0.383992
H	-3.122142	-3.427729	0.566561
C	-3.281201	-1.291423	0.208266
C	-4.702667	-1.327625	0.302356
H	-5.170335	-2.293613	0.541522
C	-5.474767	-0.189103	0.097271
C	-4.787841	1.022545	-0.217480
H	-5.395002	1.924163	-0.372471
C	-3.401809	1.134814	-0.336940
C	-2.609522	-0.052851	-0.112509
C	2.609535	-0.052827	0.112655
C	3.401869	1.134833	0.336966
C	4.787877	1.022562	0.217258
H	5.395072	1.924175	0.372135
C	5.474740	-0.189095	-0.097619
C	4.702599	-1.327606	-0.302592
H	5.170221	-2.293595	-0.541836
C	3.281147	-1.291397	-0.208272
C	2.543804	-2.501712	-0.383926
H	3.122037	-3.427670	-0.566683
C	0.602534	-3.928791	-0.466957
H	1.319388	-4.742450	-0.228384
H	0.267681	-4.060355	-1.518795
C	-0.602608	-3.928798	0.467281
H	-1.319452	-4.742467	0.228714
H	-0.267747	-4.060350	1.519119
C	-2.720834	2.480509	-0.656727
C	-1.891521	2.354179	-1.959065
C	-1.802753	2.878526	0.525658
H	-2.393799	2.989958	1.458204
H	-1.015840	2.121216	0.693249
H	-1.308331	3.850141	0.316775
H	-1.123907	1.564262	-1.868920
H	-1.382087	3.315281	-2.180516
H	-2.549778	2.111230	-2.818803
C	-3.745358	3.613634	-0.860616
H	-4.354636	3.796639	0.048054
H	-3.208144	4.555106	-1.093994
H	-4.435785	3.406719	-1.703901
C	-7.011128	-0.180165	0.193398
C	-7.447204	0.812706	1.297978
C	-7.605166	0.266899	-1.164154
C	-7.578306	-1.569375	0.538077
H	-7.039139	0.511271	2.284393
H	-7.100582	1.844500	1.088245
H	-8.553914	0.840562	1.377202
H	-7.263031	1.281085	-1.452486
H	-8.713594	0.289393	-1.112054
H	-7.312969	-0.433027	-1.973551
H	-8.684301	-1.519420	0.598020
H	-7.319742	-2.323696	-0.232656
H	-7.207383	-1.935228	1.517071
C	2.720921	2.480495	0.656952
C	3.745453	3.613650	0.860630
C	1.802536	2.878522	-0.525195
C	1.891886	2.354047	1.959457
H	4.436063	3.406771	1.703774
H	4.354535	3.796655	-0.048172
H	3.208264	4.555112	1.094105
H	2.393329	2.989996	-1.457897
H	1.308142	3.850119	-0.316161

H	1.015599	2.121196	-0.692599
H	1.382455	3.315111	2.181076
H	1.124297	1.564086	1.869393
H	2.550326	2.111065	2.819045
C	7.011088	-0.180203	-0.193963
C	7.447074	0.812797	-1.298459
C	7.605338	0.266611	1.163577
C	7.578156	-1.569391	-0.538929
H	7.038830	0.511541	-2.284856
H	7.100575	1.844591	-1.088533
H	8.553774	0.840569	-1.377848
H	7.263295	1.280764	1.452128
H	8.713759	0.289062	1.111328
H	7.313220	-0.433435	1.972899
H	8.684143	-1.519468	-0.599051
H	7.319696	-2.323809	0.231744
H	7.207061	-1.935103	-1.517910

86

[1]2b′-mono E_{tot}(RPBE0-D3(Acetonitrile)/def2TZVP/W06) = -2808.72255153 $\langle S^2 \rangle$ = 0.0000

Fe	-0.000000	0.000000	0.931914
O	-0.318618	1.415101	-0.176194
O	0.318618	-1.415101	-0.176194
N	-0.880029	-1.025274	2.160035
N	0.880029	1.025274	2.160035
C	1.199066	2.313253	2.058957
H	1.806195	2.752381	2.872935
C	0.795840	3.184840	0.997969
C	1.158041	4.561784	1.049476
H	1.765317	4.894977	1.903449
C	0.753009	5.458765	0.064874
C	-0.043033	4.946392	-1.001019
H	-0.360379	5.649219	-1.782714
C	-0.440491	3.610539	-1.115990
C	0.000000	2.694889	-0.096072
C	-0.000000	-2.694889	-0.096072
C	0.440491	-3.610539	-1.115990
C	0.043033	-4.946392	-1.001019
H	0.360379	-5.649219	-1.782714
C	-0.753009	-5.458765	0.064874
C	-1.158041	-4.561784	1.049476
H	-1.765317	-4.894977	1.903449
C	-0.795840	-3.184840	0.997969
C	-1.199066	-2.313253	2.058957
H	-1.806195	-2.752381	2.872935
C	-1.244889	-0.308576	3.388358
H	-1.982506	-0.890678	3.979926
H	-1.735972	0.638986	3.088345
C	1.244889	0.308576	3.388358
H	1.982506	0.890678	3.979926
H	1.735972	-0.638986	3.088345
C	-1.294863	3.113429	-2.300665
C	-2.617649	2.504602	-1.772194
C	-0.504828	2.047050	-3.099892
H	0.432751	2.477940	-3.507856
H	-0.242463	1.182063	-2.462996
H	-1.111793	1.681155	-3.954489
H	-2.424410	1.649842	-1.097927
H	-3.238822	2.148476	-2.620585
H	-3.204445	3.265281	-1.216970
C	-1.657461	4.255497	-3.270157
H	-0.758465	4.721434	-3.722831
H	-2.274478	3.852229	-4.098454
H	-2.247129	5.053223	-2.773796
C	1.119569	6.954725	0.081174
C	1.918757	7.299108	-1.198702
C	-0.177025	7.798871	0.120319
C	1.977979	7.327490	1.303756
H	2.858244	6.711425	-1.248499
H	1.337623	7.088029	-2.118791
H	2.185743	8.376433	-1.210648
H	-0.825041	7.603178	-0.757561
H	0.065587	8.882008	0.123003
H	-0.766422	7.576231	1.033331
H	2.220069	8.409221	1.277604
H	1.447538	7.127389	2.256925
H	2.937273	6.771211	1.319685

C	1.294863	-3.113429	-2.300665
C	1.657461	-4.255497	-3.270157
C	0.504828	-2.047050	-3.099892
C	2.617649	-2.504602	-1.772194
H	2.247129	-5.053223	-2.773796
H	0.758465	-4.721434	-3.722831
H	2.274478	-3.852229	-4.098454
H	-0.432751	-2.477940	-3.507856
H	1.111793	-1.681155	-3.954489
H	0.242463	-1.182063	-2.462996
H	3.238822	-2.148476	-2.620585
H	2.424410	-1.649842	-1.097927
H	3.204445	-3.265281	-1.216970
C	-1.119569	-6.954725	0.081174
C	-1.918757	-7.299108	-1.198702
C	0.177025	-7.798871	0.120319
C	-1.977979	-7.327490	1.303756
H	-2.858244	-6.711425	-1.248499
H	-1.337623	-7.088029	-2.118791
H	-2.185743	-8.376433	-1.210648
H	0.825041	-7.603178	-0.757561
H	-0.065587	-8.882008	0.123003
H	0.766422	-7.576231	1.033331
H	-2.220069	-8.409221	1.277604
H	-1.447538	-7.127389	2.256925
H	-2.937273	-6.771211	1.319685
C	-0.000000	0.000000	4.243575
H	0.235463	-0.861080	4.901461
H	-0.235463	0.861080	4.901461

89

^{1}2c′-mono E_{tot}(RPBE0-D3(Acetonitrile)/def2TZVP/W06) = -2848.00846959 $\langle S^2 \rangle$ = 0.0000

Fe	0.129161	1.351898	0.711115
O	1.123887	-0.154122	0.499147
O	-1.433266	0.492802	1.219097
N	-0.511742	1.920584	-0.898781
N	1.605454	2.480635	0.856448
C	2.874337	2.110876	0.772137
H	3.652929	2.876570	0.957012
C	3.331150	0.802308	0.393525
C	4.716813	0.591032	0.169241
H	5.394237	1.446844	0.322982
C	5.219332	-0.643763	-0.248413
C	4.280037	-1.689867	-0.449004
H	4.654483	-2.660770	-0.792034
C	2.898830	-1.558349	-0.240577
C	2.407892	-0.287297	0.217415
C	-2.521265	0.225767	0.536853
C	-3.580859	-0.563930	1.116901
C	-4.704036	-0.825266	0.328390
H	-5.507391	-1.436612	0.761044
C	-4.878753	-0.356250	-1.007775
C	-3.854688	0.412616	-1.550542
H	-3.929200	0.812028	-2.572526
C	-2.674622	0.719155	-0.809958
C	-1.676201	1.541476	-1.421074
H	-1.898766	1.908343	-2.441381
C	0.314772	2.849572	-1.684407
H	0.011225	2.811036	-2.752490
H	1.362758	2.498603	-1.632538
C	1.343324	3.908069	1.127092
H	2.246065	4.504094	0.867545
H	1.171825	4.039641	2.217783
C	0.193463	4.299443	-1.165378
H	-0.727061	4.753089	-1.589724
H	1.047908	4.882691	-1.570831
C	0.130910	4.442875	0.361061
H	0.021807	5.517037	0.618563
H	-0.796622	3.958602	0.738092
C	-3.451714	-1.104335	2.555788
C	-3.328053	0.082068	3.543970
C	-2.200942	-2.012115	2.662039
C	-4.677556	-1.939376	2.973939
H	-4.233969	0.721594	3.502529
H	-2.447319	0.708071	3.308480
H	-3.223904	-0.293608	4.583597
H	-1.282368	-1.454820	2.402418

H	-2.098401	-2.403876	3.695790
H	-2.285816	-2.879953	1.975582
H	-4.538274	-2.302988	4.012238
H	-4.817521	-2.828920	2.326175
H	-5.614813	-1.346397	2.952674
C	-6.167131	-0.714999	-1.771995
C	-6.180741	-0.122383	-3.192936
C	-7.391376	-0.163293	-1.002590
C	-6.283212	-2.254187	-1.885173
H	-5.337873	-0.501489	-3.806003
H	-6.125027	0.985192	-3.178401
H	-7.121387	-0.402415	-3.709049
H	-7.338124	0.941294	-0.914795
H	-8.332167	-0.421327	-1.532331
H	-7.458723	-0.581641	0.021837
H	-7.211356	-2.535420	-2.425295
H	-6.314999	-2.740664	-0.889498
H	-5.420613	-2.675713	-2.440892
C	1.924956	-2.730856	-0.485194
C	0.855067	-2.315577	-1.527143
C	1.236356	-3.125941	0.844679
C	2.648840	-3.978378	-1.029083
H	1.331412	-2.058892	-2.496036
H	0.273496	-1.441696	-1.179859
H	0.151834	-3.155821	-1.706327
H	0.660964	-2.279540	1.262374
H	0.537943	-3.973216	0.678344
H	1.987775	-3.446551	1.595659
H	1.909065	-4.785967	-1.202621
H	3.403824	-4.368988	-0.316527
H	3.156815	-3.778425	-1.994764
C	6.732650	-0.828704	-0.486615
C	7.496415	-0.535862	0.826728
C	7.203464	0.155056	-1.584332
C	7.081975	-2.259275	-0.939141
H	7.182079	-1.232645	1.630694
H	7.317190	0.497689	1.185187
H	8.589967	-0.654890	0.676718
H	7.018633	1.209966	-1.298265
H	8.292546	0.042287	-1.767967
H	6.672606	-0.034988	-2.539741
H	8.176634	-2.346519	-1.093039
H	6.589441	-2.523189	-1.897115
H	6.790512	-3.015890	-0.182463

87

^{1}2d′-mono E_{tot}(RPBE0-D3(Acetonitrile)/def2TZVP/W06) = -2921.75034857 $\langle S^2 \rangle$ = 0.0000

Fe	-0.000126	0.885651	-0.004720
O	1.273756	-0.427568	0.200360
O	-1.274382	-0.426009	-0.216126
N	-1.260448	2.231545	0.149797
N	1.261359	2.231917	-0.151824
C	2.588300	2.082414	-0.216786
H	3.216240	2.983788	-0.317703
C	3.283427	0.844731	-0.124783
C	4.706533	0.857631	-0.217683
H	5.193432	1.826366	-0.400198
C	5.454837	-0.305102	-0.077234
C	4.741436	-1.515213	0.178837
H	5.329949	-2.434621	0.295241
C	3.353286	-1.607322	0.291587
C	2.582543	-0.395897	0.120052
C	-2.582865	-0.395176	-0.129510
C	-3.353709	-1.607817	-0.292043
C	-4.741790	-1.514860	-0.179230
H	-5.330361	-2.434998	-0.289183
C	-5.455241	-0.303347	0.069734
C	-4.706648	0.859623	0.206218
H	-5.193164	1.829004	0.386267
C	-3.283472	0.845814	0.114217
C	-2.587765	2.082977	0.208329
H	-3.215333	2.984842	0.306997
C	-0.699733	3.544115	0.106014
C	0.701466	3.544418	-0.099705
C	2.654922	-2.952766	0.565120
C	1.724446	-3.291272	-0.624684
C	1.842500	-2.855728	1.880503

H	2.516866	-2.661357	2.740008
H	1.093685	-2.044581	1.831049
H	1.311270	-3.811121	2.072111
H	0.950574	-2.515134	-0.758498
H	1.215078	-4.261528	-0.449989
H	2.309214	-3.376991	-1.563860
C	3.661613	-4.109343	0.720743
H	4.360634	-3.946049	1.566479
H	3.108761	-5.048769	0.923492
H	4.261478	-4.270211	-0.198319
C	6.989741	-0.341944	-0.178529
C	7.576847	-0.870686	1.152477
C	7.399838	-1.286745	-1.334124
C	7.585089	1.050837	-0.454122
H	7.301270	-0.207533	1.997789
H	7.215123	-1.891372	1.389303
H	8.684325	-0.913035	1.094942
H	7.031696	-2.320193	-1.175353
H	8.505358	-1.334163	-1.419029
H	6.995420	-0.926736	-2.302182
H	8.689420	0.980324	-0.521917
H	7.218491	1.474273	-1.411286
H	7.346076	1.769711	0.355790
C	-2.655755	-2.955900	-0.553385
C	-3.662511	-4.114268	-0.694609
C	-1.845346	-2.872753	-1.870773
C	-1.723436	-3.281804	0.638652
H	-4.260662	-4.265047	0.227266
H	-4.363046	-3.960452	-1.540863
H	-3.109790	-5.055746	-0.887992
H	-2.520802	-2.687538	-2.731432
H	-1.313978	-3.829906	-2.052950
H	-1.096917	-2.060841	-1.830483
H	-1.215416	-4.254532	0.474069
H	-0.948245	-2.505349	0.763119
H	-2.306858	-3.356467	1.579619
C	-6.990189	-0.339618	0.170279
C	-7.576082	-0.881483	-1.156025
C	-7.400612	-1.273628	1.334501
C	-7.586189	1.055520	0.432004
H	-7.299697	-0.226738	-2.007618
H	-7.214239	-1.904510	-1.382501
H	-8.683618	-0.923199	-1.099092
H	-7.031298	-2.308183	1.185873
H	-8.506172	-1.321319	1.418822
H	-6.997382	-0.903929	2.299391
H	-8.690629	0.985499	0.498389
H	-7.221476	1.487692	1.385969
H	-7.345561	1.766958	-0.383971
C	1.395488	4.761294	-0.202872
C	-1.393028	4.760587	0.218159
C	0.694288	5.976050	-0.096233
C	-0.691239	5.975681	0.119671
H	2.481028	4.783159	-0.368520
H	-2.478382	4.781774	0.385119
H	1.241895	6.926400	-0.181625
H	-1.238279	6.925716	0.211886

42

^{1}**1,3,5-triphenylbenzene** ($^1A_1$ D_3): E_{tot}(RPBE-D3(PCM=Acetonitrile)/def2-SVP) = -923.601618092 $\langle S^2 \rangle$ = 0.0000

C	-1.231558298196	0.711040514986	0.000000000000
C	0.000000000000	1.400933163418	0.000000000000
C	1.231558298196	0.711040514986	0.000000000000
H	0.000000000000	2.500920892937	0.000000000000
C	-2.520769572042	1.455366990983	0.000000000000
C	-2.658937235436	2.672666932092	0.708284608478
C	-3.644066076765	0.966373726910	-0.708284608478
C	-3.874178848973	3.373422174220	0.708922678844
C	-4.858558725051	1.668426214905	-0.708922678844
C	-4.979772374123	2.875072920703	0.000000000000
H	-1.808836817796	3.063573173791	1.288135047874
H	-3.557550603753	0.034712048617	-1.288135047874
H	-3.959540994850	4.313770696067	1.275104028459
H	-5.715605506320	1.272177740833	-1.275104028459
H	-5.933059872549	3.425453714534	0.000000000000
C	1.213243708524	-0.700466581709	0.000000000000
C	0.000000000000	-1.422081029972	0.000000000000

C	-1.213243708524	-0.700466581709	0.000000000000
H	-2.165861026138	-1.250460446468	0.000000000000
C	0.000000000000	-2.910733981967	0.000000000000
C	0.985128841329	-3.639040659002	-0.708284608478
H	1.748713785957	-3.098285222407	-1.288135047874
C	0.984379876078	-5.041848389125	-0.708922678844
H	1.756064511470	-5.585948436900	-1.275104028459
C	0.000000000000	-5.750145841406	0.000000000000
H	0.000000000000	-6.850907429068	0.000000000000
C	-0.984379876078	-5.041848389125	0.708922678844
H	-1.756064511470	-5.585948436900	1.275104028459
C	-0.985128841329	-3.639040659002	0.708284608478
H	-1.748713785957	-3.098285222407	1.288135047874
H	2.165861026138	-1.250460446468	0.000000000000
C	2.520769572042	1.455366990983	0.000000000000
C	2.658937235436	2.672666932092	-0.708284608478
H	1.808836817796	3.063573173791	-1.288135047874
C	3.874178848973	3.373422174220	-0.708922678844
H	3.959540994850	4.313770696067	-1.275104028459
C	4.979772374123	2.875072920703	0.000000000000
H	5.933059872549	3.425453714534	0.000000000000
C	4.858558725051	1.668426214905	0.708922678844
H	5.715605506320	1.272177740833	1.275104028459
C	3.644066076765	0.966373726910	0.708284608478
H	3.557550603753	0.034712048617	1.288135047874

42

[1]1,2,4-triphenylbenzene (^{1}A C_1): E_{tot}(RPBE-D3(PCM=Acetonitrile)/def2-SVP) = -923.598758184 $\langle S^2 \rangle$ = 0.0000

C	-0.256542940913	0.262283765913	-0.009353792277
C	-0.759229796417	-1.072671454864	-0.025680332921
C	0.171965271644	-2.134726037811	-0.072907147661
H	-0.205371667805	-3.168899013225	-0.082279093606
C	-2.212056394898	-1.395540564666	0.048722894861
C	-2.786283766820	-2.326832235972	-0.846599860356
C	-4.148339648324	-2.657360500010	-0.766341004030
C	-4.961204393519	-2.069302815014	0.216787842062
C	-4.400196178246	-1.150224740940	1.120900283529
C	-3.041329379726	-0.815259650229	1.037067081491
H	-2.608971962702	-0.099294280325	1.751935977928
H	-5.026670188027	-0.691783216096	1.901659920812
H	-6.029408470297	-2.327565946997	0.280299848192
H	-4.577667340049	-3.376629611698	-1.480841292016
H	-2.157960757980	-2.781037338866	-1.628526497335
C	-1.146082262784	1.459695581125	-0.037815413277
C	-2.169980688545	1.583681893992	-1.005362295100
C	-0.956942980464	2.517488252351	0.880056904736
C	-2.982460869143	2.725648173503	-1.047459661795
C	-1.774555927227	3.658261310850	0.841610475733
C	-2.791445552574	3.766104000180	-0.121311622736
H	-2.322773071893	0.775165729925	-1.735903461002
H	-0.170572134231	2.432607807813	1.646179045030
H	-3.770400460510	2.805490351628	-1.812297597551
H	-1.617772270509	4.466251145867	1.572856975640
H	-3.433302978355	4.659903117693	-0.151979539989
C	1.552249640111	-1.910592107138	-0.076739033030
C	2.067269392408	-0.595227986168	-0.031176167684
C	1.138780944023	0.467127511472	-0.001168925055
H	1.509123810942	1.503366046834	0.021688472009
C	3.531354316376	-0.336711120020	-0.026120361652
C	4.422539320072	-1.220826749938	0.628147241829
H	4.024264759395	-2.098063158791	1.160575914693
C	5.804210986204	-0.979187751702	0.635489677963
H	6.475809138883	-1.677155548597	1.158883858583
C	6.329227746826	0.152181540410	-0.011899890098
H	7.413518776990	0.341737663978	-0.006348427848
C	5.458025219086	1.039343357563	-0.666647909682
H	5.858246265684	1.924408618918	-1.184936888101
C	4.076297062189	0.797991307617	-0.673711974398
H	3.409999960406	1.488733097847	-1.212694650707
H	2.238293874037	-2.769293348930	-0.129194627449

30

[1]1,4-a-diphenyldiene E_{tot}(RPBE-D3(Acetonitrile)/def2SVP/W06) = -616.89005668 $\langle S^2 \rangle$ = 0.0000

C	-0.230000	3.179879	0.000000
C	1.117757	3.629696	0.000000
C	-1.253519	4.163896	0.000000

C	1.420627	4.996102	0.000000
C	-0.948843	5.532016	0.000000
C	0.390824	5.956456	0.000000
H	1.940665	2.898890	0.000000
H	-2.306030	3.837640	0.000000
H	2.473278	5.319243	0.000000
H	-1.762960	6.273332	0.000000
H	0.633812	7.030081	0.000000
C	-0.606116	1.767017	0.000000
H	-1.693647	1.570566	0.000000
C	0.230000	0.682567	0.000000
H	1.324620	0.832216	0.000000
C	-0.230000	-0.682567	0.000000
H	-1.324620	-0.832216	0.000000
C	0.606116	-1.767017	0.000000
H	1.693647	-1.570566	0.000000
C	0.230000	-3.179879	0.000000
C	1.253519	-4.163896	0.000000
C	-1.117757	-3.629696	0.000000
C	0.948843	-5.532016	0.000000
C	-1.420627	-4.996102	0.000000
C	-0.390824	-5.956456	0.000000
H	2.306030	-3.837640	0.000000
H	-1.940665	-2.898890	0.000000
H	1.762960	-6.273332	0.000000
H	-2.473278	-5.319243	0.000000
H	-0.633812	-7.030081	0.000000

30

[1]1,4-b-diphenyldiene E_{tot}(RPBE-D3(Acetonitrile)/def2SVP/W06) = -616.88483404 $\langle S^2 \rangle$ = 0.0000

C	-2.739997	-0.408764	-0.004567
C	-4.060911	-0.915316	-0.120454
C	-2.584121	0.987502	0.206946
C	-5.175579	-0.070478	-0.027784
C	-3.697694	1.829949	0.298938
C	-5.000299	1.307676	0.182737
H	-4.204945	-1.995007	-0.286221
H	-1.576245	1.419774	0.299770
H	-6.188952	-0.490616	-0.120832
H	-3.550922	2.908746	0.463008
H	-5.873166	1.974235	0.255766
C	-1.613474	-1.335385	-0.108143
H	-1.887143	-2.391200	-0.286970
C	-0.281571	-1.034110	-0.012505
H	0.020678	0.009770	0.169765
C	0.759453	-2.031417	-0.093322
H	0.407912	-3.078124	-0.132295
C	2.119229	-1.848644	-0.071323
H	2.729536	-2.767057	-0.017576
C	2.899076	-0.605161	-0.069464
C	4.186592	-0.611919	0.530421
C	2.460062	0.604982	-0.669712
C	4.976308	0.544361	0.574017
C	3.253942	1.759825	-0.630199
C	4.510720	1.740369	-0.000429
H	4.560089	-1.545691	0.980304
H	1.503041	0.630626	-1.209933
H	5.965717	0.512030	1.055976
H	2.892473	2.682802	-1.109578
H	5.131516	2.648886	0.027873

30

[1]1,4-c-diphenyldiene E_{tot}(RPBE-D3(Acetonitrile)/def2SVP/W06) = -616.88422827 $\langle S^2 \rangle$ = 0.0000

C	0.003016	3.014320	0.092125
C	-0.065477	3.798292	-1.091267
C	0.058509	3.700800	1.334036
C	-0.075482	5.196132	-1.029393
C	0.048138	5.101010	1.394715
C	-0.018665	5.857695	0.212592
H	-0.113266	3.306053	-2.074374
H	0.111307	3.113477	2.264830
H	-0.129312	5.780577	-1.960991
H	0.092461	5.605841	2.372124
H	-0.027307	6.957496	0.255864
C	0.018665	1.552286	0.092840
H	0.058682	1.095807	1.097711

C	-0.004980	0.726252	-0.998107
H	-0.029975	1.186175	-2.000410
C	0.004980	-0.726252	-0.998107
H	0.029975	-1.186175	-2.000410
C	-0.018665	-1.552286	0.092840
H	-0.058682	-1.095807	1.097711
C	-0.003016	-3.014320	0.092125
C	-0.058509	-3.700800	1.334036
C	0.065477	-3.798292	-1.091267
C	-0.048138	-5.101010	1.394715
C	0.075482	-5.196132	-1.029393
C	0.018665	-5.857695	0.212592
H	-0.111307	-3.113477	2.264830
H	0.113266	-3.306053	-2.074374
H	-0.092461	-5.605841	2.372124
H	0.129312	-5.780577	-1.960991
H	0.027307	-6.957496	0.255864

30

[1]1,4-d-diphenyldiene E_{tot}(RPBE-D3(Acetonitrile)/def2SVP/W06) = -616.87666862 $\langle S^2 \rangle$ = 0.0000

C	2.159561	-0.161330	-0.201032
C	3.235613	-0.821801	0.449461
C	2.408457	1.123739	-0.749790
C	4.497514	-0.223636	0.539578
C	3.672521	1.722831	-0.657557
C	4.725093	1.051676	-0.012867
H	3.080877	-1.816659	0.893958
H	1.586268	1.654772	-1.255871
H	5.316086	-0.755647	1.048722
H	3.837403	2.720955	-1.091948
H	5.719133	1.518454	0.061729
C	0.817113	-0.730828	-0.325398
H	0.084690	-0.080380	-0.833139
C	0.397729	-1.958443	0.100590
H	1.142763	-2.640965	0.547147
C	-0.929456	-2.539078	-0.065857
H	-0.920548	-3.641984	-0.136685
C	-2.169279	-1.968458	-0.176098
H	-2.985503	-2.668035	-0.430599
C	-2.622642	-0.578856	0.004982
C	-3.781719	-0.146117	-0.687942
C	-2.007533	0.335757	0.898169
C	-4.279455	1.156244	-0.532579
C	-2.512990	1.630884	1.064025
C	-3.645132	2.053727	0.341957
H	-4.293060	-0.848743	-1.365355
H	-1.128831	0.017177	1.477151
H	-5.173990	1.469330	-1.092985
H	-2.021743	2.319146	1.769367
H	-4.035920	3.074716	0.470657

30

[1]1,4-e-diphenyldiene E_{tot}(RPBE-D3(Acetonitrile)/def2SVP/W06) = -616.87515226 $\langle S^2 \rangle$ = 0.0000

C	1.772460	-0.557733	0.036484
C	1.148755	-0.085547	-1.146300
C	2.600389	0.344052	0.753995
C	1.322676	1.237007	-1.572520
C	2.757308	1.673420	0.338823
C	2.113918	2.128362	-0.825423
H	0.527847	-0.771525	-1.738478
H	3.112569	-0.008963	1.663500
H	0.828415	1.579758	-2.494732
H	3.390014	2.358481	0.924126
H	2.236971	3.171245	-1.155746
C	1.620630	-1.923302	0.547818
H	2.447517	-2.245122	1.205443
C	0.635323	-2.856323	0.361824
H	0.862628	-3.843836	0.805324
C	-0.635359	-2.856322	-0.361810
H	-0.862684	-3.843849	-0.805271
C	-1.620648	-1.923285	-0.547835
H	-2.447529	-2.245095	-1.205471
C	-1.772468	-0.557717	-0.036506
C	-2.600390	0.344088	-0.753999
C	-1.148748	-0.085549	1.146280
C	-2.757292	1.673452	-0.338807

C	-1.322656	1.236998	1.572523
C	-2.113898	2.128371	0.825444
H	-3.112574	-0.008906	-1.663509
H	-0.527839	-0.771538	1.738442
H	-3.389997	2.358525	-0.924096
H	-0.828382	1.579731	2.494735
H	-2.236946	3.171250	1.155785

30

[1]1,3-a-diphenyldiene E_{tot}(RPBE-D3(Acetonitrile)/def2SVP/W06) = -616.87963988 $\langle S^2 \rangle$ = 0.0000

C	-2.194565	-0.066263	-0.067645
C	-3.155925	0.951469	0.167684
C	-2.670602	-1.378065	-0.322640
C	-4.526119	0.666198	0.144660
C	-4.043128	-1.663331	-0.345789
C	-4.979070	-0.641906	-0.112517
H	-2.826531	1.980927	0.374802
H	-1.941117	-2.183308	-0.505734
H	-5.252385	1.472538	0.330788
H	-4.384633	-2.690487	-0.546891
H	-6.057492	-0.861735	-0.128934
C	-0.747925	0.169597	-0.058012
H	-0.130569	-0.728724	-0.229310
C	-0.111913	1.361997	0.125937
H	-0.713522	2.275974	0.271243
C	1.336018	1.582707	0.110120
C	1.829331	2.854719	0.122066
H	2.912533	3.049227	0.120839
H	1.153997	3.725153	0.139161
C	2.276827	0.420723	0.066283
C	3.290403	0.357354	-0.916109
C	4.200846	-0.711015	-0.943637
C	4.115469	-1.736415	0.012687
C	3.113819	-1.684622	0.997336
C	2.201344	-0.618877	1.022532
H	1.427051	-0.580307	1.803879
H	3.044191	-2.479402	1.756099
H	4.827792	-2.575472	-0.009114
H	4.979418	-0.744407	-1.721442
H	3.352403	1.154408	-1.673125

30

[1]1,3-b-diphenyldiene E_{tot}(RPBE-D3(Acetonitrile)/def2SVP/W06) = -616.87678566 $\langle S^2 \rangle$ = 0.0000

C	2.551578	0.260218	-0.055978
C	2.760840	-0.931530	0.686009
C	3.674608	0.850633	-0.689511
C	4.033737	-1.506225	0.779608
C	4.950104	0.276120	-0.593354
C	5.136172	-0.907004	0.140843
H	1.915691	-1.412992	1.200973
H	3.534329	1.778025	-1.267687
H	4.171273	-2.431137	1.360863
H	5.804809	0.755351	-1.095232
H	6.135845	-1.361005	0.219198
C	1.240254	0.901783	-0.198011
H	1.245178	1.851625	-0.761814
C	0.045030	0.440111	0.262688
H	0.005729	-0.544619	0.760581
C	-1.246327	1.141707	0.137029
C	-1.329682	2.502106	0.128819
H	-2.287896	3.019113	-0.030871
H	-0.437822	3.129193	0.279437
C	-2.468330	0.291043	0.026561
C	-3.711458	0.709442	0.558468
C	-4.857048	-0.088619	0.429440
C	-4.786670	-1.328871	-0.229150
C	-3.558532	-1.763674	-0.754493
C	-2.412259	-0.964548	-0.624427
H	-1.458585	-1.309789	-1.053320
H	-3.491531	-2.732558	-1.273131
H	-5.685322	-1.957269	-0.325825
H	-5.811366	0.255105	0.857719
H	-3.772426	1.664792	1.101864

30

[1]1,3-c-diphenyldiene E_{tot}(RPBE-D3(Acetonitrile)/def2SVP/W06) = -616.87370621 $\langle S^2 \rangle$ = 0.0000

C	1.464120	-1.130341	-0.024872
C	1.174860	-0.305032	-1.139404
C	2.624312	-0.841290	0.735329
C	1.984307	0.796339	-1.448297
C	3.430247	0.264979	0.430874
C	3.108916	1.093925	-0.658264
H	0.310089	-0.542257	-1.775014
H	2.880166	-1.487158	1.590466
H	1.737724	1.426227	-2.317022
H	4.317500	0.481008	1.045988
H	3.741928	1.961545	-0.899909
C	0.628487	-2.284067	0.343958
H	1.191045	-3.147329	0.742680
C	-0.725174	-2.446295	0.267555
H	-1.097836	-3.454814	0.517833
C	-1.791220	-1.487884	-0.083598
C	-2.982330	-1.964638	-0.544569
H	-3.822775	-1.290957	-0.771077
H	-3.141310	-3.044026	-0.697124
C	-1.614003	-0.020407	0.139400
C	-2.090128	0.920951	-0.800964
C	-1.935939	2.298506	-0.582975
C	-1.306153	2.763144	0.583974
C	-0.837281	1.837797	1.532627
C	-0.987002	0.461550	1.311162
H	-0.608504	-0.257402	2.053107
H	-0.346017	2.191357	2.452378
H	-1.178029	3.843450	0.752576
H	-2.300869	3.014090	-1.336026
H	-2.559225	0.562835	-1.730456

30

[1]1,3-d-diphenyldiene E_{tot}(RPBE-D3(Acetonitrile)/def2SVP/W06) = -616.87071654 $\langle S^2 \rangle$ = 0.0000

C	2.267259	-0.476944	-0.397350
C	1.916919	0.854144	-0.058513
C	3.642691	-0.813328	-0.431973
C	2.902497	1.796908	0.260375
C	4.629267	0.128292	-0.101666
C	4.263318	1.437828	0.250116
H	0.858703	1.152313	-0.060227
H	3.937102	-1.836087	-0.717122
H	2.607424	2.827248	0.513087
H	5.691237	-0.161306	-0.126567
H	5.034822	2.181157	0.503236
C	1.273338	-1.501154	-0.772433
H	1.649565	-2.259814	-1.481876
C	-0.022305	-1.637172	-0.383315
H	-0.607172	-2.431800	-0.882718
C	-0.769036	-0.872907	0.648001
C	-0.290326	-0.766139	1.914776
H	-0.823619	-0.195674	2.690759
H	0.664850	-1.234563	2.195807
C	-2.075418	-0.286629	0.234663
C	-3.134116	-0.089679	1.154034
C	-4.343809	0.488194	0.745541
C	-4.528979	0.877390	-0.593514
C	-3.493048	0.678227	-1.520726
C	-2.282286	0.098132	-1.111762
H	-1.470768	-0.047674	-1.841969
H	-3.627223	0.977026	-2.571882
H	-5.481554	1.326954	-0.913334
H	-5.155472	0.626090	1.476643
H	-3.013688	-0.414848	2.198634

30

[1]2,3-a-diphenyldiene E_{tot}(RPBE-D3(Acetonitrile)/def2SVP/W06) = -616.87193278 $\langle S^2 \rangle$ = 0.0000

H	2.535455	0.444382	2.692154
C	1.436833	0.497135	2.638695
H	0.908015	0.969978	3.479922
C	0.749229	-0.005984	1.581475
C	-0.749229	0.005984	1.581475
C	-1.436833	-0.497135	2.638695
H	-2.535455	-0.444382	2.692154
H	-0.908015	-0.969978	3.479922

C	-1.436833	0.599939	0.399305
C	-2.705145	0.141007	-0.028511
C	-3.352210	0.729444	-1.124529
C	-2.743473	1.786909	-1.822996
C	-1.479592	2.247238	-1.416695
C	-0.831965	1.657075	-0.321333
H	0.156496	2.026418	-0.007615
H	-0.992473	3.073075	-1.957888
H	-3.249385	2.244653	-2.686895
H	-4.335506	0.350954	-1.444121
H	-3.177764	-0.705986	0.491986
C	1.436833	-0.599939	0.399305
C	0.831965	-1.657075	-0.321333
C	2.705145	-0.141007	-0.028511
C	1.479592	-2.247238	-1.416695
C	3.352210	-0.729444	-1.124529
C	2.743473	-1.786909	-1.822996
H	-0.156496	-2.026418	-0.007615
H	3.177764	0.705986	0.491986
H	0.992473	-3.073075	-1.957888
H	4.335506	-0.350954	-1.444121
H	3.249385	-2.244653	-2.686895

30

[1]2,3-b-diphenyldiene E_{tot}(RPBE-D3(Acetonitrile)/def2SVP/W06) = -616.87049554 $\langle S^2 \rangle$ = 0.0000

H	2.654361	-0.860399	-0.653924
C	1.836674	-0.123697	-0.656305
H	2.095118	0.921021	-0.884928
C	0.557252	-0.496601	-0.383515
C	-0.557252	0.496601	-0.383515
C	-1.836674	0.123697	-0.656305
H	-2.654361	0.860399	-0.653924
H	-2.095118	-0.921021	-0.884928
C	-0.244440	1.931822	-0.110970
C	-0.780108	2.955865	-0.924967
C	-0.530666	4.307666	-0.640599
C	0.259077	4.662843	0.465500
C	0.800932	3.654983	1.282418
C	0.557252	2.304248	0.994071
H	0.983906	1.520375	1.638245
H	1.418117	3.923533	2.153766
H	0.456969	5.722628	0.688200
H	-0.950479	5.088716	-1.293274
H	-1.386605	2.681584	-1.801884
C	0.244440	-1.931822	-0.110970
C	-0.557252	-2.304248	0.994071
C	0.780108	-2.955865	-0.924967
C	-0.800932	-3.654983	1.282418
C	0.530666	-4.307666	-0.640599
C	-0.259077	-4.662843	0.465500
H	-0.983906	-1.520375	1.638245
H	1.386605	-2.681584	-1.801884
H	-1.418117	-3.923533	2.153766
H	0.950479	-5.088716	-1.293274
H	-0.456969	-5.722628	0.688200
